# Diversity-oriented synthesis of heterocycles and macrocycles by controlled reactions of oxetanes with α-iminocarbenes†
†Electronic supplementary information (ESI) available: Synthetic protocols, ^1^H/^13^C NMR and HR mass spectra are provided. CCDC 1443618–1443620 and 1534812–1534815. For ESI and crystallographic data in CIF or other electronic format see DOI: 10.1039/c7sc00964j



**DOI:** 10.1039/c7sc00964j

**Published:** 2017-06-12

**Authors:** Alejandro Guarnieri-Ibáñez, Florian Medina, Céline Besnard, Sarah L. Kidd, David R. Spring, Jérôme Lacour

**Affiliations:** a Department of Organic Chemistry , University of Geneva , quai Ernest Ansermet 30 , CH-1211 Geneva 4 , Switzerland; b Laboratory of Crystallography , University of Geneva , quai Ernest Ansermet 24 , CH-1211 Geneva 4 , Switzerland; c Department of Chemistry , University of Cambridge , Lensfield Road , Cambridge , CB2 1EW , UK

## Abstract

Using *N*-sulfonyl triazoles as substrates, compounds as diverse as 2-imino tetrahydrofurans, 13- and 15-membered ring aza-macrocycles can be prepared selectively *via* formal [1 + 4], [5 + 4 + 4] and [3 + 4 + 4 + 4] condensations of α-imino carbenes and oxetanes under Rh(ii)-catalysis or thermal activation. Spirocyclic N-heterocycles are also accessible by means of Buchwald–Hartwig and Pictet–Spengler cyclizations. By reaction control, substrate selection or further derivatization, a large variety of chemical structures is thus achievable. Finally, using triazoles reacting under thermal activation, interesting mechanistic insight was obtained.

## Abstract 2

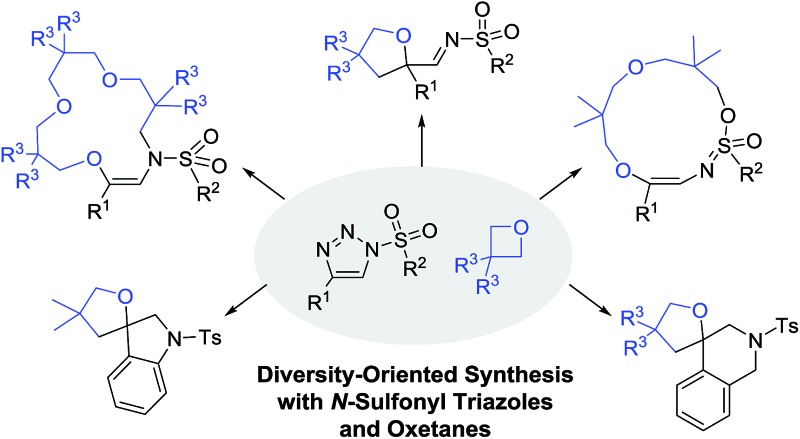
Using *N*-sulfonyl triazoles and oxetanes, a large variety of heterocycles and macrocycles were prepared *via* formal [1 + 4], [5 + 4 + 4] and [3 + 4 + 4 + 4] condensations.

*N*-Sulfonyl-1,2,3-triazoles, **1**, are important building blocks used routinely in synthetic, biological and medicinal chemistry.^1^ These compounds are readily accessible through the Cu(i)-catalyzed azide alkyne cycloadditions (CuAACs) of alkynes and sulfonyl azides.^2^ Importantly, *N*-sulfonyl-triazoles, **1**, are in equilibrium with α-diazo imines of type **1′** that decompose under metal-catalyzed conditions to afford electrophilic α-imino carbenes, **2** (Scheme 1, top).^3^ These intermediates have received much attention in recent years as they afford synthetically useful and original processes, from migrations to ylide forming reactions and subsequent transformations.^4,5^ In terms of oxonium ylide chemistry, under dirhodium catalysis, classical cyclic ethers like THF exhibit only moderate reactivity when used as substrates.^6^ It is necessary to utilize activated nucleophiles such as acetals (1,3-dioxolanes, 1,3-dioxanes and 1,3,5-trioxane)^7,8^ or epoxides^9^ to yield condensation products (6 to 9-membered rings, Scheme 1, top). Herein, the reactions of sulfonyl triazoles, **1**, with oxetanes, **3**, are reported (Scheme 1, bottom). Under dirhodium catalysis (Rh_2_(*S*-TCPTTL)_4_, 1–2 mol%) and at high temperature (100 °C), either 5-membered or 15-membered ring heterocycles are generated. In fact, using regular concentration conditions (0.1 M in CH_2_Cl_2_), 2-iminotetrahydrofurans, **4**, are formed preferentially while unsaturated aza-macrocycles, **5**, are isolated when using oxetanes as the solvent and with high concentration (1.0 M). These two sets of conditions favor the condensations of one and three oxetane moieties respectively, and hence the formation of compounds **4** and **5** occurs in the [1 + 4] and [3 + 4 + 4 + 4] processes respectively.

**Scheme 1:**
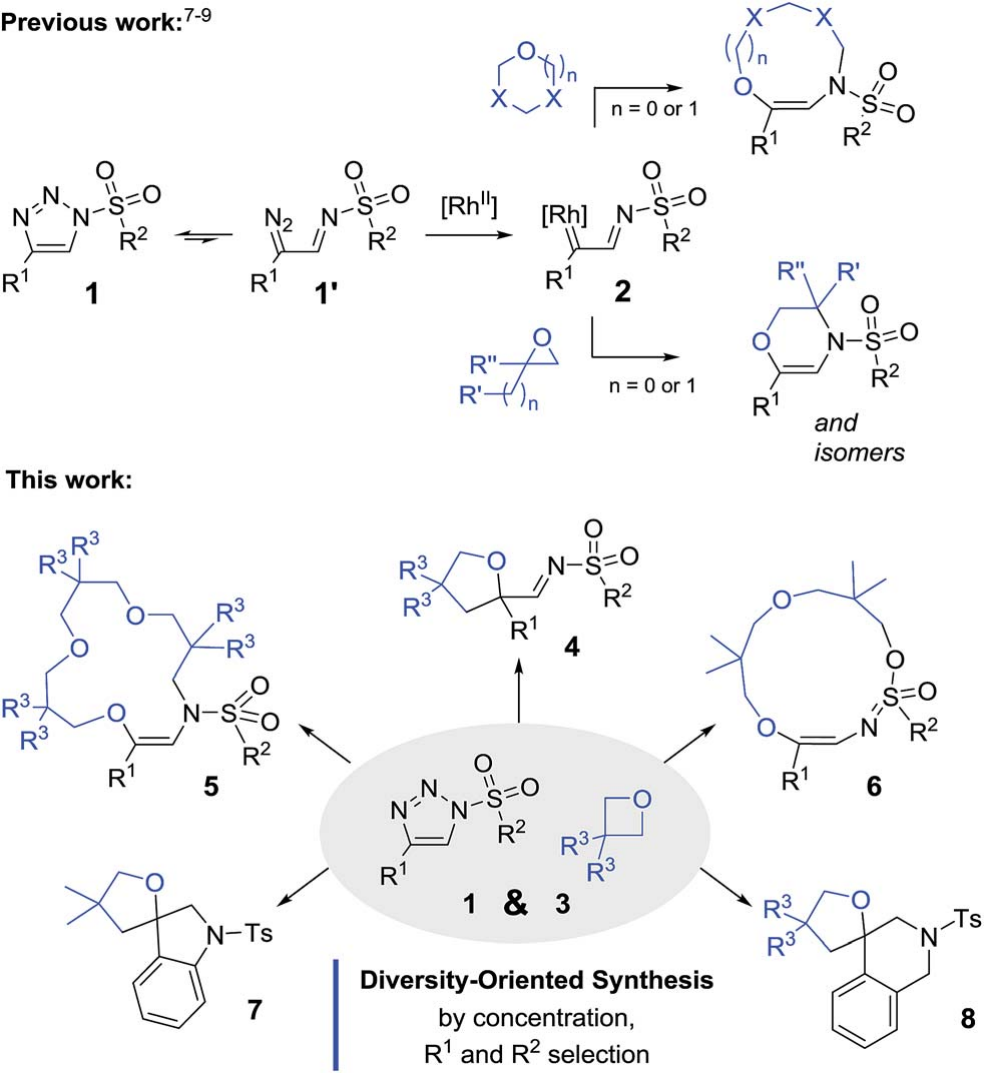
Diversity-oriented synthesis of heterocycles and macrocycles by reaction of *N*-sulfonyl-1,2,3-triazoles, **1**, with cyclic ethers, **3**.

Additionally and interestingly, using alkyl instead of aryl sulfonyl triazoles, interesting 13-membered heterocycles, **6**, are generated by the reaction of two oxetanes with the involvement of a S=O bond as an intramolecular nucleophile. Favorable structural diversity is hence created through control of the reaction conditions or substrate selection. Insight into the mechanistic pathways was also obtained using triazoles reacting under thermal activation specifically.^10,11^ Finally, the straightforward syntheses of spiro N-heterocycles, such as indoline **7** and tetrahydroquinoline **8**, were achieved by means of Buchwald–Hartwig and Pictet–Spengler cyclizations. Compounds **7** and **8** complete effectively the product diversity.^12^

The transformation of oxetanes, **3**, into tetrahydrofurans by reaction with α-keto carbenes was first reported by Noyori and Nozaki, with the reactive carbene intermediates generated *in situ* by the Cu-catalyzed decomposition of diazo ester reagents.^13^ This interesting ring expansion reaction has generated sustained interest in the synthetic organic community.^14^ Drawing from the direct analogy that exists between α-keto carbenes and α-imino intermediates, **2**, it was desirable to test the reactivity of oxetanes in the presence of *N*-sulfonyl-1,2,3-triazoles and dirhodium catalysts. A series of reagents, **1a–1i**, was prepared using CuAAC.^15^ Initial experiments were performed using *N*-tosyl-4-phenyltriazole, **1a** (R^1^ = Ph, R^2^ = *p*Tol). The results of the optimization studies are reported in Table S1.† Rh_2_(*S*-TCPTTL)_4_ was found to be generally effective (1 mol%); complete consumption of **1a** was reached after 3 h at 100 °C using a slight excess of 3,3-dimethyloxetane, **3A** (1.5 equiv.), and CH_2_Cl_2_ as the solvent (0.1 M). ^1^H-NMR spectroscopic analysis of the crude reaction mixture indicated the clean formation of imino products of type **4**. However, and not surprisingly, compound **4aA** was found to be unstable on silica gel or alumina. Product isolation was achieved by reduction with LiAlH_4_ (1.5 equiv., 20 °C). The resulting amino tetrahydrofuran, **9aA**, was isolated in 80% yield over two steps, the structure of which was confirmed by X-ray diffraction analysis (Scheme 2, top).^16^ With these conditions in hand, triazoles **1b–h** were tested with oxetane **3A** to form the corresponding tetrahydrofurans. Substitutions at the *ortho* and *para* positions of the aromatic groups (R^1^) led to the formation of **9bA–9dA** with moderate yields (58–68%). Other R^1^ substituents such as 6-methoxynaphthalene (**1e**) and thiophene (**1f**) were also compatible, leading to the expected products **9eA** and **9fA** in 74% and 75% yields respectively. The mesyl and nosyl derivatives afforded products **9gA** and **9hA** in 71% and 58% yields (R^2^ = Me and *p*NO_2_C_6_H_4_ respectively). Unsubstituted oxetane **3B**, 3,3-bis(chloromethyl) oxetane **3C**, and 2,6-dioxaspiro[3,3]heptane **3D** were found to be reactive with **1a** to afford products **9aB–9aD** in moderate to good yields (44–75%).^17^ Finally, using Davies’ *N*-tosyl-4-ethoxytriazole, **1i**, (R^1^ = OEt, R^2^ = *p*Tol), we could probe the reaction under metal-free conditions.^10*b*^ In fact, and in contrast to the previous derivatives, **1a–1h**, triazole **1i** decomposes by thermal activation only, with the strong π-donor ethoxy group (R^1^) stabilizing the transient donor/acceptor carbene. To our satisfaction, in the absence of Rh_2_(*S*-TCPTTL)_4_, product **9iA** was isolated in 44% yield. Decreasing the thermal activation temperature to 60 °C improved the yield to 50% but at the expense of the reactivity (6 h reaction time).

**Scheme 2:**
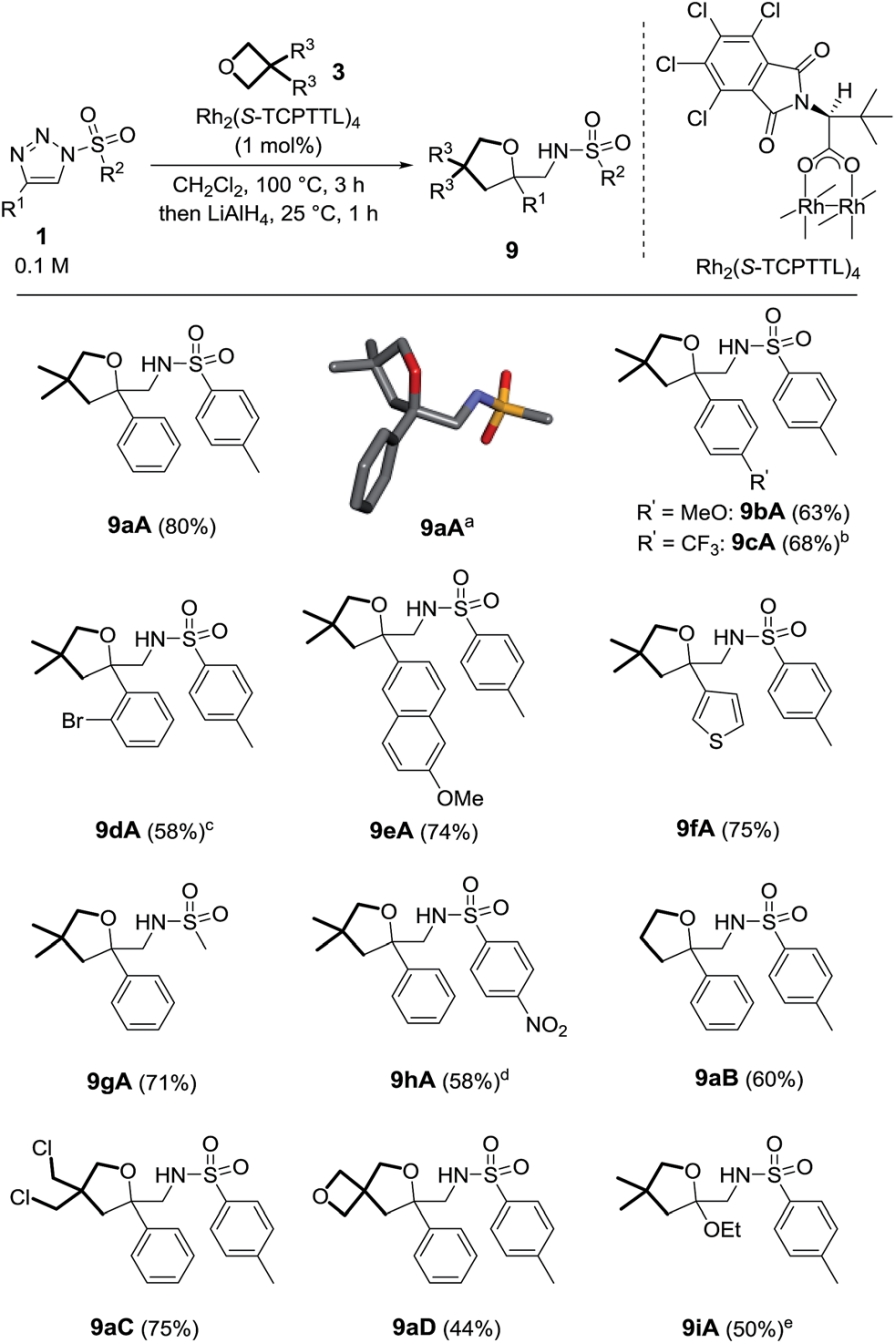
Reaction scope and isolated yields of **9**. (a) Stick view of the crystal structure of **9aA**; part of the tolyl group was removed for clarity. (b) Performed over 6 h. (c) Performed with 2 mol% of Rh_2_(*S*-TCPTTL)_4_ over 14 h. (d) Reduction with LiAlH_4_ at 0 °C over 30 min. (e) Metal-free at 60 °C over 6 h.

While optimizing the reaction conditions for the formation of products **9** (Table S1†), it was noticed that the concentration of **1** and the ratio between **1** and **3** were strongly influencing the formation of the oxolane product. For instance, using oxetane **3A** as the solvent, we observed a variety of other products in the crude reaction mixtures, including a large proportion of compounds **5** while these derivatives were absent under 0.1 M conditions; the nature of the byproducts will be discussed later in the manuscript.^18^ NMR spectroscopic analysis of **5** was compatible only with a macrocyclic structure made by the addition of three oxetane units onto one α-imino carbene moiety.^19,20^

The exact structure of compounds **5** was confirmed later by X-ray diffraction analysis (Scheme 3).^21^ Considering that the synthesis of functionalized macrocycles remains a challenge, particularly under high concentration conditions,^22^ it was deemed interesting to further study the one-pot synthesis of such 15C4 derivatives. Optimization experiments were performed using *N*-bromophenylsulfonyl-4-phenyltriazole **1k** (R^1^ = Ph, R^2^ = *p*BrC_6_H_4_) and the results are reported in Table S2.† With Rh_2_(OAc)_4_ as the catalyst (1 mol%) and 3,3-dimethyloxetane **3A** as the solvent, complete consumption of triazole **1k** (0.5 M) was reached after 5 h at 100 °C to form **5kA** in 28% yield. Higher concentration conditions afforded better catalytic activity, higher conversions and similar yields for the same amount of time. With **1k**, the highest yield (46%) was afforded using Rh_2_(*S*-TCPTTL)_4_ with a loading of 1 mol% in pure 3,3-dimethyloxetane.^23^ Such a yield could appear low in comparison to those obtained in the previous transformation (**1** → **9**, Scheme 2) but, in regard to the interesting poly-condensation [3 + 4 + 4 + 4] mechanism, it is quite remarkable.

**Scheme 3:**
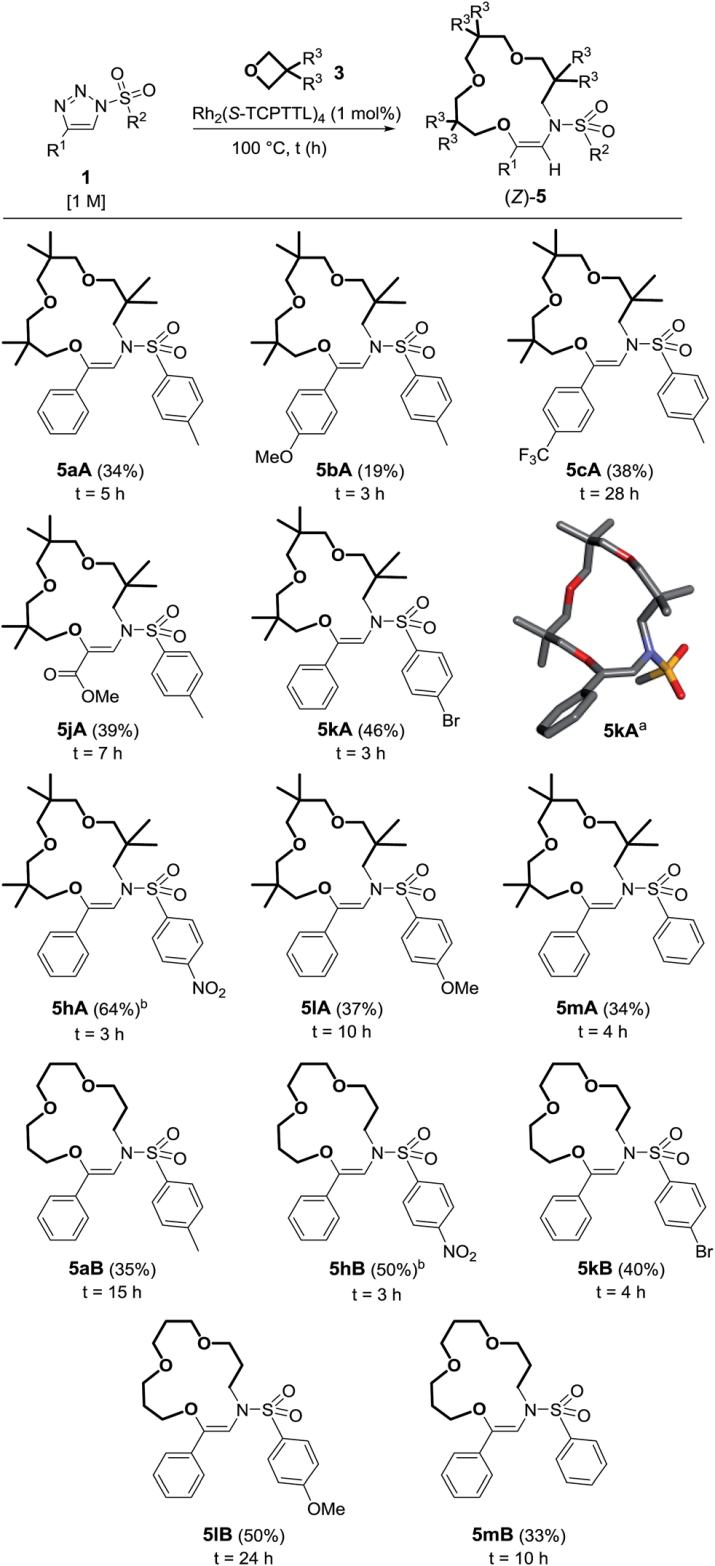
Reaction scope and yields of **5**. (a) Stick view of the crystal structure of **5kA**; part of the *p*BrC_6_H_4_ group was removed for clarity. (b) Performed with 2 mol% of Rh_2_(*S*-TCPTTL)_4_.

With these conditions in hand, **5aA** was obtained in 34% yield (Scheme 3). Substitutions at the *para* position of the aromatic group R^1^ led to the formation of **5bA** ^24^ and **5cA** in 19% and 38% yields respectively. Ester **1j** was also compatible with this transformation, leading to the formation of **5jA** in 39% yield. Nosyl derivatives afforded **5hA** in 57% yield. Increasing the catalyst loading to 2 mol% did not change the reaction time but did improve the results (64% yield). Slower reactions and lower yields were observed for macrocycles **5lA** and **5mA** (37% and 34% respectively). With unsubstituted oxetane **3B**, similar observations were made. Prolonged reaction times were necessary for making **5aB**, **5lB** and **5mB** (24 h, 10 h and 15 h), with the macrocycles being obtained in moderate yields (33–50%). **5hB** and **5kB** were afforded in 50% and 40% yields respectively.

With reagents **1a**, **1l** and **1m** (R^2^ = *p*Tol, *p*MeOC_6_H_4_ or Ph), ^1^H-NMR spectroscopic and GC-MS analysis of the crude reaction mixtures revealed the additional presence of imines, **4**, and two (by)products. Care was taken to isolate these moieties as detailed in Scheme 4 for the reaction of **1a** with **3A**. After careful analysis, 11- and 13-membered rings **10aA** and **6aA** from double oxetane addition were identified. They were generated in 5% and 11% yields respectively, while imine **4aA** was present in low quantities (8% NMR yield).

**Scheme 4:**
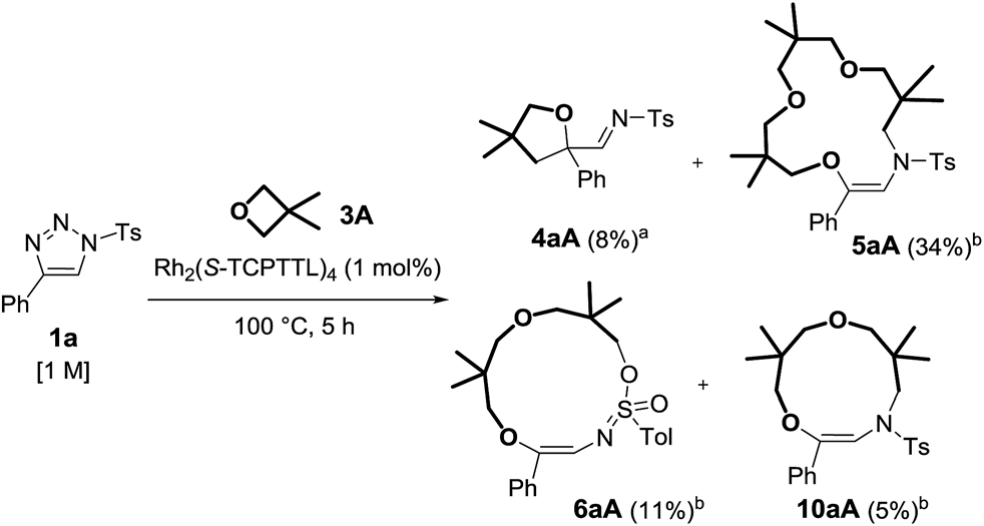
Observed products from the reaction of **1a** and **3A**. (a) NMR yield determined by ^1^H NMR spectroscopy using 1,3,5-trimethoxybenzene as a reference. (b) Isolated yields.

The occurrence of compounds of type **6** was intriguing, particularly the presence of the intracyclic sulfonimidate group. To our knowledge, there have been only a few reports detailing the direct involvement of sulfonyl groups in α-imino carbene chemistry.^25^ An effort was therefore made to improve the synthesis of this type of macrocycle. As already observed in the literature,^8^ triazoles bearing methyl (alkyl) sulfonyl groups may afford different reactivity. This was also fortunately the case as triazole **1g** yielded the 13-membered heterocycle **6gA** predominantly (57% yield, Scheme 5). The exact structure of **6gA** was confirmed by X-ray diffraction analysis. The reaction was performed with **1n** and **1o** to afford **6nA** and **6oA** in 48% and 61% yields respectively. In the case of trifluoromethylated **1n**, it was necessary to use 2 mol% of catalyst. In all cases, macrocycles of type **5** were present in the crude reaction mixture but in low yields (10–20%).^26,27^

**Scheme 5:**
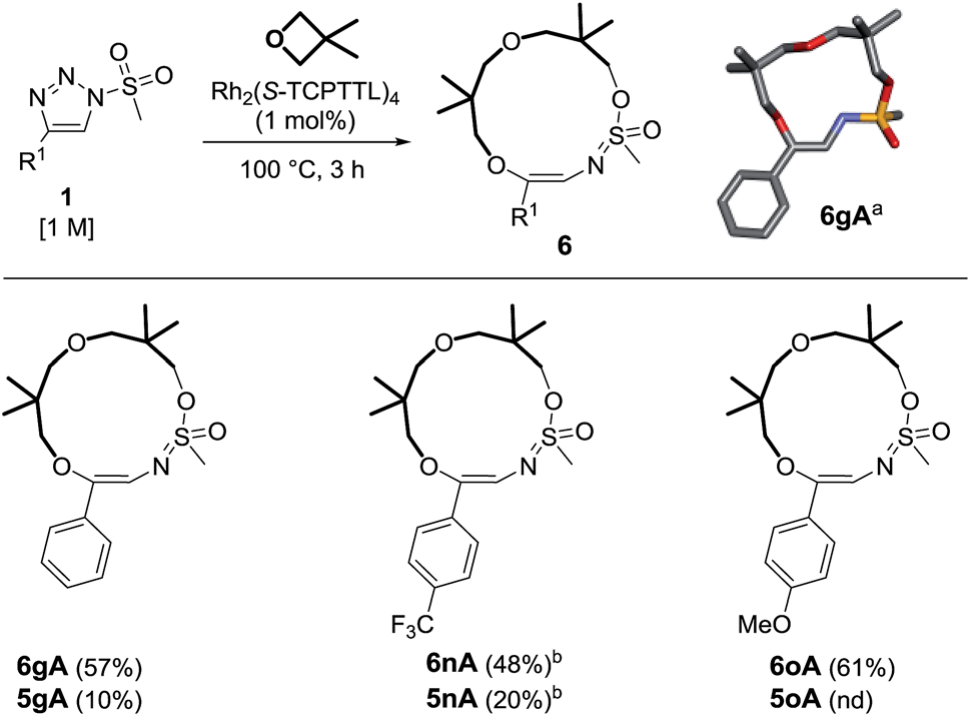
Reactivity of mesyl triazoles and 3,3-dimethyloxetane. Reagents and conditions: (a) stick view of the crystal structure of **6gA**. (b) Performed with 2 mol% of Rh_2_(*S*-TCPTTL)_4_ over 7 h.

At this stage, to gain some insight into the reaction mechanism, and into the influence (or lack thereof) of the metal catalyst on the macrocyclization steps, reactions were performed under thermal activation. Following the report of Alford and Davies on metal-free decompositions,^10*a*^ *N*-sulfonyl-triazoles **1p** and **1q** bearing a phthalimido R^1^ group were prepared (R^2^ = C_6_H_5_CH_2_ or *p*MeOC_6_H_4_). These activated substrates were heated at 60 °C (1 M concentration in 3,3-dimethyloxetane). Full conversion was achieved after only 2 hours and the macrocyclic products **5p** and **5q** were isolated (Scheme 6).^28^ Surprisingly however, mixtures of *Z* and *E*-isomers were obtained, the configurations of which were ascertained by X-ray structural analysis (Fig. S1 and further details in the ESI†). The *Z*-configured products were found to be predominant (ratio ∼ 3 : 1). The minor products, (*E*)-**5p** and (*E*)-**5q**, exhibit a *trans* orientation of the O and N-atoms across the double bond. Remarkably, preliminary calculations (DFT/BP86) indicate that the *E*-isomers of **5p** and **5q** are more stable than the *Z*-analogues by ∼2.1 kcal mol^–1^.^29^ This trend is attributed to a lower ring strain in the *E*-configured 15-membered cycles than in the *Z* geometries. A series of experiments was performed to possibly pinpoint the origin of this disparity. Firstly, the macrocyclization reaction of triazole **1p** was performed in the presence of 1 mol% of Rh_2_(*S*-TCPTTL)_4_, and neither the yield nor the (*Z*) : (*E*) ratio were modified by the presence of the metal complex.^30^ The pure isolated products, (*Z*)-**5p** and (*E*)-**5p**, were also heated at 60 °C in 3,3-dimethyloxetane for 15 h, in the absence or presence of Rh_2_(*S*-TCPTTL)_4_ (1 mol%). In these reactions, and those performed under the same conditions at 100 °C, evidence of the isomerization of either the (*Z*) or (*E*)-isomers of **5p** could not be found. These results, combined with our calculations, indicate that products **5p** and **5q** are produced under kinetic control (no equilibration was observed).

**Scheme 6:**
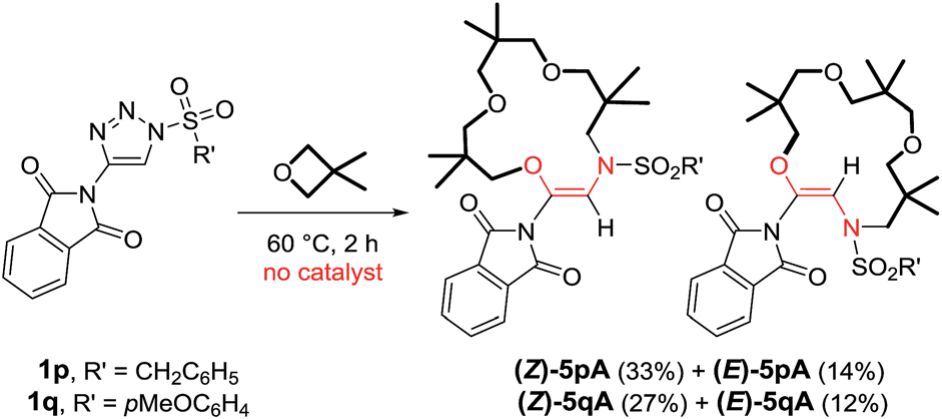
Reactivity of *N*-sulfonyl-4-phthalimido-1,2,3-triazoles **1p** and **1q** with 3,3-dimethyloxetane.

The mechanistic rationales are outlined in Schemes 7 and 8. As discussed, in the presence of Rh(ii) catalysts, *N*-sulfonyl triazoles, **1**, generate α-imino metal carbenes, **2**, by nitrogen extrusion. These electrophilic intermediates react with one molecule of oxetane to form oxonium ylides of type **11**. The high nucleophilicity of the O-atom in both regular oxetane and 3,3-dimethyloxetane facilitates this step.^31^ Currently, the lack of stereoinduction in the reactions performed with Rh_2_(*S*-TCPTTL)_4_ tends to indicate the formation of metal-free ylides, rather than metal-bound species.

**Scheme 7:**
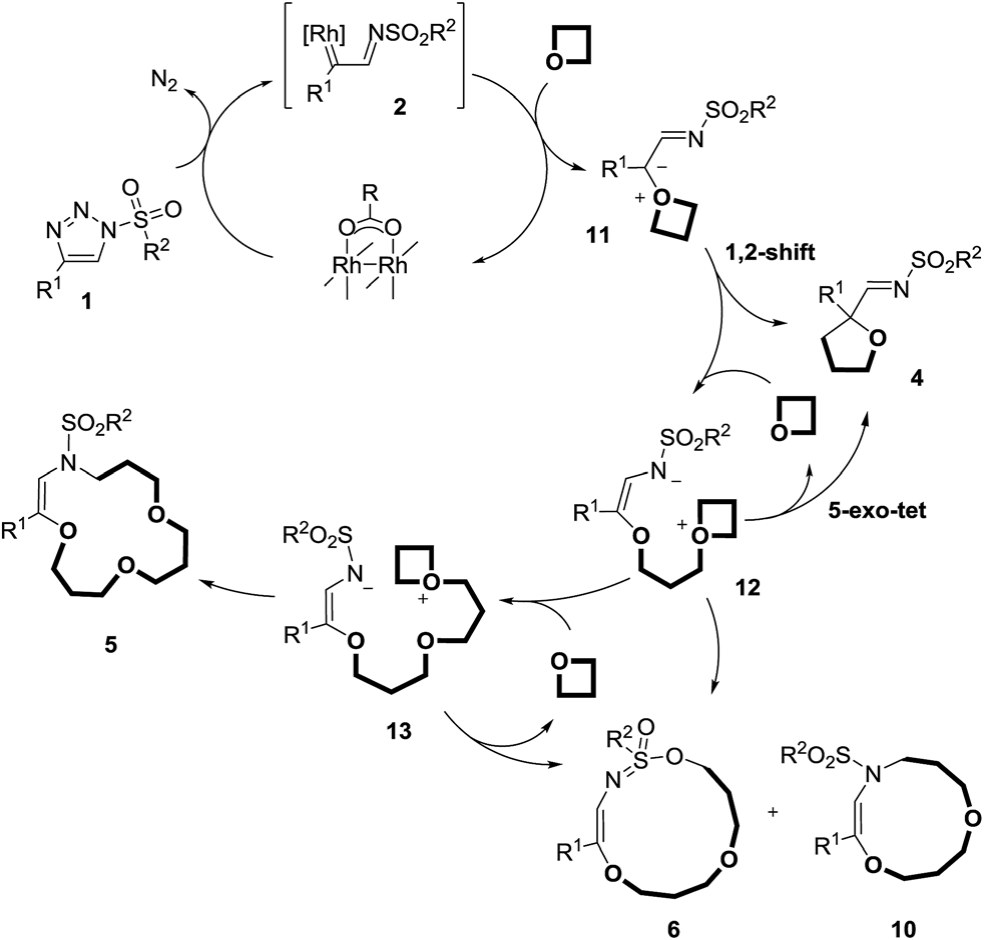
Rationale for the metal catalyzed pathway.

**Scheme 8:**
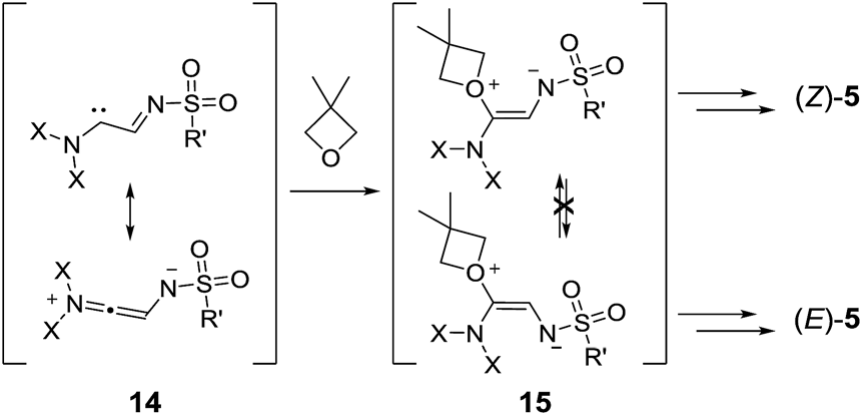
Formation of (*Z*)-**5** and (*E*)-**5**. NX_2_ represents the phthalimido group.

This highly reactive (ring-strained) intermediate can undergo a 1,2-shift and form adducts, **4**; the intramolecular process is favored by “dilute” conditions (0.1 M). At high concentration (1 M conditions), **11** reacts with the best nucleophile in the medium, the oxetane itself,^31*a*^ generating **12**. This intermediate can generate products of type **4** again by means of a 5-*exo*-tet cyclization, or products **6** and **10** *via* 11-*endo*-tet or 9-*endo*-tet cyclizations respectively. A third oxetane addition affords species **13**, which are then trapped intramolecularly by the terminal nitrogen atom, leading to the final products, **5**.^20*d*^ The reasons for the sequential addition of three oxetanes is due primarily to the need for S_N_2-like transition states and/or trans-annular strain.^20*d*^

In thermally activated processes (**1p** and **1q**), the situation is somewhat different. Resonance-stabilized carbene intermediates, **14**, are formed by direct nitrogen extrusion (Scheme 8).^10*a*^ Reactions with one equivalent of oxetane form ylide intermediates, **15**, that are probably configurationally stable and exist in non-interconvertible (*Z*) and (*E*) forms. Further reactions with oxetanes and ring closures led to the formation of the macrocycles; the ratio between products **5pA** and **5qA** reflected the (*Z*) : (*E*) ratio among the intermediates, **15**.

With compounds **5** in hand, hydrogenation conditions were also developed. Not surprisingly, it was necessary to use more forcing conditions with sterically hindered **5A** than with **5B** (Scheme 9). In effect, Pd(OH)_2_ on activated charcoal, Pearlman’s catalyst, was required with adducts made from 3,3-dimethyloxetane, and derivatives of **16A** were obtained in good to excellent yields (92–97%). While compounds carrying methoxy and methyl groups reacted without interference from the functional groups, derivative **5hA**, substituted with a nitro moiety, afforded the amino compound, **16hA**. With **16B**, heterogeneous catalysis with Pd/C was possible and nitro group reduction was again observed. Treatment of N-macrocycles **16aA** and **16aB** with LiAlH_4_ in Bu_2_O at 120 °C resulted in the cleavage of the sulfonyl group,^32^ and free-amino building blocks **17aA** and **17aB** were isolated in 70% and 90% yields (Scheme 9).

**Scheme 9:**
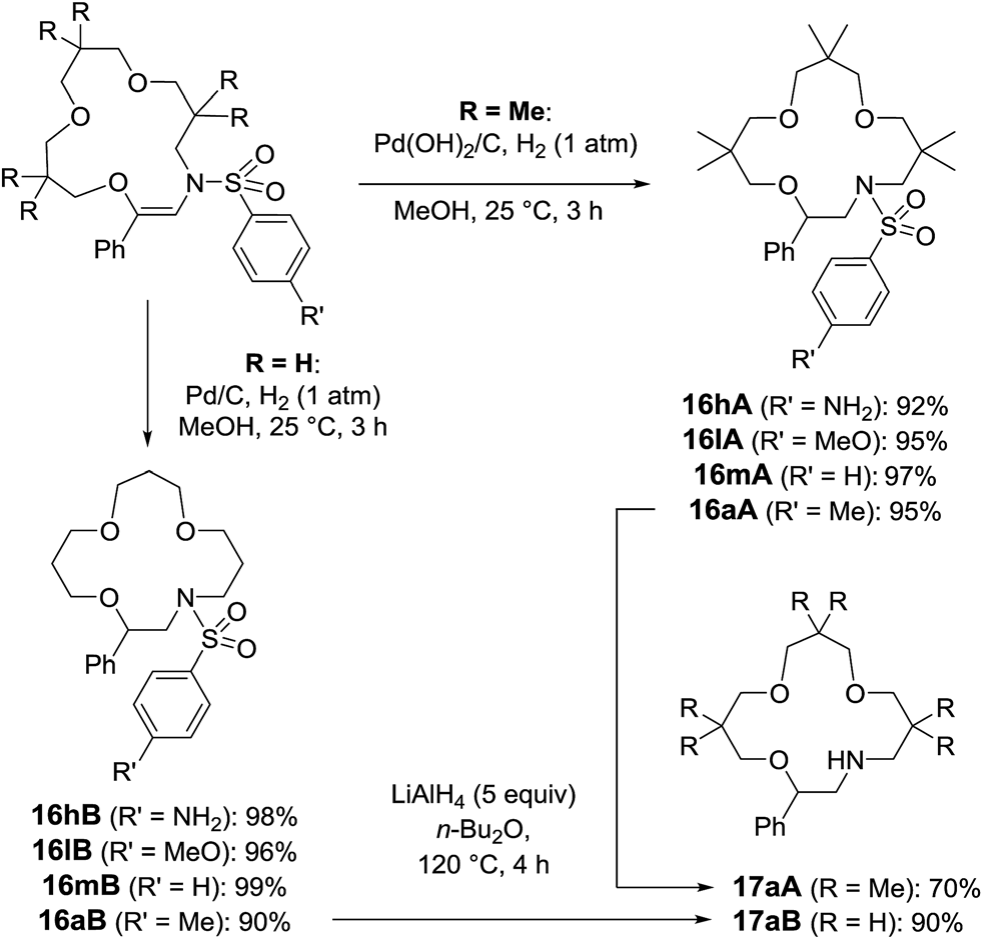
Hydrogenation and tosyl group removal conditions.

Furthermore, care was taken to extend the structural diversity of the products made in this study by examining different trapping protocols for oxolane **4aA** or derivatizations of compounds **9** (Scheme 10). Firstly, it was possible to hydrolyze imine **4aA** to aldehyde **18** or transform it into dithiane^33^ **19** in 69% and 70% yields respectively (in two steps, with no isolation of **4aA** required). In another set of experiments, the nosyl group of **9hA** was successfully removed, leading to the formation of free amine **20** (65% yield).^34^ Direct construction of spiro N-heterocycles was achieved by intramolecular C–N bond formations. For instance, spiroindoline **7** was prepared from **9dA** *via* a Buchwald–Hartwig reaction (86% yield).^35^ 2-Amino tetrahydrofurans **9aA**, **9aB** and **9aC** were reacted with paraformaldehyde under acidic conditions to afford spirotetrahydroquinolines **8A**, **8B** and **8C** in moderate to good yields (65–90%) *via* Pictet–Spengler cyclizations.^5g,36^

**Scheme 10:**
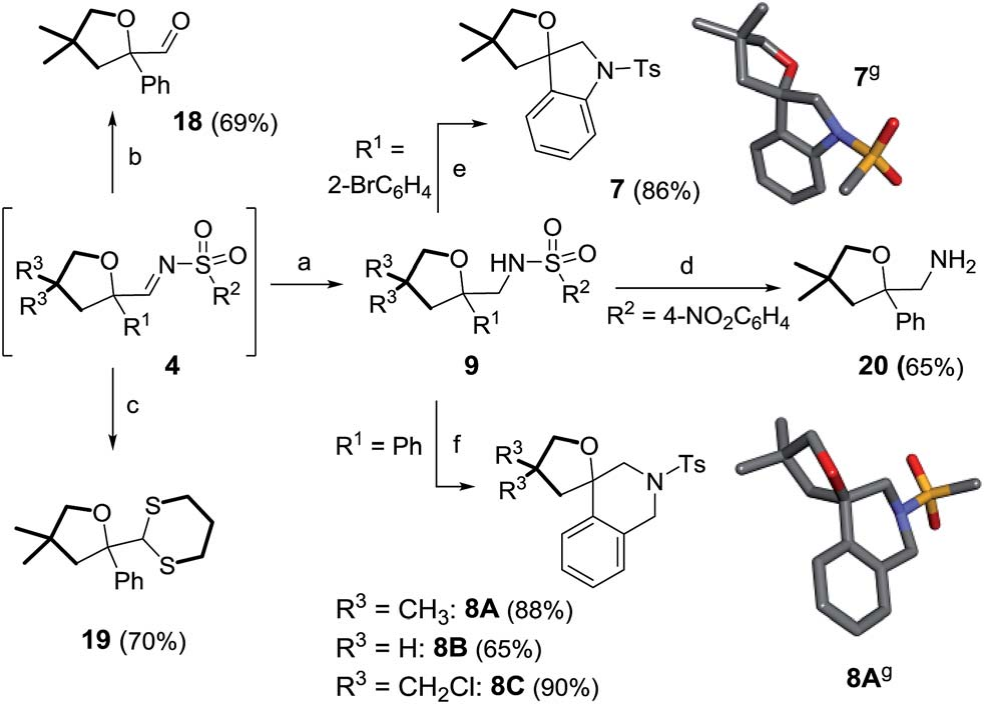
Post-functionalization of **4** or **9**. Reagents and conditions: (a) LiAlH_4_, 25 °C, 1 h. (b) Silica gel, 25 °C, 24 h. (c) HS(CH_2_)_3_SH, ClSiMe_3_, Zn(OTf)_2_, 25 °C, 1 h. (d) PhSH, K_2_CO_3_, CH_3_CN/DMSO (49 : 1), 50 °C, 2 h. (e) Pd(OAc)_2_, ±BINAP, K_2_CO_3_, toluene, 115 °C, 14 h. (f) (HCHO)_*n*_, TFAA, MsOH, 1,2-DCE, 0 °C, 25 min. (g) Stick view of the crystal structures of **7** and **8A**; part of the tolyl groups has been removed for clarity.

Finally, with compounds **5–9** and **16–20** in hand, care was taken to evaluate the structural diversity generated by these derivatives. In fact, geometrically constrained macrocycles and spiro derivatives are attractive and possibly underrepresented targets in drug discovery.^37^ Due to the conformational pre-organization associated with these skeletons, such moieties often display higher affinity and selectivity for biological targets. Herein, using the classical tools of “Diversity-Oriented Synthesis”,^12,38^ we report the chemoinformatic analysis of the diversity of the library, in terms of (i) the molecular shape distribution *via* principal-moment of inertia (PMI) analysis (Fig. 1) and (ii) 15 chemo-physical properties *via* principal component analysis (PCA) (Fig. S3†), in comparison to two reference sets constituting 40 top-selling drugs and 60 natural products (see the ESI† for details). PMI analysis suggested that the DOS library exhibited a broad shape distribution, comparable to that of the natural product reference set, with decreased rod-like features compared to the drug reference set. Notably, macrocycle **5jA** and spirocycle **7** displayed significant ‘spherical’ characteristics, populating the far right-hand side of the plot. Pleasingly, the DOS library also had considerable overlap with both reference sets in the PCA analysis, suggesting that the DOS library accessed attractive areas of chemical space and contained compounds with drug-like properties (Fig. S3†).

**Fig. 1:**
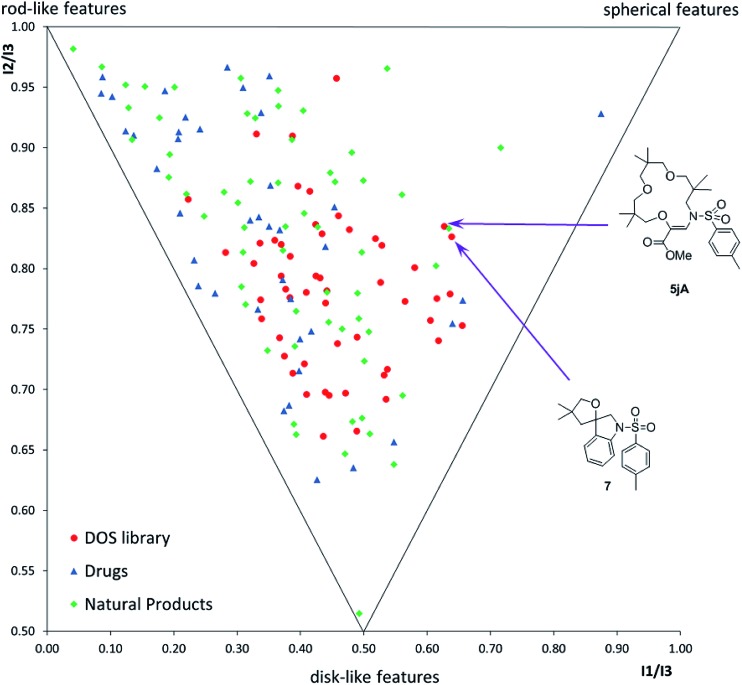
Comparative PMI plot of fifty-three DOS library compounds (red dots), 40 top-selling brand-name drugs (blue triangles) and 60 diverse-natural products (green rhombi).

## Conclusions

In summary, 2-iminotetrahydrofurans, **4**, are readily formed by [1 + 4] condensations of α-imino carbenes and oxetanes under “dilute” conditions. These compounds possess a rather large range of reactivity, from reductions and hydrolyses to conversions into heterocycles such as spiroindolines, **7**, or spirotetrahydroquinolines, **8**. Under high concentration conditions (1 M), however, 13- and 15-azamacrocycles can be obtained *via* formal [5 + 4 + 4] and [3 + 4 + 4 + 4] condensations of α-imino carbenes and two/three oxetanes. In terms of mechanistic information and in the case of macrocycles **5** in particular, the *E* or *Z*-geometry of the unsaturated products was found to depend on the mode of carbene initiation – thermal activation *vs.* metal-catalysis. This afforded an interesting insight into the configurational stability of the subsequent ylide intermediates. Finally, chemoinformatic analysis suggested that the resulting library exhibited a broad molecular shape distribution, with significant spherical shape, covering attractive areas of chemical space.
