# Supplementary material for: Diversity-oriented synthesis of heterocycles and macrocycles by controlled reactions of oxetanes with α-iminocarbenes
Source: Chem Sci. 2017 Jun 12;8(8):5713–20. doi: 10.1039/c7sc00964j (PMC5621157; doi:10.1039/c7sc00964j)
Supplement: Supplementary file 1 [file SC-008-C7SC00964J-s001.pdf]

Electronic Supplementary Material (ESI) for Chemical Science.
This journal is © The Royal Society of Chemistry 2017

Diversity-Oriented Synthesis of Heterocycles and Macrocycles by Controlled Reactions of Oxetanes with α-Iminocarbenes

Alejandro Guarnieri-Ibáñez,[a] Florian Medina,[a] Céline Besnard,[b] Sarah L. Kidd,[c] David R. Spring,[c] and Jérôme Lacour[a,*]

[a] University of Geneva, Department of Organic Chemistry, Quai Ernest Ansermet 30, CH-1211 Geneva 4, Switzerland
[b] Laboratory of Crystallography, University of Geneva, quai Ernest Ansermet 24, CH-1211 Genève 4, Switzerland
[c] Department of Chemistry, University of Cambridge, Lensfield Road, Cambridge, CB2 1EW, UK

jerome.lacour@unige.ch

Supporting Information

Table of Contents

1. General remarks S3
2. Table S1. Initial experiments and optimization studies. Products **4** S3
3. Table S2. Initial experiments and optimization studies. Products **5** S4
4. Figure S1 S5
5. General procedure I: synthesis of *N*-sulfonyl-1,2,3-triazoles S5
Analysis data for unreported triazoles S6
6. General procedure II: synthesis of tetrahydrofurans **9** S6
Analysis data for tetrahydrofurans **9** S7

7. General procedure III: synthesis of macrocycles **5** and **6** S11

Analysis data for macrocycles **5** S11

Analysis data for macrocycles **6** S17

Analysis data for byproducts **6aA** and **10aA** S19

8. General procedure IV: hydrogenation of macrocycles **5** S20

Analysis data for hydrogenated products **16** S20

9. Deprotection of *N*-macrocycles **16aA** and **16aB** S23

10. Synthesis of aldehyde **18** S23

11. Synthesis of dithiane **19** S24

12. Synthesis of amine **20** S24

13. Synthesis of spiroindoline **7** S25

14. Synthesis of spirotetrahydroisoquinoline **8** S25

15. NMR spectra of new compounds S27

16. Computational details S82

17. Crystallographic data S88

18. Structural Diversity Computational Analysis S95

A) Principal Moment of Inertia (PMI) S95

Table S3 S103

Table S4 S105

Table S5 S106

Figure S2 S107

B) Principal component analysis S108

Figure S3 S115

19. References S116

1. General remarks

Unless otherwise stated, reagents were purchased from commercial sources and used without further purification. 2,6-Dioxaspiro[3.3]heptane and 3,3-bis(chloromethyl)oxetane were synthesized according to the reported procedures.[1] NMR spectra were recorded on 400 or 500 MHz spectrometer at 23 °C. ^{1}H-NMR: chemical shifts are given in ppm relative to Me_4Si with solvent resonances used as internal standards ($CDCl_3$ δ = 7.26 ppm or acetone-d$_6$ δ = 2.05 ppm). Data were reported as follows: chemical shift (δ) in ppm on the δ scale, multiplicity (*s* = singulet, *d* = doublet, *t* = triplet, *dd* = doublet of doublet, *q* = quintuplet and *m* = multiplet), coupling constant (Hz) and integration. ^{13}C-NMR: chemicals shifts were given in ppm relative to Me_4Si with solvent resonances used as internal standards ($CDCl_3$ δ = 77.16 ppm or acetone-d$_6$ δ = 29.84 and 206.26 ppm). IR spectra were recorded using an ATR sampler and are reported in wave numbers (cm^{-1}). Melting points (Mp) were measured in open capillary tubes and were uncorrected. Electrospray mass spectra (ESI) were obtained by the department of Mass Spectrometry of the University of Geneva. Flash column chromatography was performed with silica gel 40 - 63 µm or alumina (neutral Brockmann I, 50 - 200 µm).

2. Table S1. Initial experiments and optimization studies. Products 4

In a 2 mL screw-cap vial equipped with a magnetic stirring bar, $Rh_2(L)_4$ (1 mol %), 4-phenyl-*N*-tosyltriazole **1a** (0.05 mmol, 1 equiv) and 3,3-dimethyloxetane **3A** (0.075 mmol, 1.5 equiv) were dissolved in 0.5 mL (0.1 M) of solvent. The vial was flushed with nitrogen, capped and stirred at the corresponding temperature for the corresponding amount of time. The solution was cooled to room temperature and 1,3,5-trimethoxybenzene (0.25 equiv) was added as reference for NMR yield determination.

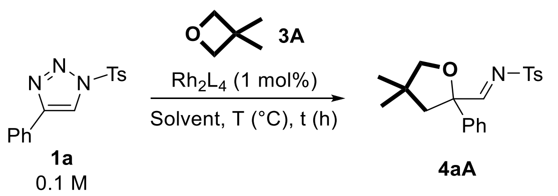

Entry	Solvent	Catalyst	T (°C)	Time (h)	Yield[a]
1	CH_2Cl_2	-	60 °C	24 h	-
2	CH_2Cl_2	-	100 °C	24 h	-
3	CH_2Cl_2	Rh_2(*S*-TCPTTL)$_4$	60 °C	24 h	62%
4	CH_2Cl_2	Rh_2(*S*-TCPTTL)$_4$	80 °C	5 h	70%
5	CH_2Cl_2	Rh_2(*S*-TCPTTL)$_4$	100 °C	3 h	80%
6[b]	CH_2Cl_2	Rh_2(*S*-TCPTTL)$_4$	100 °C	3 h	81%
7	Toluene	Rh_2(*S*-TCPTTL)$_4$	100 °C	3 h	60%
8	$CHCl_3$	Rh_2(*S*-TCPTTL)$_4$	100 °C	3 h	70%[c]
9	1,2-DCE	Rh_2(*S*-TCPTTL)$_4$	100 °C	3 h	50%[c]
10	CH_2Cl_2	$Rh_2(Oct)_4$	100 °C	3 h	75%
11	CH_2Cl_2	$Rh_2(Piv)_4$	100 °C	3 h	75%
12	CH_2Cl_2	Rh_2(*S*-PTTL)$_4$	100 °C	3 h	69%
13	CH_2Cl_2	Rh_2(*R*-DOSP)$_4$	100 °C	3 h	70%
14	CH_2Cl_2	$Rh_2(esp)_4$	100 °C	3 h	79%

[a]Determined by ^{1}H NMR. [b]3 equivalents of oxetane. [c] Partial hydrolysis of the imine.

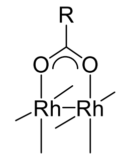

R = *n*-C_7H_{15} : $Rh_2(Oct)_4$
R = *t*-Bu : $Rh_2(Piv)_4$

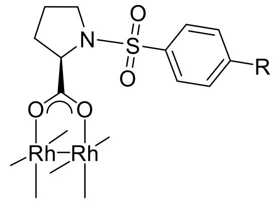

R = *n*-$C_{12}H_{25}$: Rh_2(*R*-DOSP)$_4$

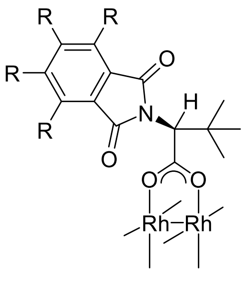

R = H : Rh_2(*S*-PTTL)$_4$
R = Cl : Rh_2(*S*-TCPTTL)$_4$

3. Table S2. Initial experiments and optimization studies. Products 5

In a 2 mL screw-cap vial equipped with a magnetic stirring bar, $Rh_2(L)_4$ (1 mol %) and triazole **1k** (0.1 mmol, 1 equiv) were dissolved in 0.1 mL (1 M) of 3,3-dimethyloxetane. The vial was flushed with nitrogen, capped and stirred at the corresponding temperature for the corresponding amount of time. The solution was cooled to room temperature and 1,3,5-trimethoxybenzene (4.21 mg, 0.25 equiv) was added as reference for NMR yield determination.

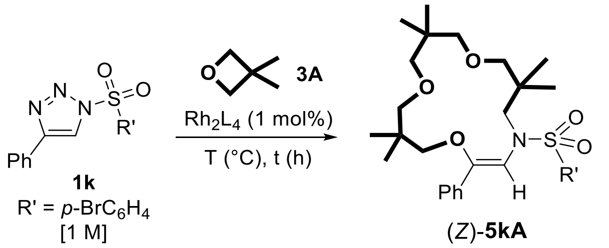

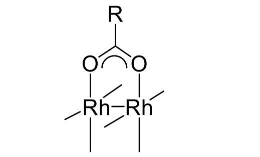

R = Me : $Rh_2(OAc)_4$
R = n-C_7H_{15} : $Rh_2(Oct)_4$
R = t-Bu : $Rh_2(Piv)_4$

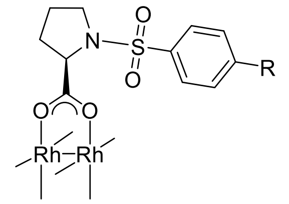

R = n-$C_{12}H_{25}$: Rh_2(R-DOSP$)_4$
R = t-Bu : Rh_2(R-TBSP$)_4$

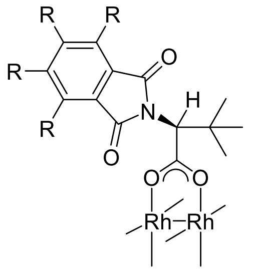

R = H : Rh_2(S-PTTL$)_4$
R = F : Rh_2(S-TFPTTL$)_4$
R = Cl : Rh_2(S-TCPTTL$)_4$

Entry	Solvent	Catalyst	Temperature	Time	Yield[a]
1	-	-	100 °C	24 h	-
2[b]	-	$Rh_2(OAc)_4$	100 °C	5 h	28%
3	-	$Rh_2(OAc)_4$	100 °C	3 h	31%
4	-	$Rh_2(Oct)_4$	100 °C	3 h	36%
5	-	$Rh_2(Piv)_4$	100 °C	3 h	42%
6	-	$Rh_2(esp)_2$	100 °C	3 h	36%
7	-	Rh_2(R-DOSP$)_4$	100 °C	3 h	28%
8	-	Rh_2(R-TBSP$)_4$	100 °C	3 h	27%
9	-	Rh_2(S-PTTL$)_4$	100 °C	13 h	21%
10	-	Rh_2(S-TFPTTL$)_4$	100 °C	3 h	45%
11	-	Rh_2(S-TCPTTL$)_4$	100 °C	3 h	46%
12[c]	-	Rh_2(S-TCPTTL$)_4$	100 °C	3 h	47%
13	-	Rh_2(S-TCPTTL$)_4$	80 °C	10 h	45%
14	CH_2Cl_2	Rh_2(S-TCPTTL$)_4$	100 °C	3 h	36%
15	$CHCl_3$	Rh_2(S-TCPTTL$)_4$	100 °C	3 h	40%
16	Toluene	Rh_2(S-TCPTTL$)_4$	100 °C	3 h	38%

[a]Determined by ^{1}H NMR. [b]Performed at 0.5 M. [c]Performed with 2 mol%.

4. Figure S1

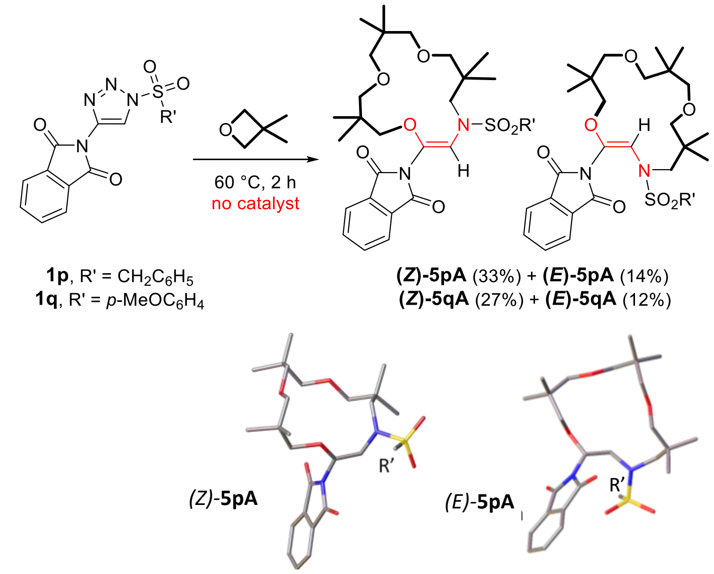

Top: reactivity of the *N*-sulfonyl-4-phthalimido-1,2,3-triazoles **1p** and **1q** with 3,3-diemthyloxetane. Bottom: Stick views of the crystal structures of (*Z*)-**5pA** (left) and (*E*)-**5pA** (right). The benzyl group R' and H-atoms are omitted for clarity.

5. General procedure I: synthesis of *N*-sulfonyl-1,2,3-triazoles

Important note: *Sulfonyl azides are potentially explosive materials and must be handled with caution.*

Azide synthesis: Following the reported procedure,[2] to a stirred solution of sulfonyl chloride (10.0 mmol, 1.0 equiv) in 60 mL water/acetone mixture (1:2), NaN_3 (1.3 equiv, 845 mg) was slowly added at 0 °C. The resulting solution was stirred at room temperature for 12 h. The residue was suspended in 20 mL of Et_2O, the layers were separated and the aqueous phase was extracted with Et_2O (3 x 20 mL). The organic layers were combined, dried over $MgSO_4$, filtered and concentrated under reduced pressure. The desired azide was obtained sufficiently pure to be used without any further purification.

Caution: *Care should be taken to protect the reaction mixture from light at each step of the synthesis of the triazoles.*

Method A:
Following the reported procedure,[3] 0.1 mmol (0.05 equiv, 19 mg) of copper(I) thiophene-2-carboxylate (CuTC) and 2 mmol (1 equiv) of the corresponding sulfonyl azide were diluted in 7 mL of toluene. Then 2.6 mmol (1.3 equiv) of the corresponding alkyne was added and the solution was stirred at room temperature overnight and protected from light. The mixture was diluted with 20 mL of saturated NH_4Cl_{aq} and extracted with EtOAc (3 x 15 mL). The combined organic layers were dried over $MgSO_4$ and concentrated under reduced pressure. The yellowish solid obtained was filtrated over a pad of silica gel or purified by column chromatography (silica gel, pentane/EtOAc) to afford the desired product. Products of type **1** were then stored at -20 °C in the dark under nitrogen atmosphere.
Method B:
Following the reported procedure,[4] 1 mmol (1 equiv, 0.171 g) of *N*-ethynylphthalimide, 1.2 mmol (1.2 equiv) of the corresponding sulfonyl azide and 10 mmol (0.1 equiv, 0.019 g) of CuTC were dissolved in

4 mL of toluene and stirred at room temperature overnight and protected from light. The mixture was filtrated through a short pad of silica gel with acetone and the resulting solid was washed three times with Et_2O, to yield the desired triazole. Products of type **1** were then stored at -20 °C in the dark under nitrogen atmosphere.

Analysis data for unreported triazoles

1-(benzylsulfonyl)-4-phthalimido-1*H*-1,2,3-triazole (1p):

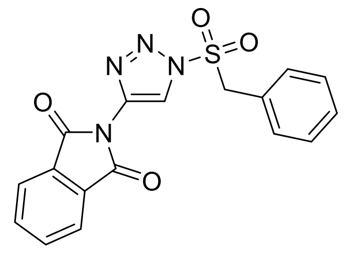

Following general procedure I, Method B: **1p** is obtained as a white solid (0.32 g, 88% yield) starting from the corresponding alpha-toluenesulfonyl azide (0.236 g, 1.2 mmol, 1.2 equiv).

M.p. = 134-136 °C; **Rf** = 0.45 (silica gel, pentane/EtOAc, 1:1); **^{1}H NMR (500 MHz, $CDCl_3$):** δ 7.98 (*dd*, *J* = 5.5, 3.0 Hz, 2H), 7.92 (*s*, 1H), 7.84 (*dd*, *J* = 5.5, 3.1 Hz, 2H), 7.43-7.32 (*m*, 3H), 7.15-7.12 (*m*, 2H), 4.90 (*s*, 2H) ppm; **^{13}C NMR (100 MHz, $CDCl_3$):** δ 165.2 (C), 136.7 (C), 135.2 (CH), 131.6 (C), 130.8 (CH), 130.3 (CH), 129.6 (CH), 125.0 (C), 124.5 (CH), 120.8 (CH), 61.7 (CH_2) ppm; **IR (neat):** 1727, 1568, 1380, 1367, 1171, 1080, 979, 781 cm^{-1}; **HR-MS (ESI):** m/z = 369.0649 $[M+H]^+$ (calculated for $C_{17}H_{13}N_4O_4S$ m/z = 369.0652).

1-(4-methoxybenze)-4-phthalimido-1*H*-1,2,3-triazole (1q):

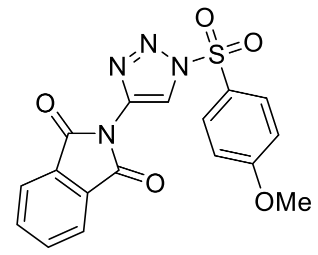

Following general procedure I, Method B: **1q** is obtained as a white solid (0.18 g, 70% yield) starting from the corresponding 4-methoxybenzenesulfonyl azide (0.17 g, 0.8 mmol; 1.2 equiv).

M.p. = 99-101 °C; **Rf** = 0.34 (silica gel, pentane/EtOAc, 1:1); **^{1}H NMR (500 MHz, $CDCl_3$):** δ 8.42 (*s*, 1H), 8.13-8.07 (*m*, 2H), 7.98 (*dd*, *J* = 5.5, 3.1 Hz, 2H), 7.83 (*dd*, *J* = 5.5, 3.0 Hz, 2H), 7.09-7.03 (*m*, 2H), 3.91 (*s*, 3H) ppm; **^{13}C NMR (100 MHz, $CDCl_3$):** 165.9 (C), 165.3 (C), 136.8 (C), 135.1 (CH_2), 131.8 (CH_2), 131.6 (C), 126.4 (C), 124.4 (CH_2), 118.6 (CH), 115.4 (CH_2), 56.2 (CH_3) ppm; **IR (neat):** 1731, 1591, 1572, 1372, 1166, 714 cm^{-1}; **HR-MS (ESI):** m/z = 417.0846 $[M+H+CH_3OH]^+$ (calculated for $C_{18}H_{17}N_4O_6S$ m/z = 417.0863).

6. General procedure II: synthesis of tetrahydrofurans 9

In a 2 mL screw-cap vial equipped with a magnetic stirring bar, Rh_2(*S*-TCPTTL)$_4$ (2.96 mg, 0.0015 mmol, 1 mol%), *N*-sulfonyltriazole **1** (0.15 mmol, 1 equiv) and the corresponding oxetane **3** (0.225 mmol, 1.5 equiv) were dissolved in 1.5 mL of anhydrous CH_2Cl_2 (0.1 M). The vial was flushed with nitrogen, capped and stirred at 100 °C for a specific amount of time (see procedures below). The solution was cooled to 0 °C and $LiAlH_4$ (8.54 mg, 0.225 mmol, 1.5 equiv.) was slowly added. The solution was stirred at room temperature for 1 h. Then, 5 mL of EtOAc were added dropwise followed by 200 µL of H_2O and dried over Na_2SO_4. The solution was filtered, concentrated under reduced pressure and purified by column chromatography.

Analysis data for tetrahydrofurans 9

Compound 9aA:

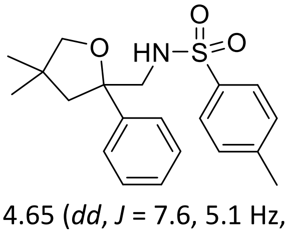

Following general procedure II, compound **9aA** is obtained as a white solid (42.3 mg, 80% yield) starting from triazole **1a** (44.04 mg, 0.15 mmol) and 3,3-dimethyloxetane **3A** with a 3 hours reaction time.
Purification: column chromatography (neutral Al_2O_3, pentane/Et_2O, 1:1)
M.p. = 99.5-100.5 °C; **Rf =** 0.59 (neutral Al_2O_3, pentane/Et_2O, 2:8); **^{1}H NMR (400 MHz, CDCl$_3$):** δ 7.62-7.54 (*m*, 2H), 7.31-7.27 (*m*, 4H), 7.24-7.18 (*m*, 3H), 4.65 (*dd*, *J* = 7.6, 5.1 Hz, 1H), 3.60 (*d*, *J* = 8.3 Hz, 1H), 3.55 (*d*, *J* = 8.3 Hz, 1H), 3.22 (*dd*, *J* = 12.4, 7.6 Hz, 1H), 2.96 (*dd*, *J* = 12.4, 5.0 Hz, 1H), 3.13 (*s*, 3H), 2.23 (*d*, *J* = 12.7 Hz, 1H), 2.06 (*d*, *J* = 12.7 Hz, 1H), 1.13 (*s*, 3H), 0.85 (*s*, 3H) ppm; **^{13}C NMR (100 MHz, CDCl$_3$):** δ 144.8 (C), 143.3 (C), 137.0 (C), 129.7 (CH), 128.5 (CH), 127.2 (CH), 127.1 (CH), 125.2 (CH), 86.4 (C), 80.3 (CH_2), 52.6 (CH_2), 50.0 (CH_2), 40.6 (C), 27.5 (CH_3), 27.3 (CH_2), 21.6 (CH_2) ppm; **IR (neat):** 3249, 2958, 2865, 1444, 1337, 1161, 1095, 1027, 817, 694, 537 cm^{-1}; **HR-MS (ESI):** m/z = 360.1631 [M+H]$^+$ (calculated for $C_{20}H_{26}NO_3S$ m/z = 360.1628).

Compound 9bA:

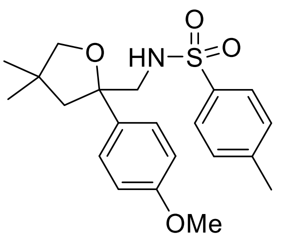

Following general procedure II, compound **9bA** is obtained as a yellowish solid (35.2 mg, 63% yield) starting from triazole **1b** (48.4 mg, 0.15 mmol) and 3,3-dimethyloxetane **3A** with a 3 hours reaction time.
Purification: column chromatography (neutral Al_2O_3, pentane/EtOAc, 7:3).
M.p. = 119-121 °C; **Rf =** 0.30 (silica gel, pentane/EtOAc, 8:2); **^{1}H NMR (400 MHz, CDCl$_3$):** δ 7.62-7.54 (*m*, 2H), 7.24-7.15 (*m*, 4H), 6.84-6.76 (*m*, 2H), 4.61 (*dd*, *J* = 7.5, 5.1 Hz, 1H), 3.79 (*s*, 3H), 3.58 (*d*, *J* = 8.3 Hz, 1H), 3.52 (*d*, *J* = 8.3 Hz, 1H), 3.19 (*dd*, *J* = 12.4, 7.5 Hz, 1H), 2.93 (*dd*, *J* = 12.4, 5.1 Hz, 1H), 2.39 (*s*, 3H), 2.19 (*d*, *J* = 12.6 Hz, 1H), 2.03 (*d*, *J* = 12.7 Hz, 1H), 1.13 (*s*, 3H), 0.86 (*s*, 3H) ppm; **^{13}C NMR (100 MHz, CDCl$_3$):** δ 158.7 (C), 143.3 (C), 137.0 (C), 136.7 (C), 129.7 (CH), 127.1 (CH), 126.4 (CH), 113.8 (CH), 86.1 (C), 80.2 (CH_2), 55.4 (CH_3), 52.7 (CH_2), 50.0 (CH_2), 40.6 (C), 27.6 (CH_3), 27.3 (CH_3), 21.6 (CH_3) ppm; **IR (neat):** 3252, 2928, 1513, 1410, 1317, 1251, 1160, 1062, 1033, 827, 809 cm^{-1}; **HR-MS (ESI):** m/z = 412.1554 [M+Na]$^+$ (calculated for $C_{21}H_{27}NNaO_4S$ m/z = 412.1553).

Compound 9cA:

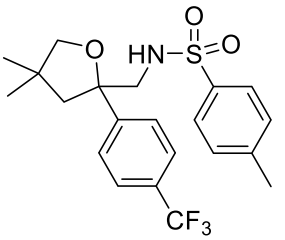

Following general procedure II, compound **9cA** is obtained as a white solid (42.3 mg, 68% yield) starting from triazole **1c** (53.5 mg, 0.15 mmol) and 3,3-dimethyloxetane **3A** with a 6 hours reaction time.
Purification: column chromatography (neutral Al_2O_3, pentane/EtOAc, 8:2).
M.p. = 130-131 °C; **Rf =** 0.51 (silica gel, pentane/EtOAc, 8:2); **^{1}H NMR (400 MHz, CDCl$_3$):** δ 7.53-7.46 (*m*, 4H), 7.37 (*d*, *J* = 8.2 Hz, 2H), 7.18 (*d*, *J* = 8.1 Hz, 2H), 4.67 (*t*, *J* = 6.4 Hz, 1H), 3.62 (*d*, *J* = 8.4 Hz, 1H), 3.56 (*d*, *J* = 8.4 Hz, 1H), 3.27 (*dd*, *J* = 12.6, 6.5 Hz, 1H), 3.01 (*dd*, *J* = 12.6, 6.3 Hz, 1H), 2.38 (*s*, 3H), 2.22 (*d*, *J* = 12.8 Hz, 1H), 2.01 (*d*, *J* = 12.8 Hz, 1H), 1.14 (*s*, 3H), 0.85 (*s*, 3H) ppm; **^{13}C NMR (100 MHz, CDCl$_3$):** δ 149.1 (C), 143.5 (C), 136.8 (C), 129.7 (CH), 129.4 (*q*, *J* = 32.3 Hz, C), 127.0 (CH), 125.7 (CH), 125.4 (*q*, *J* = 3.8 Hz, CH), 124.2 (*q*, *J* = 272.0 Hz, CF_3), 86.2 (C), 80.3 (CH_2), 52.5 (CH_2), 50.4 (CH_2), 40.7 (C), 27.2 (CH_3), 27.1 (CH_3), 21.6 (CH_3) ppm; **IR (neat):** 3317, 2961, 1330, 1314, 1155, 1115, 1068, 831, 808, 655 cm^{-1}; **HR-MS (ESI):** m/z = 450.1314 [M+Na]$^+$ (calculated for $C_{21}H_{24}F_3NNaO_3S$ m/z = 450.1321).

Compound 9dA:

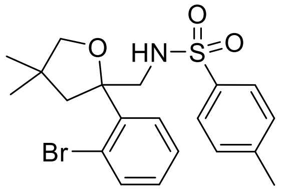

Following general procedure II, compound **9dA** is obtained as a white solid (38.2 mg, 58% yield) starting from triazole **1d** (56.7 mg, 0.15 mmol), 2 mol% of Rh_2(*S*-TCPTTL)$_4$ and 3,3-dimethyloxetane **3A** with a 6 hours reaction time. Purification: column chromatography (neutral Al_2O_3, pentane/EtOAc, 8:2) **M.p. =** 112-114 °C; **Rf =** 0.51 (silica gel, pentane/EtOAc, 8:2); **^{1}H NMR (400 MHz, CDCl$_3$):** δ 7.66 (*dd*, *J* = 7.9, 1.8 Hz, 1H), 7.50 (*d*, *J* = 8.3 Hz, 2H), 7.40 (*dd*, *J* = 7.9, 1.3 Hz, 1H), 7.29-7.23 (*m*, 1H), 7.15 (*d*, *J* = 8.0 Hz, 2H), 7.06 (*td*, *J* = 7.6, 1.8 Hz, 1H), 4.76-4.61 (*m*, 1H), 3.65-3.54 (*m*, 2H), 3.53-3.44 (*m*, 2H), 2.38 (*s*, 3H), 2.24 (*d*, *J* = 13.4 Hz, 1H), 2.17 (*d*, *J* = 13.5 Hz, 1H), 1.12 (*s*, 3H), 0.88 (*s*, 3H) ppm; **^{13}C NMR (100 MHz, CDCl$_3$):** δ 143.0 (C), 142.8 (C), 137.0 (C), 134.7 (CH), 129.6 (CH), 128.9 (CH), 128.3 (CH), 127.4 (CH), 127.1 (CH), 120.4 (C), 86.5 (C), 79.1 (CH_2), 50.5 (CH_2), 49.3 (CH_2), 40.9 (C), 27.2 (CH_3), 26.9 (CH_3), 21.6 (CH_3) ppm; **IR (neat):** 3289, 2853, 1322, 1163, 1034, 816, 758, 673 cm^{-1}; **HR-MS (ESI):** m/z = 438.0743 [M+H]$^+$ (calculated for $C_{20}H_{25}BrNO_3S$ m/z = 438.0733).

Compound 9eA:

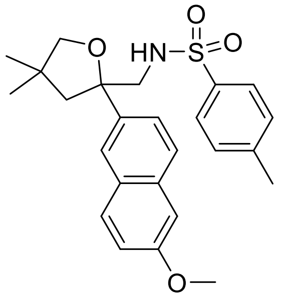

Following general procedure II, compound **9eA** is obtained as a white solid (47.5 mg, 74% yield) starting from triazole **1e** (55.2 mg, 0.15 mmol) and 3,3-dimethyloxetane **3A** with a 3 hours reaction time.
Purification: column chromatography (neutral Al_2O_3, pentane/EtOAc, 8:2).
M.p. = 137-139 °C; **Rf =** 0.52 (silica gel, pentane/EtOAc (7:3); **^{1}H NMR (400 MHz, CDCl$_3$):** δ 7.69-7.59 (*m*, 3H), 7.51-7.44 (*m*, 2H), 7.28 (*dd*, *J* = 8.6, 2.0 Hz, 1H), 7.15 (*dd*, *J* = 8.9, 2.5 Hz, 1H), 7.10 (*d*, *J* = 2.5 Hz, 1H), 7.05 (*d*, *J* = 8.0 Hz, 2H), 4.70 (*t*, *J* = 6.2 Hz, 1H), 3.92 (*s*, 3H), 3.65 (*d*, *J* = 8.4 Hz, 1H), 3.61 (*d*, *J* = 8.3 Hz, 1H), 3.32 (*dd*, *J* = 12.5, 7.1 Hz, 1H), 3.05 (*dd*, *J* = 12.5, 5.5 Hz, 1H), 2.31 (*s*, 3H), 2.25 (*d*, *J* = 12.7 Hz, 1H), 2.15 (d, *J* = 12.7 Hz, 1H), 1.16 (*s*, 3H), 0.85 (s, 3H) ppm; **^{13}C NMR (100 MHz, CDCl$_3$):** δ 157.9 (C), 143.1 (C), 139.8 (C), 136.9 (C), 133.7 (C), 129.7 (CH), 129.5 (CH), 128.7 (C), 127.2 (CH), 127.0 (CH), 124.0 (CH), 123.8 (CH), 119.2 (CH), 105.6 (CH), 86.4 (C), 80.2 (CH_2), 55.5 (CH_3), 52.7 (CH_2), 50.2 (CH_2), 40.7 (C), 27.5 (CH_3), 27.3 (CH_3), 21.6 (CH_3) ppm; **IR (neat):** 2926, 1605, 1312, 1293, 1213, 1152, 1062, 1033, 804, 655 cm^{-1}; **HR-MS (ESI):** m/z = 462.1715 [M+Na]$^+$ (calculated for $C_{25}H_{29}NNaO_4S$ m/z = 462.1710).

Compound 9fA:

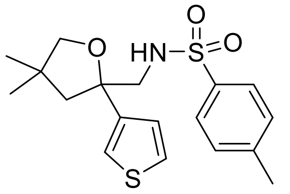

Following general procedure II, compound **9fA** is obtained as a white solid (40.8 mg, 75% yield) starting from triazole **1f** (45.2 mg, 0.15 mmol) and 3,3-dimethyloxetane **3A** with a 3 hours reaction time.
Purification: column chromatography (neutral Al_2O_3, pentane/EtOAc, 8:2).
M.p. = 92.5-94.5 °C; **Rf =** 0.31 (silica gel, pentane/EtOAc (8:2); **^{1}H NMR (400 MHz, CDCl$_3$):** δ 7.62 (*d*, *J* = 8.3 Hz, 2H), 7.25-7.20 (m, 3H), 7.11 (*dd*, *J* = 3.1, 1.3 Hz, 1H), 6.86 (*dd*, *J* = 5.0, 1.3 Hz, 1H), 4.65-4.60 (*m*, 1H), 3.56 (*s*, 2H), 3.19 (*dd*, *J* = 12.4, 7.4 Hz, 1H), 3.01 (*dd*, *J* = 12.4, 5.3 Hz, 1H), 2.40 (*s*, 3H), 2.15 (d, *J* = 12.7 Hz, 1H), 2.00 (*d*, *J* = 12.7 Hz, 1H), 1.12 (*s*, 3H), 0.91 (*s*, 3H) ppm; **^{13}C NMR (100 MHz, CDCl$_3$):** δ 146.4 (C), 143.4 (C), 137.0 (C), 129.8 (CH), 127.1 (CH), 126.5 (CH), 125.6 (CH), 120.7 (CH), 85.2 (C), 80.3 (CH_2), 52.0 (CH_2), 49.8 (CH_2), 40.6 (C), 27.5 (CH_3), 27.3 (CH_3), 21.7 (CH_3) ppm; **IR (neat):** 3237, 2922, 2861, 1448, 1329, 1160, 1099, 1026, 791, 665 cm^{-1}; **HR-MS (ESI):** m/z = 366.1194 [M+H]$^+$ (calculated for $C_{18}H_{24}NO_3S_2$ m/z = 366.1192).

Compound 9gA:

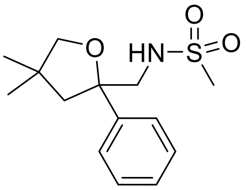

Following general procedure II, compound **9gA** is obtained as a white solid (29.4 mg, 71% yield) starting from triazole **1g** (32.7 mg, 0.15 mmol) and 3,3-dimethyloxetane **3A** with a 3 hours reaction time.

Purification: column chromatography (silica gel, pentane/EtOAc, 8:2).

M.p. = 92.5-94.5 °C; **Rf =** 0.51 (silica gel, pentane /EtOAc, 6:4); ^{1}**H NMR (400 MHz, CDCl$_3$):** δ 7.41-7.32 (*m*, 4H), 7.28-7.23 (*m*, 1H), 4.44 (*t*, *J* = 6.3 Hz, 1H), 3.67 (*d*, *J* = 8.4 Hz, 1H), 3.61 (*d*, *J* = 8.4 Hz, 1H), 3.48 (*dd*, *J* = 13.4, 6.5 Hz, 1H), 3.32 (*dd*, *J* = 13.4, 5.9 Hz, 1H), 2.61 (*s*, 3H), 2.11 (*s*, 2H), 1.16 (*s*, 3H), 0.90 (*s*, 3H) ppm; ^{13}**C NMR (100 MHz, CDCl$_3$):** δ 144.9 (C), 128.7 (CH), 127.4 (CH), 125.5 (CH), 86.5 (C), 79.9 (CH_2), 53.3 (CH_2), 50.9 (CH_2), 40.8 (CH_3), 40.7 (C), 27.3 (CH_3), 27.1 (CH_3) ppm; **IR (neat):** 3277, 2961, 1425, 1312, 1150, 1050, 1027, 968, 768, 699 cm^{-1}, **HR-MS (ESI):** m/z = 306.1126 [M+Na]$^+$ (calculated for $C_{14}H_{21}NNaO_3S$ m/z = 306.1134).

Compound 9hA:

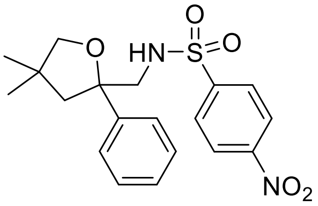

Following general procedure II, compound **9hA** is obtained as a yellowish solid (33.0 mg, 58% yield) starting from triazole **9h** (48.3 mg, 0.15 mmol) and 3,3-dimethyloxetane **3A** with a 3 hours reaction time. The treatment with $LiAlH_4$ is at 0 °C during 30 minutes.

Purification: column chromatography (neutral Al_2O_3, pentane/EtOAc, 9:1).

M.p. = 126-127 °C; **Rf =** 0.44 (silica gel, pentane/EtOAc, 8:2); ^{1}**H NMR (400 MHz, CDCl$_3$):** δ 8.19 (*d*, *J* = 8.8 Hz, 2H), 7.76 (*d*, *J* = 8.8 Hz, 2H), 7.25-7.16 (*m*, 5H), 4.87 (*t*, *J* = 6.0 Hz, 1H), 3.55 (*q*, *J* = 8.4 Hz, 2H), 3.40 (*dd*, *J* = 12.7, 6.0 Hz, 1H), 3.13 (*dd*, *J* = 12.7, 5.9 Hz, 1H), 2.15-1.98 (*m*, 2H), 1.13 (*s*, 1H), 0.86 (*s*, 1H) ppm; ^{13}**C NMR (100 MHz, CDCl$_3$):** δ 149.9 (C), 146.1 (C), 144.4 (C), 128.6 (CH), 128.2 (CH), 127.3 (CH), 125.2 (CH), 124.2 (CH), 86.0 (C), 79.9 (CH_2), 52.9 (CH_2), 50.9 (CH_2), 40.7 (C), 27.3 (CH_3), 27.1 (CH_3) ppm; **IR (neat):** 3275, 2962, 2925, 1529, 1343, 1166, 1091, 1055, 1027, 851, 763, 736, 684 cm^{-1}; **HR-MS (ESI):** m/z = 391.1321 [M+H]$^+$ (calculated for $C_{19}H_{23}N_2O_5S$ m/z = 391.1322).

Compound 9aB:

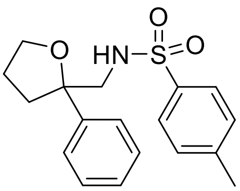

Following general procedure II, compound **9aB** is obtained as a white solid (29.8 mg, 60% yield) starting from triazole **1a** (44.9 mg, 0.15 mmol) and unsubstituted oxetane **3B** with a 3 hours reaction time.

Purification: column chromatography (neutral Al_2O_3, pentane/Et_2O, 1:1).

M.p. = 72.5-74.5 °C; **Rf =** 0.44 (neutral Al_2O_3, pentane/Et_2O, 2:8); ^{1}**H NMR (400 MHz, CDCl$_3$):** δ 7.66-7.58 (*m*, 2H), 7.32-4.27 (*m*, 4H), 7.25-7.21 (*m*, 3H), 4.69 (*dd*, *J* = 8.8, 4.2 Hz, 1H), 4.01-3.92 (*m*, 1H), 3.90-3.82 (*m*, 1H), 3.29 (*dd*, *J* = 12.3 Hz, 8.5 Hz, 1H), 2.98 (*dd*, *J* = 12.3, 4.1 Hz, 1H), 2.49-2.34 (*m*, 4H), 2.17-2.07 (*m*, 1H), 2.04-1.92 (*m*, 1H), 1.87-1.74 (*m*, 1H) ppm; ^{13}**C NMR (100 MHz, CDCl$_3$):** δ 144.3 (C), 143.4 (C), 136.9 (C), 129.8 (CH), 128.5 (CH), 127.4 (CH), 127.1 (CH), 125.2 (CH), 85.8 (C), 68.6 (CH_2), 51.7 (CH_2), 35.2 (CH_2), 26.1 (CH_2), 21.6 (CH_3) ppm; **IR (neat):** 3256, 2920, 1429, 1323, 1158, 1089, 1064, 816, 697 cm^{-1}; **HR-MS (ESI):** m/z = 332.1309 [M+H]$^+$ (calculated for $C_{18}H_{22}NO_3S$ m/z = 332.1315).

Compound 9aC:

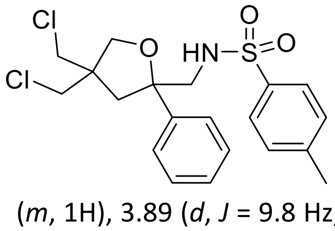

Following general procedure II, compound **9aC** is obtained as a white solid (48.1 mg, 75% yield) starting from triazole **1a** (44.8 mg, 0.15 mmol) and 3,3-bis(chloromethyl)oxetane **3C** with a 3 hours reaction time.
Purification: column chromatography (neutral Al_2O_3, pentane/Et_2O, 1:1).
M.p. = 97.5-99.5 °C; **Rf =** 0.5 (neutral Al_2O_3, pentane/Et_2O, 2:8); **^{1}H NMR (400 MHz, $CDCl_3$):** δ 7.63 (*d*, *J* = 8.3 Hz, 2H), 7.39-7.23 (*m*, 7H), 4.79-4.70 (*m*, 1H), 3.89 (*d*, *J* = 9.8 Hz, 1H), 3.83-3.66 (*m*, 3H), 3.40 (*d*, *J* = 11.1 Hz, 1H), 3.35 (*d*, *J* = 11.2 Hz, 1H), 3.21 (*dd*, *J* = 13.0, 8.8 Hz, 1H), 2.95 (*dd*, *J* = 13.0, 4.5 Hz, 1H), 2.46 (*d*, *J* = 13.8 Hz, 1H), 2.40 (*s*, 3H), 2.35 (*d*, *J* = 13.8 Hz, 1H) ppm; **^{13}C NMR (100 MHz, $CDCl_3$):** δ 143.7 (C), 142.7 (C), 136.9 (C), 129.9 (CH), 129.0 (CH), 128.0 (CH), 127.1 (CH), 125.1 (CH), 86.8 (C), 73.5 (CH_2), 52.0 (CH_2), 51.8 (C), 48.2 (CH_2), 47.5 (CH_2), 42.7 (CH_2), 21.7 (CH_3) ppm; **IR (neat):** 3285, 1440, 1408, 1326, 1163, 1042, 815, 707, 661 cm^{-1}; **HR-MS (ESI):** m/z = 428.0863 $[M+H]^+$ (calculated for $C_{20}H_{24}Cl_2NO_3S$ m/z = 428.0849).

Compound 9aD:

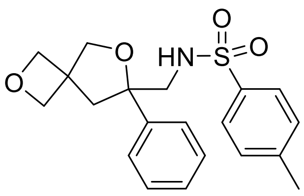

Following general procedure II, compound **9aD** is obtained as a white solid (24.6 mg, 44% yield) starting from triazole **1a** (44.9 mg, 0.15 mmol) and 2,6-dioxaspiro[3.3]heptane **3D** with a 3 hours reaction time.
Purification: column chromatography (neutral Al_2O_3, pentane/EtOAc, 6:4).
M.p. = 192.5-194.5 °C; **Rf =** 0.25 (silica gel, pentane/EtOAc, 1:1); **^{1}H NMR (400 MHz, $CDCl_3$):** δ 7.67 (*d*, *J* = 8.0 Hz, 2H), 7.40-7.27 (*m*, 7H), 4.13 (*d*, *J* = 7.7 Hz, 1H), 3.88 (*d*, *J* = 10.8 Hz, 1H), 3.82-3.73 (*m*, 2H), 3.70 (*s*, 2H), 2.71 (*d*, *J* = 10.9 Hz, 1H), 2.66 (*d*, *J* = 11.4 Hz, 1H), 2.43 (*s*, 3H), 1.98 (*d*, *J* = 11.2 Hz, 1H), 1.85 (*d*, *J* = 11.2 Hz, 1H) ppm; **^{13}C NMR (100 MHz, $CDCl_3$):** δ 143.7 (C), 141.6 (C), 134.3 (C), 129.8 (CH), 128.6 (CH), 128.0 (CH), 127.6 (CH), 125.4 (CH), 82.8 (C), 74.3 (CH_2), 65.6 (CH_2), 56.6 (CH_2), 52.1 (CH_2), 46.7 (C), 43.0 (CH_2), 21.7 (CH_3) ppm; **IR (neat):** 2874, 1333, 1152, 1001, 812, 699, 666, 561 cm^{-1}; **HR-MS (ESI):** m/z = 374.1423 $[M+H]^+$ (calculated for $C_{20}H_{23}NO_4S$ m/z = 374.1421).

Compound 9iA:

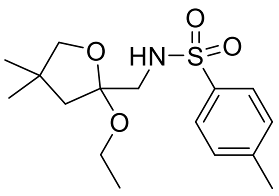

Following general procedure II but without adding Rh_2(*S*-TCPTTL)$_4$, compound **9iA** is obtained as a colorless oil (24.6 mg, 50% yield) starting from triazole **1i** (40.1 mg, 0.15 mmol) and 3,3-dimethyloxetane **3A**, heating at 60 °C during 6 hours.
Purification: column chromatography (neutral Al_2O_3, pentane/Et_2O, 7:3).
Rf = 0.41 (silica gel, pentane/Et_2O, 1:1); **^{1}H NMR (400 MHz, $CDCl_3$):** δ 7.74 (*d*, *J* = 8.3 Hz, 2H), 7.31 (*d*, *J* = 8.0 Hz, 2H), 4.54 (*t*, *J* = 6.0 Hz, 1H), 3.61 (*d*, *J* = 8.2 Hz, 1H), 3.52 (*d*, J = 8.2 Hz, 1H), 3.49-3.38 (*m*, 1H), 3.23-3.08 (*m*, 2H), 3.00 (*dd*, J = 12.1, 6.8 Hz, 1H), 2.43 (*s*, 3H), 1.84 (*d*, *J* = 13.4 Hz, 1H), 1.77 (*d*, *J* = 13.4 Hz, 1H), 1.13 (*s*, 3H), 1.08 (*t*, *J* = 7.0 Hz, 3H), 1.04 (*s*, 3H) ppm; **^{13}C NMR (100 MHz, $CDCl_3$):** δ 143.7 (C), 136.7 (C), 129.9 (CH), 127.3 (CH), 108.3 (C), 80.7 (CH_2), 56.5 (CH_2), 49.4 (CH_2), 46.7 (CH_2), 39.2 (C), 28.4 (CH_3), 26.8 (CH_3), 21.7 (CH_3), 15.8 (CH_3) ppm; **IR (neat):** 1599, 1443, 1325, 1157, 1081, 1040, 813, 660 cm^{-1}; **HR-MS (ESI):** m/z = 350.1391 $[M+Na]^+$ (calculated for $C_{16}H_{25}NNaO_4S$ m/z = 350.1397).

7. General procedure III: synthesis of macrocycles 5 and 6

Method A:

In a 2 mL screw-cap vial equipped with a magnetic stirring bar, Rh_2(*S*-TCPTTL)$_4$ (1 or 2 mol %) and the corresponding triazole (0.2 mmol, 1 equiv) were dissolved in 0.20 mL (1 M) of the corresponding oxetane. The vial was flushed with nitrogen and capped. After several hours stirring at 100 °C, the solution was concentrated under reduced pressure and the residue was purified by column chromatography.

Method B:

In a 2 mL screw-cap vial equipped with a magnetic stirring bar, 0.1 mmol (1 equiv) of the corresponding 4-phthalimido *N*-sulfonyltriazole was dissolved in 0.10 mL (1 M) of 3,3-dimethyloxetane. The vial was flushed with nitrogen and capped. After stirring at 60 °C during 2 h, the solution was concentrated under reduced pressure and the residue was purified by column chromatography.

Analysis data for macrocycles 5

Compound 5aA:

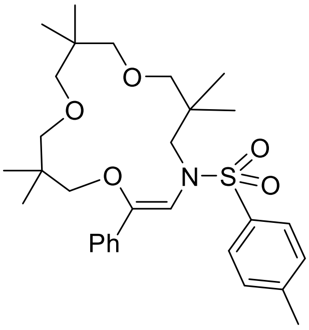

Following general procedure III, Method A: **5aA** is obtained as a white solid (34.2 mg, 34% yield) starting from triazole **1a** (57.2 mg, 0.19 mmol), 3,3-dimethyloxetane **3A** and using 1 mol% of Rh_2(*S*-TCPTTL)$_4$ with a 5 hour reaction time.

Purification: column chromatography (silica gel, pentane/Et_2O, 8:2).

M.p. = 135-137 °C; **Rf =** 0.47 (Silica gel, pentane/Et_2O, 8:2); **^{1}H NMR (400 MHz, acetone-*d*$_6$):** δ 7.77-7.68 (*m*, 2H), 7.48-7.38 (*m*, 5H), 7.34-7.28 (*m*, 2H), 5.66 (*s*, 1H), 3.50 (*s*, 2H), 3.24 (*s*, 2H), 3.19 (*s*, 2H), 3.16 (*s*, 2H), 3.12 (*s*, 2H), 2.82 (*s*, 2H), 2.44 (*s*, 3H), 1.08 (*s*, 6H), 0.83 (*s*, 6H), 0.69 (*s*, 6H) ppm; **^{13}C NMR (100 MHz, acetone-*d*$_6$):** 153.9 (C), 143.9 (C), 138.2 (C), 135.1 (C), 130.3 (CH), 129.8 (CH), 129.4 (CH), 128.4 (CH), 128.2 (CH), 113.4 (CH), 83.2 (CH_2), 78.0 (CH_2), 76.6 (CH_2), 75.3 (CH_2), 73.2 (CH_2), 58.1 (CH_2), 38.1 (C), 36.7 (C), 36.4 (C), 24.3 (CH_3), 22.9 (CH_3), 22.2 (CH_3), 21.4 (CH_3) ppm; **IR (neat):** 2962, 2856, 1345, 1331, 1119, 1102, 1075, 763, 672 cm^{-1}; **HR-MS (ESI):** m/z = 530.2957 $[M+H]^+$ (calculated for $C_{30}H_{44}NO_5S$ m/z = 530.2935).

Compound 5bA:

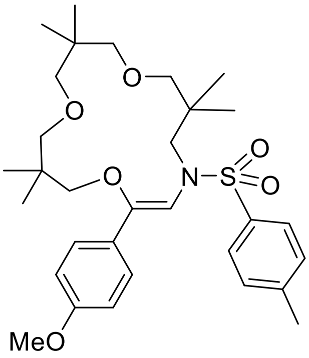

Following general procedure III, Method A: **5bA** is obtained as a white solid (21.3 mg, 19% yield) starting from triazole **1b** (65.8 mg, 0.2 mmol), 3,3-dimethyloxetane **3A** and using 1 mol% of Rh_2(*S*-TCPTTL)$_4$ with a 3 hour reaction time.

Purification: column chromatography (silica gel, pentane/Et_2O, 8:2).

M.p. = 93-95 °C; **Rf =** 0.38 (silica gel, pentane/Et_2O, 8:2); **^{1}H NMR (400 MHz, acetone-*d*$_6$):** δ 7.72 (*d*, *J* = 8.2 Hz, 2H), 7.41 (*d*, *J* = 8.0 Hz, 2H), 7.23 (*d*, *J* = 8.7 Hz, 2H), 6.97 (*d*, *J* = 8.7 Hz, 2H), 5.56 (*s*, 1H), 3.83 (*s*, 3H), 3.47 (*s*, 2H), 3.23 (*s*, 2H), 3.18 (*s*, 2H), 3.15 (*s*, 2H), 3.11 (*s*, 2H), 2.81 (*s*, 2H); 2.43 (*s*, 3H), 1.07 (*s*, 6H), 0.83 (*s*, 3H), 0.69 (*s*, 3H) ppm; **^{13}C NMR (100 MHz, acetone-*d*$_6$):** δ 161.3 (C), 153.8 (C), 143.8 (C), 138.2 (C), 130.2 (CH), 129.6 (CH), 128.4 (CH), 127.3 (C), 114.8 (CH), 112.1 (CH), 83.2 (CH_2), 77.9 (CH_2), 76.5 (CH_2), 75.3 (CH_2), 73.0 (CH_2), 58.0 (CH_2), 55.7 (CH_3), 38.0 (C), 36.7 (C), 36.4 (C), 24.3 (CH_3), 22.9 (CH_3), 22.2 (CH_3), 21.4 (CH_3) ppm; **IR (neat):** 2959, 2867, 1605, 1511, 1333, 1250, 1157, 1111, 1074, 1026, 1000, 846, 738, 658, 547 cm^{-1}; **HR-MS (ESI):** m/z = 582.2860 $[M+Na]^+$ (calculated for $C_{31}H_{45}NNaO_6S$ m/z = 582.2860).

Compound 5cA:

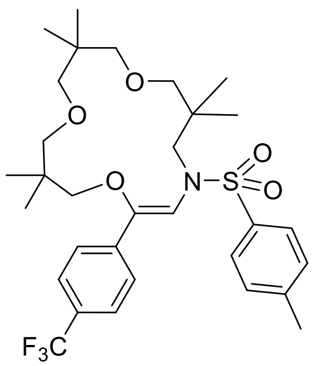

Following general procedure III, Method A: **5cA** is obtained as white solid (45.7 mg, 38% yield) starting from triazole **1c** (73.9 mg, 0.2 mmol), 3,3-dimethyloxetane **3A** and using 1 mol% of Rh_2(*S*-TCPTTL)$_4$ with a 28 hour reaction time.

Purification: column chromatography (silica gel, pentane/Et_2O, 9:1).

M.p. = 148-150 °C; **Rf =** 0.51 (silica gel, pentane/Et_2O, 8:2); ^{1}**H NMR (400 MHz, acetone-*d*$_6$):** δ 7.77 (*d*, *J* = 7.9 Hz, 2H), 7.72 (*d*, *J* = 8.3 Hz, 2H), 7.56 (*d*, *J* = 8.0 Hz, 2H), 7.44 (*d*, *J* = 8.0 Hz, 2H), 5.86 (*s*, 1H), 3.53 (*s*, 2H), 3.24 (*s*, 2H), 3.22 (*s*, 2H), 3.16 (*s*, 2H), 3.12 (*s*, 2H), 2.84 (*s*, 2H), 2.44 (*s*, 3H), 1.08 (*s*, 3H), 0.83 (*s*, 3H), 0.72 (*s*, 3H) ppm; ^{13}**C NMR (100 MHz, acetone-*d*$_6$):** δ 152.3 (C), 144.1 (C), 139.3 (C), 138.0 (C), 131.0 (*q*, J = 32.3 Hz, C), 130.4 (CH), 128.7 (CH), 128.3 (CH), 125.2 (*q*, J = 271.6 Hz, CF_3), 126.4 (*q*, *J* = 3.8 Hz, CH), 115.4 (CH), 83.1 (CH_2), 78.0 (CH_2), 76.7 (CH_2), 75.2 (CH_2), 73.7 (CH_2), 58.2 (CH_2), 38.1 (C), 36.7 (C), 36.5 (C), 24.2 (CH_3), 22.9 (CH_3), 22.2 (CH_3), 21.4 (CH_3) ppm; **IR (neat):** 2963, 2874, 1324, 1162, 1121, 1106, 1063, 1003, 851, 547 cm^{-1}; **HR-MS (ESI):** m/z = 598.2805 $[M+H]^+$ (calculated for $C_{31}H_{43}F_3NO_5S$ m/z = 598.2809).

Compound 5gA:

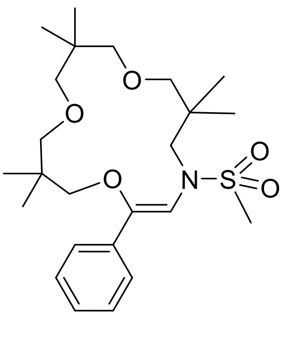

Following general procedure III, Method A: **5gA** is obtained as a white solid (8.7 mg, 10% yield) starting from triazole **1g** (44.8 mg, 0.2 mmol), 3,3-dimethyloxetane **3A** and using 1 mol% of Rh_2(*S*-TCPTTL)$_4$ with a 3 hour reaction time.

Purification: column chromatography (silica gel, pentane/Et_2O, 8:2).

M.p. = 124-125 °C; **Rf =** 0.61 (silica gel, pentane/Et_2O, 7:3); ^{1}**H NMR (400 MHz, acetone-*d*$_6$):** δ 7.44 (*s*, 5H), 5.61 (*s*, 1H), 3.56 (*s*, 2H), 3.53 (*s*, 2H), 3.33 (*s*, 2H), 3.28 (*s*, 2H), 3.23 (*s*, 2H), 3.21 (*s*, 2H), 2.93 (*s*, 3H), 1.04 (*s*, 6H), 0.97 (*s*, 6H), 0.85 (*s*, 6H) ppm; ^{13}**C NMR (100 MHz, acetone-*d*$_6$):** δ 154.4 (C), 134.8 (C), 129.9 (CH), 129.4 (CH), 128.5 (CH), 113.5 (CH), 82.6 (CH_2), 77.9 (CH_2), 76.7 (CH_2), 75.9 (CH_2), 73.8 (CH_2), 58.2 (CH_2), 38.1 (C), 37.8 (CH_3), 36.83 (C), 36.78 (C), 24.2 (CH_3), 22.8 (CH_3), 22.5 (CH_3) ppm; **IR (neat):** 2961, 2871, 1483, 1334, 1151, 1107, 1067, 1002, 966, 789, 773, 707 cm^{-1}; **HR-MS (ESI):** m/z = 454.2622 $[M+H]^+$ (calculated for $C_{24}H_{40}NO_5S$ m/z = 454.2622).

Compound 5hA:

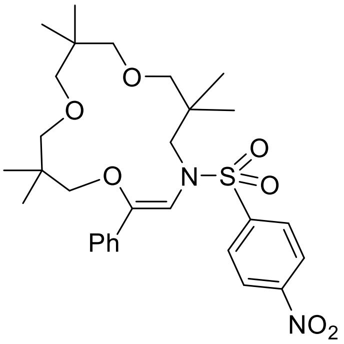

Following general procedure III, Method A: **5hA** is obtained as a yellowish solid (69.2 mg, 64% yield) starting from triazole **1h** (63.9 mg, 0.19 mmol), 3,3-dimethyloxetane **3A** and using 2 mol% of Rh_2(*S*-TCPTTL)$_4$ with a 3 hour reaction time.

Purification: column chromatography (silica gel, pentane/Et_2O, 8:2).

M.p. = 120.5-122.5 °C; **Rf =** 0.56 (silica gel, pentane/Et_2O, 8:2); ^{1}**H NMR (400 MHz, $CDCl_3$):** δ 8.37-8.30 (*m*, 2H), 8.07-8.00 (*m*, 2H), 7.43-7.34 (*m*, 3H), 7.21-7.13 (*m*, 2H), 5.72 (*s*, 1H), 3.52 (*s*, 2H), 3.20 (*s*, 2H), 3.15 (*s*, 2H), 3.11 (*s*, 2H), 3.06 (*s*, 2H), 2.72 (*s*, 2H), 1.11 (*s*, 6H), 0.82 (*s*, 6H), 0.58 (*s*, 6H) ppm; ^{13}**C NMR (100 MHz, $CDCl_3$):** δ 154.2 (C), 150.0 (C), 145.8 (C), 133.6 (CH), 129.5 (CH), 128.8 (CH), 128.7 (CH), 127.4 (CH), 123.8 (CH), 111.5 (CH), 82.6 (CH_2), 77.7 (CH_2), 76.3 (CH_2), 74.6 (CH_2), 72.8 (CH_2), 57.8 (CH_2), 37.6 (C), 36.2 (CH), 35.8 (CH), 24.0 (CH_3), 22.7 (CH_3), 21.9 (CH_3) ppm; **IR (neat):** 2964, 2875, 1528, 1349, 1332, 1165, 1107, 1074, 1004, 767, 741 cm^{-1}; **HR-MS (ESI):** m/z = 583.2445 $[M+Na]^+$ (calculated for $C_{29}H_{40}N_2NaO_7S$ m/z = 583.2449).

Compound 5jA:

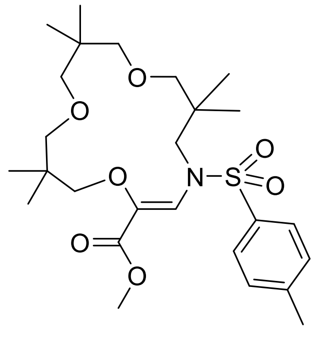

Following general procedure III, Method A: **5jA** is obtained as a white solid (38.4 mg, 39% yield) starting from triazole **1j** (54.1 mg, 0.19 mmol), 3,3-dimethyloxetane **3A** and using 1 mol% of Rh_2(*S*-TCPTTL)$_4$ with a 7 hour reaction time.

Purification: column chromatography (silica gel, pentane/Et_2O, 8:2).

M.p. = 97-99 °C; **Rf =** 0.52 (silica gel, pentane/Et_2O, 7:3); **^{1}H NMR (400 MHz, acetone-*d$_6$*):** δ 7.69 (*d*, *J* = 8.3 Hz, 2H), 7.42 (*d*, *J* = 8.0 Hz, 2H), 6.89 (*s*, 1H), 3.74 (*s*, 3H), 3.71 (*s*, 2H), 3.30 (*s*, 2H), 3.20 (*s*, 2H), 3.17 (*s*, 2H), 3.09 (*s*, 2H), 2.95 (*s*, 2H), 2.42 (*s*, 3H), 0.98 (*s*, 6H), 0.83 (*s*, 6H), 0.73 (*s*, 6H) ppm; **^{13}C NMR (100 MHz, acetone-*d$_6$*):** δ 164.4 (C), 144.7 (C), 139.7 (C), 137.7 (C), 130.6 (CH), 127.8 (CH), 124.0 (CH), 82.3 (CH_2), 78.1 (CH_2), 76.9 (CH_2), 76.2 (CH_2), 75.8 (CH_2), 55.9 (CH_2), 52.3 (CH_3), 38.8 (C), 36.6 (C), 36.5 (C), 23.9 (CH_3), 22.9 (CH_3), 22.3 (CH_3), 21.4 (CH_3) ppm; **IR (neat):** 2961, 2879, 1723, 1353, 1267, 1164, 1111, 1020, 724, 655 cm^{-1}; **HR-MS (ESI):** m/z = 512.2671 [M+H]$^+$ (calculated for $C_{26}H_{42}NO_7S$ m/z = 512.2677).

Compound 5kA:

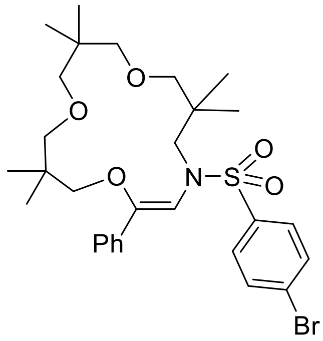

Following general procedure III, Method A: **5kA** is obtained as a white solid (55.1 mg, 46% yield) starting from triazole **1k** (72.7 mg, 0.20 mmol), 3,3-dimethyloxetane **3A** and using 1 mol% of Rh_2(*S*-TCPTTL)$_4$ with a 3 hour reaction time.

Purification: column chromatography (silica gel, pentane/Et_2O, 9:1).

M.p. = 117.5-119.5 °C; **Rf =** 0.40 (silica gel, pentane/Et_2O, 9:1); **^{1}H NMR (400 MHz, acetone-*d$_6$*):** δ 7.88-7.82 (*m*, 2H), 7.81-7.75 (*m*, 2H), 7.47-7.40 (*m*, 3H), 7.35-7.29 (*m*, 2H), 5.68 (*s*, 1H), 3.51 (*s*, 2H), 3.24 (*s*, 2H), 3.20 (*s*, 2H), 3.15 (*s*, 2H), 3.12 (*s*, 2H), 2.82 (*s*, 2H), 1.09 (*s*, 6H), 0.83 (*s*, 6H), 0.70 (*s*, 6H) ppm; **^{13}C NMR (100 MHz, acetone-*d$_6$*):** 154.5 (C), 140.1 (C), 134.7 (C), 133.0 (CH), 130.2 (CH), 130.0 (CH), 129.5 (CH), 128.2 (CH), 127.7 (CH), 112.9 (CH), 83.1 (CH_2), 78.0 (CH_2), 76.6 (CH_2), 75.2 (CH_2), 73.2 (CH_2), 58.2 (CH_2), 38.0 (C), 36.7 (C), 36.3 (CH), 24.2 (CH_3), 22.9 (CH_3), 22.2 (CH_3) ppm; **IR (neat):** 2963, 2871, 1469, 1346, 1330, 1163, 1101, 1074, 1004, 766, 596, 552 cm^{-1}; **HR-MS (ESI):** m/z = 594.1894 [M+H]$^+$ (calculated for $C_{29}H_{41}BrNO_5S$ m/z = 594.1883).

Compound 5lA:

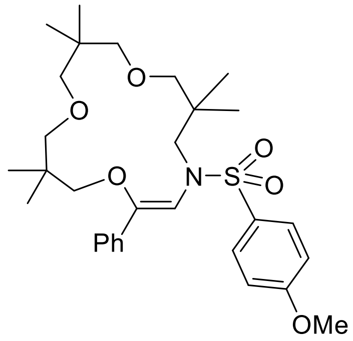

Following general procedure III, Method A: **5lA** is obtained as a white solid (40.2 mg, 37% yield) starting from triazole **1l** (62.3 mg, 0.20 mmol), 3,3-dimethyloxetane **3A** and using 1 mol% of Rh_2(*S*-TCPTTL)$_4$ with a 10 hour reaction time.

Purification: column chromatography (silica gel, pentane/Et_2O, 7:3).

M.p. = 96-98 °C; **Rf =** 0.44 (silica gel, pentane/Et_2O, 7:3); **^{1}H NMR (400 MHz, acetone-*d$_6$*):** δ 7.80-7.74 (*m*, 2H), 7.46-7.39 (*m*, 3H), 7.35-7.30 (*m*, 2H), 7.17-7.11 (*m*, 2H), 5.65 (*s*, 1H), 3.91 (*s*, 3H), 3.49 (*s*, 2H), 3.24 (*s*, 2H), 3.22 (*s*, 2H), 3.16 (*s*, 2H), 3.13 (*s*, 2H), 2.86 (*s*, 2H), 1.08 (*s*, 3H), 0.83 (*s*, 3H), 0.71 (*s*, 3H) ppm; **^{13}C NMR (100 MHz, acetone-*d$_6$*):** 163.9 (C), 153.9 (C), 135.2 (C), 132.9 (C), 130.4 (CH), 129.8 (CH), 129.4 (CH), 128.2 (CH), 114.9 (CH), 113.5 (CH), 83.2 (CH_2), 78.0 (CH_2), 76.6 (CH_2), 75.3 (CH_2), 73.3 (CH_2), 58.1 (CH_2), 56.1 (CH_3), 38.1 (C), 36.7 (C), 36.5 (C), 24.3 (CH_3), 22.9 (CH_3), 22.3 (CH_3) ppm; **IR (neat):** 2960, 2872, 1340, 1258, 1157, 1114, 766 cm^{-1}; **HR-MS (ESI):** m/z = 546.2888 [M+H]$^+$ (calculated for $C_{30}H_{44}NO_6S$ m/z = 546.2884).

Compound 5mA:

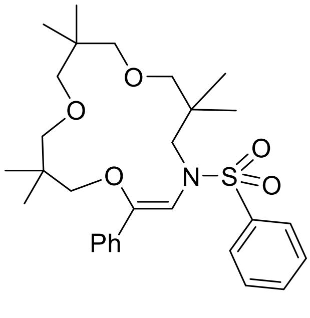

Following general procedure III, Method A: **5mA** is obtained as a white solid (35.3 mg, 34% yield) starting from triazole **1m** (57.0 mg, 0.20 mmol), 3,3-dimethyloxetane **3A** and using 1 mol% of Rh_2(*S*-TCPTTL)$_4$ with a 4 hour reaction time.

Purification: column chromatography (silica gel, pentane/Et_2O, 8:2).

M.p. = 145-147 °C; **Rf =** 0.51 (silica gel, pentane/Et_2O, 8:2); **^{1}H NMR (400 MHz, acetone-*d*$_6$):** δ 7.89-7.81 (*m*, 2H), 7.71-7.58 (*m*, 3H), 7.45-7.39 (*m*, 3H), 7.33-7.30 (*m*, 2H), 5.68 (*s*, 1H), 3.54 (*s*, 2H), 3.25 (*s*, 2H), 3.21 (*s*, 2H), 3.16 (*s*, 2H), 3.12 (*s*, 2H), 2.84 (*s*, 2H), 1.09 (*s*, 6H), 0.83 (*s*, 6H), 0.68 (*s*, 6H) ppm; **^{13}C NMR (100 MHz, acetone-*d*$_6$):** 154.0 (C), 141.1 (C), 135.1 (C), 133.2 (CH), 129.9 (CH), 129.7 (CH), 129.4 (CH), 128.3 (CH), 128.2 (CH), 113.1 (CH), 83.1 (CH_2), 78.0 (CH_2), 76.6 (CH_2), 75.4 (CH_2), 73.4 (CH_2), 58.1 (CH_2), 38.1 (C), 36.7 (C), 36.5 (C), 24.3 (CH_3), 22.9 (CH_3), 22.4 (CH_3) ppm; **IR (neat):** 2970, 2865, 1333, 1163, 1104, 1074, 998, 746 cm^{-1}; **HR-MS (ESI):** m/z = 516.2779 [M+H]$^+$ (calculated for $C_{29}H_{42}NO_5S$ m/z = 516.2778).

Compound 5nA:

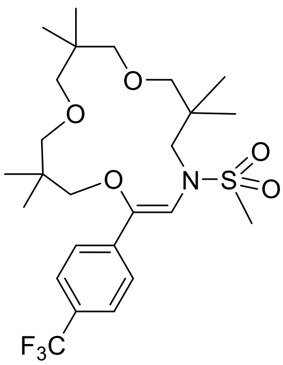

Following general procedure III, Method A: **5nA** is obtained as white solid (20.9 mg, 20% yield) starting from triazole **1n** (58.2 mg, 0.2 mmol), 3,3-dimethyloxetane **3A** and using 2 mol% of Rh_2(*S*-TCPTTL)$_4$ with a 7 hour reaction time.

Purification: column chromatography (silica gel, pentane/EtOAc, 9:1).

M.p. = 139.5-141.5 °C; **Rf =** 0.65 (silica gel, pentane/EtOAc, 9:1); **^{1}H NMR (400 MHz, acetone-*d*$_6$):** δ 7.79 (*d*, *J* = 8.1 Hz, 2H), 7.68 (*d*, *J* = 8.1 Hz, 2H), 5.80 (*s*, 1H), 3.60 (*s*, 2H), 3.58 (s, 2H), 3.33 (*s*, 2H), 3.28 (*s*, 2H), 3.23 (*s*, 2H), 3.21 (*s*, 2H), 2.95 (*s*, 3H), 1.04 (*s*, 6H), 1.00 (*s*, 6H), 0.85 (*s*, 6H) ppm; **^{13}C NMR (100 MHz, acetone-*d*$_6$):** δ 152.7 (C), 139.0 (C), 131.1 (*q*, *J* = 32.3 Hz, C), 129.2 (CH), 126.4 (*q*, *J* = 3.9 Hz, CH), 125.2 (*q*, *J* = 271.3 Hz, CF_3), 115.4 (CH), 82.6 (CH_2), 77.9 (CH_2), 76.8 (CH_2), 75.8 (CH_2), 74.2 (CH_2), 58.3 (CH_2), 38.1 (C), 37.9 (CH_3), 36.9 (C), 36.8 (C), 24.2 (CH_3), 22.8 (CH_3), 22.5 (CH_3) ppm; **IR (neat):** 2961, 2875, 1327, 1152, 1111, 1082, 1065, 1006, 793 cm^{-1}; **HR-MS (ESI):** m/z = 522.2503 [M+H]$^+$ (calculated for $C_{25}H_{39}F_3NO_5S$ m/z = 522.2496).

Compound 5aB:

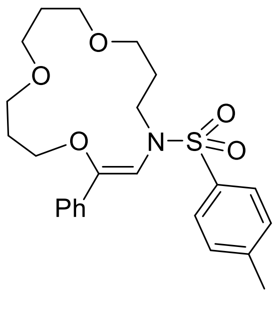

Following general procedure III, Method A: **5aB** is obtained as a colourless oil (31.0 mg, 35% yield) starting from triazole **1a** (59.7 mg, 0.20 mmol), oxetane **3B** and using 1 mol% of Rh_2(*S*-TCPTTL)$_4$ with a 17 hour reaction time.

Purification: column chromatography (neutral Al_2O_3, pentane/EtOAc, 8:2).

Rf = 0.56 (neutral Al_2O_3, pentane/EtOAc, 8:2); **^{1}H NMR (400 MHz, acetone-*d*$_6$):** δ 7.80-7.72 (*m*, 2H), 7.48-7.34 (*m*, 7H), 5.64 (*s*, 1H), 3.75 (*t*, *J* = 5.8 Hz, 2H), 3.65-3.55 (*m*, 4H), 3.50-3.45 (*m*, 4H), 3.42 (*dd*, *J* = 5.9, 4.9, 2H), 2.44 (*s*, 3H), 1.90-1.80 (*m*, 2H), 1.75 (*q*, *J* = 5.7 Hz, 2H), 1.68 (*q*, *J* = 5.4 Hz, 2H); **^{13}C NMR (100 MHz, acetone-*d*$_6$):** 152.9 (C), 144.3 (C), 137.5 (C), 135.7 (C), 130.4 (CH), 129.6 (CH), 129.4 (CH), 128.2 (CH), 127.6 (CH), 109.5 (CH), 69.3 (CH_2), 67.4 (CH_2), 66.8 (CH_2), 66.7 (CH_2), 66.4 (CH_2), 47.6 (CH_2), 30.7 (2 CH_2), 29.2 (CH_2), 21.43 (CH_3); **IR (neat):** 2923, 2861, 1340, 1163, 1120, 1078, 766, 668 cm^{-1}; **HR-MS (ESI):** m/z = 446.2007 [M+H]$^+$ (calculated for $C_{24}H_{32}NO_5S$ m/z = 446.1996).

Compound 5hB:

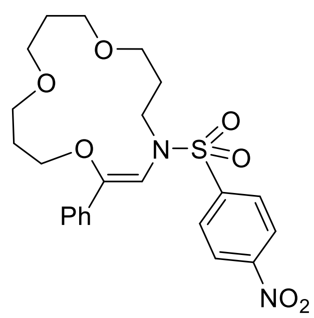

Following general procedure III, Method A: **5hB** is obtained as a yellowish solid (48.5 mg, 50% yield) starting from te triazole **1h** (67.4 mg, 0.20 mmol), oxetane **3B** and using 2 mol% of Rh_2(*S*-TCPTTL)$_4$ with a 3 hour reaction time.
Purification: column chromatography (neutral Al_2O_3, pentane/EtOAc, 7:3).
M.p. = 100.5-102.5 °C; **Rf =** 0.67 (neutral Al_2O_3, pentane/EtOAc, 7:3); **^{1}H NMR (400 MHz, acetone-*d$_6$*):** δ 8.51-8.46 (*m*, 2H), 8.19-8.14 (*m*, 2H), 7.41 (*s*, 5H), 5.64 (*s*, 1H), 3.71-3.64 (*m*, 4H), 3.60 (*t*, *J* = 5.8 Hz, 2H), 3.46 (*dd*, *J* = 5.2, 10.6 Hz, 4H), 3.33 (*dd*, *J* = 4.9, 6.0 Hz 2H), 1.97-1.88 (*m*, 2H), 1.71-1.62 (*m*, 4H) ppm; **^{13}C NMR (100 MHz, acetone-*d$_6$*):** δ 155.5 (C), 151.2 (C), 145.8 (C), 134.8 (C), 130.0 (CH), 129.6 (CH), 129.4 (CH), 128.1 (CH), 125.1 (CH), 108.4 (CH), 69.2 (CH_2), 67.0 (CH_2), 66.6 (CH_2), 66.5 (CH_2), 66.3 (CH_2), 47.9 (CH_2), 30.7 (CH_3), 30.5 (CH_3), 29.3 (CH_3) ppm; **IR (neat):** 2918, 2870, 1530, 1345, 1165, 1118, 1084, 741, 752, 604 cm^{-1}; **HR-MS (ESI):** m/z = 499.1506 [M+Na]$^+$ (calculated for $C_{23}H_{32}NNaO_5S$ m/z = 499.1509).

Compound 5kB:

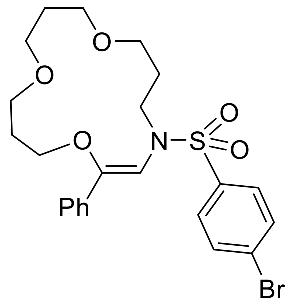

Following general procedure III, Method A: **5kB** is obtained as a white solid (42.2 mg, 40% yield) starting from triazole **1k** (74.1 mg, 0.20 mmol), oxetane **3B** and using 1 mol% of Rh_2(*S*-TCPTTL)$_4$ with a 4 hour reaction time.
Purification: column chromatography (neutral Al_2O_3, pentane/EtOAc, 7:3).
M.p. = 97.5-98.5 °C; **Rf =** 0.71 (neutral Al_2O_3, pentane/EtOAc, 7:3); **^{1}H NMR (400 MHz, acetone-*d$_6$*):** δ 7.87-7.79 (*m*, 4H), 7.41 (*s*, 5H), 5.61 (*s*, 1H), 3.71 (*t*, *J* = 5.8 Hz, 2H), 3.65-3.56 (*m*, 4H), 3.50-3.42 (*m*, 4H), 3.38 (*t*, *J* = 5.4 Hz, 2H), 1.93-1.84 (*m*, 2H), 1.75-1.64 (*m*, 4H) ppm; **^{13}C NMR (100 MHz, acetone-*d$_6$*):** δ 154.5 (C), 139.5 (C), 135.2 (C), 133.1 (CH), 130.0 (CH), 129.8 (CH) 129.4 (CH), 127.9 (CH), 127.7 (C), 108.9 (CH), 69.2 (CH_2), 67.1 (CH_2), 66.7 (CH_2), 66.7 (CH_2), 66.3 (CH_2), 47.8 (CH_2), 30.7 (CH_2), 30.6 (CH_2), 29.2 (CH_2) ppm; **IR (neat):** 2945, 2920, 2863, 1347, 1167, 1125, 1070, 751 cm^{-1}; **HR-MS (ESI):** m/z = 510.0947 [M+H]$^+$ (calculated for $C_{23}H_{29}N_2BrO_5S$ m/z = 510.0944).

Compound 5lB:

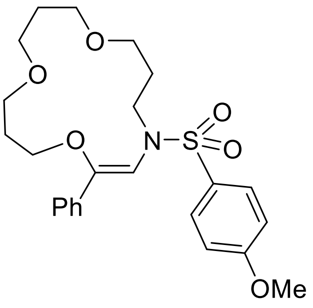

Following general procedure III, Method A: **5lB** is obtained as a colourless oil (45.8 mg, 50% yield) starting from triazole **1l** (63.4 mg, 0.2 mmol), oxetane **3B** and using 1 mol% of Rh_2(*S*-TCPTTL)$_4$ with a 24 hour reaction time.
Purification: column chromatography (neutral Al_2O_3, pentane/EtOAc, 8:2).
Rf = 0.54 (neutral Al_2O_3, pentane/EtOAc, 7:3); **^{1}H NMR (400 MHz, acetone-*d$_6$*):** δ 7.84-7.77 (*m*, 2H), 7.45-7.35 (*m*, 5H), 7.17-7.10 (*m*, 2H), 5.64 (*s*, 1H), 3.91 (*s*, 3H), 3.77 (*t*, *J* = 5.8 Hz, 2H), 3.62-3.54 (*m*, 4H), 3.50-3.41 (*m*, 6H), 1.89-1.80 (*m*, 2H), 1.77 (*q*, *J* = 5.7 Hz, 2H), 1.68 (*q*, *J* = 5.4 Hz, 2H) ppm; **^{13}C NMR (100 MHz, acetone-*d$_6$*):** δ 164.0 (C), 152.7 (C), 135.8 (C), 132.0 (C), 130.3 (CH), 129.5 (CH), 129.4 (CH), 127.6 (CH), 115.1 (CH), 109.7 (CH), 69.3 (CH_2), 67.4 (CH_2), 66.9 (CH_2), 66.7 (CH_2), 66.4 (CH_2), 56.2 (CH_3), 47.6 (CH_2), 30.8 (CH_2), 30.7 (CH_2), 29.2 (CH_2) ppm; **IR (neat):** 2926, 2862, 1254, 1158, 1121, 1080, 736 cm^{-1}; **HR-MS (ESI):** m/z = 462.1958 [M+H]$^+$ (calculated for $C_{24}H_{32}NO_6S$ m/z = 462.1945).

Compound 5mA:

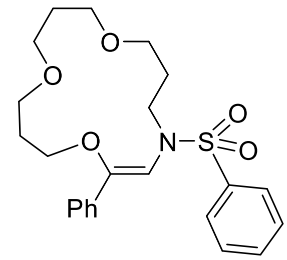

Following general procedure III, Method A: **5mA** is obtained as a colourless oil (27.8 mg, 33% yield) starting from triazole **1m** (55.4 mg, 0.19 mmol), oxetane **3B** and using 1 mol% of Rh_2(*S*-TCPTTL)$_4$ with a 10 hour reaction time. Purification: column chromatography (neutral Al_2O_3, pentane/EtOAc, 8:2).
Rf = 0.75 (neutral Al_2O_3, pentane/EtOAc, 7:3); **^{1}H NMR (400 MHz, acetone-*d$_6$*):** δ 7.92-7.85 (*m*, 2H), 7.73-7.60 (*m*, 3H), 7.44-7.35 (*m*, 5H), 5.66 (*s*, 1H), 3.72 (*t*, *J* = 5.8 Hz, 2H), 3.67-3.61 (*m*, 2H), 3.58 (*t*, *J* = 5.9 Hz, 2H), 3.50-3.44 (*m*, 4H), 3.40 (*dd*, *J* = 5.9, 4.9 Hz, 2H), 1.90-1.83 (*m*, 2H), 1.76-1.64 (*m*, 4H) ppm; **^{13}C NMR (100 MHz, acetone-*d$_6$*):** δ 153.3 (C), 140.3 (C), 135.5 (C), 133.5 (CH), 129.9 (CH), 129.6 (CH), 129.47 (CH), 128.1 (CH), 127.7 (CH), 109.4 (CH), 69.3 (CH_2), 67.3 (CH_2), 66.8 (CH_2), 66.7 (CH_2), 66.3 (CH_2), 47.7 (CH_2), 30.7 (CH_2), 30.7 (CH_2), 29.2 (CH_2) ppm; **IR (neat):** 2924, 2861, 1338, 1164, 1121, 1076, 740, 690 cm^{-1}; **HR-MS (ESI):** m/z = 432.1860 [M+H]$^+$ (calculated for $C_{23}H_{30}NO_5S$ m/z = 432.1839).

Compound (*Z*)-5pA:

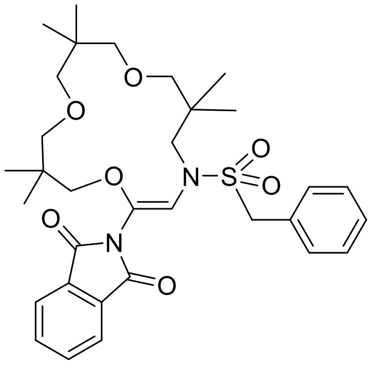

Following general procedure III, Method B: **(*Z*)-5pA** is obtained as a white solid (20.3 mg, 34% yield) starting from triazole **1p** (36.8 mg, 0.10 mmol) and 3,3-dimethyloxetane **3A**.
Purification: column chromatography (silica gel, pentane/EtOAc, 7:3).
M.p. = 176-178 °C; **Rf =** 0.58 (silica gel, pentane/EtOAc, 7:3); **^{1}H NMR (400 MHz, CDCl$_3$):** δ 7.96 (*dd*, *J* = 5.5, 3.1 Hz, 2H), 7.84 (*dd*, *J* = 5.5, 3.1 Hz, 2H), 7.55-7.48 (*m*, 2H), 7.42-7.34 (*m*, 3H), 5.70 (*s*, 1H), 4.37 (*s*, 2H), 3.55 (*s*, 2H), 3.47 (*s*, 2H), 3.28 (*s*, 2H), 3.22 (*s*, 2H), 3.21 (*s*, 2H), 3.11 (*s*, 2H), 1.01 (*s*, 6H), 0.89 (*s*, 6H), 0.82 (*s*, 6H) ppm; **^{13}C NMR (100 MHz, CDCl$_3$):** 167.0 (C), 135.4 (C), 135.1 (CH), 131.5 (C), 131.2 (CH), 129.0 (C), 128.8 (CH), 128.6 (CH), 124.4 (CH), 110.5 (CH), 80.5 (CH_2), 76.6 (CH_2), 76.1 (CH_2), 75.3 (CH_2), 72.7 (CH_2), 56.8 (CH_2), 55.7 (CH_2), 37.8 (C), 36.3 (C), 35.8 (C), 24.0 (CH_3), 22.7 (CH_3), 22.4 (CH_3) ppm; **IR (neat):** 1728, 1348, 1118, 723, 519 cm^{-1}; **HR-MS (ESI):** m/z = 599.2791 [M+H]$^+$ (calculated for $C_{32}H_{43}N_2O_7S$ m/z = 599.2786).

Compound (*E*)-5pA:

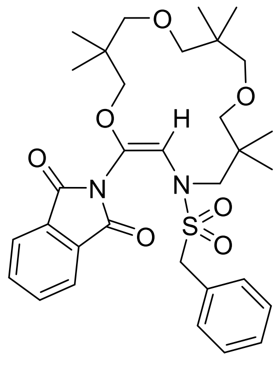

Following general procedure III, Method B: **(*E*)-5pA** is obtained as a white solid (7.7 g, 13% yield) starting from triazole **1p** (36.8 mg, 0.10 mmol) and 3,3-dimethyloxetane **3A**.
Purification: column chromatography (silica gel, pentane/EtOAc, 7:3).
M.p. = 204-206 °C; **Rf =** 0.76 (silica gel, pentane/EtOAc, 7:3); **^{1}H NMR (500 MHz, acetone-*d$_6$*):** δ 7.94-7.90 (*m*, 2H), 7.90-7.87 (*m*, 2H), 7.45-7.41 (*m*, 2H), 7.41-7.35 (*m*, 3H), 5.83 (*s*, 1H), 4.47 (*s*, 2H), 3.49 (*s*, 4H), 3.27 (*s*, 2H), 3.23 (*s*, 4H), 3.05 (*s*, 2H), 0.90 (*s*, 6H), 0.88 (*s*, 6H), 0.84 (*s*, 6H) ppm; **^{13}C NMR (100 MHz, acetone-*d$_6$*):** δ 166.7 (C), 137.7 (C), 135.3 (CH), 133.3 (C), 132.0 (CH), 129.7 (C), 129.30 (CH), 129.29 (CH), 124.3 (CH), 119.5 (CH), 76.5 (CH_2), 76.4 (CH_2), 76.0 (CH_2), 75.9 (CH_2), 75.5 (CH_2), 61.3 (CH_2), 55.2 (CH_2), 37.2 (C), 36.6 (C), 36.5 (C), 23.7 (CH_3), 22.9 (CH_3), 22.1 (CH_3) ppm; **IR (neat):** 1726, 1348, 1103, 907, 726, 533, 504 cm^{-1}; **HR-MS (ESI):** m/z = 616.3053 [M+NH$_4$]$^+$ (calculated for $C_{32}H_{46}N_3O_7S$ m/z = 616.3051).

Compound (*Z*)-5qA:

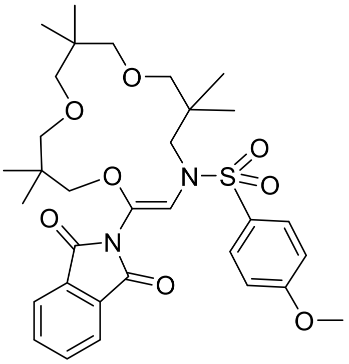

Following general procedure III, Method B: **(*Z*)-5qA** is obtained as a white solid (16.4 mg, 27% yield) starting from triazole **1q** (38.0 mg, 0.10 mmol) and 3,3-dimethyloxetane **3A**.
Purification: column chromatography (silica gel, pentane/EtOAc, 8:2).
M.p. = 145-147 °C; **Rf =** 0.50 (silica gel, pentane/EtOAc, 7:3); **^{1}H NMR (400 MHz, $CDCl_3$):** δ 7.95-7.90 (*m*, 4H), 7.85-7.79 (*m*, 2H), 7.01 (*d*, *J* = 8.9 Hz, 2H), 5.55 (*s*, 1H), 3.84 (*s*, 3H), 3.41 (*s*, 2H), 3.31 (*s*, 2H), 3.26 (*s*, 2H), 3.18 (*s*, 2H), 3.06 (*s*, 2H), 2.83 (*s*, 2H), 1.09 (*s*, 6H), 0.81 (*s*, 6H), 0.57 (*s*, 6H) ppm; **^{13}C NMR (100 MHz, $CDCl_3$):** 166.8 (C), 162.9 (C), 137.9 (C), 135.0 (CH), 131.6 (C), 131.5 (C), 129.8 (CH), 124.3 (CH), 114.2 (CH), 111.3 (CH), 80.8 (CH_2), 76.5 (CH_2), 75.8 (CH_2), 75.0 (CH_2), 72.2 (CH_2), 55.7 (CH_3), 55.6 (CH_2), 37.5 (C), 36.3 (C), 35.4 (C), 24.0 (CH_3), 22.7 (CH_3), 22.0 (CH_3) ppm.
IR (neat): 2962, 2874, 1728, 1346, 1158, 1115, 726, 454 cm^{-1}; **HR-MS (ESI):** m/z = 615.2744 $[M+H]^+$ (calculated for $C_{32}H_{43}N_2O_8S$ m/z = 615.2735).

Compound (*E*)-5qA:

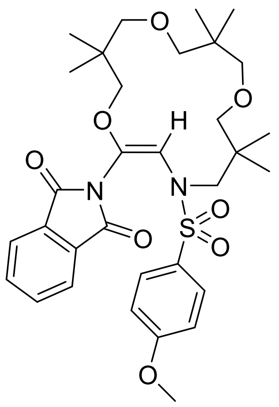

Following general procedure III, Method B: **(*E*)-5qA** is obtained as a white solid (7.1 mg, 12% yield) starting from triazole **1q** (38.0 mg, 0.10 mmol) and 3,3-dimethyloxetane **3A**.
Purification: column chromatography (silica gel, pentane/EtOAc, 8:2).
M.p. = 187-189 °C; **Rf =** 0.71 (silica gel, pentane/EtOAc, 7:3); **^{1}H NMR (400 MHz, $CDCl_3$):** δ 7.93 (*dd*, *J* = 5.5, 3.0 Hz, 2H), 7.81-7.77 (*m*, 2H), 7.75 (*dd*, *J* = 5.5, 3.0, 2H), 7.02-6.97 (*m*, 2H), 5.32 (*s*, 1H), 3.88 (*s*, 3H), 3.52 (*s*, 2H), 3.40 (*s*, 2H), 3.17 (*s*, 2H), 3.14 (*s*, 2H), 3.05 (*s*, 2H), 2.98 (*s*, 2H), 0.96 (*s*, 3H), 0.84 (*s*, 3H) and 0.83 (*s*, 3H) ppm; **^{13}C NMR (100 MHz, $CDCl_3$):** 166.3 (C), 163.2 (C), 139.3 (C), 134.3 (CH), 132.4 (C), 130.3 (C), 130.0 (CH), 124.0 (CH), 118.0 (CH), 114.3 (CH), 76.1 (CH_2), 75.9 (CH_2), 75.4 (CH_2), 75.3 (CH_2), 74.8 (CH_2), 60.3 (CH_2), 55.7 (CH_3), 36.6 (C), 36.04 (C), 36.02 (C), 23.8 (CH_3), 22.7 (CH_3), 22.0 (CH_3) ppm; **IR (neat):**2925, 2852, 1730, 1356, 1165, 1102, 751, 559 cm^{-1}; **HR-MS (ESI):** m/z = 615.2726 $[M+H]^+$ (calculated for $C_{32}H_{43}N_2O_8S$ m/z = 615.2735).

Analysis data for macrocycles 6

Compound 6gA:

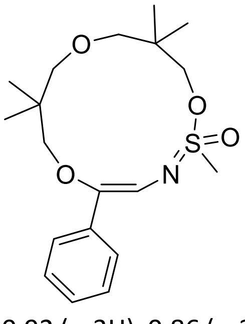

Following general procedure III, Method A: **6gA** is obtained as a white solid (41.5 mg, 57% yield) starting from triazole **1g** (44.8 mg, 0.2 mmol), 3,3-dimethyloxetane **3A** and using 1 mol% of Rh_2(*S*-TCPTTL)$_4$ with a 3 hour reaction time.
Purification: column chromatography (silica gel, pentane/Et_2O, 8:2).
M.p. = 84-85 °C; **Rf =** 0.53 (silica gel, pentane/Et_2O, 7:3); **^{1}H NMR (400 MHz, acetone-d_6):** δ 7.46-7.39 (*m*, 2H), 7.31-7.24 (*m*, 2H), 7.17-7.10 (*m*, 1H), 6.61 (*s*, 1H), 4.52 (*d*, *J* = 9.2 Hz, 1H), 3.76 (*d*, *J* = 7.5 Hz, 1H), 3.60-3.50 (*m*, 3H), 3.30-3.24 (*m*, 4H), 3.08 (*d*, *J* = 8.3 Hz, 1H), 3.02 (*d*, *J* = 7.5 Hz, 1H), 1.01 (*s*, 3H), 0.94 (*s*, 3H), 0.92 (*s*, 3H), 0.86 (*s*, 3H) ppm; **^{13}C NMR (100 MHz, acetone-d_6):** δ 143.4 (C), 137.9 (C), 129.2 (CH), 126.6 (CH), 124.1 (CH), 116.8 (CH), 76.3 (CH_2), 75.4 (CH_2), 73.9 (CH_2), 69.8 (CH_2), 40.8 (CH_3), 37.1 (C), 36.7 (C), 22.7 (CH_3), 22.3 (CH_3), 21.6 (CH_3), 21.6 (CH_3) ppm; **IR (neat):** 2950, 2857, 1636, 1305, 1245, 1203, 1114, 1083, 1028, 936, 811, 762, 690 cm^{-1}; **HR-MS (ESI):** m/z = 390.1707 $[M+Na]^+$ (calculated for $C_{19}H_{29}NNaO_4S$ m/z = 390.1710).

Compound 6nA:

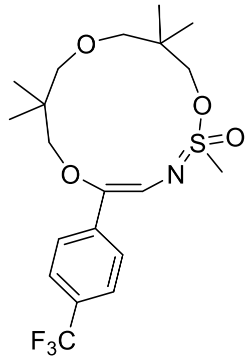

Following general procedure III, Method A: **6nA** is obtained as white solid (41.8 mg, 48% yield) starting from triazole **1n** (58.2 mg, 0.2 mmol), 3,3-dimethyloxetane **3A** and using 2 mol% of Rh_2(*S*-TCPTTL)$_4$ with a 7 hour reaction time.

Purification: column chromatography (silica gel, pentane/EtOAc, 9:1).

M.p. = 62-64°C; **Rf =** 0.54 (silica gel, pentane/EtOAc, 8:2); **^{1}H NMR (400 MHz, acetone-*d$_6$*):** δ 7.66-7.56 (*m*, 4H), 6.85 (*s*, 1H), 4.56 (*d*, *J* = 9.2 Hz, 1H), 3.76 (*d*, *J* = 7.5 Hz, 1H), 3.65-3.58 (*m*, 2H), 3.54 (*d*, *J* = 7.9 Hz, 1H), 3.31 (*s*, 3H), 3.29 (*d*, *J* = 8.5 Hz, 1H), 3.08 (*d*, *J* = 8.5 Hz, 1H), 3.03 (*d*, *J* = 7.6 Hz, 1H), 1.02 (*s*, 3H), 0.95 (*s*, 3H), 0.93 (*s*, 3H), 0.87 (*s*, 3H) ppm; **^{13}C NMR (100 MHz, acetone-*d$_6$*):** δ 142.2 (C), 141.8 (C), 127.5 (*q*, *J* = 32.0 Hz, C), 126.1 (*q*, *J* = 3.9 Hz, CH), 125.7 (*q*, *J* = 270.5 Hz, CF_3), 124.0 (CH), 120.2 (CH), 76.4 (CH_2), 75.3 (CH_2), 73.8 (CH_2), 70.2 (CH_2), 40.8 (CH_3), 37.1 (C), 36.7 (C), 22.6 (CH_3), 22.3 (CH_3), 21.6 (CH_3), 21.6 (CH_3) ppm; **IR (neat):** 2956, 2870, 1638, 1607, 1326, 1301, 1323, 1205, 1102, 1078, 1026, 943, 822 cm^{-1}; **HR-MS (ESI):** m/z = 458.1578 $[M+H]^+$ (calculated for $C_{20}H_{28}F_3NNaO_4S$ m/z = 458.1583).

Compound 6oA:

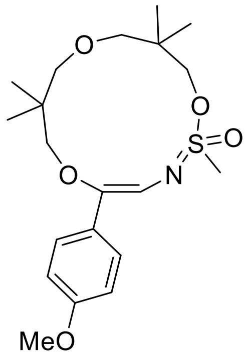

Following general procedure III, Method A: **6oA** is obtained as a colorless oil (46.2 mg, 61% yield) starting from triazole **1o** (49.1 mg, 0.19 mmol), 3,3-dimethyloxetane **3A** and using 1 mol% of Rh_2(*S*-TCPTTL)$_4$ with a 3 hour reaction time.

Purification: column chromatography (neutral Al_2O_3, pentane/EtOAc, 8:2).

Rf = 0.39 (silica gel, pentane/EtOAc, 8:2); **^{1}H NMR (400 MHz, acetone-*d$_6$*):** δ 7.34 (*d*, *J* = 8.8 Hz, 2H), 6.86 (*d*, *J* = 8.9 Hz, 2H), 6.42 (s, 1H), 4.49 (*d*, *J* = 9.2 Hz, 1H), 3.78 (*s*, 3H), 3.74 (*d*, *J* = 7.5 Hz, 1H), 3.57 (*d*, *J* = 9.2 Hz, 1H), 3.52 (*s*, 2H), 3.26 (*d*, *J* = 8.6 Hz, 1H), 3.24 (*s*, 3H), 3.07 (*d*, *J* = 8.5 Hz, 1H), 3.02 (*d*, *J* = 7.4 Hz, 1H), 0.99 (*s*, 3H), 0.93 (*s*, 3H), 0.91 (*s*, 3H), 0.85 (*s*, 3H) ppm; **^{13}C NMR (100 MHz, acetone-*d$_6$*):** δ 159.2 (C), 143.5 (C), 130.4 (C), 125.6 (CH), 114.7 (CH), 114.6 (CH), 76.2 (CH_2), 75.5 (CH_2), 73.9 (CH_2), 69.7 (CH_2), 55.5 (CH_3), 40.8 (CH_3), 37.1 (C), 36.7 (C), 22.7 (CH_3), 22.4 (CH_3), 21.6 (CH_3), 21.6 (CH_2) ppm; **IR (neat):** 2959, 2871, 1638, 1510, 1303, 1242, 1206, 1078, 1023, 929, 800 cm^{-1}; **HR-MS (ESI):** m/z = 398.1996 $[M+H]^+$ (calculated for $C_{20}H_{32}NO_5S$ m/z = 398.1996).

Analysis data for byproducts 6aA and 10aA

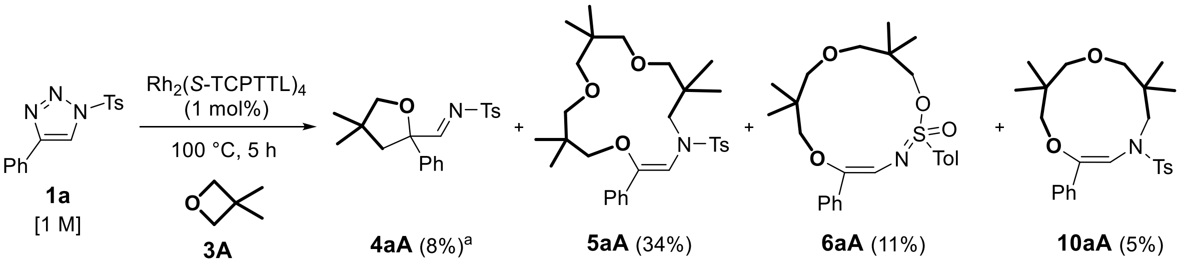

Compound 6aA:

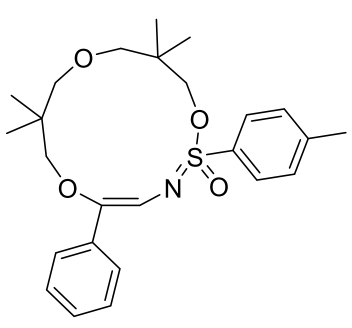

Following general procedure III, Method A: **6aA** is obtained as a colourless oil (9.3 mg, 11% yield) starting from triazole **1a** (57.2 mg, 0.19 mmol), 3,3-dimethyloxetane **3A** and using 1 mol% of Rh_2(*S*-TCPTTL)$_4$ with a 5 hour reaction time.

Purification: column chromatography (silica gel, pentane/Et_2O, 8:2).

Rf = 0. 53 (silica gel, pentane/Et_2O, 8:2); **^{1}H NMR (400 MHz, $CDCl_3$):** δ 7.87 (*d*, *J* = 8.4 Hz, 2H), 7.51-7.41 (*m*, 4H), 7.26 (*t*, *J* = 7.8 Hz, 2H), 7.17-7.09 (*m*, 1H), 6.63 (*s*, 1H), 4.57 (*d*, *J* = 9.2 Hz, 1H), 3.85 (*d*, *J* = 9.1 Hz, 1H), 3.78-3.71 (*m*, 2H), 3.67 (*d*, *J* = 8.1 Hz, 1H), 3.27 (*d*, *J* = 8.4 Hz, 1H), 3.15 (*t*, *J* = 8.1 Hz, 2H), 2.45 (*s*, 3H), 1.04 (*s*, 3H), 0.96 (*s*, 3H), 0.93 (*s*, 3H), 0.84 (*s*, 3H) ppm; **^{13}C NMR (100 MHz, $CDCl_3$):** δ 145.2 (C), 144.1 (C), 137.8 (C), 137.1 (C), 130.8 (CH), 129.2 (CH), 128.3 (CH), 126.8 (CH), 123.9 (CH), 116.2 (CH), 76.1 (CH_2), 75.4 (CH_2), 74.1 (CH_2), 72.0 (CH_2), 37.2 (C), 36.8 (C), 22.7 (CH_3), 22.4 (CH_3), 21.7 (CH_3), 21.6 (CH_3), 21.5 (CH_3) ppm; **IR (neat):** 2924, 2854, 1633, 1311, 1249, 1204, 1074, 931, 762 cm^{-1}; **HR-MS (ESI):** m/z = 444.2199 $[M+H]^+$ (calculated for $C_{25}H_{34}NO_4S$ m/z = 444.2203).

Compound 10aA:

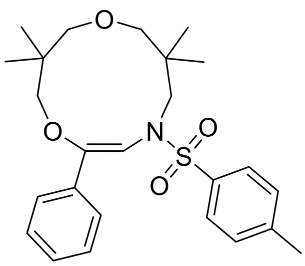

Following general procedure III, Method A: **10aA** is obtained as a colourless oil (4.2 mg, 5% yield) starting from triazole **1a** (57.2 mg, 0.19 mmol), 3,3-dimethyloxetane **3A** and using 1 mol% of Rh_2(*S*-TCPTTL)$_4$ with a 5 hour reaction time.

Purification: column chromatography (silica gel, pentane/Et_2O, 8:2).

Rf = 0.29 (silica gel, pentane/Et_2O, 8:2); **^{1}H NMR (400 MHz, $CDCl_3$):** δ 7.68-7.60 (*m*, 2H), 7.52-7.46 (*m*, 2H), 7.43-7.33 (*m*, 5H), 5.24 (*s*, 1H), 3.64 (*s*, 2H), 3.44 (*s*, 2H), 3.35 (*s*, 2H), 3.08 (*s*, 2H), 2.41 (*s*, 3H), 0.98 (s, 6H), 0.91 (s, 6H) ppm; **^{13}C NMR (100 MHz, $CDCl_3$):** δ 154.9 (C), 144.2 (C), 136.8 (C), 136.6 (C), 130.4 (CH), 129.5 (CH), 129.5 (CH), 128.4 (CH), 127.7 (CH), 112.0 (CH), 77.7 (CH_2), 77.4 (CH_2), 77.2 (CH_2), 61.6 (CH_2), 37.6 (C), 37.1 (C), 25.0 (CH_3), 22.7 (CH_3), 21.4 (CH_3) ppm; **IR (neat):** 2923, 2854, 1727, 1346, 1262, 1110, 1081, 805, 768, 739, 654 cm^{-1}; **HR-MS (ESI):** m/z = 466.2018 $[M+Na]^+$ (calculated for $C_{25}H_{33}NNaO_4S$ m/z = 466.2023).

8. General procedure IV: hydrogenation of macrocycles 5

To a stirred solution of the corresponding macrocycle **5** (0.1 mmol) in 10 mL of MeOH was added 40% w/w $Pd(OH)_2/C$ (20% Pd, 50% water) for macrocycles of type **5A** or 20% w/w Pd/C (10%) for macrocycles **5B** and the heterogeneous mixture was stirred under hydrogen (1 atm) for 3 h. Then it was filtered over celite and washed with 20 mL of MeOH. The organic phase was concentrated to afford the desired product **16** without further purification.

Analysis data for hydrogenated products 16

Compound 16aA:

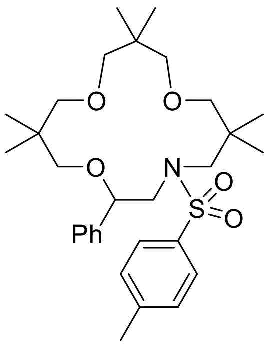

Following general procedure IV, **16aA** is obtained as a white solid (57.9 mg, 95% yield) starting from **5aA** (61.1 mg, 0.11 mmol). **M.p. =** 104-106 °C; **Rf =** 0.62 (silica gel, pentane/Et_2O, 7:3); **^{1}H NMR (400 MHz, $CDCl_3$):** δ 7.69 (*d*, *J* = 8.2 Hz, 2H), 7.39-7.27 (*m*, 5H), 7.25-7.20 (*m*, 2H), 4.46 (*t*, *J* = 6.5 Hz, 1H), 3.59 (*d*, *J* = 15.3 Hz, 1H), 3.42-3.28 (*m*, 6H), 3.19-3.08 (*m*, 3H), 2.95-2.77 (*m*, 4H), 2.40 (*s*, 3H), 1.16 (*s*, 3H), 0.98 (*s*, 3H), 0.87 (*s*, 3H), 0.84 (*s*, 3H), 0.71 (*s*, 3H), 0.64 (*s*, 3H) ppm; **^{13}C NMR (100 MHz, $CDCl_3$):** δ 143.1 (C), 140.3 (C), 136.9 (C), 129.7 (CH), 128.6 (CH), 128.0 (CH), 127.8 (CH), 126.9 (CH), 80.8 (CH), 80.5 (CH_2), 77.0 (CH_2), 75.9 (CH_2), 75.8 (CH_2), 75.0 (CH_2), 56.8 (CH_2), 56.2 (CH_2), 37.5 (C), 36.5 (C), 36.1 (C), 24.5 (CH_3), 24.1 (CH_3), 22.7 (CH_3), 22.6 (CH_3), 22.6 (CH_3), 22.4 (CH_3), 21.6 (CH_3) ppm; **IR (neat):** 2959, 2870, 1338, 1158, 1103, 988, 740, 701, 657 cm^{-1}; **HR-MS (ESI):** m/z = 532.3075 $[M+H]^+$ (calculated for $C_{30}H_{46}NO_5S$ m/z = 532.3091).

Compound 16hA:

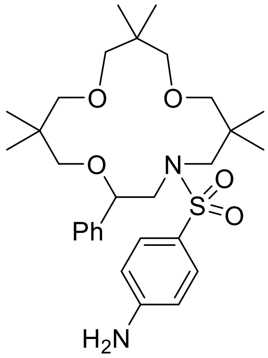

Following general procedure IV, **16hA** is obtained as a white solid (41.6 mg, 92% yield) starting from **5hA** (47.8 mg, 0.09 mmol). **M.p. =** 92-94 °C; **Rf =** 0.73 (silica gel, pentane/EtOAc, 1:1); **^{1}H NMR (400 MHz, $CDCl_3$):** δ 7.56 (*d*, *J* = 8.6 Hz, 2H), 7.37-7.22 (*m*, 5H), 6.65 (*d*, *J* = 8.5 Hz, 2H), 4.52 (*dd*, *J* = 8.6, 4.0 Hz, 1H), 4.07 (*br s*, 2H), 3.55 (*d*, *J* = 15.1 Hz, 1H), 3.41-3.20 (*m*, 8H), 3.13 (*d*, *J* = 8.7 Hz, 1H), 2.93 (*t*, *J* = 8.2 Hz, 2H), 2.88-2.79 (*m*, 2H), 1.15 (*s*, 3H), 0.96 (*s*, 3H), 0.86 (*s*, 3H), 0.84 (*s*, 3H), 0.74 (*s*, 3H), 0.70 (*s*, 3H) ppm; **^{13}C NMR (100 MHz, $CDCl_3$):** δ 150.3 (C), 140.7 (C), 129.9 (CH), 128.5 (CH), 128.2 (C), 127.9 (CH), 127.0 (CH), 114.2 (CH), 81.3 (CH), 80.1 (CH_2), 77.4 (CH_2), 76.0 (CH_2), 75.9 (CH_2), 75.2 (CH_2), 57.2 (CH_2), 56.9 (CH_2), 37.5 (C), 36.6 (C), 36.2 (C), 24.4 (CH_3), 24.3 (CH_3), 22.7 (CH_3), 22.63 (CH_3), 22.61 (CH_3), 22.5 (CH_3) ppm; **IR (neat):** 3447, 3360, 2958, 2863, 1598, 1316, 1147, 1114, 1087, 767, 703 cm^{-1}; **HR-MS (ESI):** m/z = 533.3046 $[M+H]^+$ (calculated for $C_{29}H_{45}N_2O_5S$ m/z = 533.3044).

Compound 16lA:

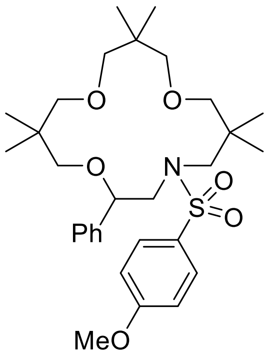

Following general procedure IV, **16lA** is obtained as a white solid (45.6 mg, 95% yield) starting from **5lA** (47.6 mg, 0.09 mmol).

M.p. = 89-91 °C ; **Rf =** 0.44 (silica gel, pentane/Et_2O, 7:3); **^{1}H NMR (400 MHz, $CDCl_3$):** δ 7.83-7.74 (*m*, 2H), 7.41-7.27 (*m*, 5H), 7.15-7.07 (*m*, 2H), 4.56 (*dd*, *J* = 9.3, 3.8 Hz, 1H), 3.90 (*s*, 3H), 3.56 (*d*, *J* = 15.3 Hz, 1H), 3.45-3.33 (*m*, 5H), 3.33-3.23 (*m*, 2H), 3.16 (*dd*, *J* = 8.5, 4.5 Hz, 2H), 2.94 (*d*, *J* = 2.7 Hz, 1H), 2.91 (*d*, *J* = 9.6 Hz, 1H), 2.85 (*d*, *J* = 8.4 Hz, 1H), 2.80-2.77 (*m*, 1H), 1.17 (*s*, 3H), 0.98 (*s*, 3H), 0.88 (*s*, 3H), 0.85 (*s*, 3H), 0.73 (*s*, 3H), 0.66 (*s*, 3H) ppm; **^{13}C NMR (100 MHz, $CDCl_3$):** δ 163.8 (C), 141.2 (C), 132.4 (C), 130.6 (CH), 129.3 (CH), 128.8 (CH), 127.8 (CH), 115.1 (CH), 81.4 (CH), 81.1 (CH_2), 77.3 (CH_2), 76.3 (CH_2), 76.2 (CH_2), 75.4 (CH_2), 57.6 (CH_2), 56.9 (CH_2), 56.1 (CH_3), 37.9 (C), 37.0 (C), 36.5 (C), 24.8 (CH_3), 24.2 (CH_3), 22.9 (CH_3), 22.8 (CH_3), 22.7 (2 CH_3) ppm; **IR (neat):** 2961, 2852, 1597, 1495, 1454, 1337, 1259, 1154, 1121, 1105, 1025, 763, 699 cm^{-1}; **HR-MS (ESI):** m/z = 548.3032 $[M+H]^+$ (calculated for $C_{30}H_{46}NO_6S$ m/z = 548.3040).

Compound 16mA:

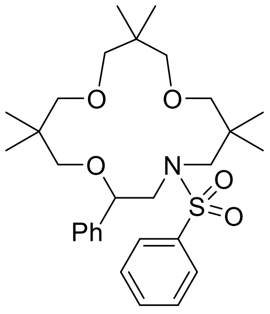

Following general procedure IV, **16mA** is obtained as a white solid (49.8 mg, 97% yield) starting from **5mA** (51.0 mg, 0.10 mmol).

M.p. = 115.5-117.5 °C; **Rf =** 0.61 (silica gel, pentane/Et_2O, 7:3); **^{1}H NMR (400 MHz, $CDCl_3$):** δ 7.86-7.77 (*m*, 2H), 7.58-7.52 (*m*, 1H), 7.52-7.45 (*m*, 2H), 7.38-7.27 (*m*, 3H), 7.25-7.19 (*m*, 2H), 4.44 (*t*, *J* = 6.5 Hz, 1H), 3.62 (*d*, *J* = 15.3 Hz, 1H), 3.42-3.26 (*m*, 6H) 3.17-3.07 (*m*, 3H), 2.98-2.88 (*m*, 2H), 2.82 (*dd*, *J* = 13.5, 8.7 Hz, 2H), 1.16 (*s*, 3H), 0.99 (*s*, 3H), 0.87 (*s*, 3H), 0.84 (*s*, 3H), 0.71 (*s*, 3H), 0.64 (*s*, 3H) ppm; **^{13}C NMR (100 MHz, $CDCl_3$):** δ 140.2 (C), 140.0 (C), 132.4 (CH), 129.1 (CH), 128.6 (CH), 128.1 (CH), 127.8 (CH), 126.9 (CH), 80.7 (CH), 80.6 (CH_2), 77.1 (CH_2), 76.0 (CH_2), 75.9 (CH_2), 75.1 (CH_2), 56.7 (CH_2), 56.1 (CH_2), 37.5 (C), 36.5 (C), 36.1 (C), 24.5 (CH_3), 24.1 (CH_3), 22.7 (CH_3), 22.62 (CH_3), 22.61 (CH_3), 22.5 (CH_3) ppm; **IR (neat):** 2958, 2851, 1335, 1160, 1106. 1084, 774, 748 cm^{-1}; **HR-MS (ESI):** m/z = 518.2951 $[M+H]^+$ (calculated for $C_{29}H_{44}NO_5S$ m/z = 518.2935).

Compound 16aB:

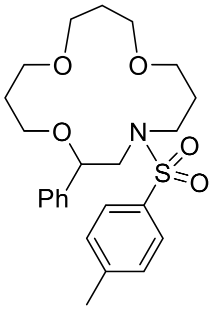

Following general procedure IV, **16aB** is obtained as a white solid (43.4 mg, 90% yield) starting from **5aB** (48.2 mg, 0.11 mmol).

M.p. = 117.5-119.5 °C; **Rf =** 0.52 (silica gel, pentane/EtOAc, 1:1); **^{1}H NMR (400 MHz, $CDCl_3$):** δ 7.68-7.59 (*m*, 2H), 7.39-7.27 (*m*, 5H), 7.25-7.21 (*m*, 2H), 4.57 (*dd*, *J* = 8.7, 2.3 Hz, 1H), 3.72-3.46 (*m*, 10H), 3.42 (*dd*, *J* = 14.9, 2.3 Hz, 1H), 3.37-3.29 (*m*, 1H), 3.19-3.09 (*m*, 1H), 3.03 (*dd*, *J* = 14.9, 8.6 Hz, 1H), 2.39 (*s*, 3H), 2.26-2.13 (*m*, 1H), 1.94-1.65 (*m*, 5H) ppm ; **^{13}C NMR (100 MHz, $CDCl_3$):** δ 143.3 (C), 140.5 (C), 136.4 (C), 129.8 (CH), 128.7 (CH), 128.1 (CH), 127.4 (CH), 126.7 (CH), 82.9 (CH), 69.1 (CH_2), 67.1 (CH_2), 66.2 (CH_2), 66.0 (CH_2), 65.9 (CH_2), 56.7 (CH_2), 47.9 (CH_2), 30.4 (CH_2), 30.1 (CH_2), 28.9 (CH_2), 21.6 (CH_3) ppm; **IR (neat):** 2917, 1660, 1444, 1302, 1120, 703, 552 cm^{-1}; **HR-MS (ESI):** m/z = 448.2148 $[M+H]^+$ (calculated for $C_{24}H_{34}NO_5S$ m/z = 448.2152).

Compound 16hB:

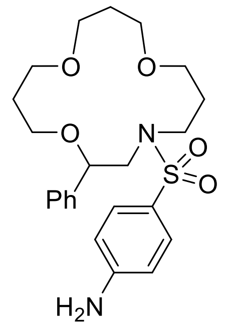

Following general procedure IV, **16hB** is obtained as a white solid (44.2 mg, 98% yield) starting from **5hB** (47.8 mg, 0.11 mmol).
M.p. = 49-51 °C; **Rf =** 0.31 (silica gel, pentane/EtOAc, 3:7); **^{1}H NMR (400 MHz, acetone-*d$_6$*):** δ 7.52-7.43 (*m*, 2H), 7.41-7.34 (*m*, 4H), 7.33-7.27 (*m*, 1H), 6.77-6.70 (*m*, 2H), 5.44 (*br s*, 2H), 4.55 (*dd*, *J* = 8.6, 2.6 Hz, 1H), 3.65-3.36 (*m*, 10H), 3.35-3.24 (*m*, 2H), 3.18-3.04 (*m*, 2H), 2.21-2.09 (*m*, 1H), 1.91-1.80 (*m*, 1H), 1.78-1.61 (*m*, 4H) ppm; **^{13}C NMR (100 MHz, acetone-*d$_6$*):** δ 153.5 (C), 141.7 (C), 130.1 (CH), 129.4 (CH), 128.6 (CH), 127.4 (CH), 126.5 (C), 114.1 (CH), 83.2 (CH), 69.6 (CH_2), 67.2 (CH_2), 66.4 (CH_2), 66.3 (CH_2), 66.2 (CH_2), 57.4 (CH_2), 48.0 (CH_2), 31.2 (CH_2), 30.8 (CH_2), 29.7 (CH_2). ppm; **IR (neat):** 3464, 3368, 2925, 2864, 1596, 1312, 1139, 1116, 1089, 701 cm^{-1}; **HR-MS (ESI):** m/z = 449.2112 [M+H]$^+$ (calculated for $C_{23}H_{33}N_2O_5S$ m/z = 449.2105).

Compound 16lB:

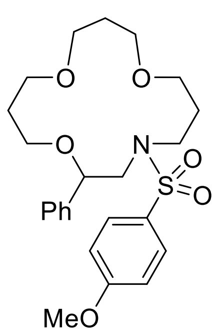

Following general procedure IV, **16lB** is obtained as a white solid (49.5 mg, 96% yield) starting from **5lB** (51.2 mg, 0.11 mmol).
M.p. = 80-82 °C; **Rf =** 0.48 (silica gel, pentane/EtOAc, 1:1); **^{1}H NMR (400 MHz, acetone-*d$_6$*):** δ 7.74 (*d*, *J* = 8.9 Hz, 2H), 7.43-7.27 (*m*, 5H), 7.08 (*d*, *J* = 8.9 Hz, 2H), 4.56 (*dd*, *J* = 8.7, 2.8 Hz, 1H), 3.88 (*s*, 3H), 3.64-3.39 (*m*, 10H), 3.36-3.26 (*m*, 2H), 3.25-3.13 (*m*, 2H) 2.20-2.09 (*m*, 1H), 1.93-1.81 (*m*, 1H), 1.78-1.61 (*m*, 4H) ppm; **^{13}C NMR (100 MHz, acetone-*d$_6$*):** δ 163.8 (C), 141.5 (C), 132.2 (C), 130.2 (CH), 129.4 (CH), 128.7 (CH), 127.5 (CH), 115.1 (CH), 82.9 (CH), 69.4 (CH_2), 67.2 (CH_2), 66.3 (CH_2), 66.3 (CH_2), 66.2 (CH_2), 57.1 (CH_2), 56.1 (CH_3), 47.9 (CH_2), 31.2 (CH_2), 30.8 (CH_2), 29.6 (CH_2) ppm; **IR (neat):** 2923, 2864, 1338, 1255, 1154, 1115, 1091, 701, 557 cm^{-1}; **HR-MS (ESI):** m/z = 464.2090 [M+H]$^+$ (calculated for $C_{24}H_{34}NO_6S$ m/z = 464.2101).

Compound 16mB:

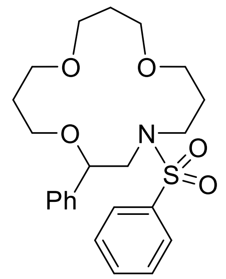

Following general procedure IV, **16mB** is obtained as a white solid (42.5 mg, 99% yield) starting from **5mB** (42.9 mg, 0.10 mmol).
M.p. = 87-89 °C; **Rf =** 0.54 (silica gel, pentane/EtOAc, 1:1); **^{1}H NMR (400 MHz, acetone-*d$_6$*):** δ 7.87-7.79 (*m*, 2H), 7.69-7.55 (*m*, 3H), 7.42-7.28 (*m*, 5H), 4.56 (*dd*, *J* = 8.7, 2.9 Hz, 1H), 3.68-3.15 (*m*, 14H), 2.13 (*m*, 1H), 1.87 (*m*, 1H), 1.68 (*m*, 4H); **^{13}C NMR (100 MHz, acetone-*d$_6$*):** δ 141.3 (C), 140.6 (C), 133.4 (CH), 130.0 (CH), 129.4 (CH), 128.8 (CH), 128.1 (CH), 127.5 (CH), 82.8 (CH), 69.3 (CH_2), 67.2 (CH_2), 66.3 (CH_2), 66.3 (CH_2), 66.2 (CH_2), 56.9 (CH_2), 47.8 (CH_2), 31.2 (CH_2), 30.7 (CH_2), 29.5 (CH_2) ppm; **IR (neat):** 2922, 2862, 1338, 1159, 1118, 1090, 736, 696 cm^{-1}; **HR-MS (ESI):** m/z = 434.2008 [M+H]$^+$ (calculated for $C_{23}H_{32}NO_5S$ m/z = 434.1996).

9. Deprotection of *N*-macrocycles 16aA and 16aB

In a 2 mL screw-cap vial equipped with a magnetic stirring bar the corresponding macrocycle (0.1 mmol) was dissolved in 1 mL of *n*-Bu_2O. Then $LiAlH_4$ (5 equiv) was slowly added at 0°C. The vial was flushed with nitrogen and capped. After 4 hours stirring at 120 °C, the reaction mixture was cooled down to room temperature and 200 µL of H_2O were added dropwise at 0 °C. The mixture was stirred at room temperature during 15 minutes, filtered over celite and purified by flash chromatography (neutral Al_2O_3, pentane/EtOAc 1:1).

Compound 17aA:

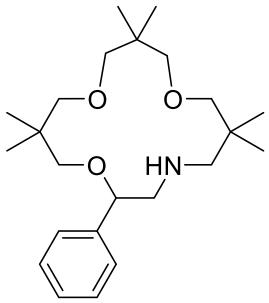

17aA is obtained as a colorless oil (25.2 mg, 70% yield) starting from **16aA** (50.7 mg, 0.09 mmol).
Rf = 0.53 (neutral Al_2O_3, pentane/EtOAc 1:1); **^{1}H NMR (400 MHz, $CDCl_3$):** δ 7.35-7.29 (*m*, 4H), 7.26-7.22 (*m*, 1H), 4.37 (*d*, *J* = 9.6 Hz, 1H), 3.55-3.42 (*m*, 4H), 3.30 (*d*, *J* = 8.7 Hz, 1H), 3.11 (*d*, *J* = 8.6 Hz, 1H), 3.01-2.81 (*m*, 5H), 2.59-2.45 (*m*, 3H), 3.07 (*s*, 3H), 0.89 (*s*, 3H), 0.87 (*s*, 6H), 0.83 (*s*, 3H), 0.80 (*s*, 3H) ppm; **^{13}C NMR (100 MHz, $CDCl_3$):** δ 142.0 (C), 128.3 (CH), 127.4 (CH), 126.5 (CH), 80.43 (CH_2), 80.40 (CH), 76.7 (CH_2), 76.3 (CH_2), 75.7 (CH_2), 75.3 (CH_2), 60.3 (CH_2), 58.9 (CH_2), 36.2 (C), 36.2 (C), 35.4 (C), 25.0 (CH_3), 23.6 (CH_3), 23.0 (CH_3), 22.7 (CH_3), 22.7 (CH_3), 22.5 (CH_3) ppm; **IR (neat):** 2957, 2871, 1109, 700 cm^{-1}; **HR-MS (ESI):** m/z = 378.3007 $[M+H]^+$ (calculated for $C_{23}H_{40}NO_3$ m/z = 378.3003).

Compound 17aB:

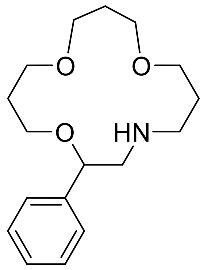

17aB is obtained as a colorless oil (26.6 mg, 90 % yield) starting **from 16aB** (45.1 mg, 0.10 mmol).
Rf = 0.25 (neutral Al_2O_3, pentane/EtOAc 1:1); **^{1}H NMR (400 MHz, $CDCl_3$):** δ 7.38-7.30 (*m*, 4H), 7.29-7.24 (*m*, 1H), 4.48 (*dd*, *J* = 9.9, 2.2 Hz, 1H), 3.81-3.71 (*m*, 1H), 3.70-3.60 (*m*, 5H), 3.60-3.53 (*m*, 2H), 3.52-3.44 (*m*, 2H), 2.96-2.85 (*m*, 2H), 2.82-2.74 (*m*, 1H), 2.63 (*dd*, *J* = 12.9, 2.4 Hz, 1H), 1.96-1.74 (*m*, 6H) ppm; **^{13}C NMR (100 MHz, $CDCl_3$):** δ 141.5 (C), 128.5 (CH), 127.6 (CH), 126.5 (CH), 81.0 (CH), 71.0 (CH_2), 67.5 (CH_2), 67.3 (CH_2), 67.2 (CH_2), 65.5 (CH_2), 58.0 (CH_2), 48.5 (CH_2), 30.44 (CH_2), 30.41 (CH_2), 29.8 (CH_2) ppm; **IR (neat):** 2919, 2860, 1112, 701, 516 cm^{-1}; **HR-MS (ESI):** m/z = 294.2064 $[M+H]^+$ (calculated for $C_{17}H_{28}NO_3$ m/z = 294.2064).

10. Synthesis of aldehyde 18

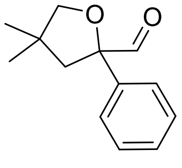

In a 2 mL screw-cap vial equipped with a magnetic stirring bar, Rh_2(*S*-TCPTTL)$_4$ (2.96 mg, 0.0015 mmol, 1 mol%), *N*-tosyl-4-phenyl-1,2,3-triazole **1a** (44.9 mg, 0.15 mmol, 1 equiv) and 3,3-dimethyloxetane **3A** (23 µL, 0.225 mmol, 1.5 equiv) were dissolved in 1.5 mL of anhydrous CH_2Cl_2 (0.1 M). The vial was flushed with nitrogen, capped and stirred at 100 °C for 3 h. The solution was cooled to room temperature and 180 mg of silica gel were added. The obtained suspension was stirred at room temperature for 24 h until hydrolysis of the imine was complete. Solvent was removed under reduced pressure and the pre-absorbed silica was purified by column chromatography (silica gel, pentane/Et_2O 9:1) to afford aldehyde **18** as a colorless oil (21.2 mg, 69% yield).
Rf = 0.45 (silica gel, pentane/Et_2O, 9:1); **^{1}H NMR (400 MHz, $CDCl_3$):** δ 9.53 (*s*, 1H), 7.44-7.33 (*m*, 4H), 7.32-7.27 (*m*, 1H), 3.73 (*d*, *J* = 8.3 Hz, 1H), 3.67 (*d*, *J* = 8.2 Hz, 1H), 2.69 (*d*, *J* = 12.7 Hz, 1H), 1.92 (*d*, *J* = 12.7 Hz, 1H), 1.07 (*s*, 3H), 1.05 (*s*, 3H) ppm; **^{13}C NMR (100 MHz, $CDCl_3$):** 199.7 (CH), 139.7 (C), 128.8 (CH), 127.9 (CH), 125.6 (CH), 91.3 (C), 80.4 (CH_2), 48.5 (CH_2), 40.4 (C), 26.3 (CH_3), 26.1 (CH_3) ppm; **IR (neat):** 2975, 1712, 1452, 1369, 1271, 1175, 1112, 1070, 1026, 709, 501 cm^{-1}; **HR-MS (ESI):** m/z = 205.1225 $[M+H]^+$ (calculated for $C_{13}H_{16}O_2$ m/z = 205.1223).

11. Synthesis of dithiane 19

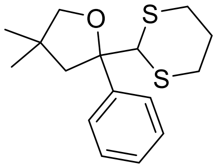

In a 2 mL screw-cap vial equipped with a magnetic stirring bar, Rh_2(*S*-TCPTTL)$_4$ (2.96 mg, 0.0015 mmol, 1 mol%), *N*-tosyl-4-phenyl-1,2,3-triazole **1a** (44.7 mg, 0.15 mmol, 1 equiv) and 3,3-dimethyloxetane **3A** (23 µL, 0.225 mmol, 1.5 equiv) were dissolved in 1.5 mL of anhydrous CH_2Cl_2 (0.1 M). The vial was flushed with nitrogen, capped and stirred at 100 °C for 3 h. The solution was cooled to room temperature and 1,3-propanedithiol (30 µL, 0.3 mmol, 2 equiv), chlorotrimethylsilane (6 µL, 0.045 mmol, 0.3 equiv) and Zn(OTf)$_2$ (5.5 mg, 0.015 mmol, 0.1 equiv) were added to the reaction mixture. The solution was stirred at room temperature for 1 h. Solvent was removed under reduced pressure and the residue was purified by column chromatography (neutral Al_2O_3, pentane/Et_2O 9:1) to afford **19** as a white solid (30.6 mg, 70% yield).

M.p. = 83-85 °C; **Rf =** 0.27 (silica gel, pentane/Et_2O, 9:1); **^{1}H NMR (400 MHz, $CDCl_3$):** δ 7.54-7.46 (*m*, 2H), 7.39-7.31 (*m*, 2H), 7.31-7.27 (*m*, 1H), 4.33 (*s*, 1H), 3.78 (*d*, *J* = 8.3 Hz, 1H), 3.59 (*d*, *J* = 8.3 Hz, 1H), 2.84-2.74 (*m*, 4H), 2.62 (*d*, *J* = 12.9 Hz, 1H), 2.27 (*d*, *J* = 12.9 Hz, 1H), 2.06-1.93 (*m*, 1H), 1.87-1.71 (*m*, 1H), 1.19 (*s*, 3H), 0.80 (s, 3H) ppm; **^{13}C NMR (100 MHz, $CDCl_3$):** δ 143.7 (C), 127.8 (CH), 127.5 (CH), 126.5 (CH), 89.1 (C), 80.9 (CH_2), 60.2 (CH), 50.1 (CH_2), 40.2 (C), 31.0 (CH_2), 30.8 (CH_2), 27.6 (CH_3), 27.2 (CH_3), 25.8 (CH_2) ppm; **IR (neat):** 2958, 2865, 1465, 1441, 1277, 1045, 906, 763, 706, 681, 571 cm^{-1}; **HR-MS (ESI):** m/z = 317.0992 [M+Na]$^+$ (calculated for $C_{16}H_{22}NaOS_2$ m/z = 317.1004).

12. Synthesis of amine 20

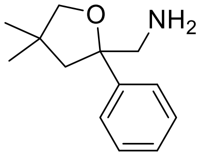

To a stirred solution of compound **9hA** (137.6 mg, 0.35 mmol, 1 equiv) in a mixture of CH_3CN/DMSO (49:1, 14 mL) were added K_2CO_3 (194 mg, 1.4 mmol, 4 equiv) and PhSH (180 µL, 1.75 mmol, 5 equiv). The reaction mixture was stirred at 50 °C for 2 h. After being cooled to 20 °C, solvent was evaporated and the residue was directly purified by column chromatography (silica gel, pentane/EtOAc 7:3) to afford compound **20** as a colorless oil (46.6 mg, 65% yield).

^{1}H NMR (400 MHz, $CDCl_3$): δ 7.43-7.29 (*m*, 4H), 7.25-7.18 (*m*, 1H), 3.62 (*d*, *J* = 8.3 Hz, 1H), 3.58 (*d*, *J* = 8.3 Hz, 1H), 3.02-2.63 (*m*, 2H), 2.06 (*d*, *J* = 12.5 Hz, 1H), 1.96 (*d*, *J* = 12.5 Hz, 1H), 1.14 (*s*, 3H), 0.91 (*s*, 3H) ppm; **^{13}C NMR (100 MHz, $CDCl_3$):** δ 145.9 (C), 128.3 (CH), 126.6 (CH), 125.7 (CH), 79.5 (CH_2), 53.4 (CH_2), 51.4 (CH_2), 40.5 (C), 27.4 (CH_3), 27.2 (CH_3) ppm; **IR (neat):** 2957, 2866, 1448, 1260, 1054, 764, 734, 703 cm^{-1}; **HR-MS (ESI):** m/z = 206.1539 [M+H]$^+$ (calculated for $C_{13}H_{20}NO$ m/z = 206.1539).

13. Synthesis of spiroindoline 7

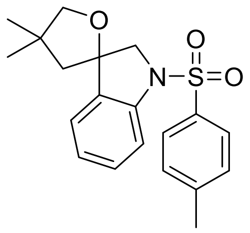

In a 2 mL screw-cap vial equipped with a magnetic stirring bar, 2-aminotetrahydrofuran **9dA** (65.8 mg, 0.15 mmol, 1 equiv) was dissolved in 0.9 mL of dry toluene. $Pd(OAc)_2$ (6.7 mg, 0.03 mmol, 20 mol %), (±)-BINAP (37.4 mg, 0.06 mmol, 40 mol %) and K_2CO_3 (51.8 mg, 0.38 mmol, 2.5 equiv) were added and the mixture was stirred at 115 °C for 12 h. Then, it was cooled to room temperature, quenched with H_2O and extracted with EtOAc (3 x 10 mL). The combined organic fraction was washed with H_2O (3 x 10 mL), brine (20 mL), dried over $MgSO_4$ and concentrated under reduced pressure. The residue was purified by column chromatography (silica gel, pentane/EtOAc, 9:1) to afford compound **7** as a white solid (46.1 mg, 86% yield).

M.p. = 139.5-141.5 °C; **Rf =** 0.52 (silica gel, pentane/EtOAc, 8:2); **^{1}H NMR (400 MHz, $CDCl_3$):** δ 7.70 (*d*, *J* = 8.3 Hz, 2H), 7.66 (*d*, *J* = 8.2 Hz, 1H), 7.29 (*ddd*, *J* = 8.3, 7.5, 1.3 Hz, 1H), 7.25-7.18 (*m*, 3H), 7.05 (*td*, *J* = 7.5, 1.0 Hz, 1H), 4.00 (*d*, *J* = 11.2 Hz, 1H), 3.82 (*d*, *J* = 11.2 Hz, 1H), 3.61 (*d*, *J* = 8.5 Hz, 1H), 3.55 (*d*, *J* = 8.5 Hz, 1H), 2.35 (*s*, 3H), 2.06 (*d*, *J* = 13.4 Hz, 1H), 1.86 (*d*, *J* = 13.4 Hz, 1H), 1.18 (*s*, 3H), 1.17 (*s*, 3H) ppm; **^{13}C NMR (100 MHz, $CDCl_3$):** δ 144.3 (C), 141.7 (C), 135.4 (C), 134.1 (C), 129.9 (CH), 129.8 (CH), 127.5 (CH), 124.2 (CH), 123.9 (CH), 114.7 (CH), 86.9 (C), 80.6 (CH_2), 63.0 (CH_2), 52.7 (CH_2), 40.3 (C), 26.60 (CH_3), 26.59 (CH_3), 21.7 (CH_3); **IR (neat):** 2969, 2840, 1598, 1459, 1343, 1162, 1117, 1096, 1051, 1034, 1015, 948, 759, 660 cm^{-1}; **HR-MS (ESI):** m/z = 358.1459 $[M+H]^+$ (calculated for $C_{20}H_{24}NO_3S$ m/z = 358.1471).

14. Synthesis of spirotetrahydroisoquinoline 8

In a 2 mL screw-cap vial equipped with a magnetic stirring bar, 2-aminotetrahydrofuran **9** (0.15 mmol, 1 equiv) and paraformaldehyde ($(HCHO)_n$, 9.5 mg, 0.315 mmol, 2.1 equiv) were dissolved in 0.7 mL of anhydrous 1,2-DCE. Trifluoroacetic anhydride (TFAA, 64 µL, 0.45 mmol, 3 equiv) was added and the solution was cooled to 0 °C. MsOH (97 µL, 1.5 mmol, 10 equiv) was slowly added and the solution was stirred at 0 °C for 25 min. Then, 4 mL of H_2O was added and the mixture was stirred for an additional 5 min at 0 °C and 10 min at room temperature. After that, 15 mL of $CHCl_3$ and 20 mL of H_2O were added to the reaction mixture. The layers were separated and the aqueous fraction was extracted with $CHCl_3$ (3 x 15 mL). The organic fraction was washed with 50 mL of sat. $NaHCO_3$ solution, 50 mL of brine, dried over Na_2SO_4 and concentrated under vacuum. The residue was purified by column chromatography

Compound 8A:

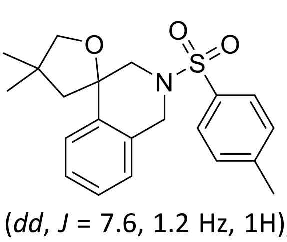

Compound **8A** is obtained as a white solid (49.2 mg, 88% yield) starting from 2-aminotetrahydrofuran **9aA** (53.9 mg, 0.15 mmol).
Purification: column chromatography (silica gel, pentane/Et_2O, 8:2)
M.p. = 107-108 °C; **Rf =** 0.38 (silica gel, pentane/Et_2O, 7:3); **^{1}H NMR (400 MHz, $CDCl_3$):** δ 7.73 (*d*, *J* = 8.3 Hz, 2H), 7.52 (*dd*, *J* = 7.8, 1.3 Hz, 1H), 7.34 (*d*, *J* = 8.0 Hz, 2H), 7.23 (*dd*, *J* = 7.7, 1.3 Hz, 1H), 7.17 (*td*, *J* = 7.5, 1.4 Hz, 1H), 6.99 (*dd*, *J* = 7.6, 1.2 Hz, 1H), 4.59 (*d*, *J* = 14.8 Hz, 1H), 3.95-3.87 (*m*, 2H), 3.86-3.77 (*m*, 2H), 2.62 (*dd*, *J* = 11.0, 1.3 Hz, 1H), 2.47-2.39 (*m*, 4H), 1.83 (*dd*, *J* = 13.5, 1.4 Hz, 1H), 1.27 (*s*, 3H), 1.24 (*s*, 3H) ppm; **^{13}C NMR (100 MHz, $CDCl_3$):** δ 144.0 (C), 141.2 (C), 133.4 (C), 130.7 (C), 129.9 (CH), 127.8 (CH), 127.6 (CH), 127.3 (CH), 125.8 (CH), 125.4 (CH), 82.6 (C), 81.6 (CH_2), 53.4 (CH_2), 52.2 (CH_2), 47.6 (CH_2), 40.6 (C), 28.8 (CH_3), 27.6 (CH_3), 21.7 (CH_3) ppm; **IR (neat):** 2955, 2845, 1336, 1166, 1054, 971, 815, 763, 657 cm^{-1}; **HR-MS (ESI):** m/z = 372.1629 $[M+H]^+$ (calculated for $C_{21}H_{26}NO_3S$ m/z = 372.1628).

Compound 8B:

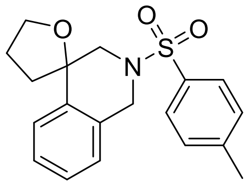

Compound **8B** is obtained as a white solid (33.3 mg, 65% yield) starting from 2-aminotetrahydrofuran **9aB** (49.7 mg, 0.15 mmol).

Purification: column chromatography (silica gel, pentane/EtOAc, 8:2)

M.p. = 156-158 °C; **Rf =** 0.48 (silica gel, pentane/EtOAc, 7:3); **^{1}H NMR (400 MHz, $CDCl_3$):** δ 7.73 (*d*, *J* = 8.3 Hz, 2H), 7.41 (*dd*, *J* = 7.7, 1.4 Hz, 1H), 7.35 (*d*, *J* = 8.1 Hz, 2H), 7.23 (*t*, *J* = 7.6 Hz, 1H), 7.17 (*td*, *J* = 7.4, 1.5 Hz, 1H), 6.99 (*dd*, *J* = 7.6, 1.3 Hz, 1H), 4.63 (*d*, *J* = 14.8 Hz, 1H), 4.22-4.06 (*m*, 2H), 3.82-3.69 (*m*, 2H), 2.60-2.49 (*m*, 2H), 2.43 (*s*, 3H), 2.21-2.10 (*m*, 2H), 1.97-1.86 (*m*, 1H) ppm; **^{13}C NMR (100 MHz, $CDCl_3$):** δ 144.0 (C), 140.7 (C), 133.2 (C), 131.0 (C), 130.0 (CH), 127.8 (CH), 127.5 (CH), 127.4 (CH), 125.7 (CH), 125.6 (CH), 81.6 (C), 70.1 (CH_2), 51.9 (CH_2), 47.9 (CH_2), 39.4 (CH_2), 26.1 (CH_2), 21.7 (CH_3) ppm; **IR (neat):** 2925, 2858, 1333, 1164, 1051, 962, 810, 762, 657 cm^{-1}; **HR-MS (ESI):** m/z = 344.1321 $[M+H]^+$ (calculated for $C_{19}H_{22}NO_3S$ m/z = 344.1315).

Compound 8C:

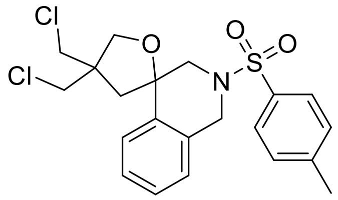

Compound **8C** is obtained as a white solid (59.7 mg, 90% yield) starting from 2-iminotetrahydrofuran **9aC** (64.3 mg, 0.15 mmol).

Purification: column chromatography (silica gel, pentane/Et_2O, 8:2)

M.p. = 57-59 °C; **Rf =** 0.28 (silica gel, pentane/Et_2O, 7:3); **^{1}H NMR (400 MHz, $CDCl_3$):** δ 7.74 (*d*, *J* = 8.3 Hz, 2H), 7.48 (*dd*, *J* = 7.7, 1.4 Hz, 1H), 7.37 (*d*, *J* = 8.0 Hz, 2H), 7.31-7.26 (*m*, 1H), 7.22 (*td*, *J* = 7.5, 1.5 Hz, 1H), 7.02 (*dd*, *J* = 7.6, 1.3 Hz, 1H), 4.63 (*d*, *J* = 14.9 Hz, 1H), 4.14 (*d*, *J* = 10.2 Hz, 1H), 4.04-3.93 (*m*, 2H), 3.92-3.75 (*m*, 5H), 2.63 (*dd*, *J* = 11.2, 1.4 Hz, 1H), 2.56 (*d*, *J* = 14.6 Hz, 1H), 2.44 (*s*, 3H), 2.06 (*dd*, *J* = 14.6, 1.5 Hz, 1H) ppm; **^{13}C NMR (100 MHz, $CDCl_3$):** 144.3 (C), 138.8 (C), 133.3 (C), 131.0 (C), 130.1 (CH), 128.0 (CH), 127.9 (CH), 127.8 (CH), 126.0 (CH), 125.0 (CH), 82.5 (C), 74.4 (CH_2), 51.8 (C), 51.1 (CH_2), 49.1 (CH_2), 48.2 (CH_2), 47.6 (CH_2), 46.8 (CH_2), 21.7 (CH_3) ppm; **IR (neat):** 2864, 1333, 1161, 1045, 1018, 811, 735, 657 cm^{-1}; **HR-MS (ESI):** m/z = 440.0858 $[M+H]^+$ (calculated for $C_{21}H_{24}Cl_2NO_3S$ m/z = 440.0849).

15. NMR spectra of new compounds

Compound 1p: ^{1}H NMR ($CDCl_3$, 500 MHz)

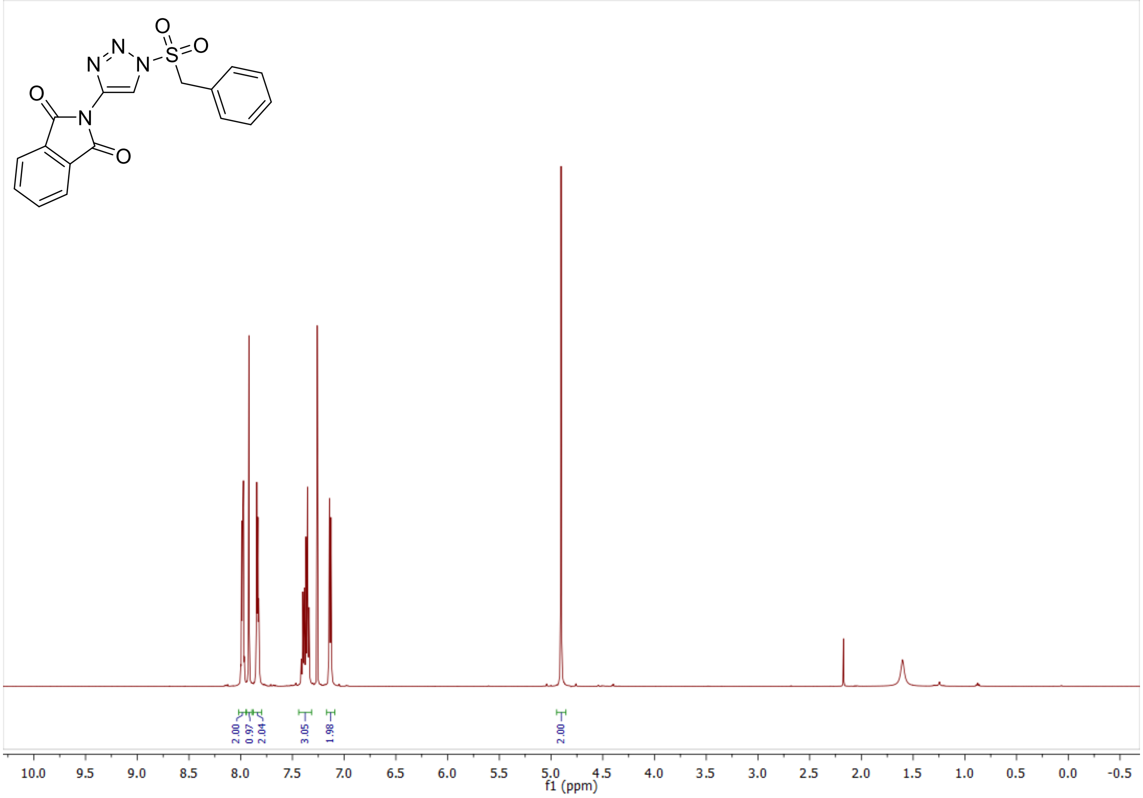

Compound 1p: ^{13}C NMR ($CDCl_3$, 100 MHz)

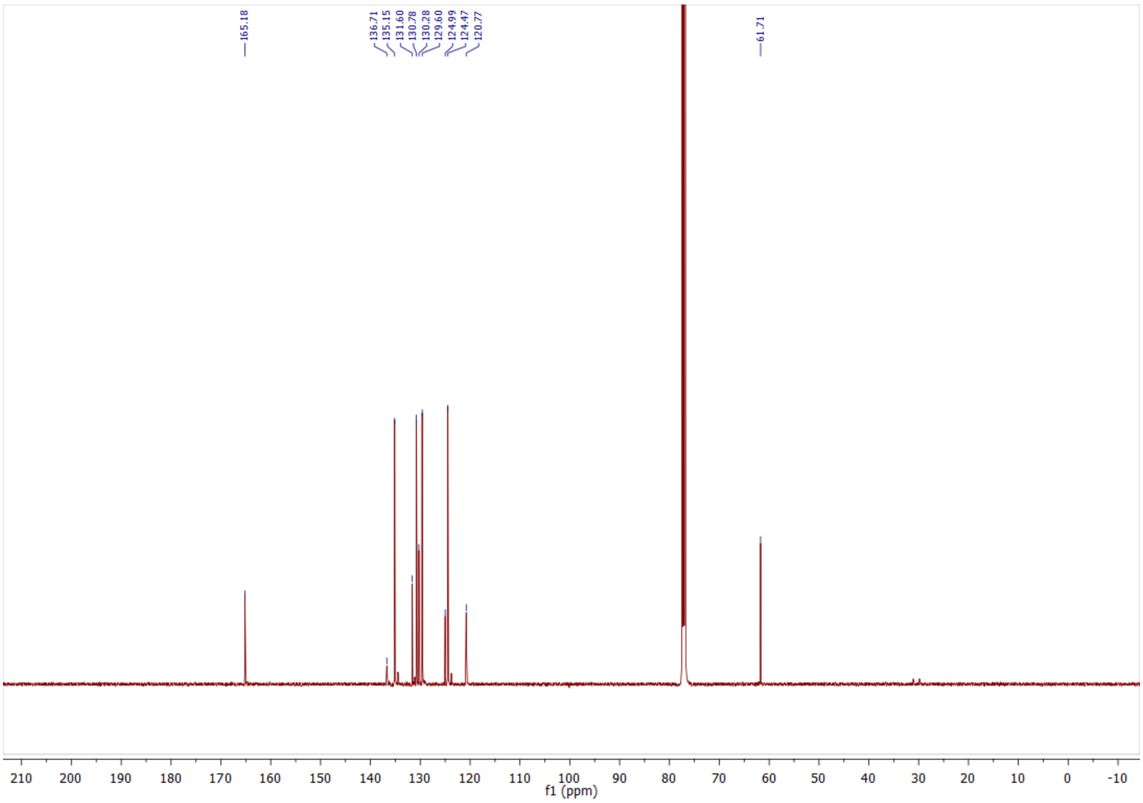

Compound 1q, ^{1}H NMR ($CDCl_3$, 500 MHz)

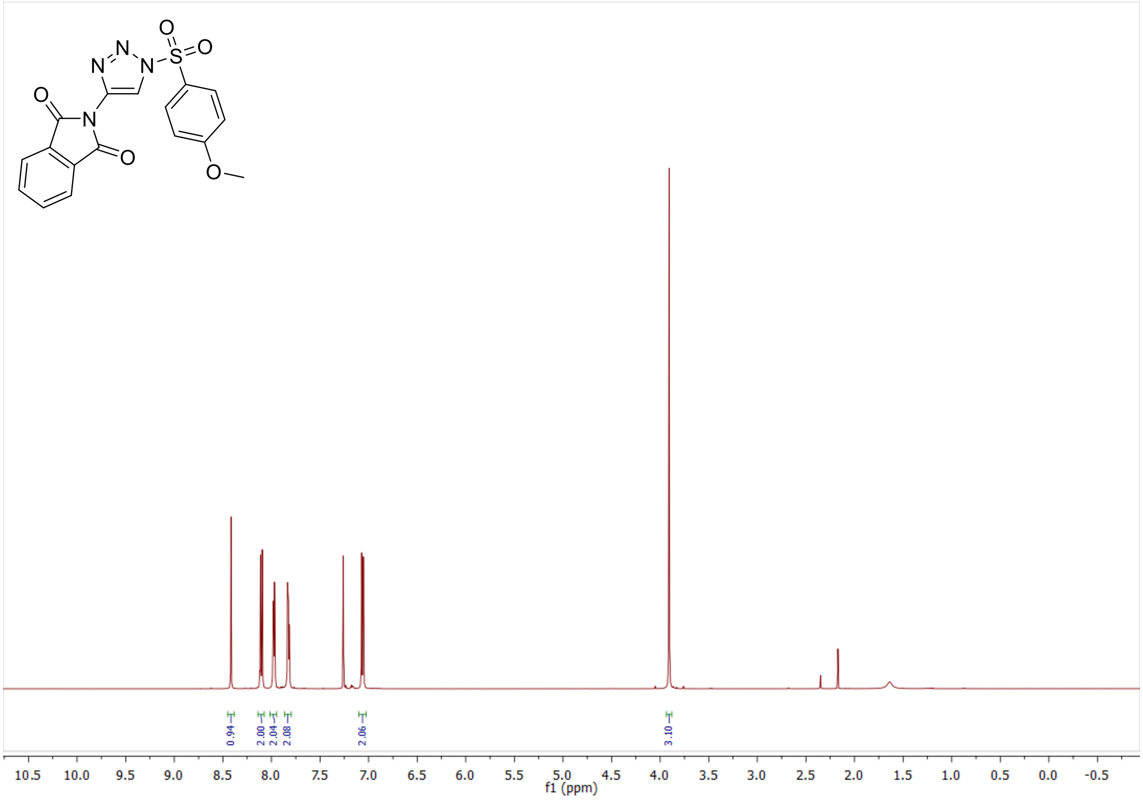

Compound 1q, ^{13}C NMR ($CDCl_3$, 100 MHz)

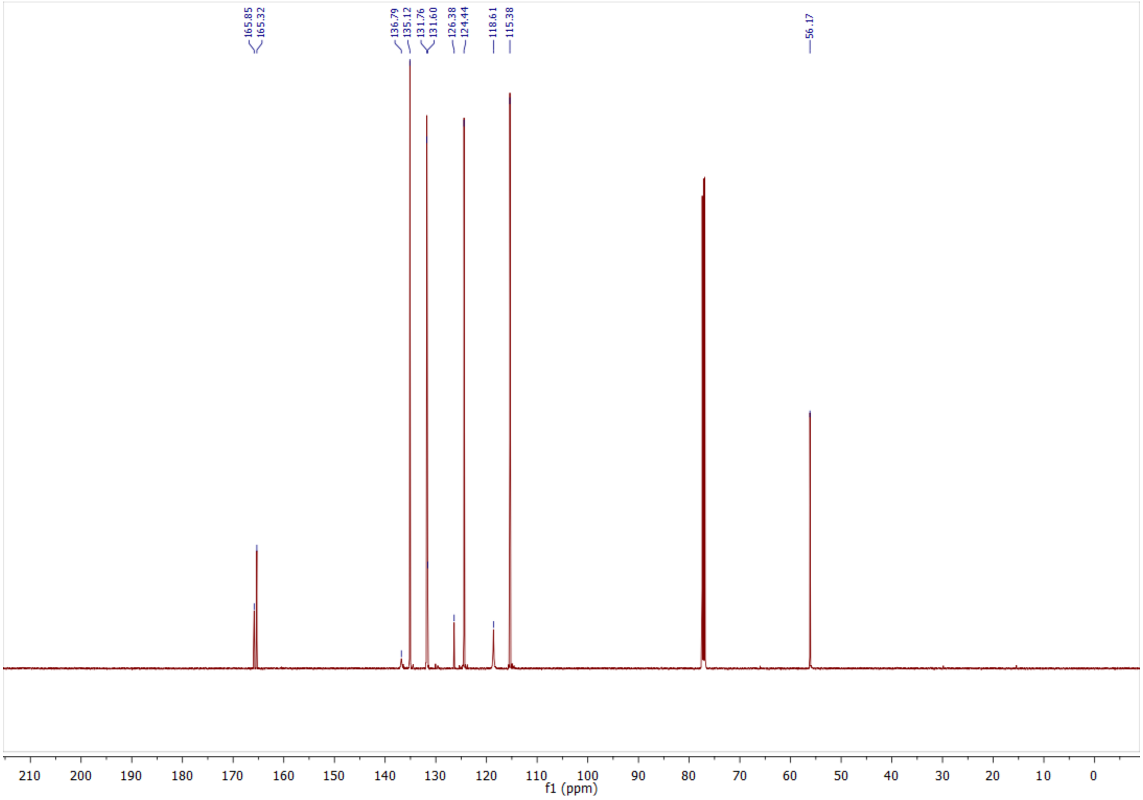

Compound 9aA: ^{1}H NMR ($CDCl_3$, 400 MHz)

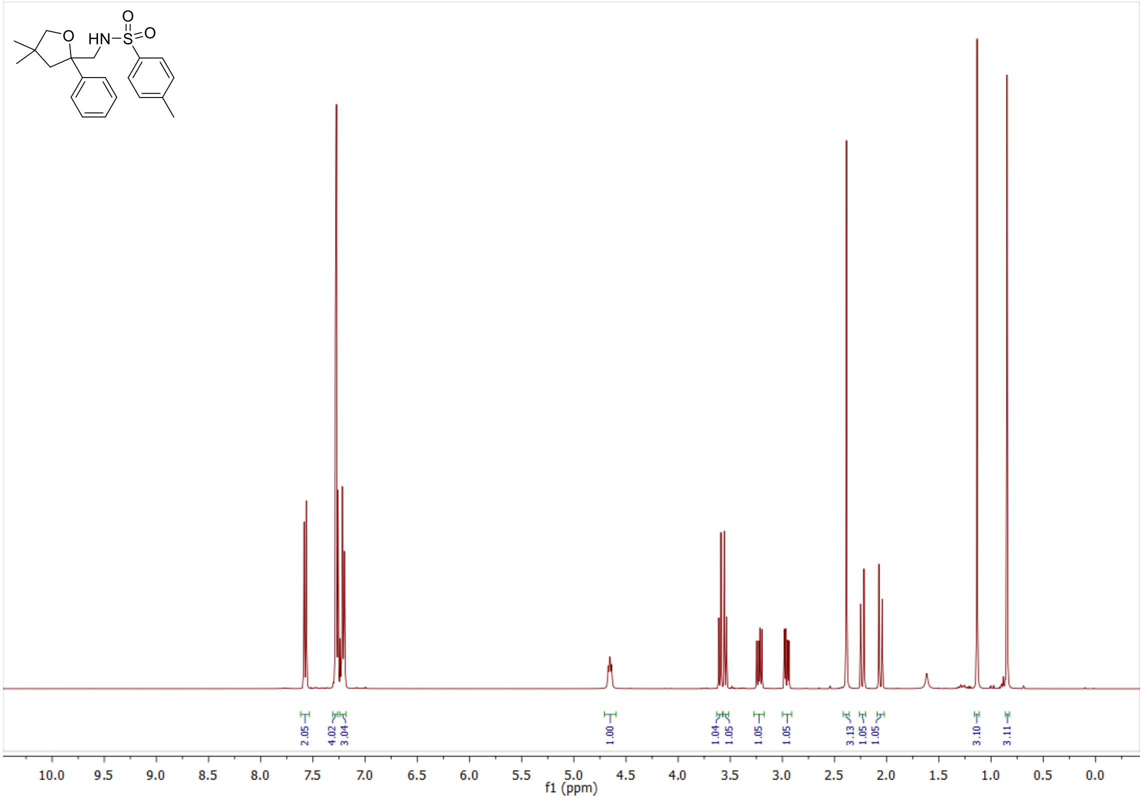

Compound 9aA: ^{13}C NMR ($CDCl_3$, 100 MHz)

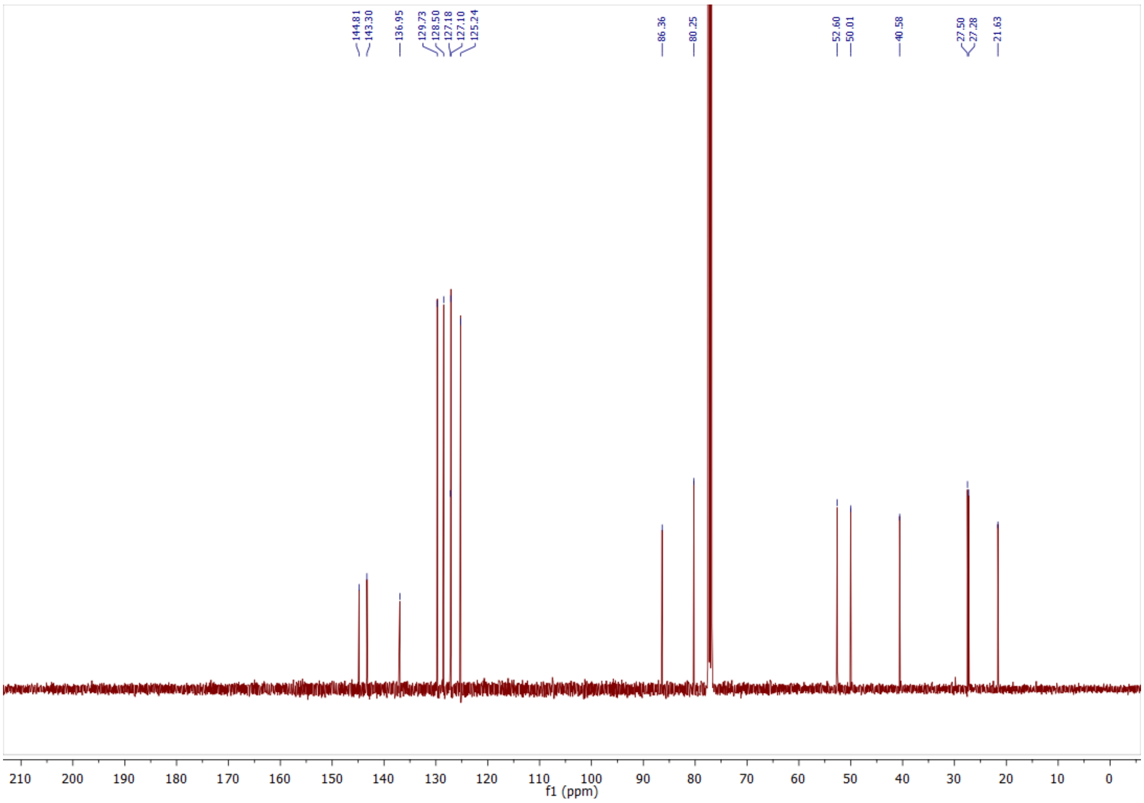

Compound 9bA: ^{1}H NMR ($CDCl_3$, 400 MHz)

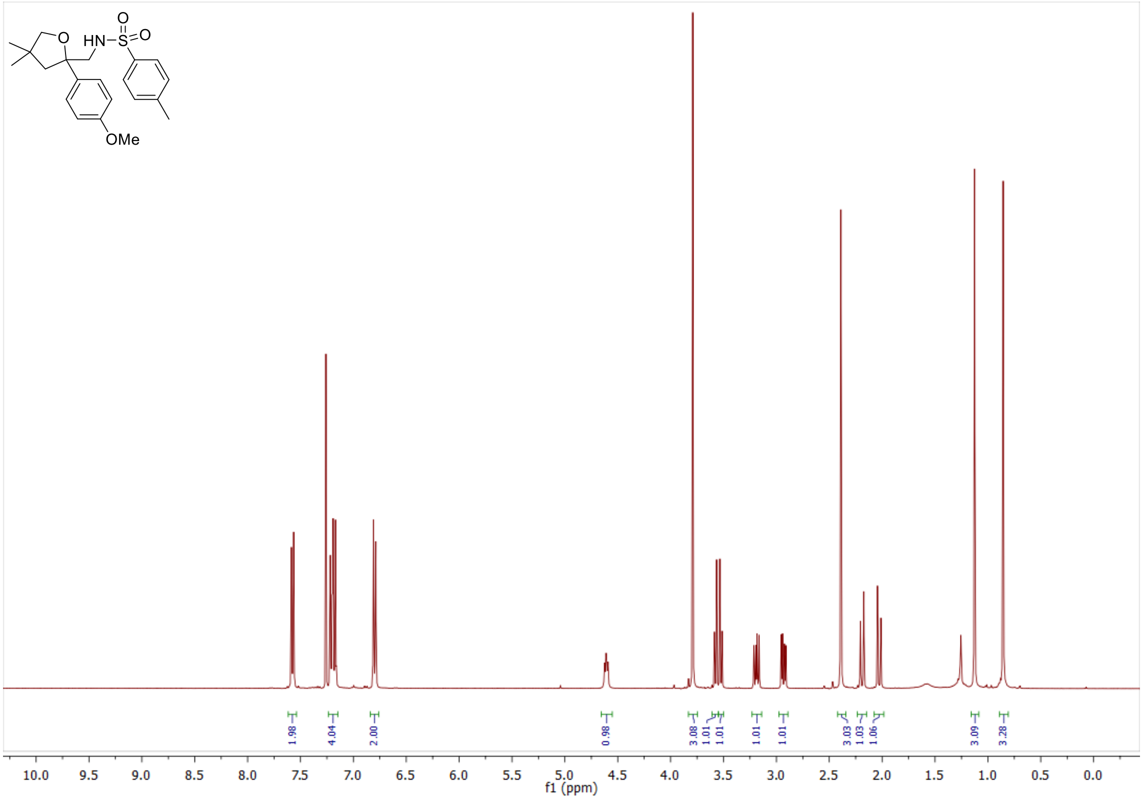

Compound 9bA: ^{13}C NMR ($CDCl_3$, 400 MHz)

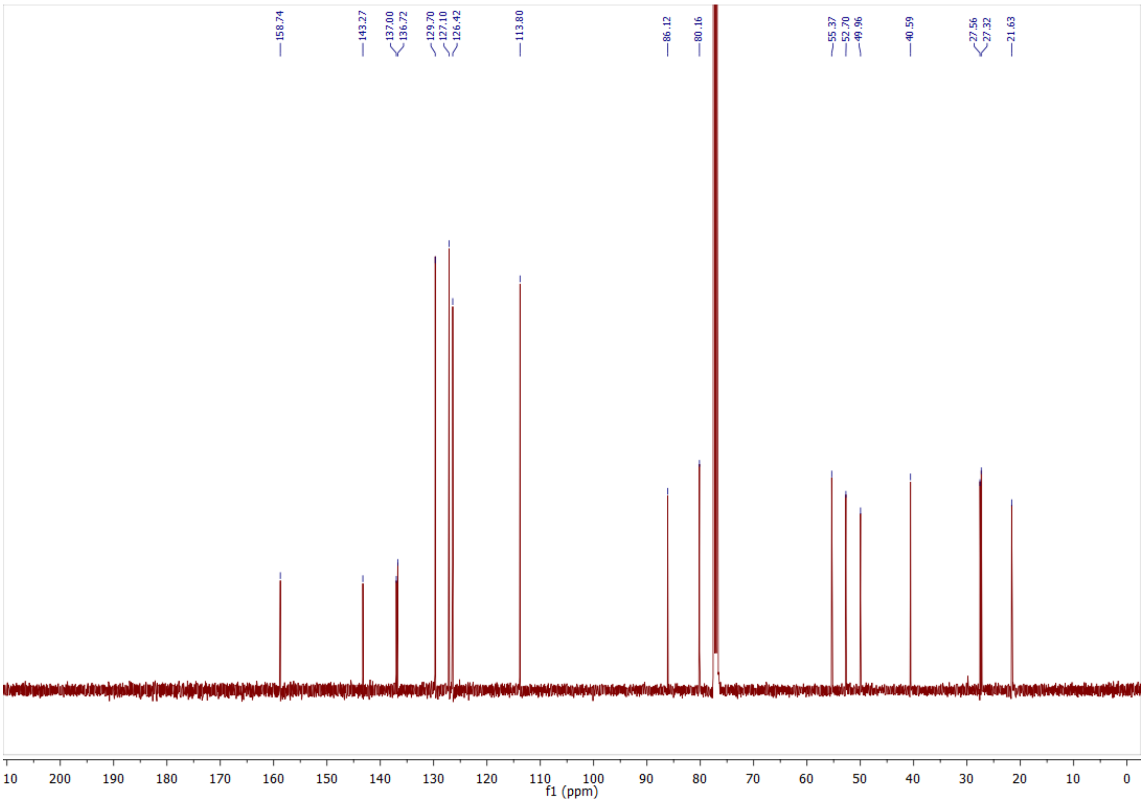

Compound 9cA: ^{1}H NMR ($CDCl_3$, 400 MHz)

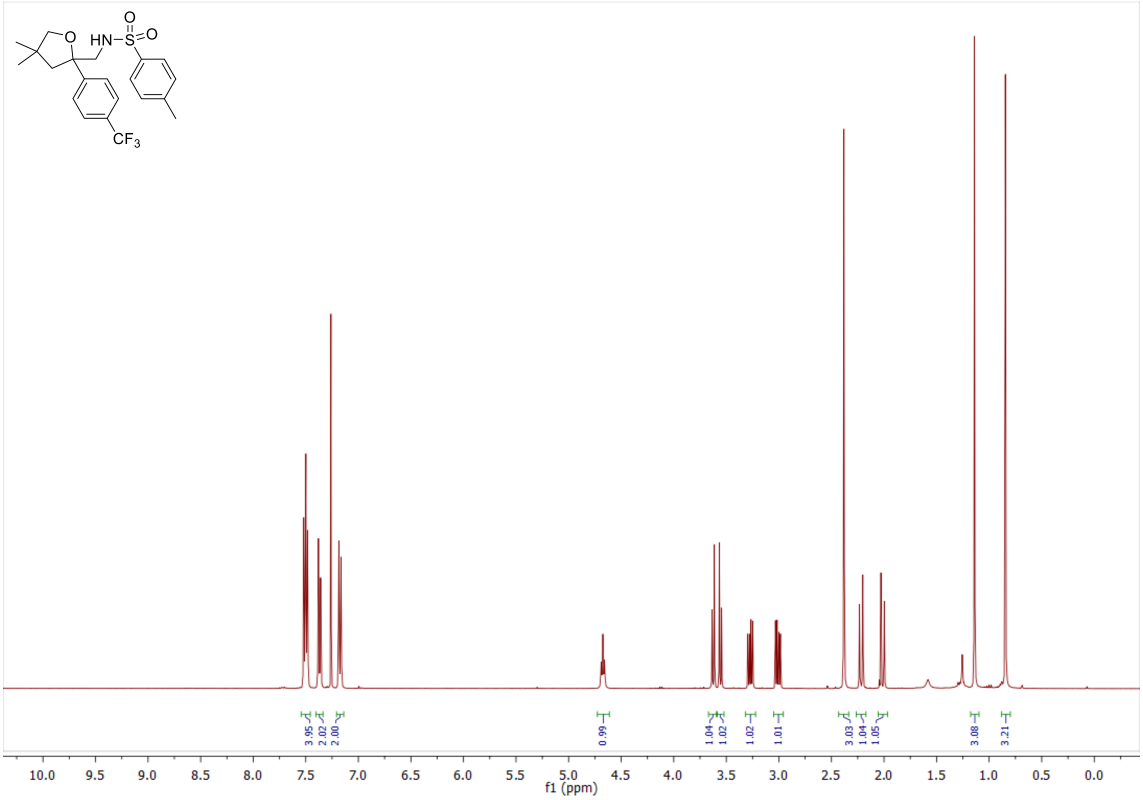

Compound 9cA: ^{13}C NMR ($CDCl_3$, 400 MHz)

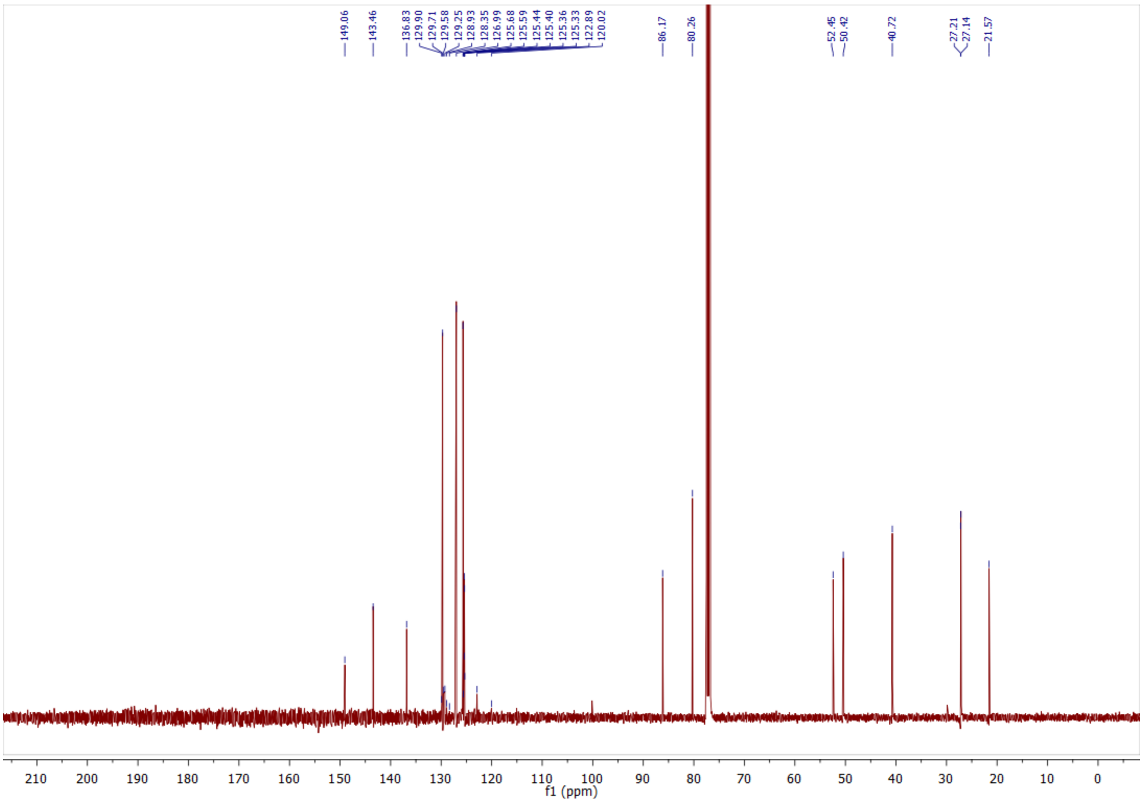

Compound 9dA: ^{1}H NMR ($CDCl_3$, 400 MHz)

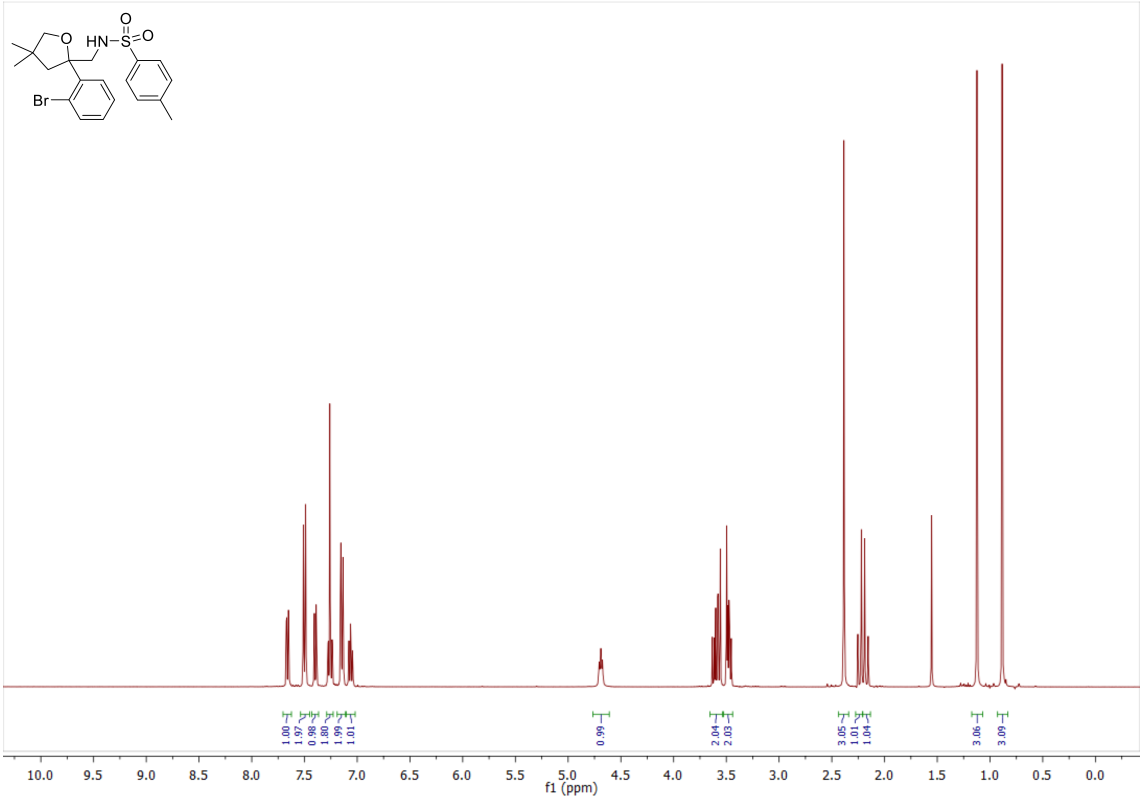

Compound 9dA: ^{13}C NMR ($CDCl_3$, 100 MHz)

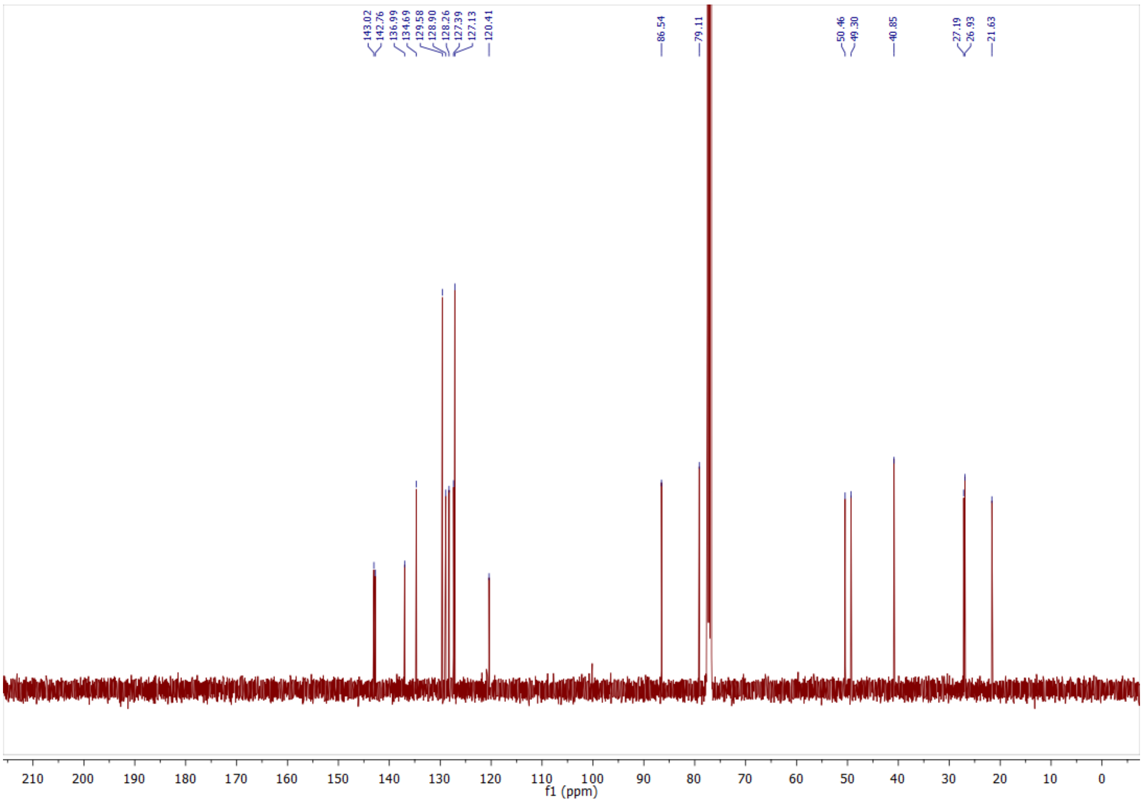

Compound 9eA: ^{1}H NMR ($CDCl_3$, 400 MHz)

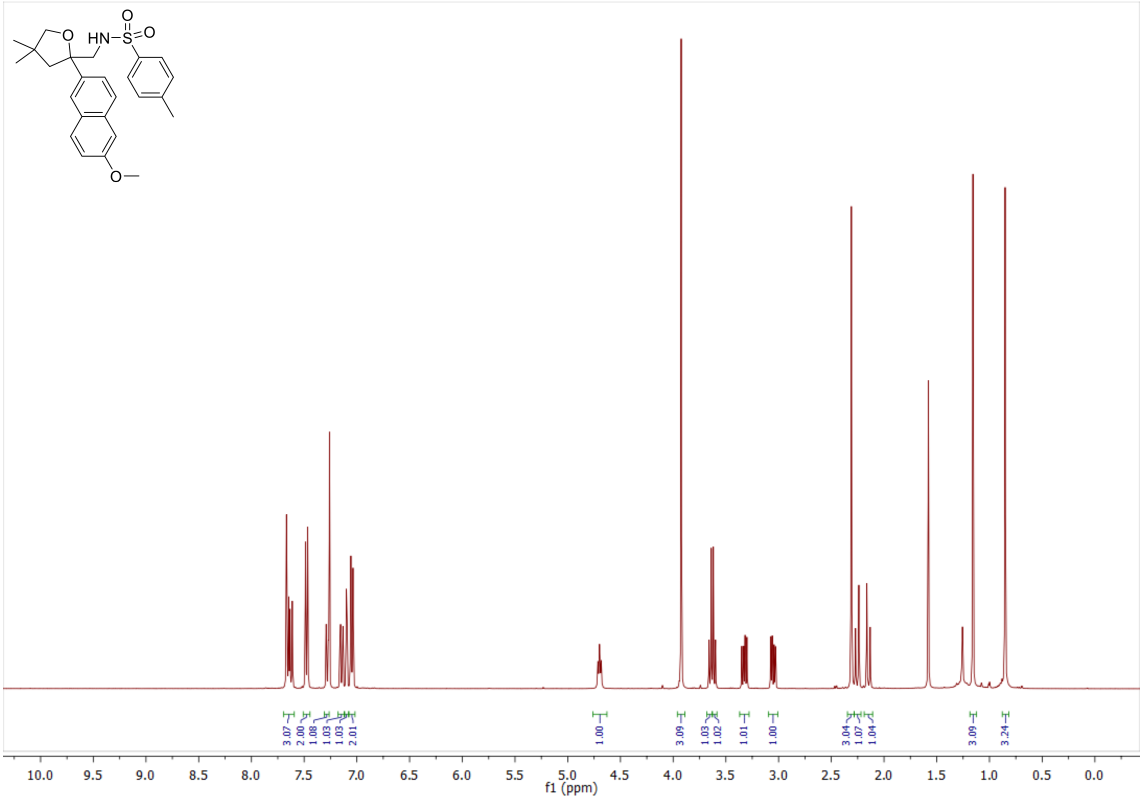

Compound 9eA: ^{13}C NMR ($CDCl_3$, 100 MHz)

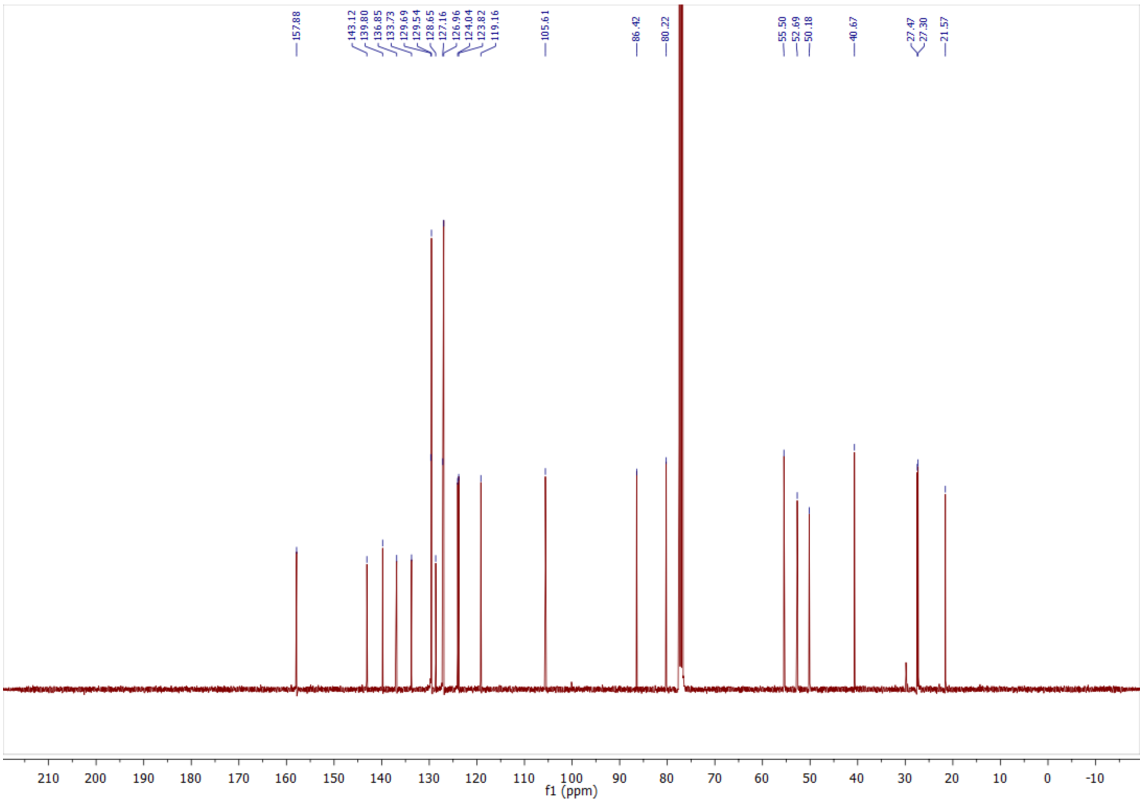

Compound 9fA: ^{1}H NMR ($CDCl_3$, 400 MHz)

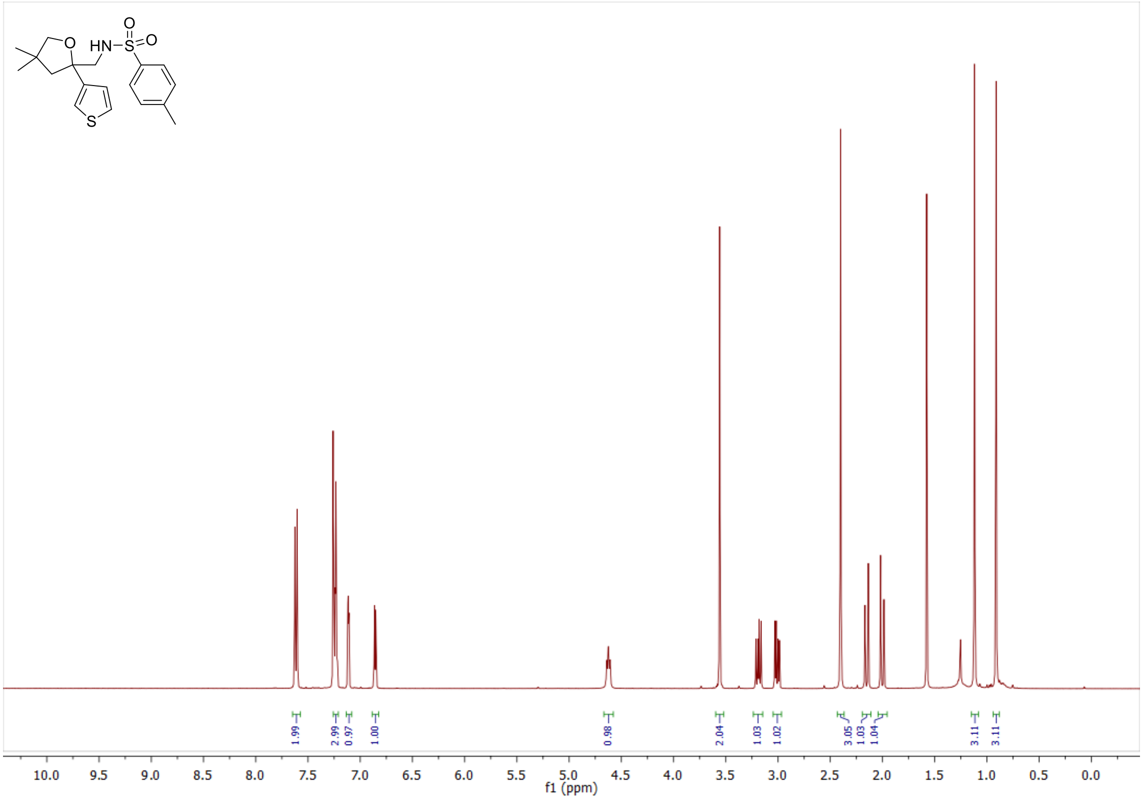

Compound 9fA: ^{13}C NMR ($CDCl_3$, 100 MHz)

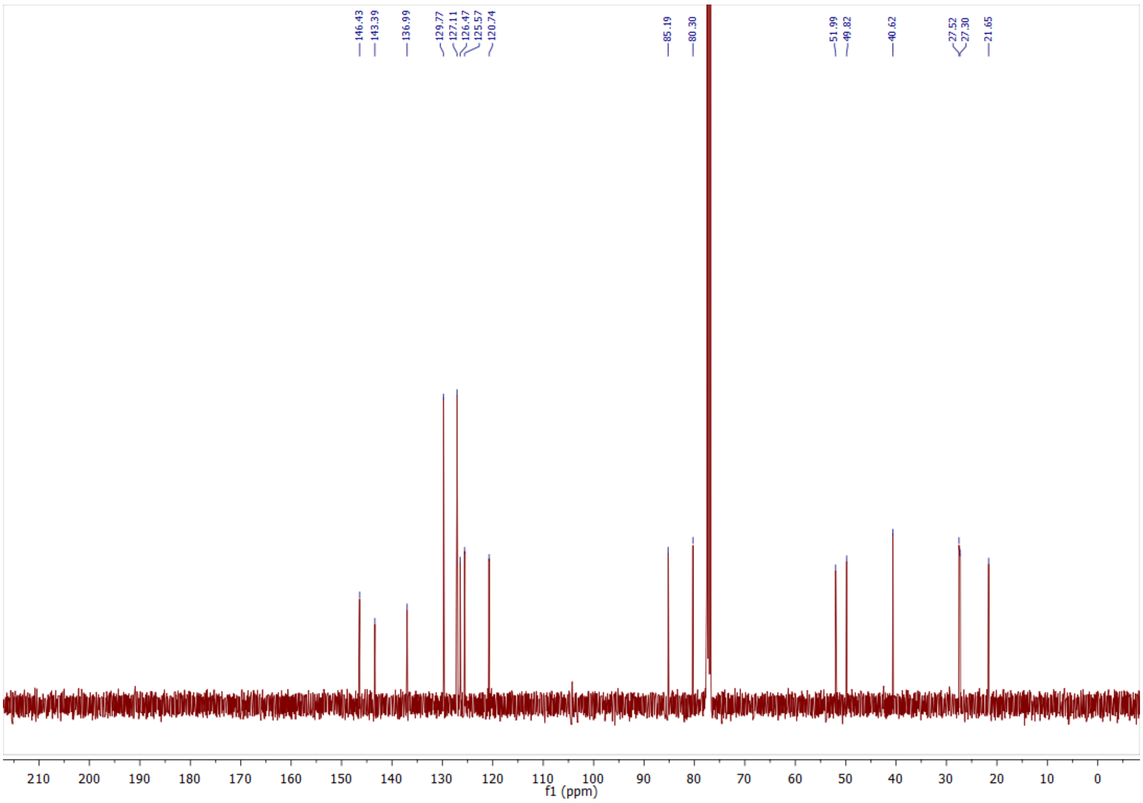

Compound 9gA: ^{1}H NMR ($CDCl_3$, 400 MHz)

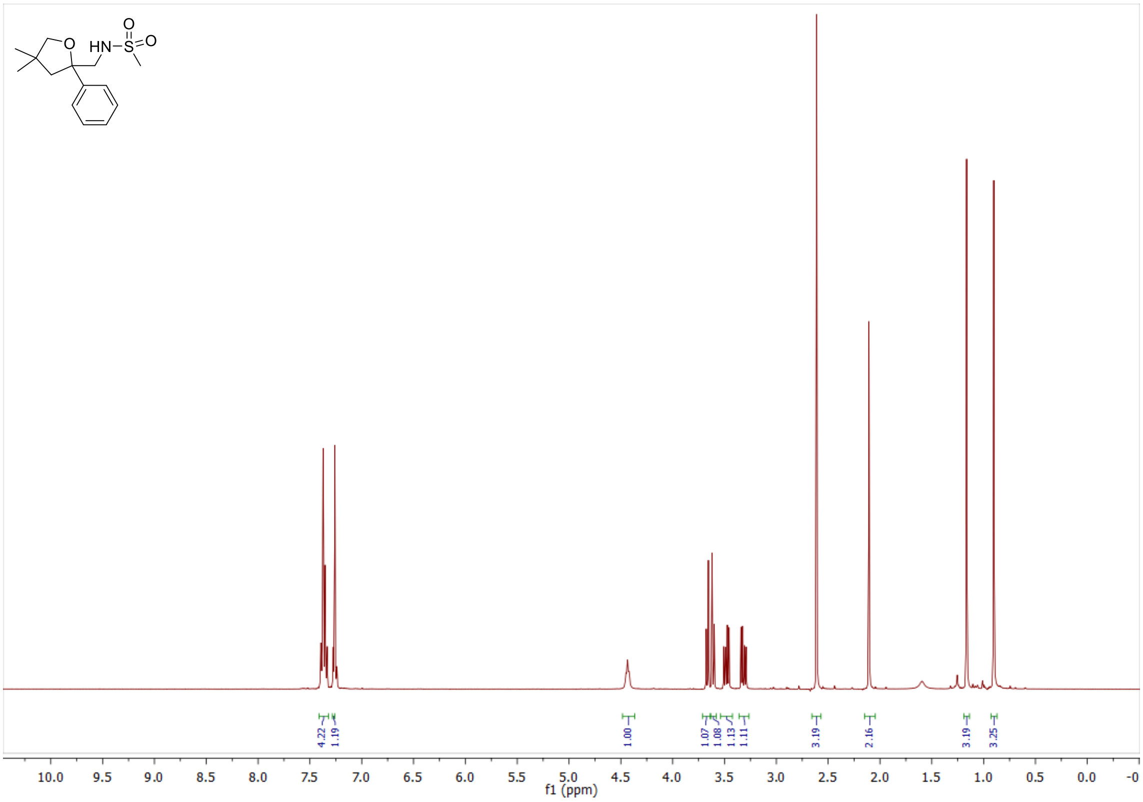

Compound 9gA: ^{13}C NMR ($CDCl_3$, 400 MHz)

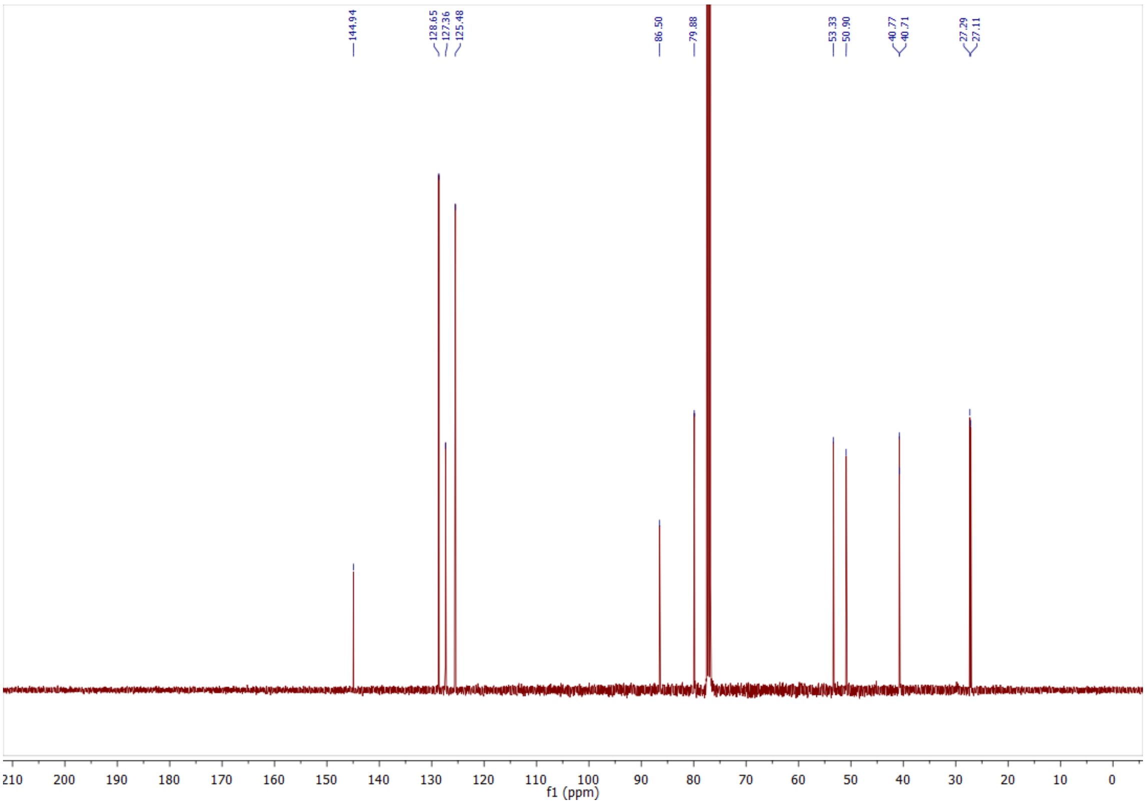

Compound 9hA: ^{1}H NMR ($CDCl_3$, 400 MHz)

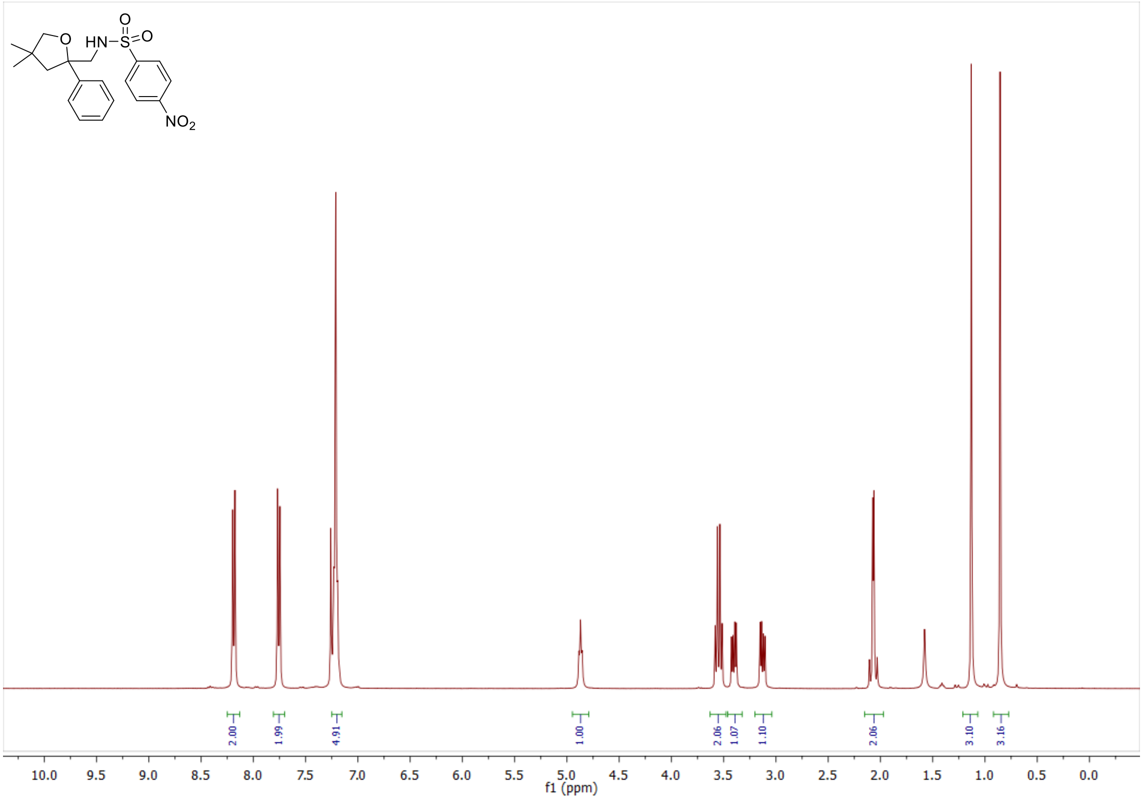

Compound 9hA: ^{1}H NMR ($CDCl_3$, 400 MHz)

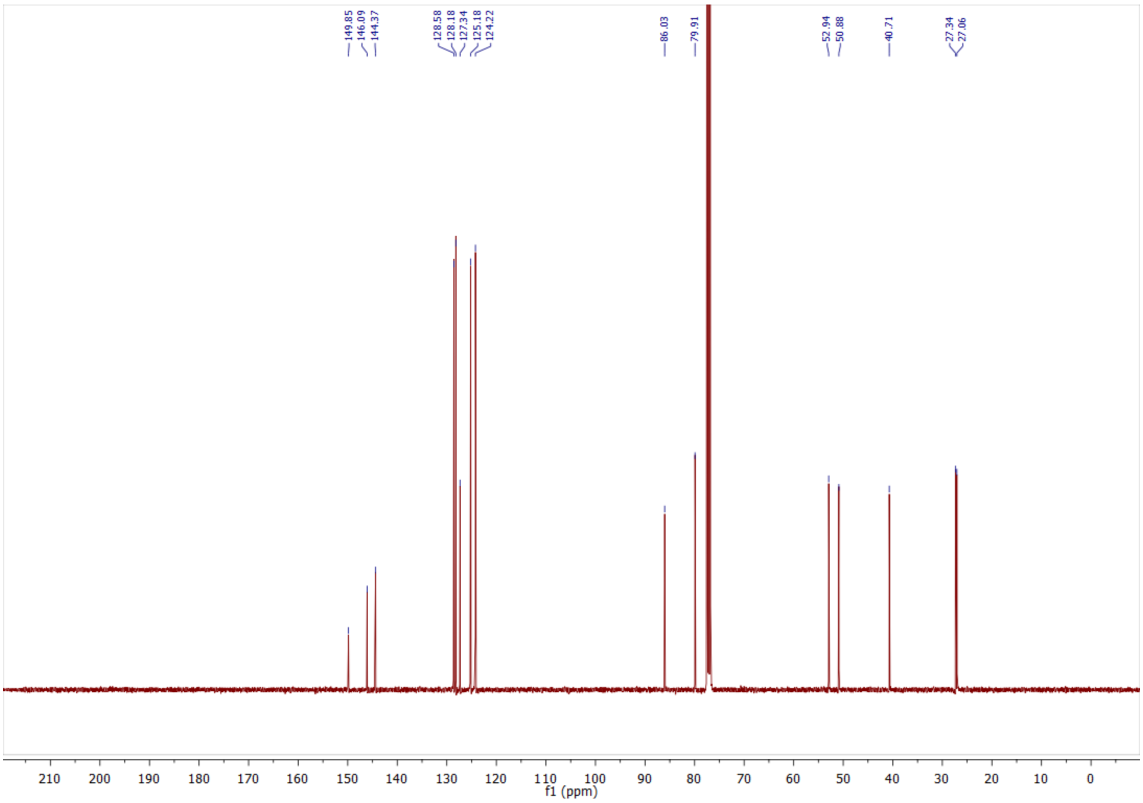

Compound 9aB: ^{1}H NMR ($CDCl_3$, 400 MHz)

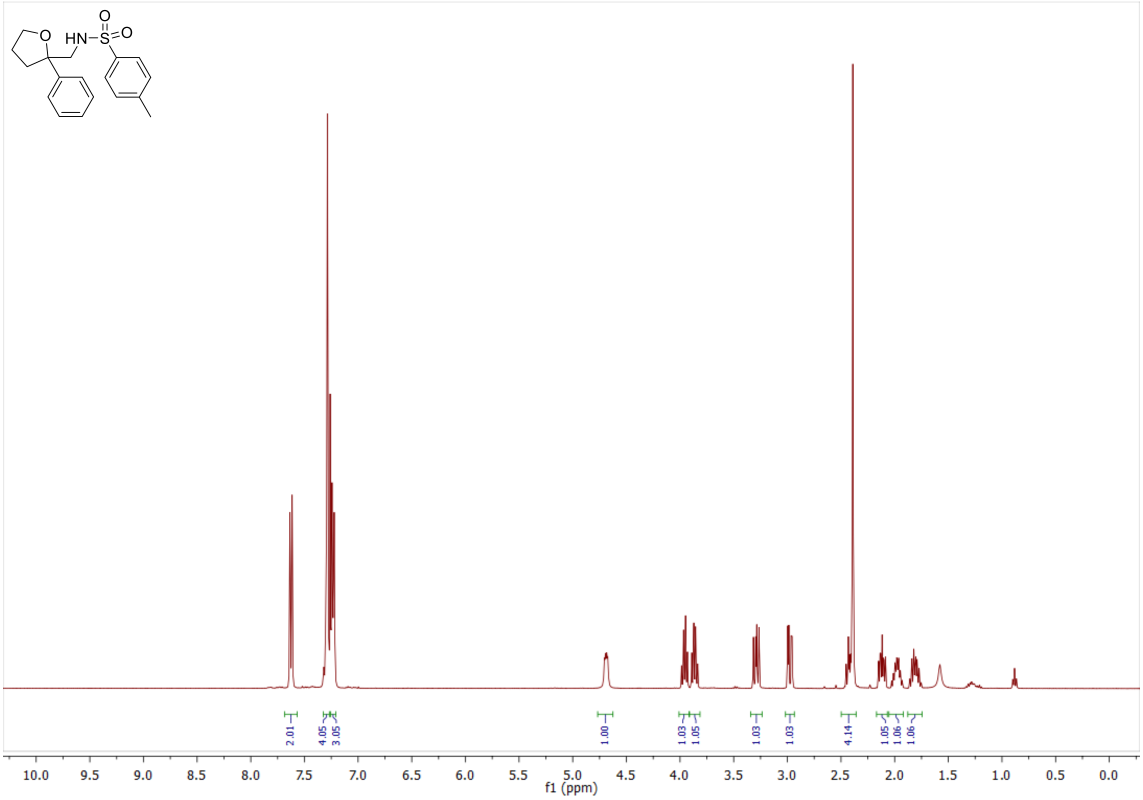

Compound 9aB: ^{13}C NMR ($CDCl_3$, 100 MHz)

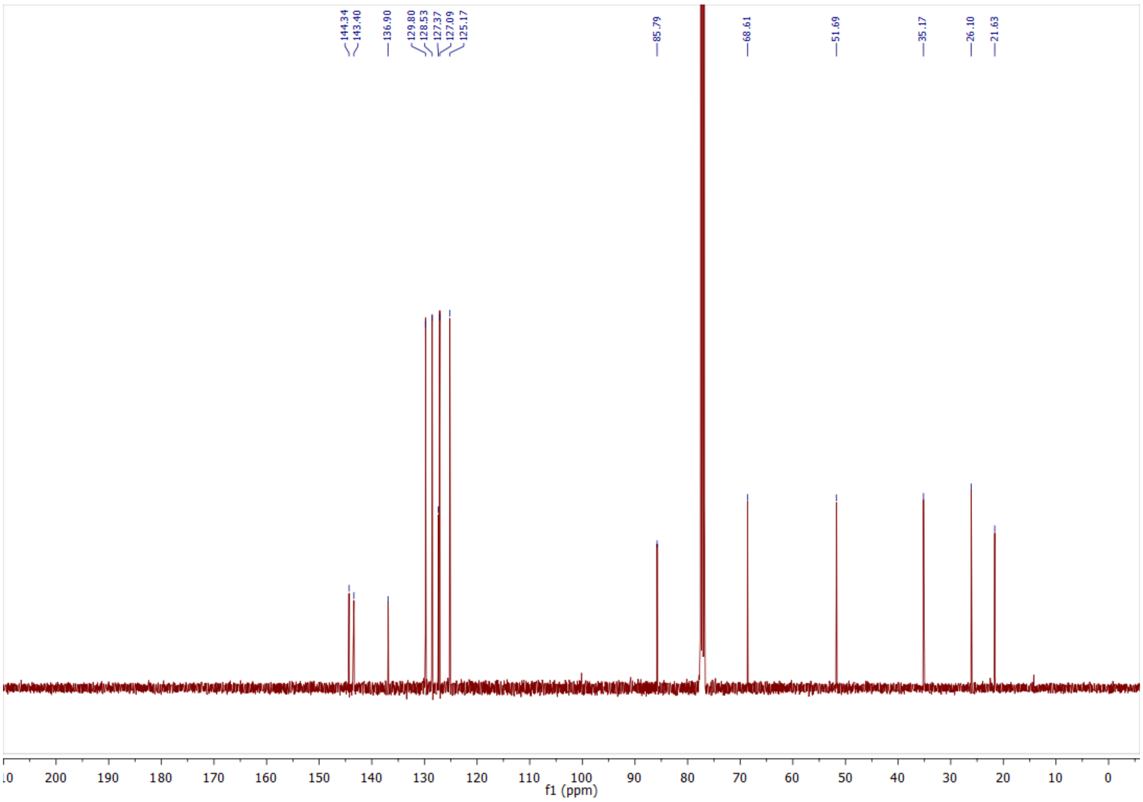

Compound 9aC: ^{1}H NMR ($CDCl_3$, 400 MHz)

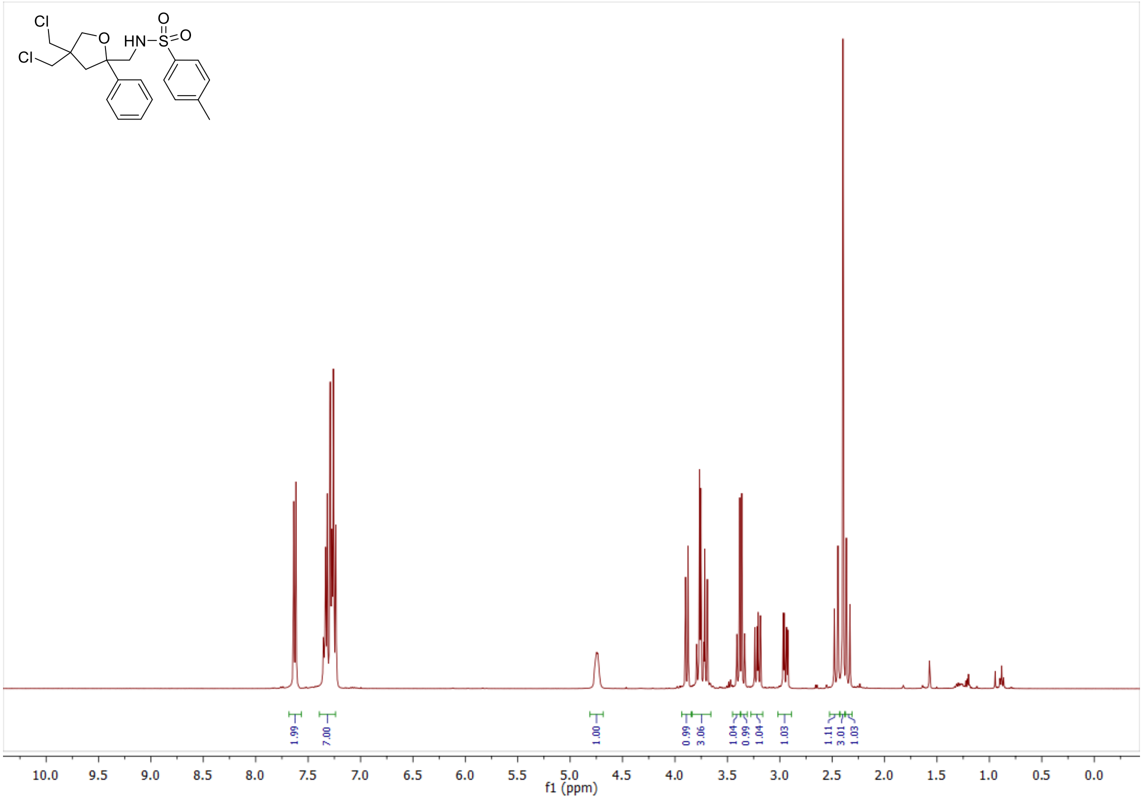

Compound 9aC: ^{13}C NMR ($CDCl_3$, 100 MHz)

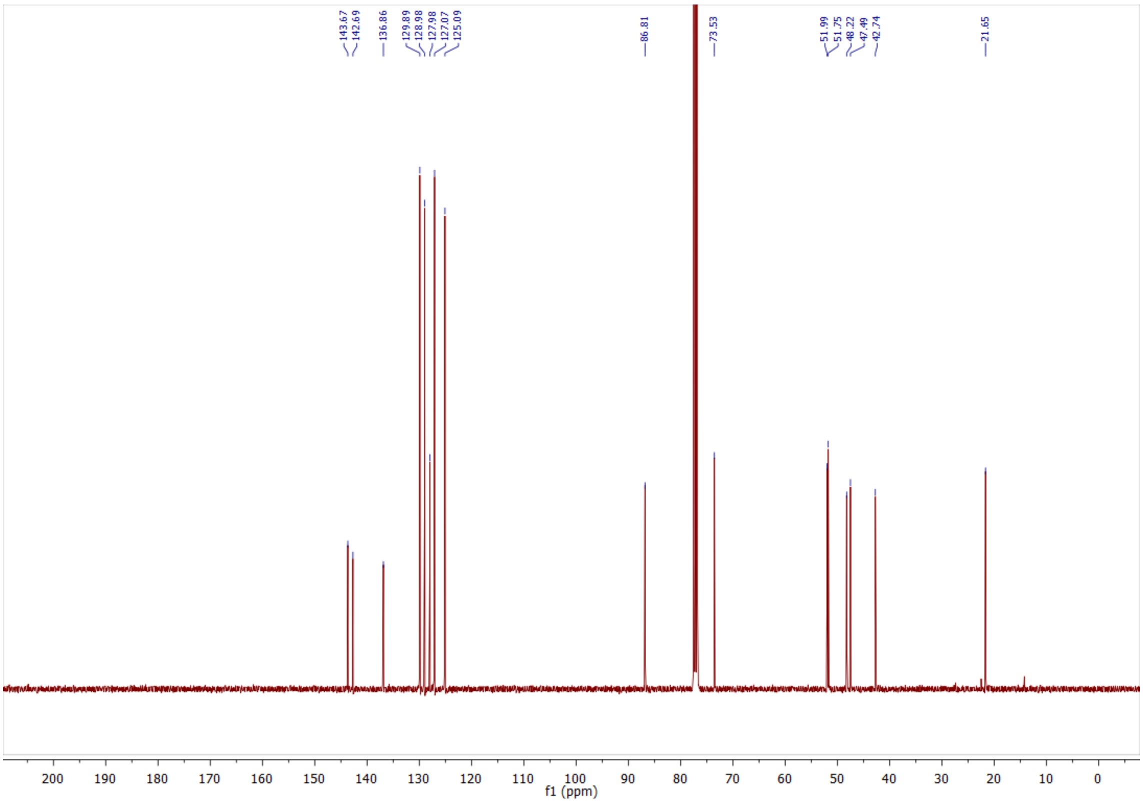

Compound 9aD: ^{1}H NMR ($CDCl_3$, 400 MHz)

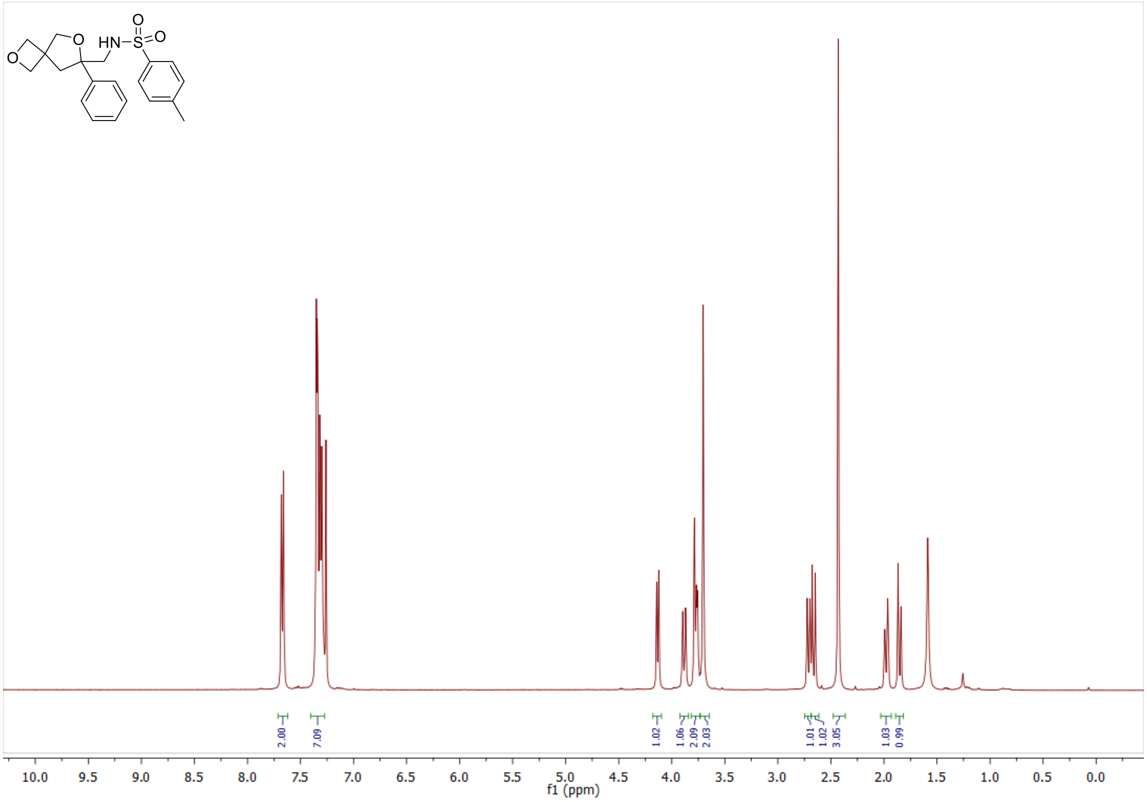

Compound 9aD: ^{13}C NMR ($CDCl_3$, 100 MHz)

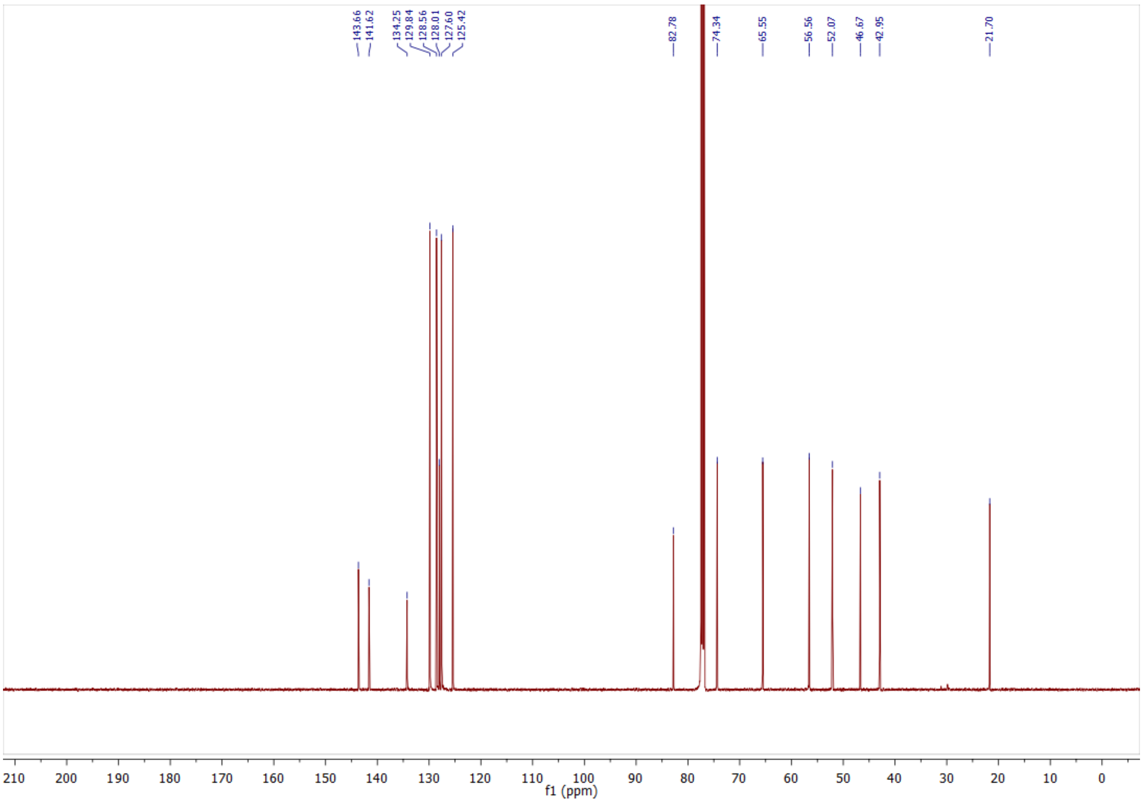

Compound 9iA: ¹H NMR ($CDCl_3$, 400 MHz)

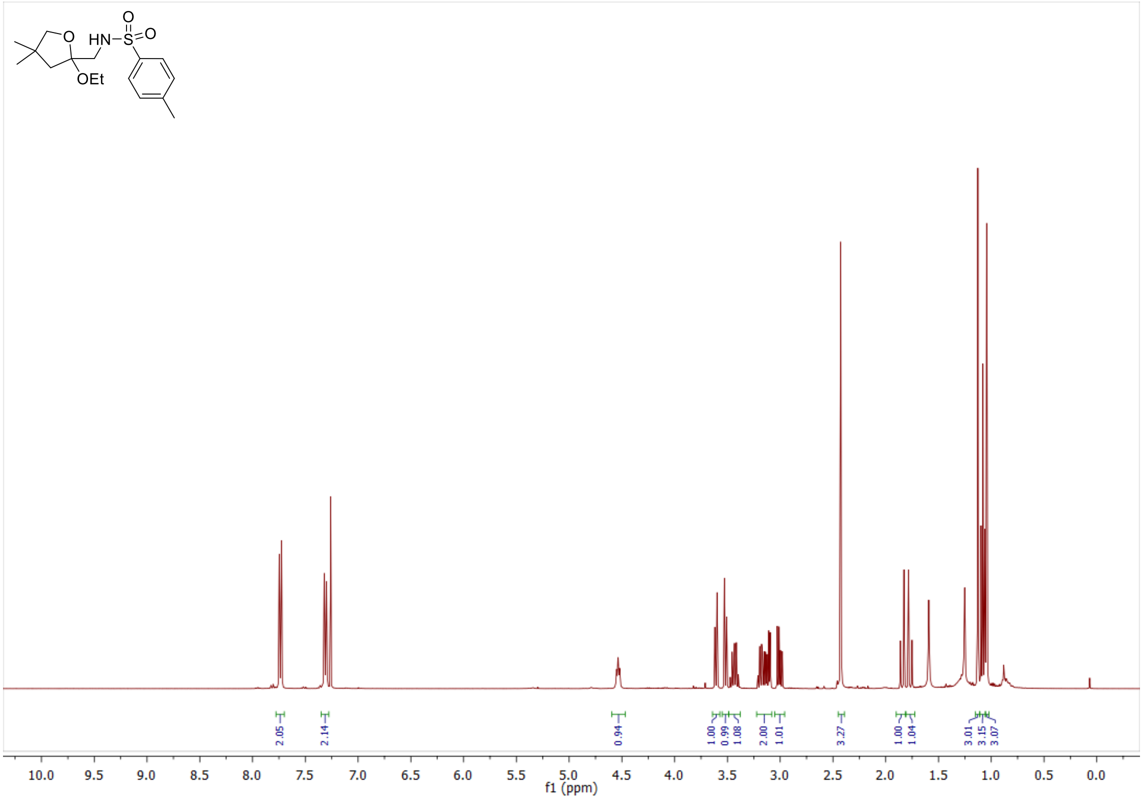

Compound 9iA: ¹³C NMR ($CDCl_3$, 400 MHz)

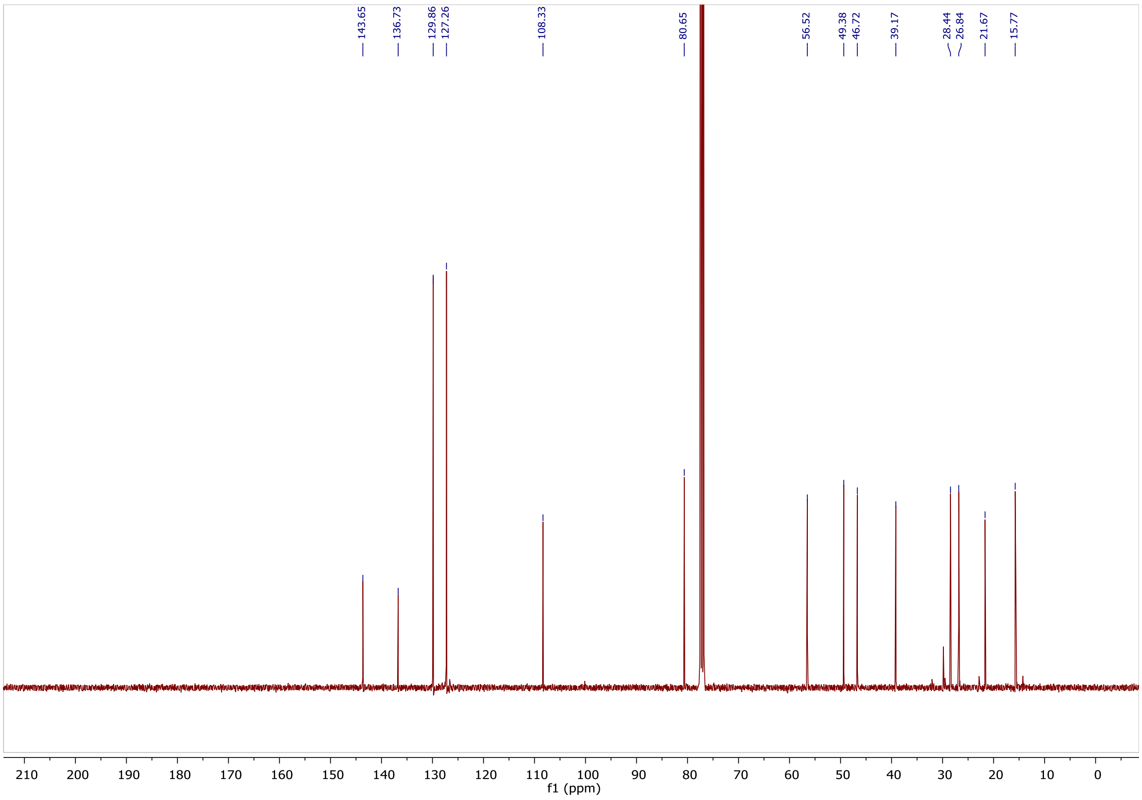

Compound 5aA: ^{1}H NMR (acetone-d_6, 400 MHz)

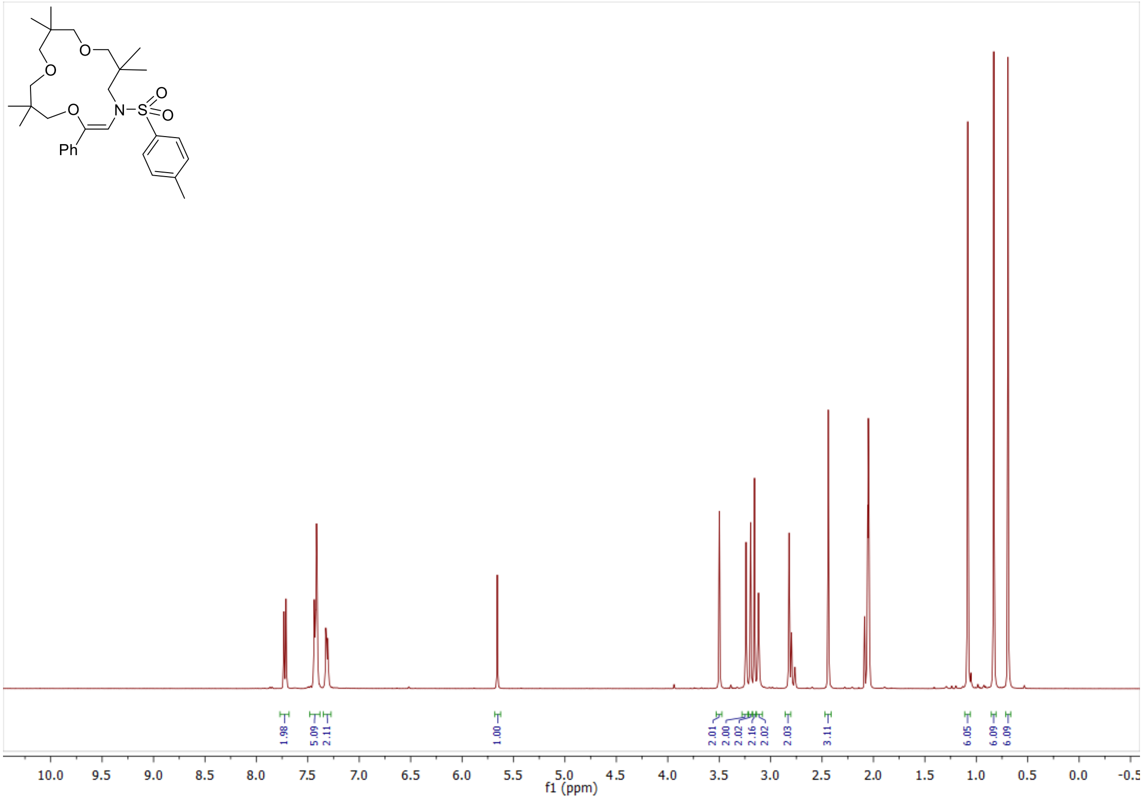

Compound 5aA: ^{13}C NMR (acetone-d_6, 100 MHz)

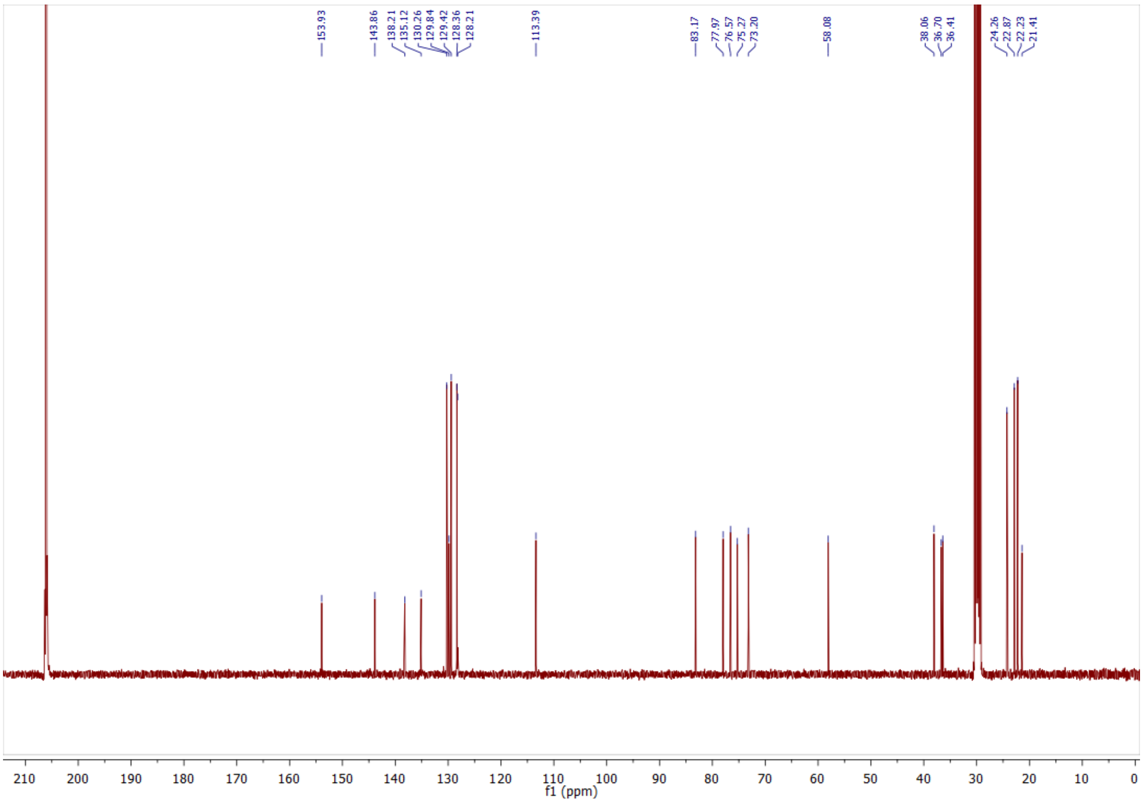

Compound 5bA: ^{1}H NMR (acetone-d_6, 400 MHz)

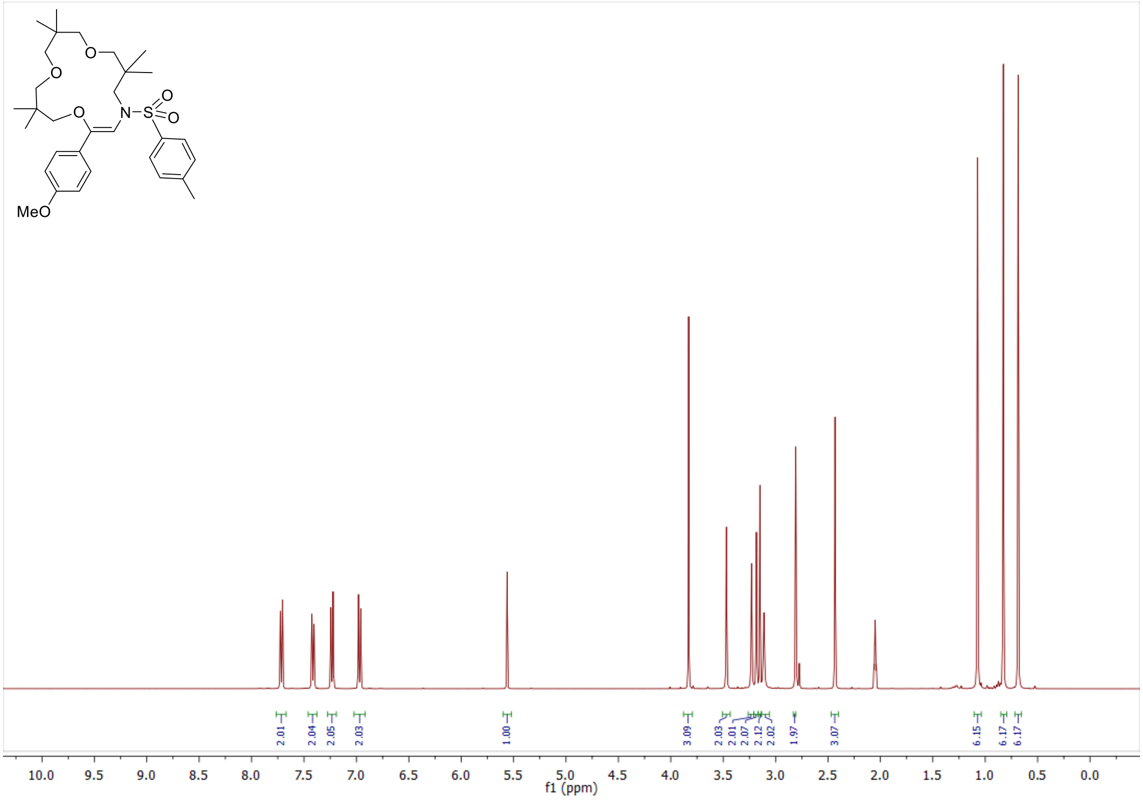

Compound 5bA: ^{13}C NMR (acetone-d_6, 100 MHz)

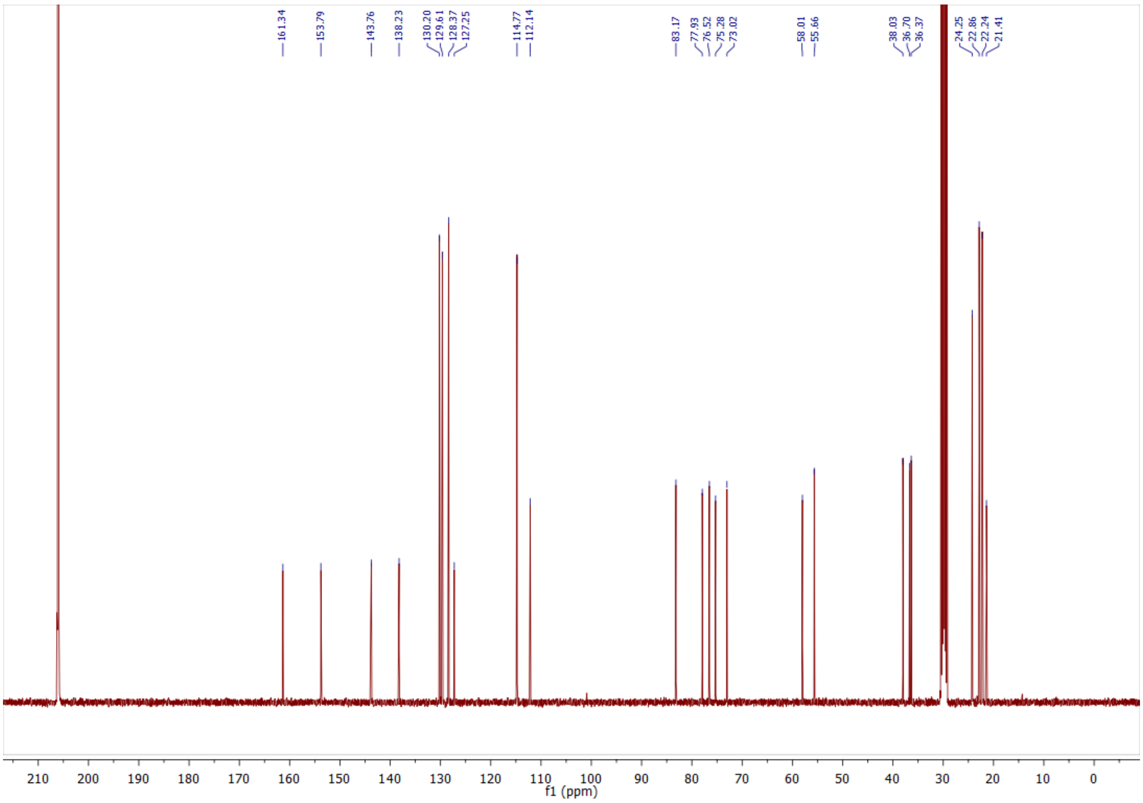

Compound 5cA: ^{1}H NMR (acetone-d_6, 400 MHz)

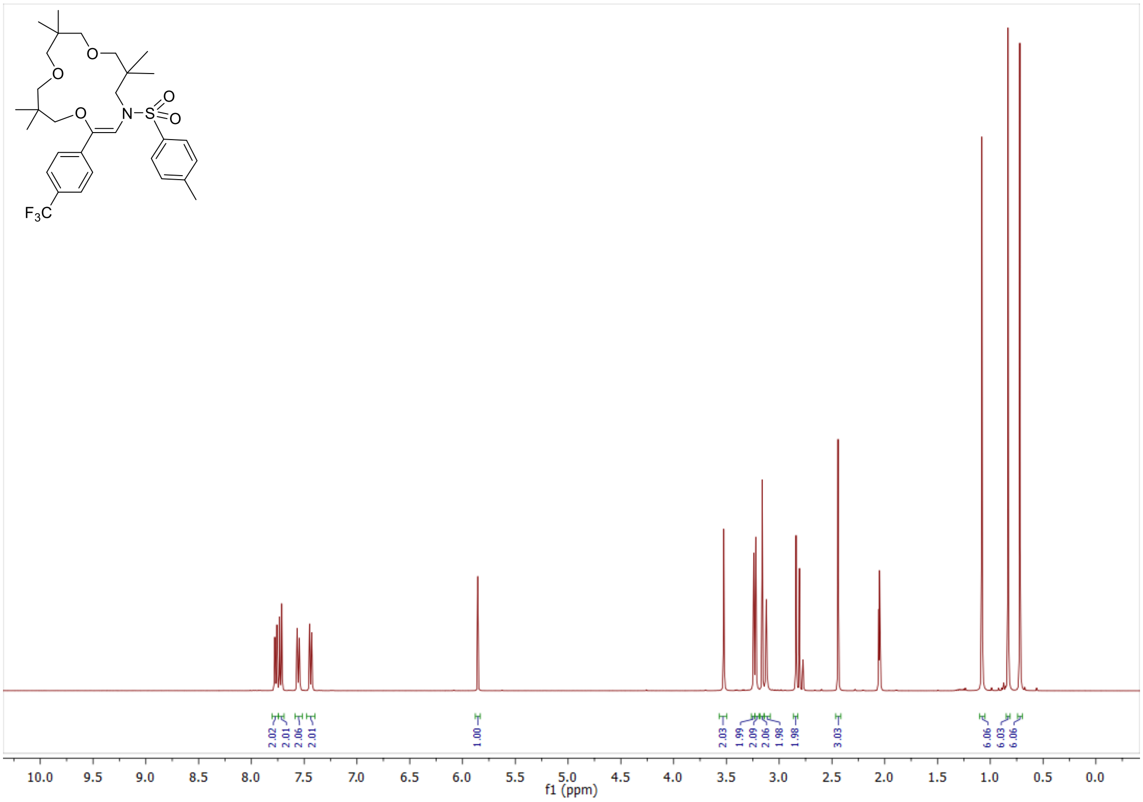

Compound 5cA: ^{13}C NMR (acetone-d_6, 100 MHz)

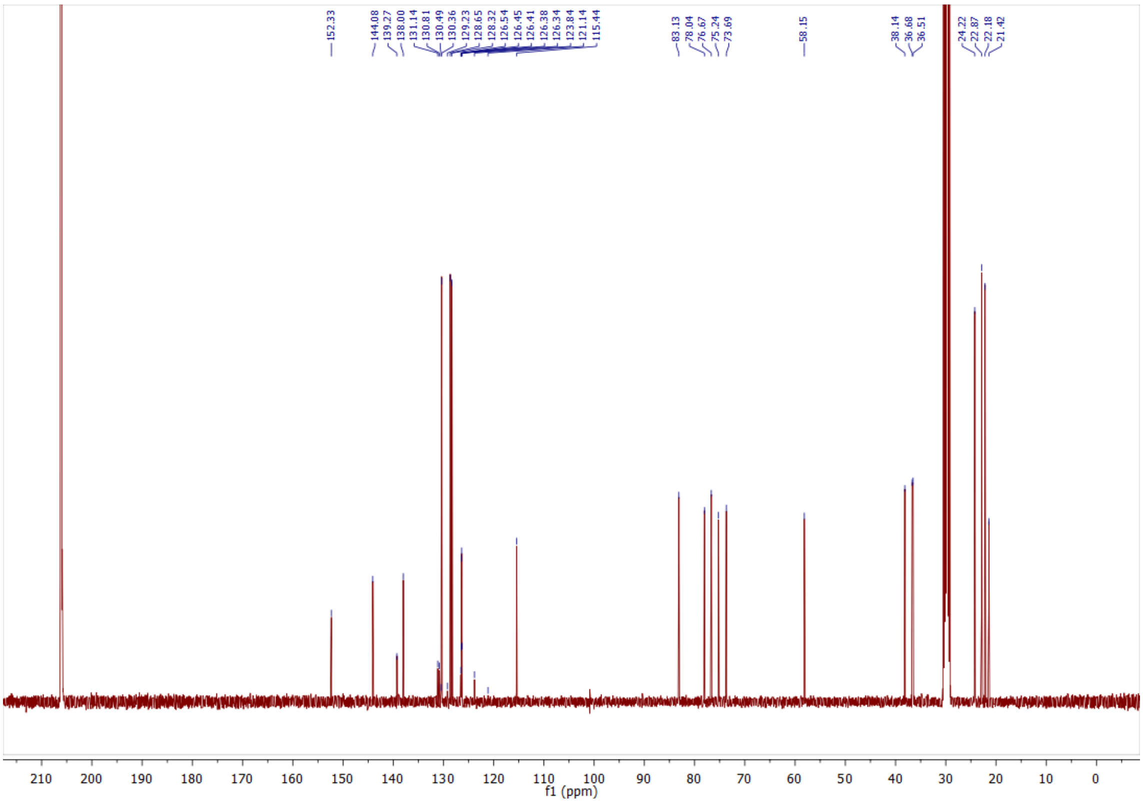

Compound 5gA: ^{1}H NMR (acetone-d_6, 400 MHz)

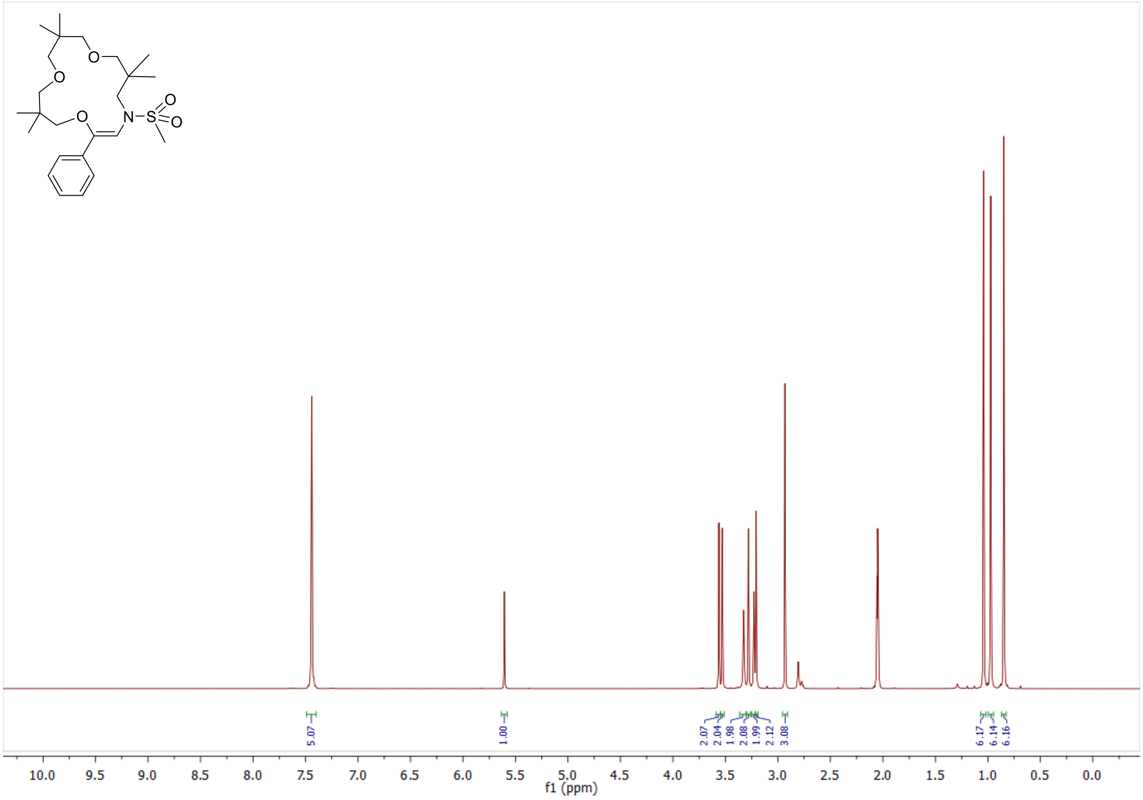

Compound 5gA: ^{13}C NMR (acetone-d_6, 100 MHz)

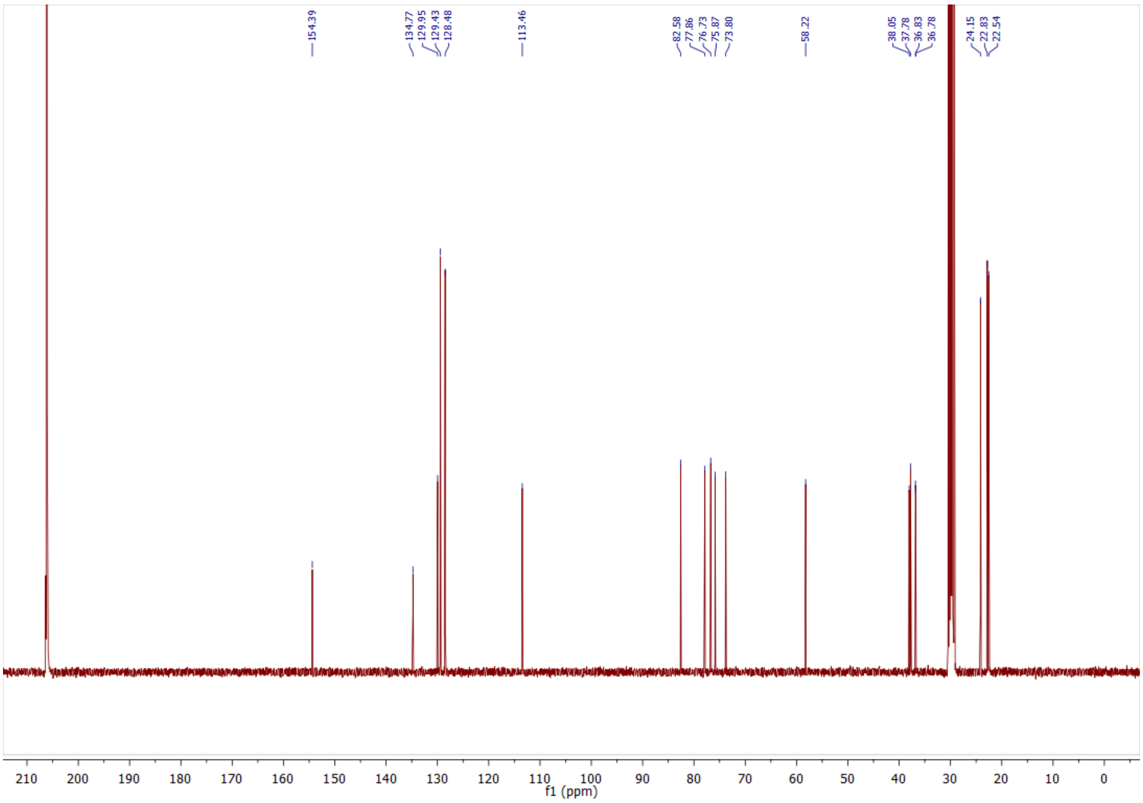

Compound 5hA: ^{1}H NMR ($CDCl_3$, 400 MHz)

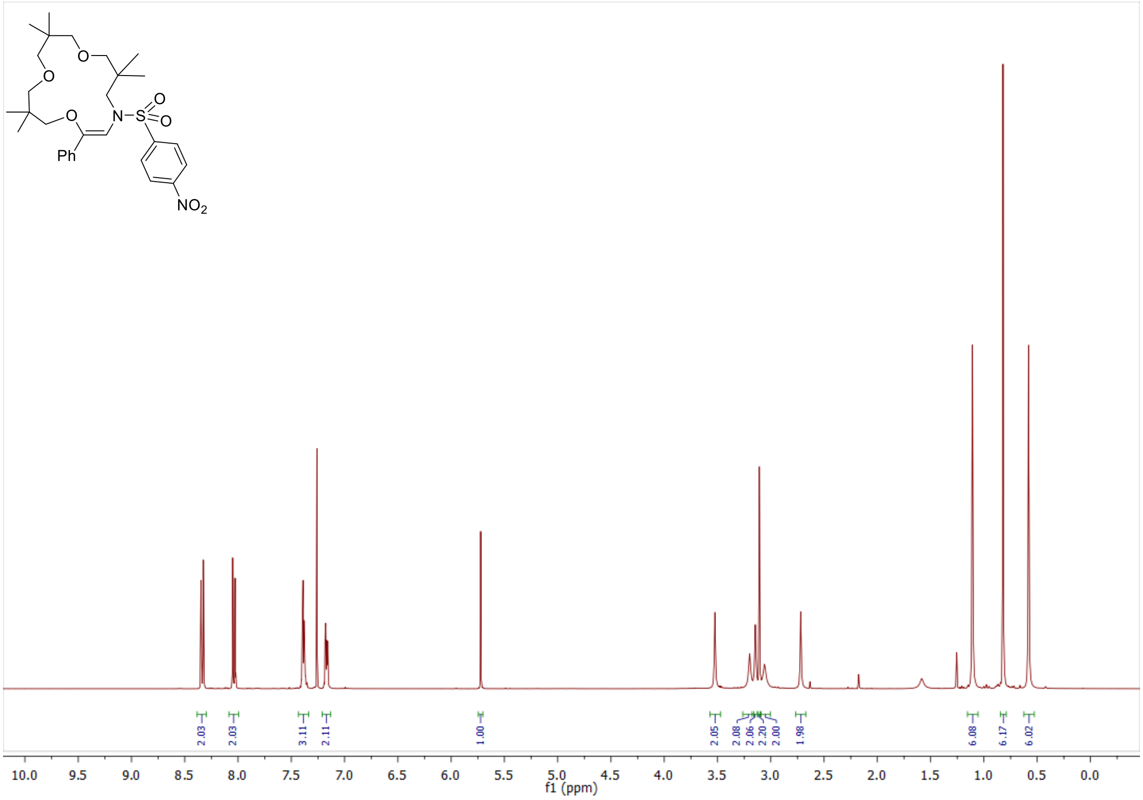

Compound 5hA: ^{13}C NMR ($CDCl_3$, 100 MHz)

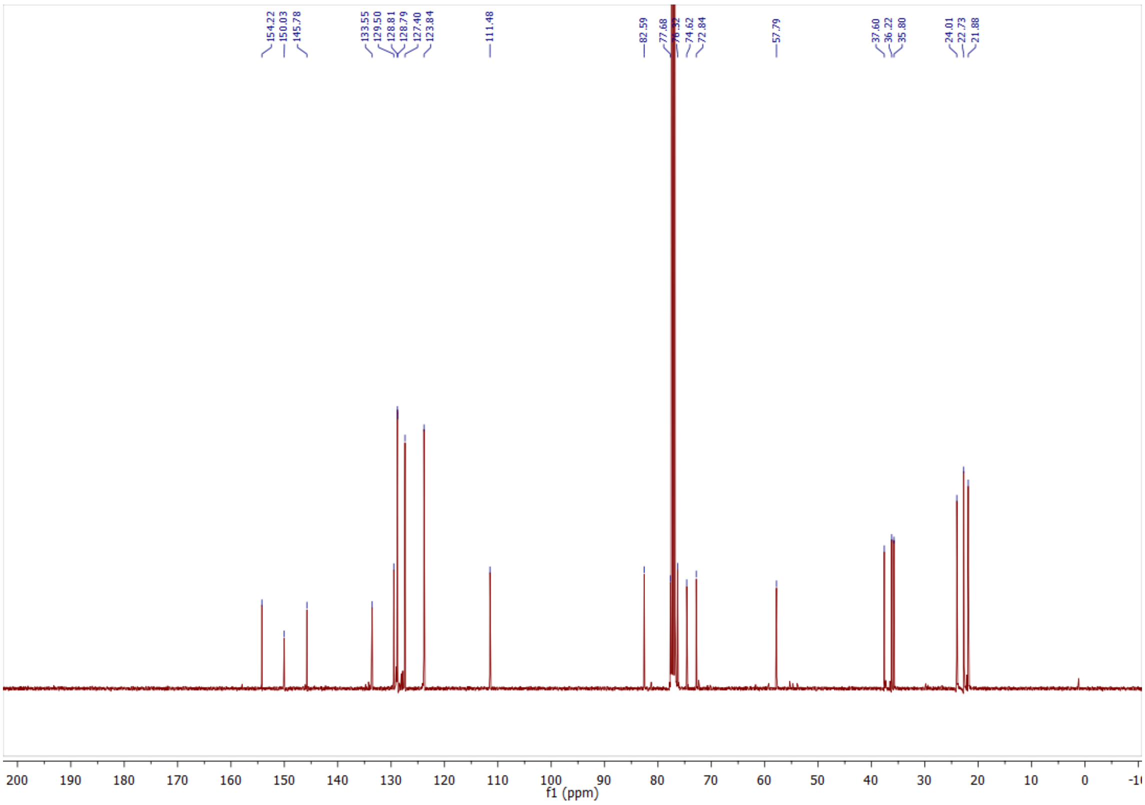

Compound 5jA: ^{1}H NMR (acetone-d_6, 400 MHz)

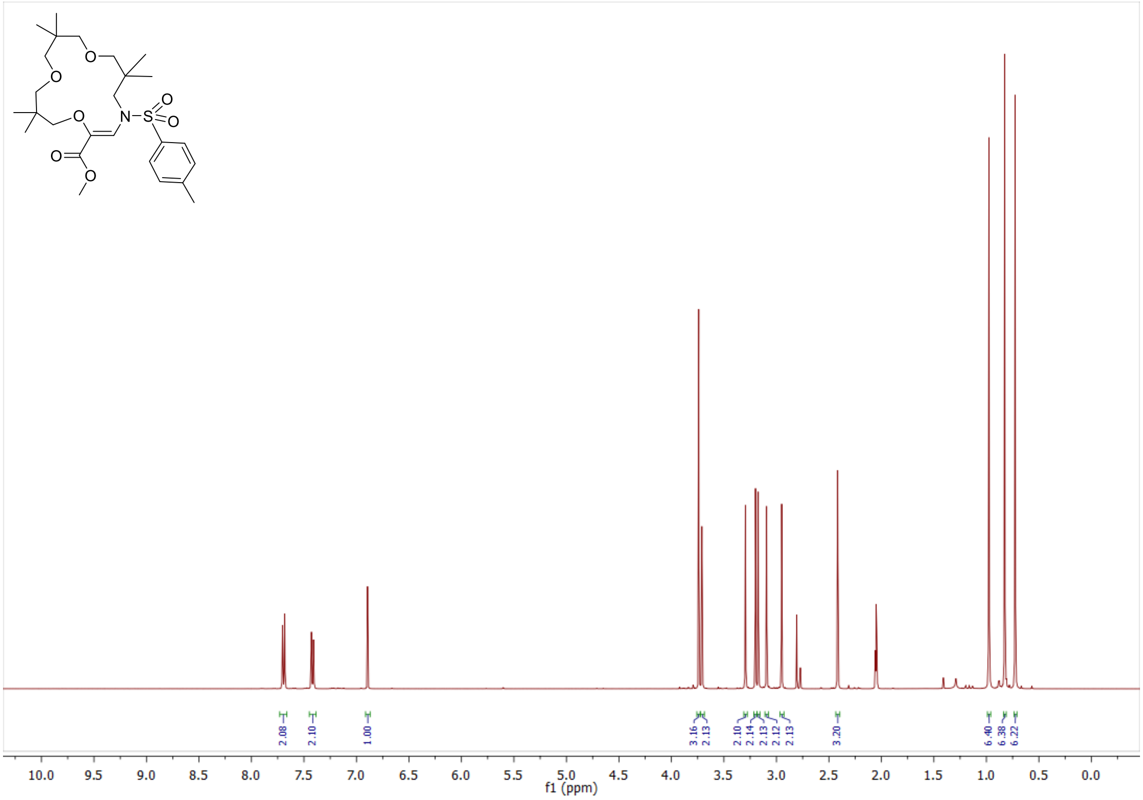

Compound 5jA: ^{13}C NMR (acetone-d_6, 100 MHz)

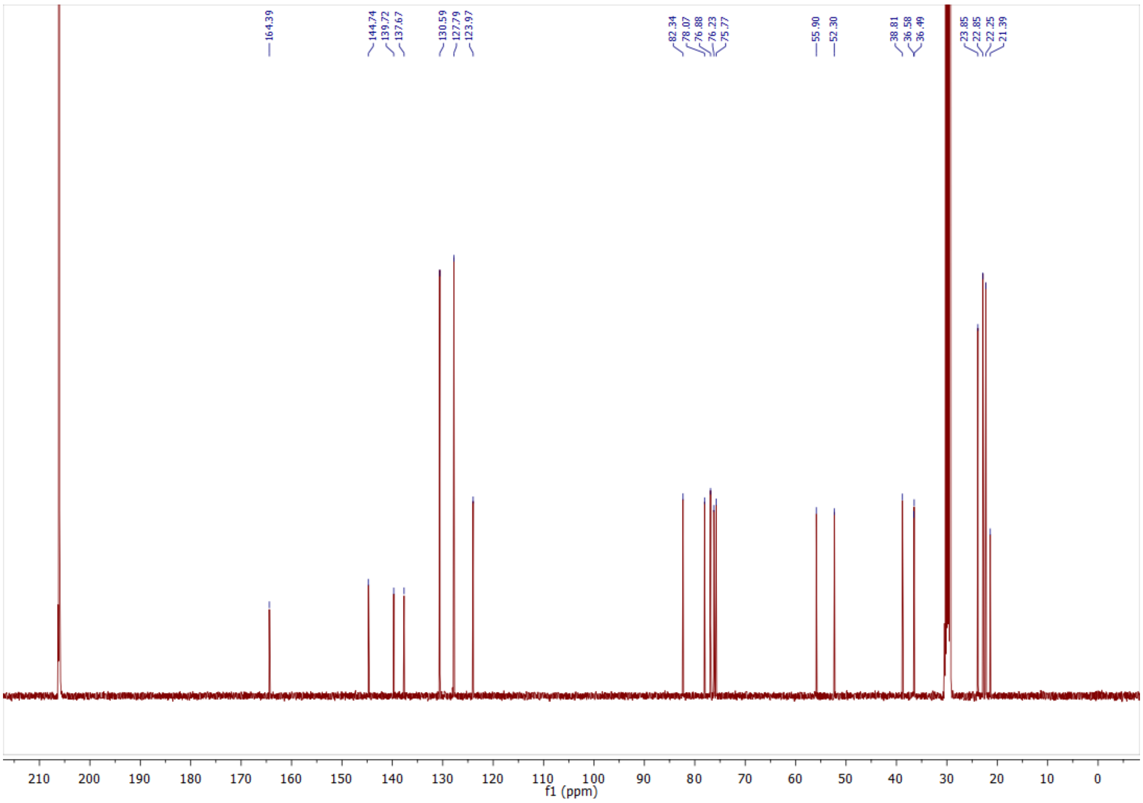

Compound 5kA: ^{1}H NMR (acetone-d_6, 400 MHz)

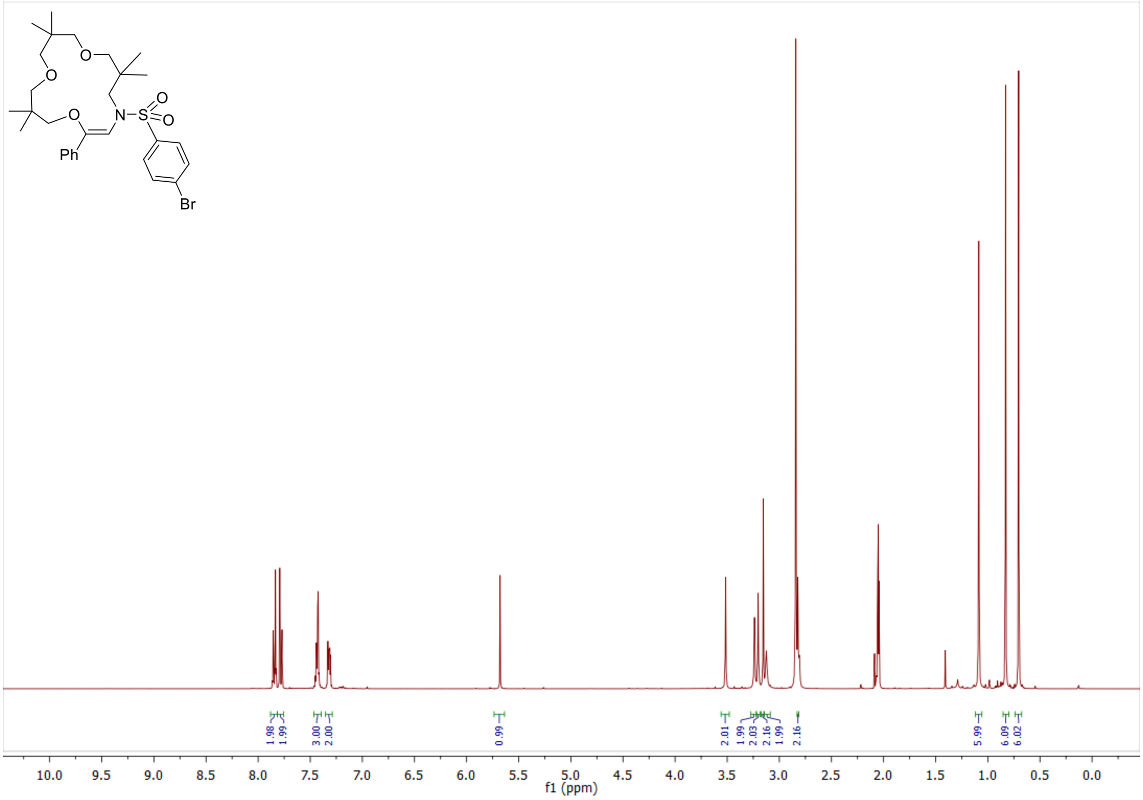

Compound 5kA: ^{13}C NMR (acetone-d_6, 100 MHz)

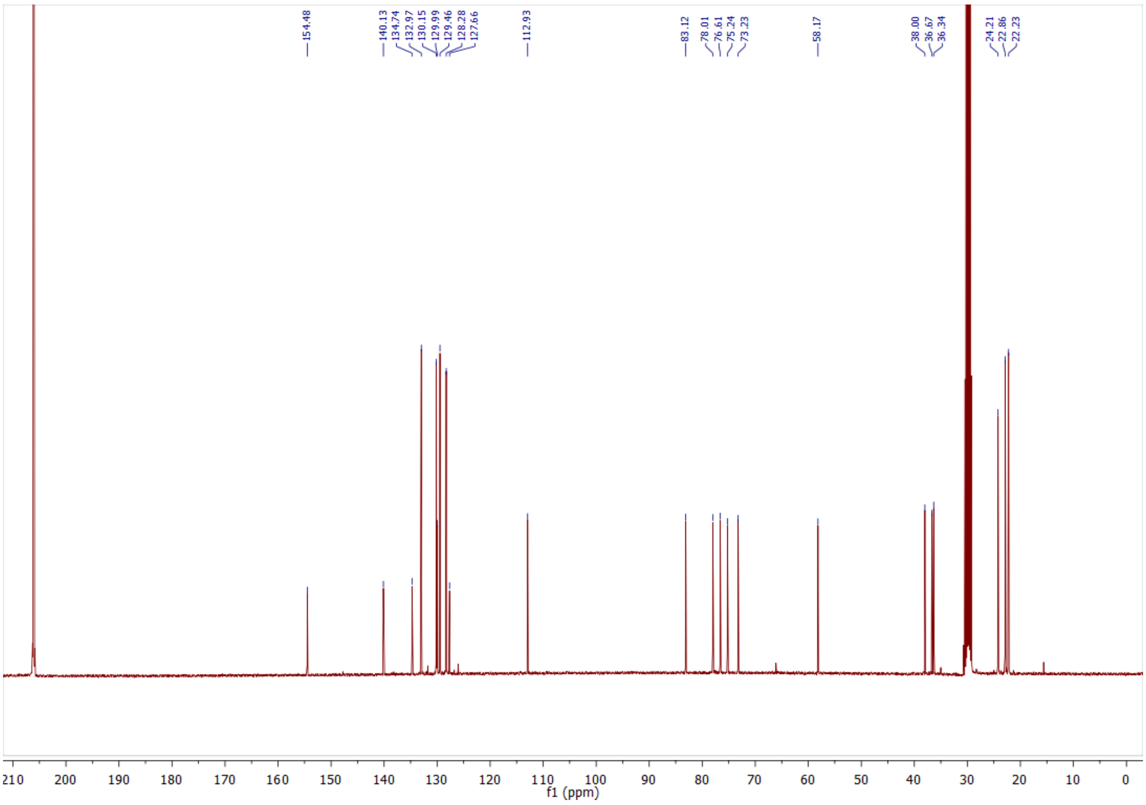

Compound 5lA: ^{1}H NMR (acetone-d_6, 400 MHz)

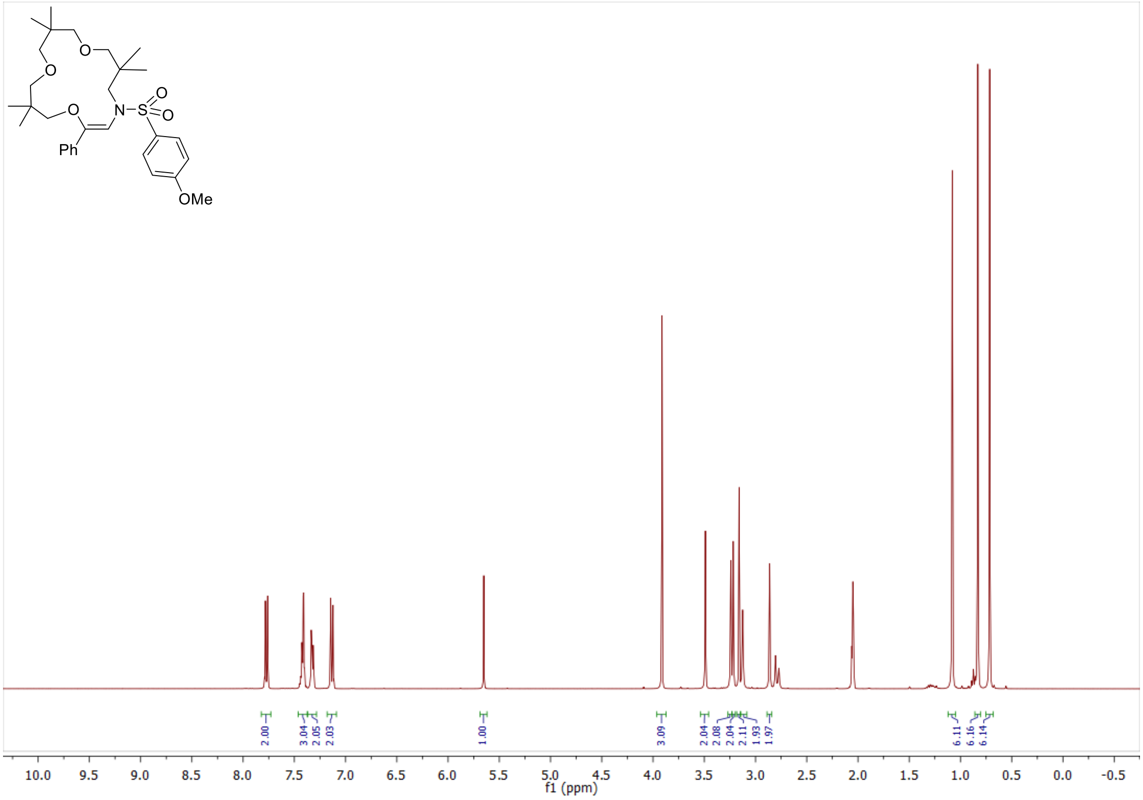

Compound 5lA: ^{13}C NMR (acetone-d_6, 100 MHz)

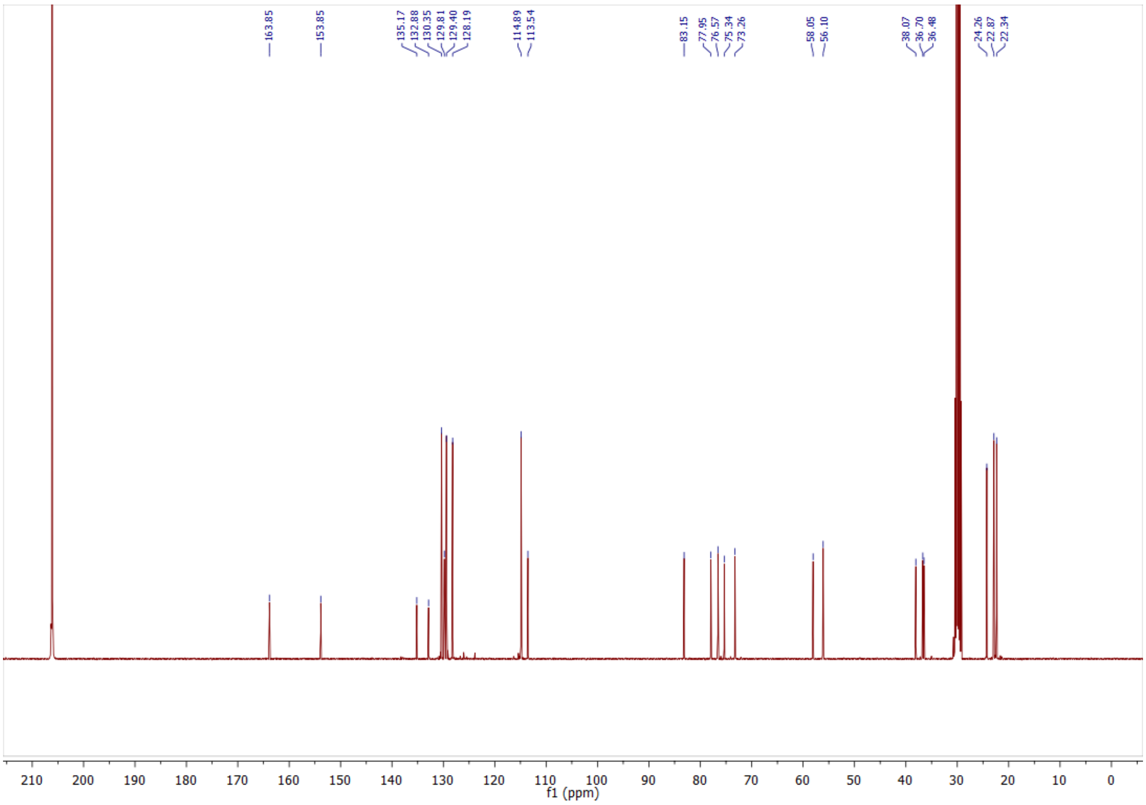

Compound 5mA: ^{1}H NMR (acetone-d_6, 400 MHz)

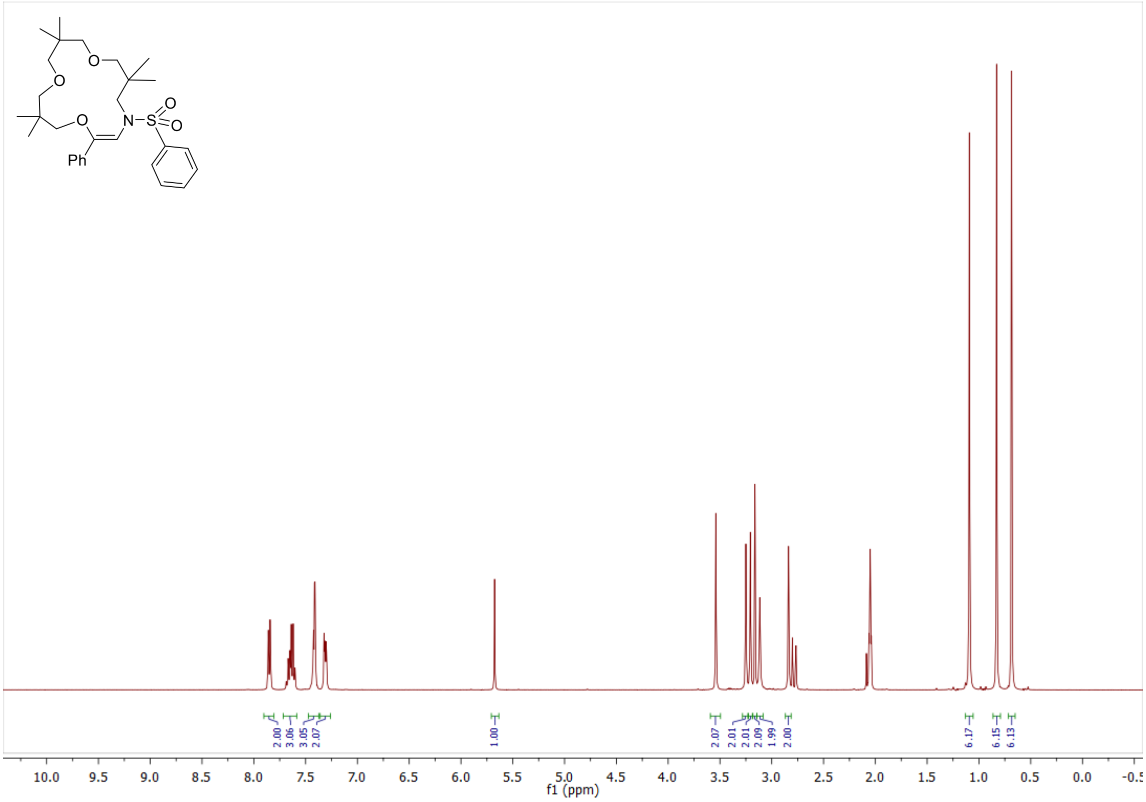

Compound 5mA: ^{13}C NMR (acetone-d_6, 100 MHz)

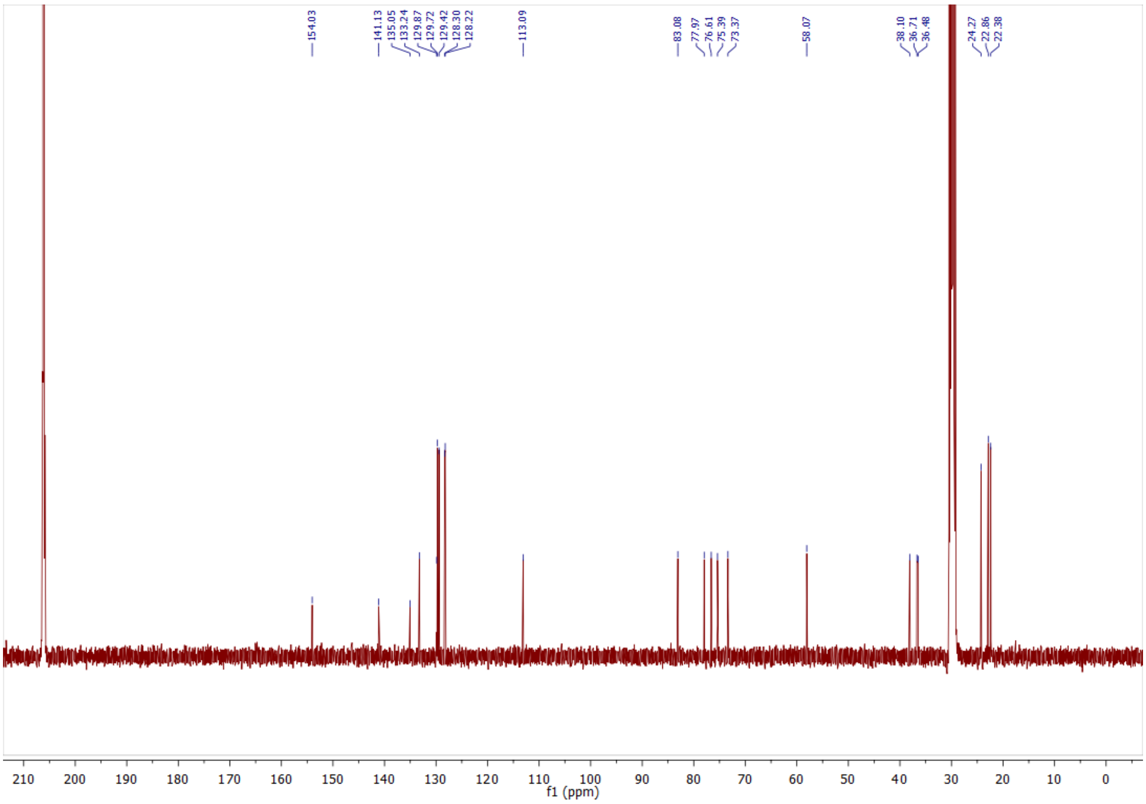

Compound 5nA: ^{1}H NMR (acetone-d_6, 400 MHz)

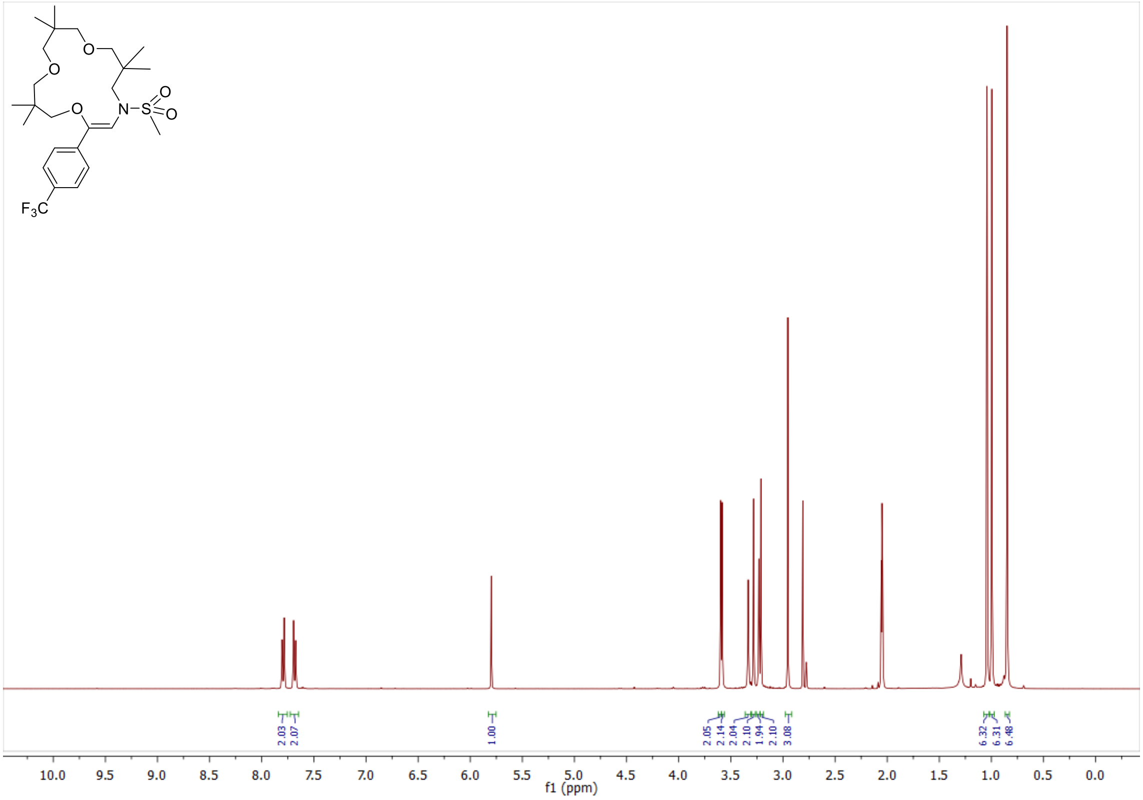

Compound 5nA: ^{13}C NMR (acetone-d_6, 100 MHz)

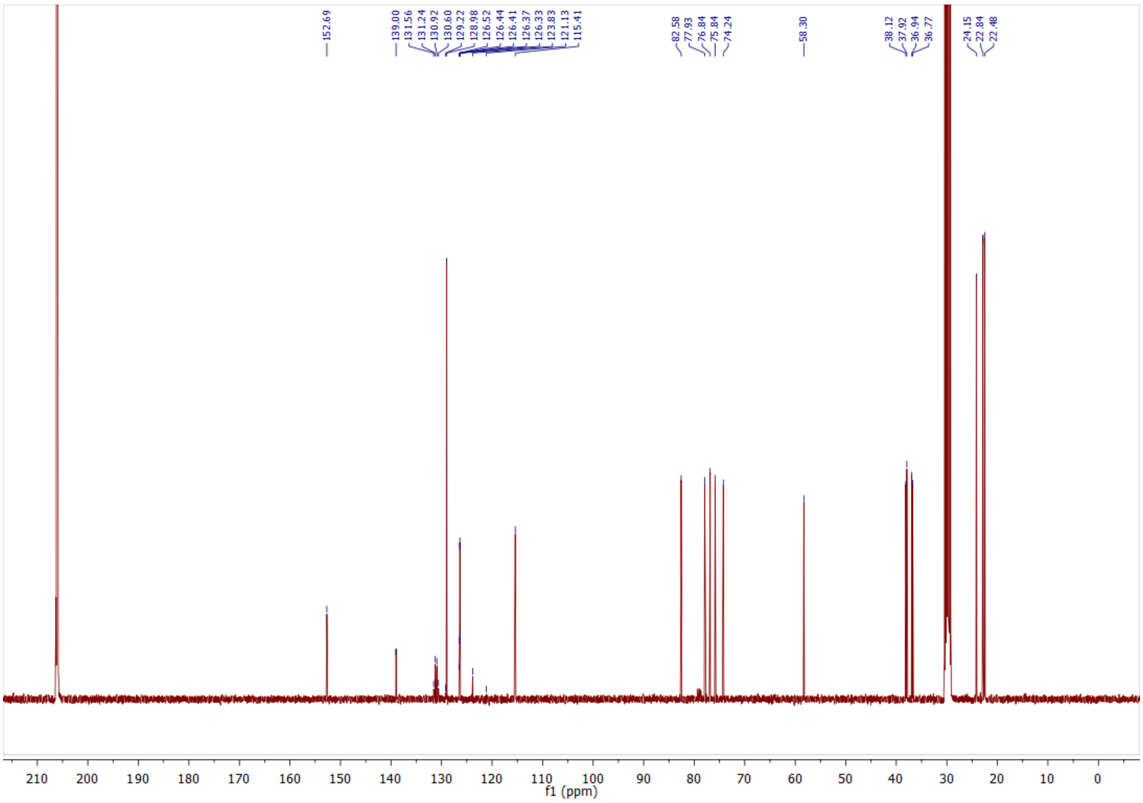

Compound 5aB: ^{1}H NMR (acetone-d_6, 400 MHz)

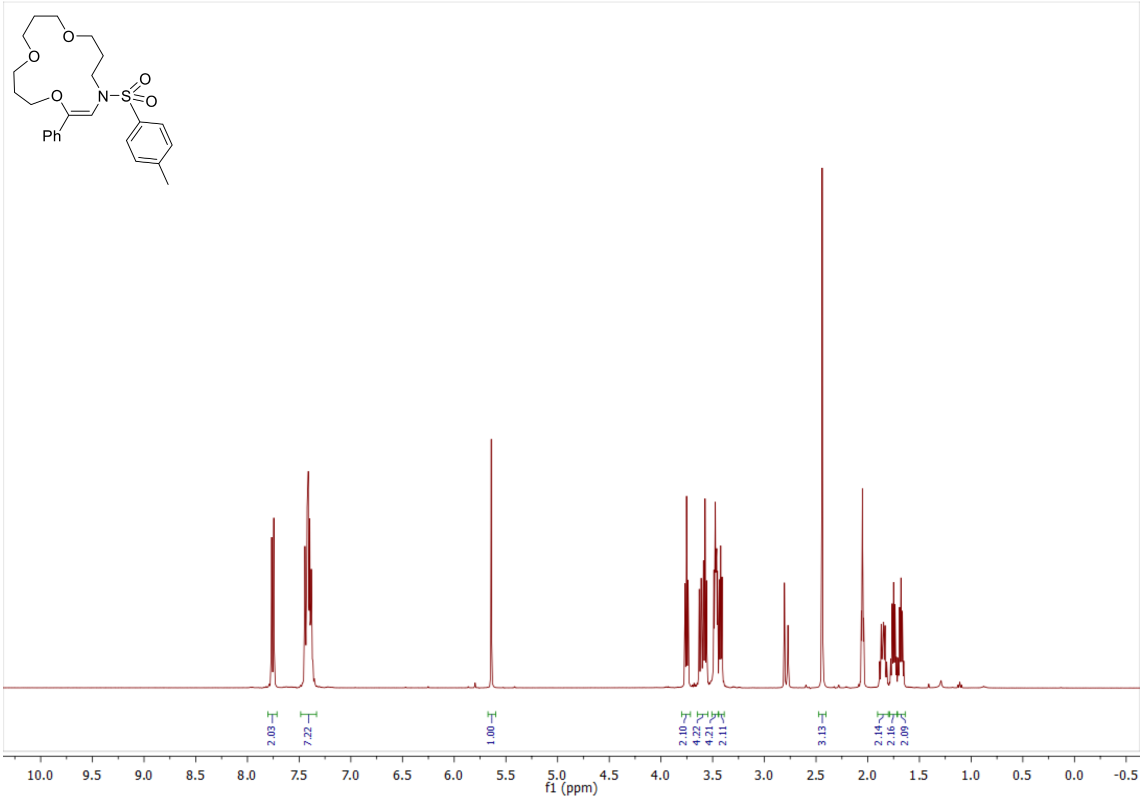

Compound 5aB: ^{13}C NMR (acetone-d_6, 100 MHz)

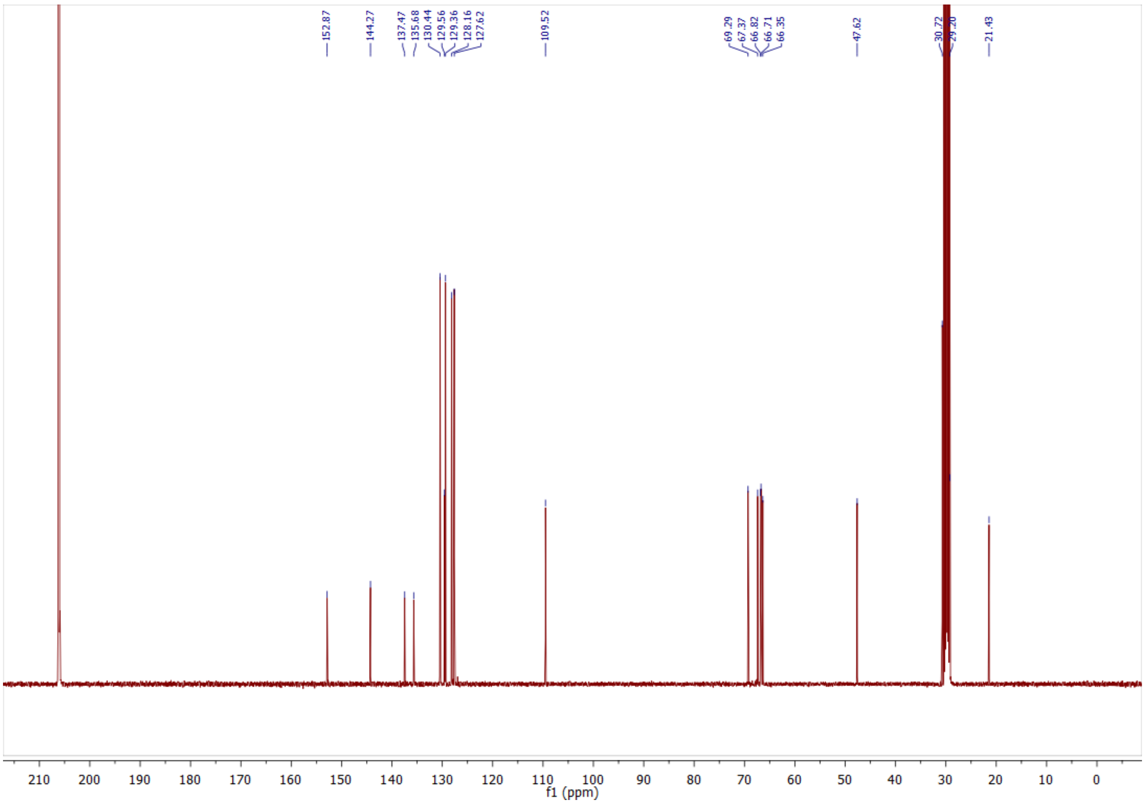

Compound 5hB: ^{1}H NMR (acetone-d_6, 400 MHz)

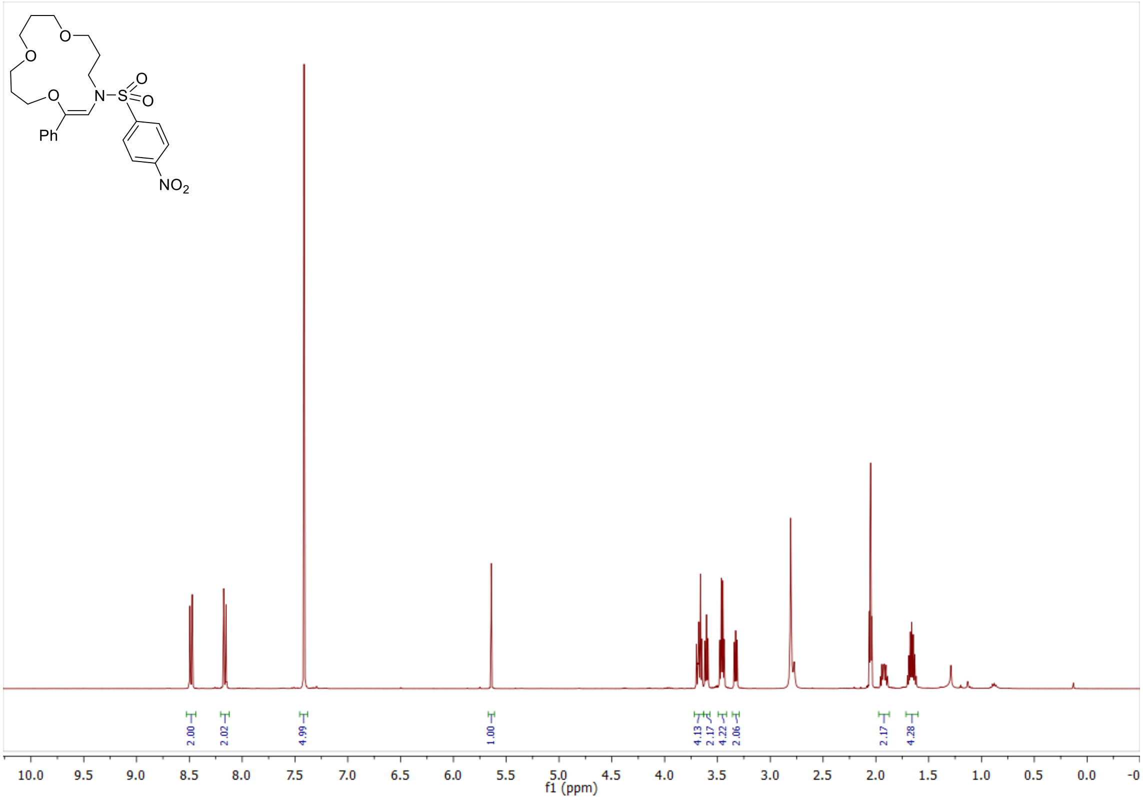

Compound 5hB: ^{13}C NMR (acetone-d_6, 100 MHz)

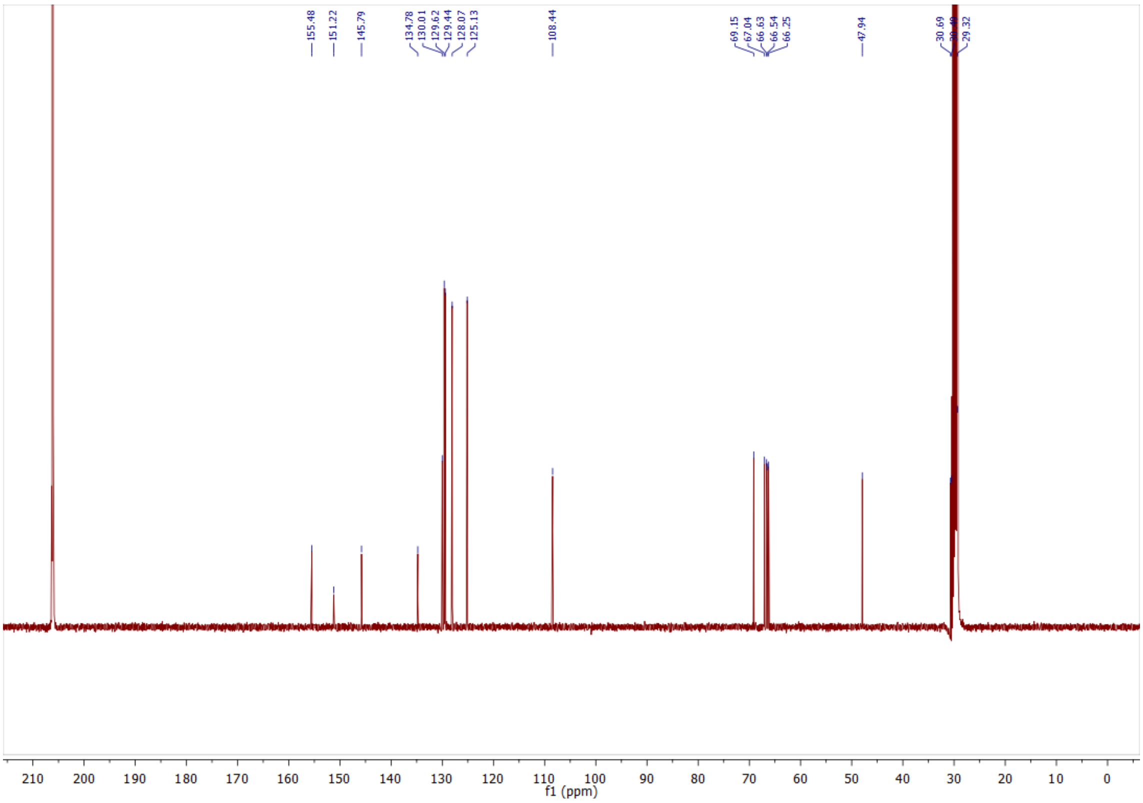

Compound 5kB: ^{1}H NMR (acetone-d_6, 400 MHz)

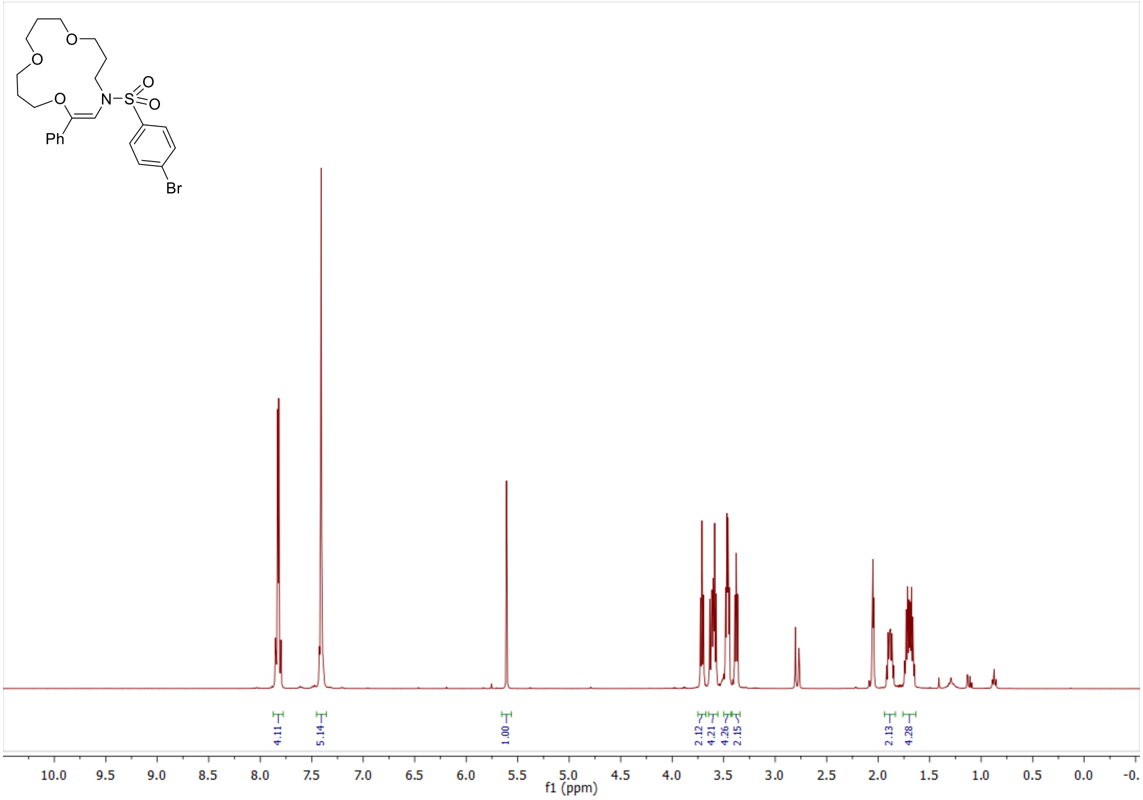

Compound 5kB: ^{13}C NMR (acetone-d_6, 100 MHz)

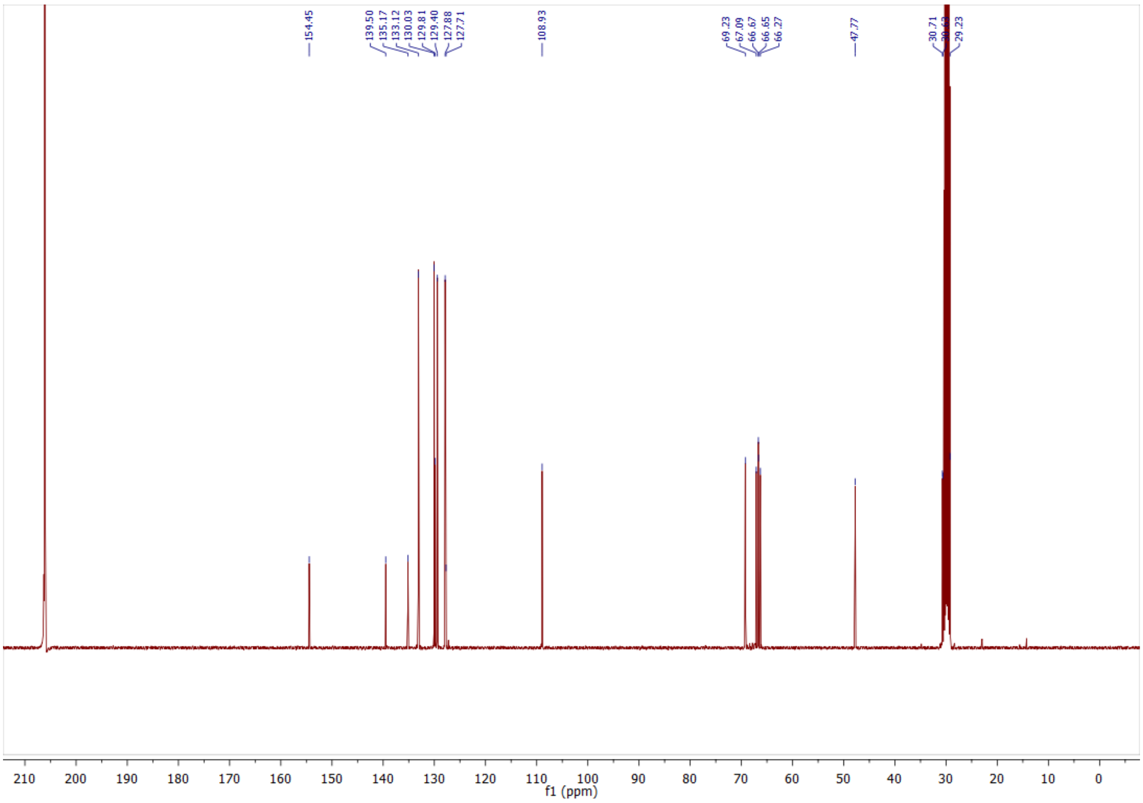

Compound 5lB: ^{1}H NMR (acetone-d_6, 400 MHz)

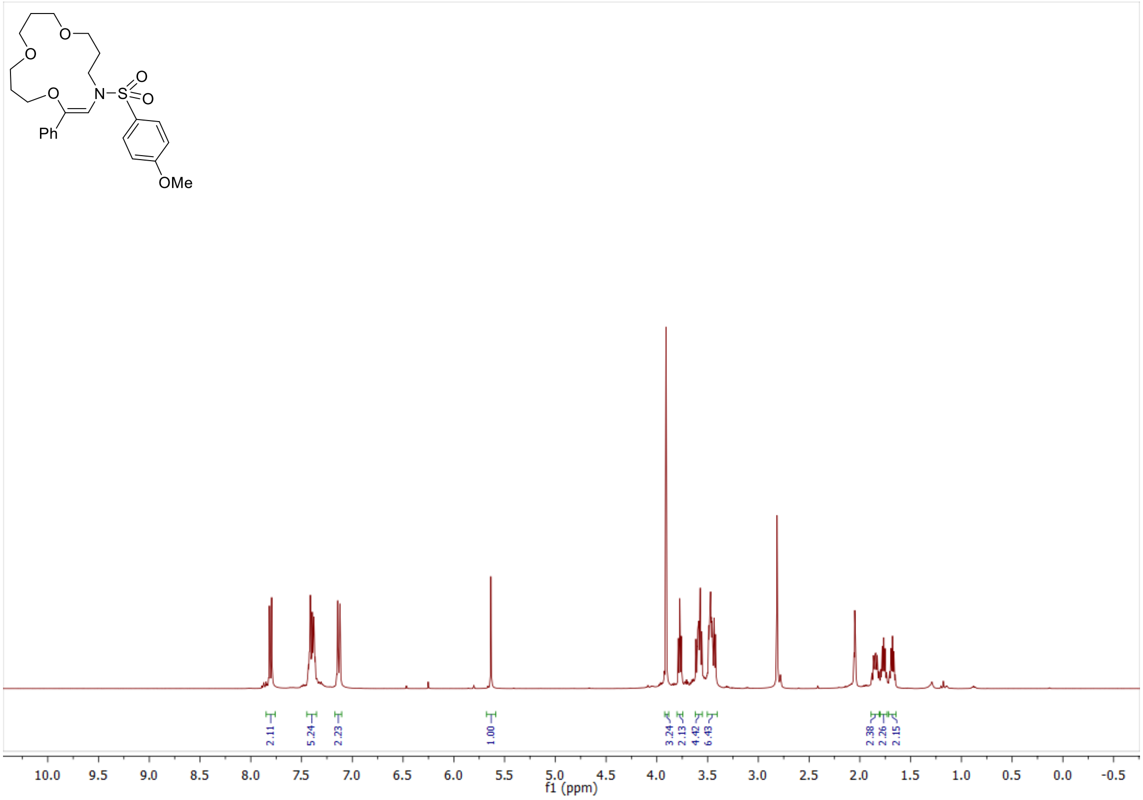

Compound 5lB: ^{13}C NMR (acetone-d_6, 100 MHz)

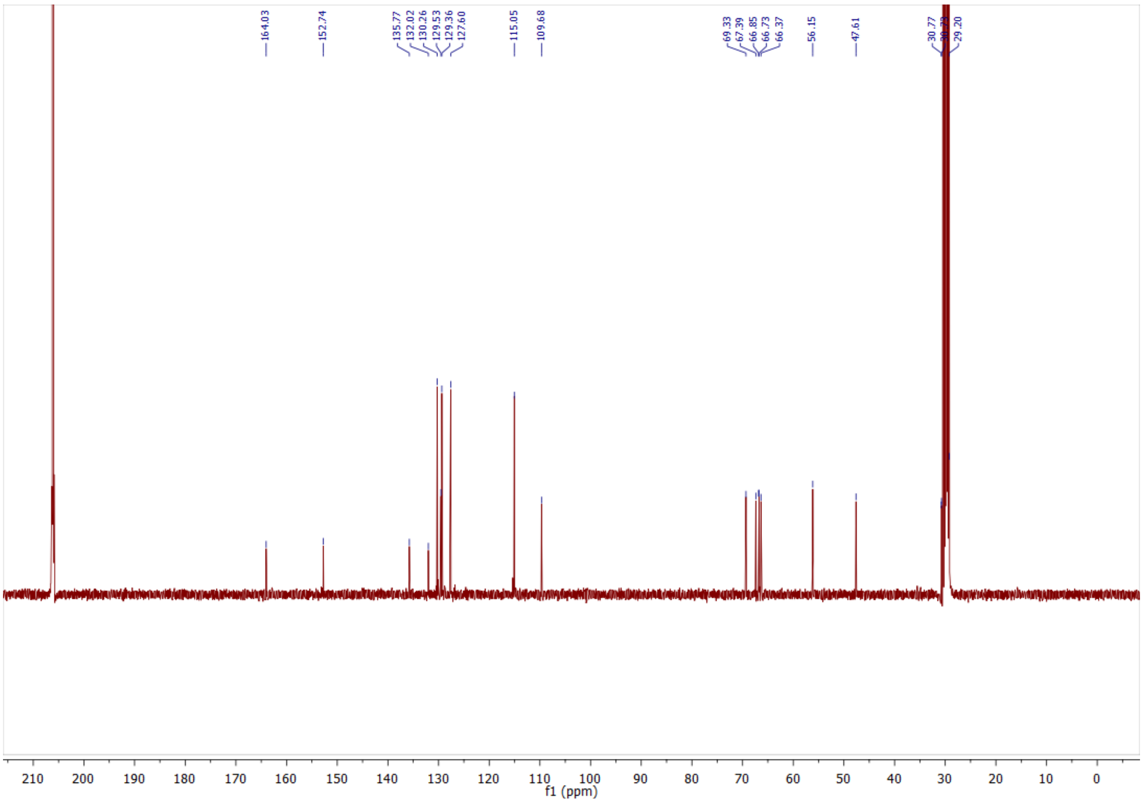

Compound 5mB: ^{1}H NMR (acetone-d_6, 400 MHz)

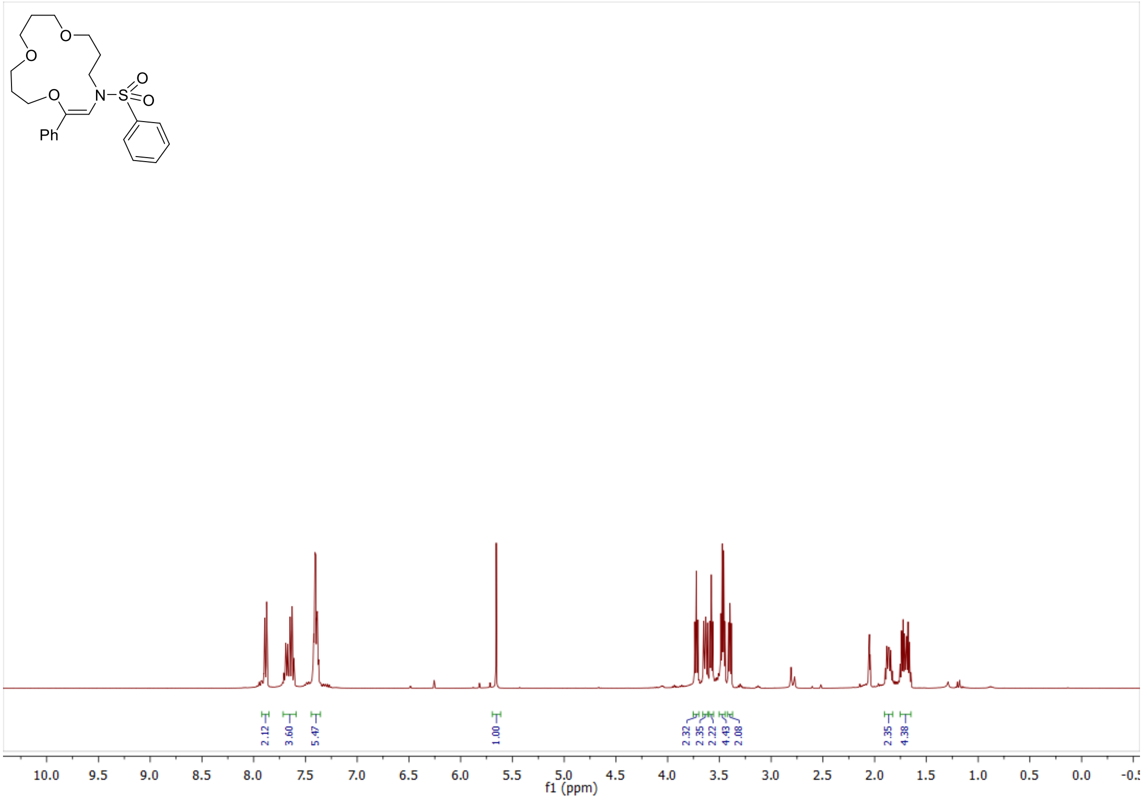

Compound 5mB: ^{13}C NMR (acetone-d_6, 100 MHz)

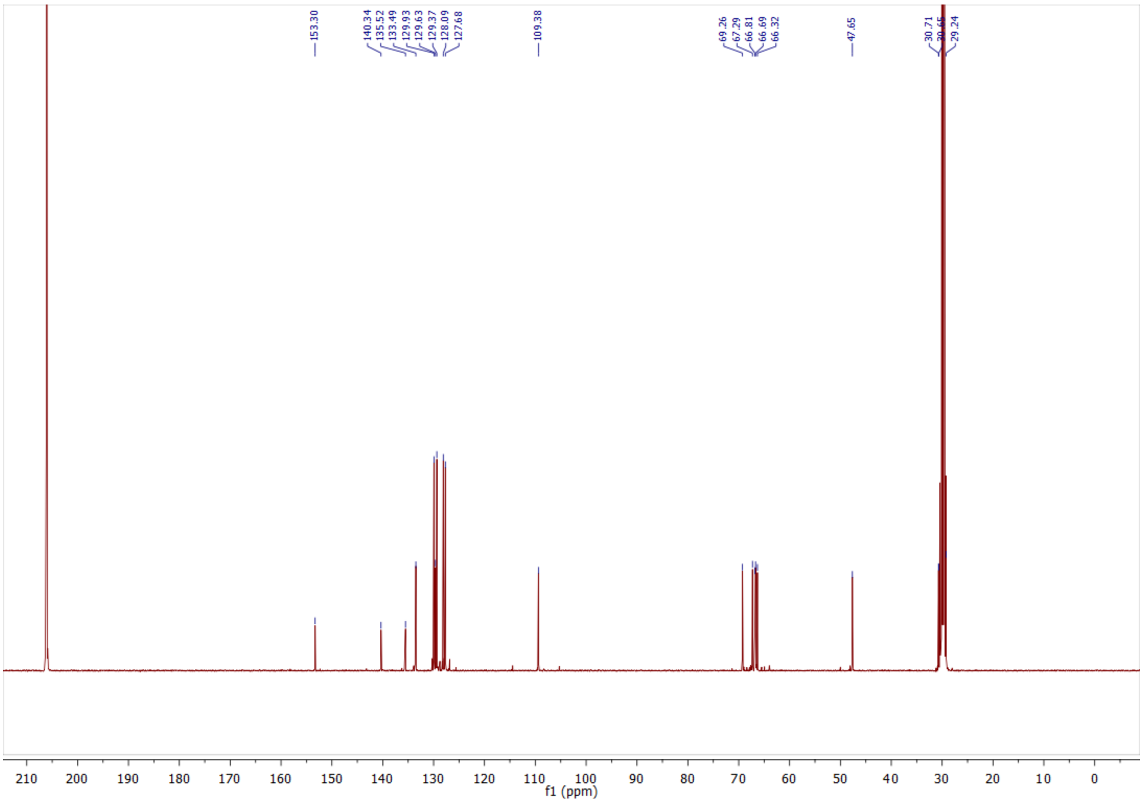

Compound (*E*)-5pA: ^{1}H NMR (acetone-d_6, 500 MHz)

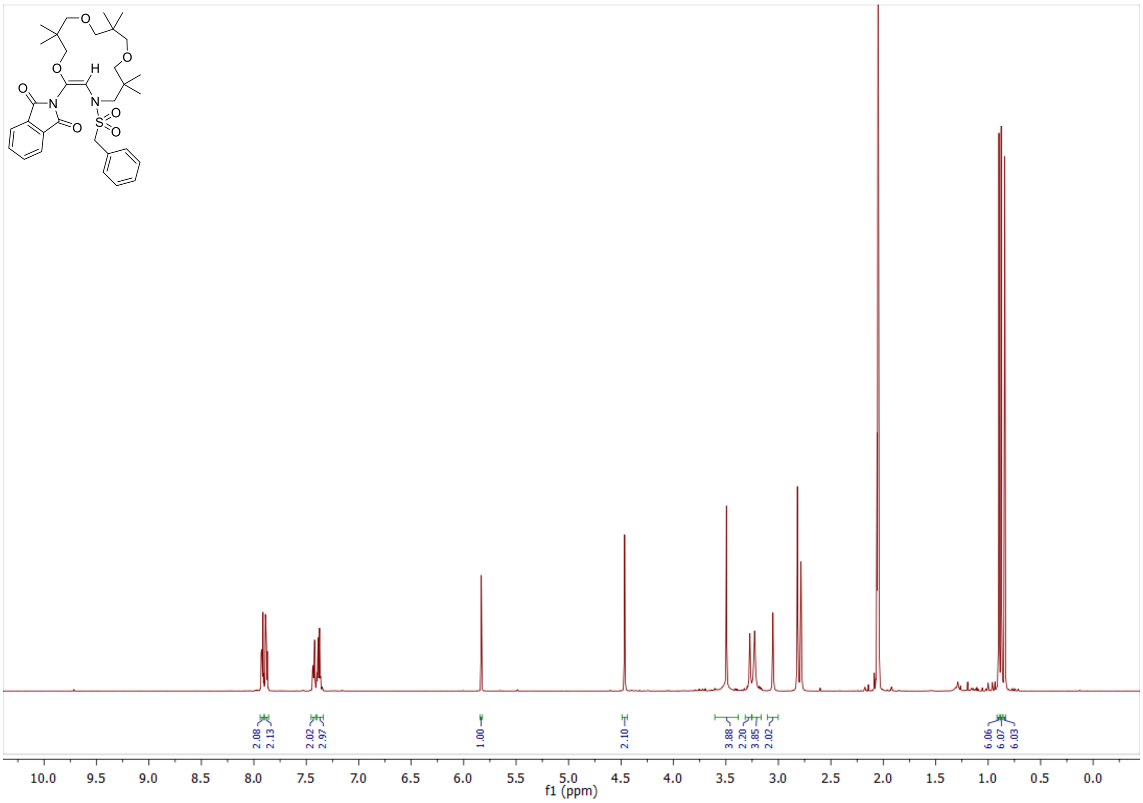

Compound (*E*)-5pA, ^{13}C NMR (acetone-d_6, 100 MHz)

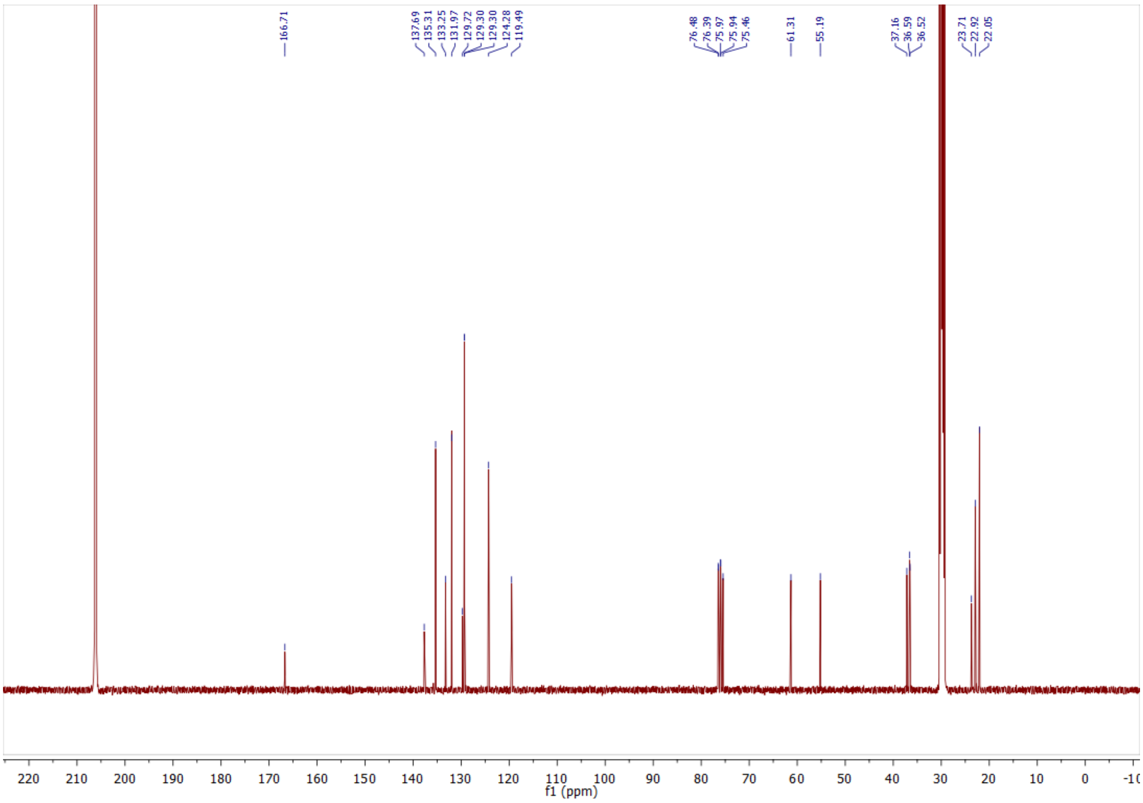

Compound (*Z*)-5pA: ^{1}H NMR ($CDCl_3$, 400 MHz)

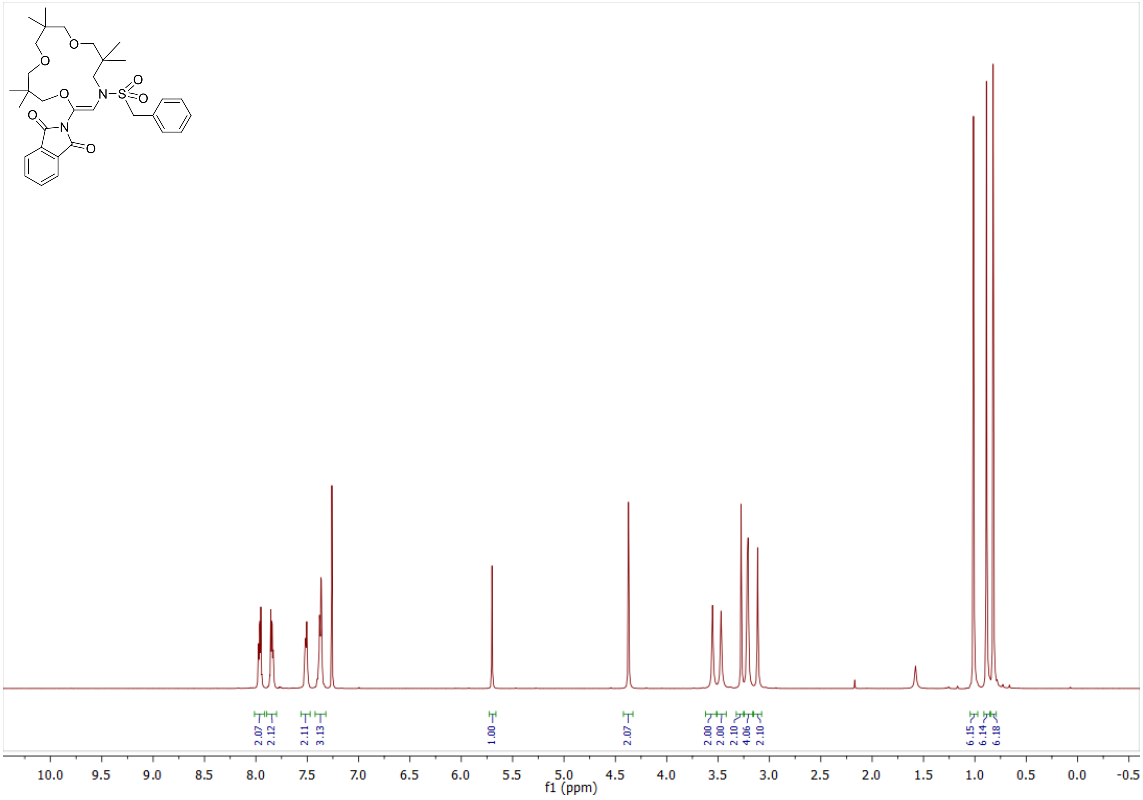

Compound (*Z*)- 5pA: ^{13}C NMR ($CDCl_3$, 100 MHz)

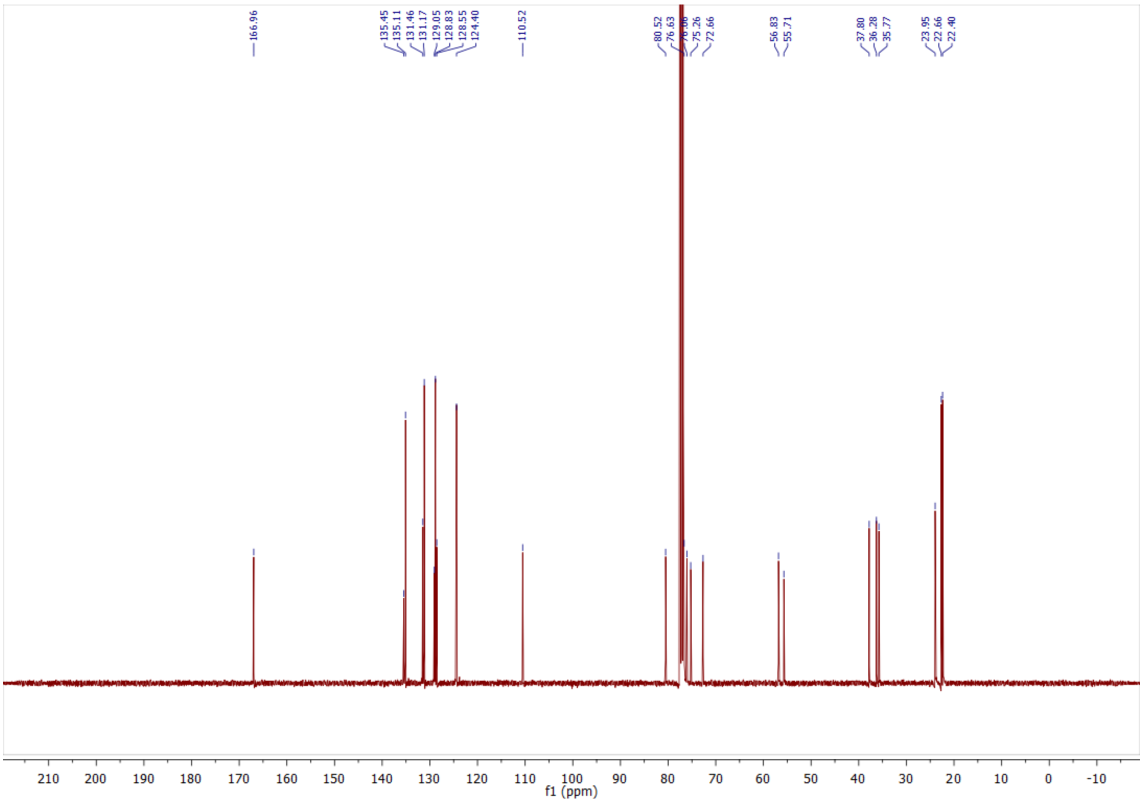

Compound (*E*)-5qA: ^{1}H NMR ($CDCl_3$, 400 MHz)

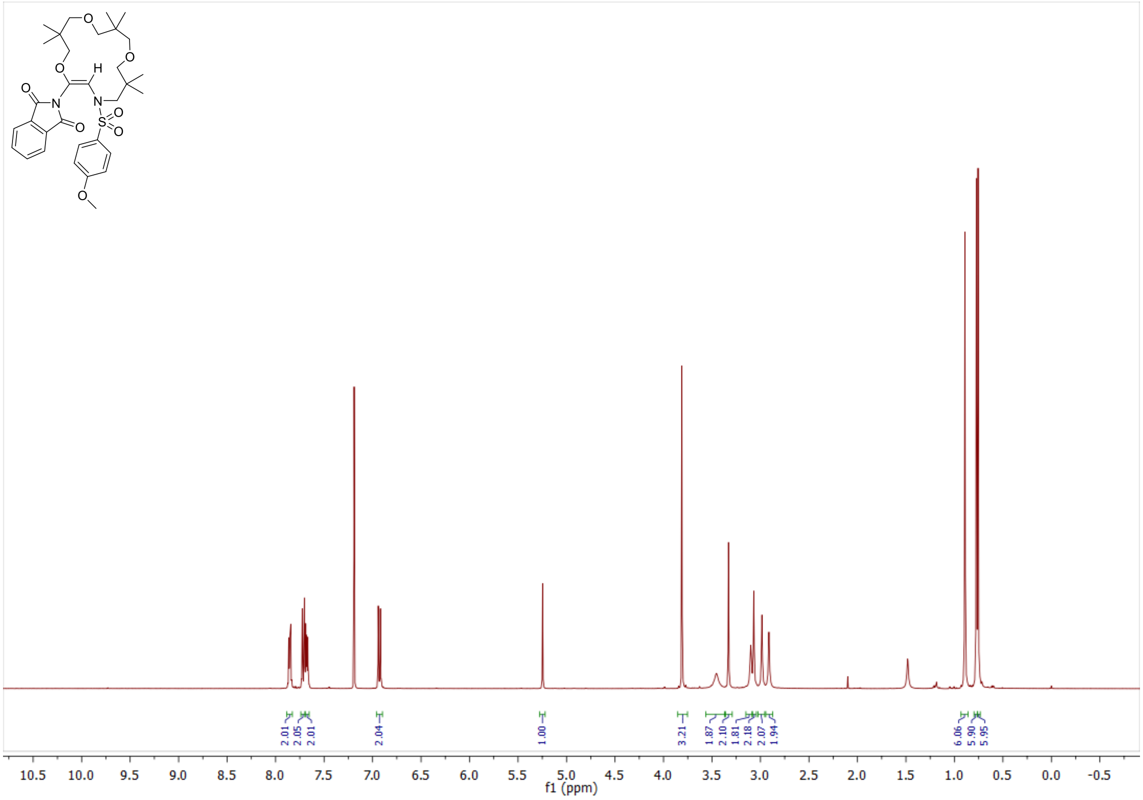

Compound (*E*)-5qA: ^{13}C NMR ($CDCl_3$, 100 MHz)

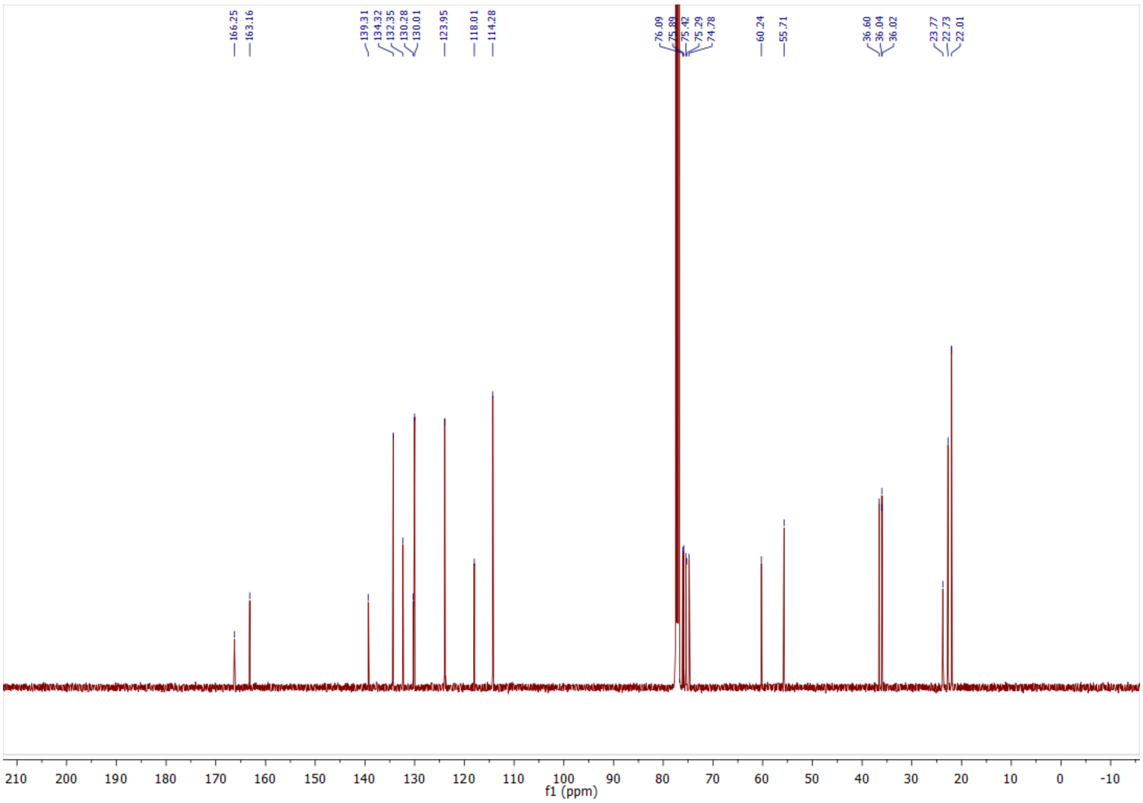

Compound (*Z*)-5qA: ^{1}H NMR ($CDCl_3$, 400 MHz)

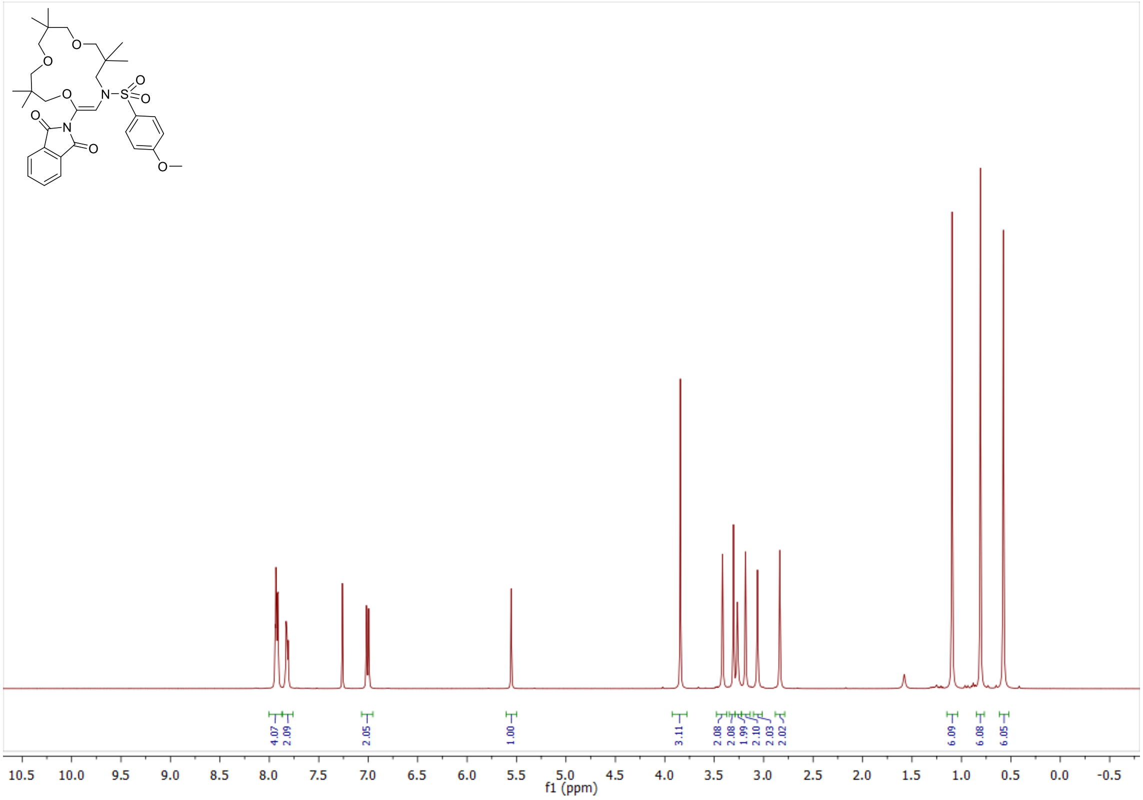

Compound (*Z*)-5qA: ^{13}C NMR ($CDCl_3$, 100 MHz)

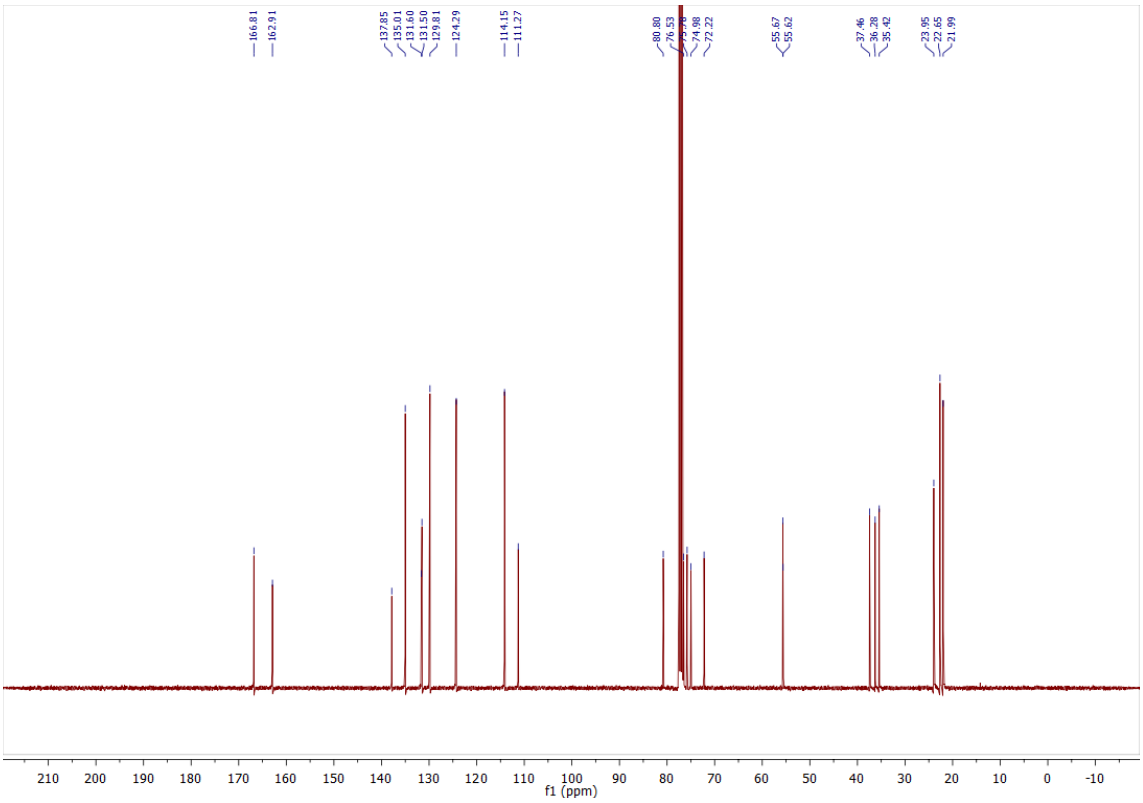

Compound 6gA: ^{1}H NMR (acetone-d_6, 400 MHz)

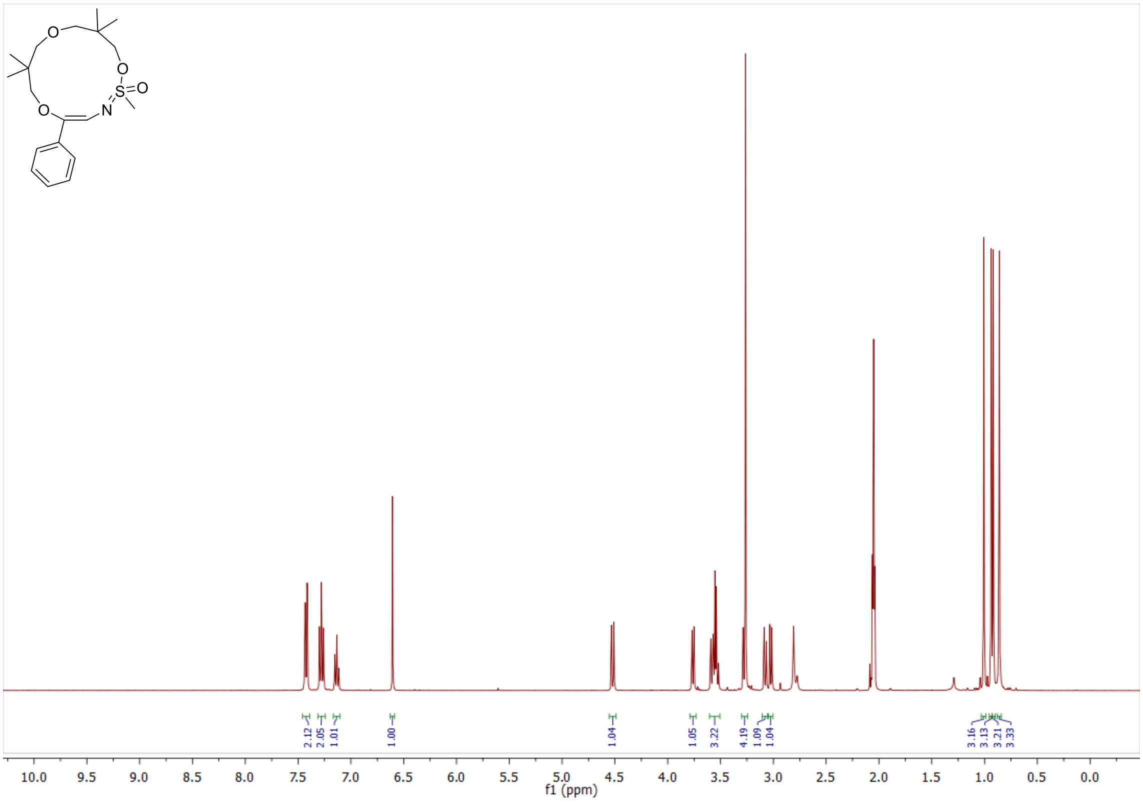

Compound 6gA: ^{13}C NMR (acetone-d_6, 100 MHz)

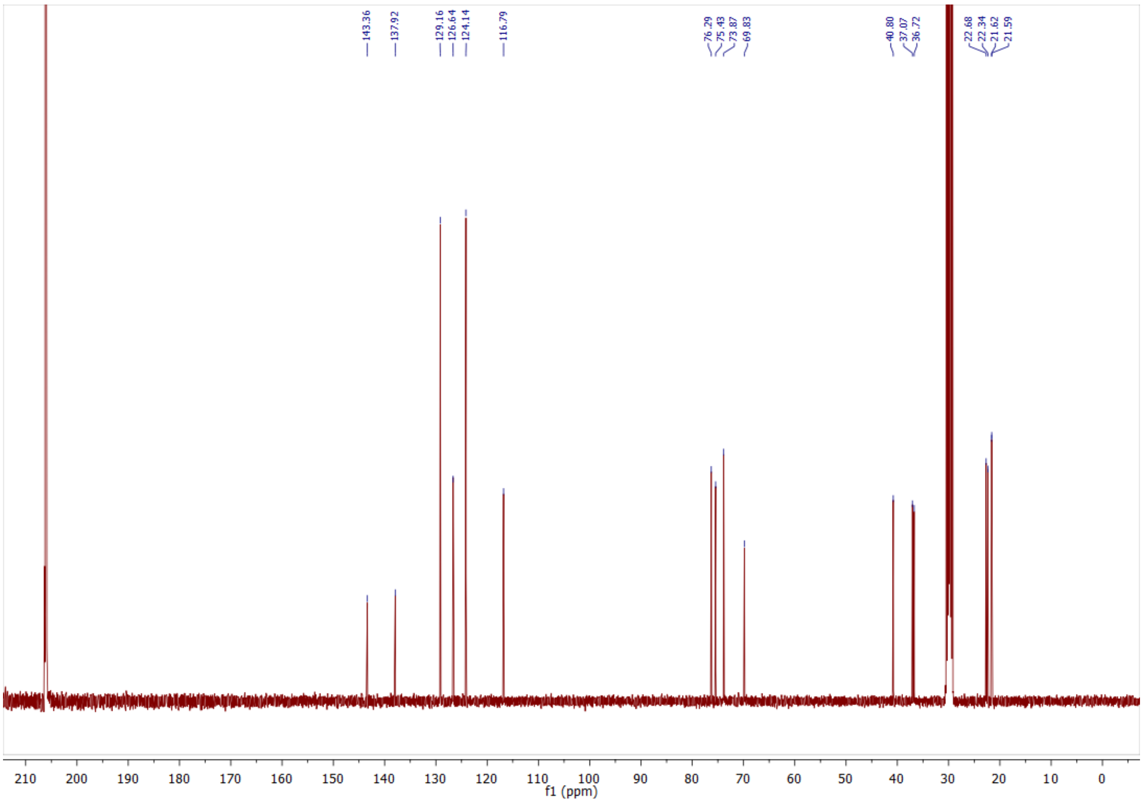

Compound 6nA: ^{1}H NMR (acetone-d_6, 400 MHz)

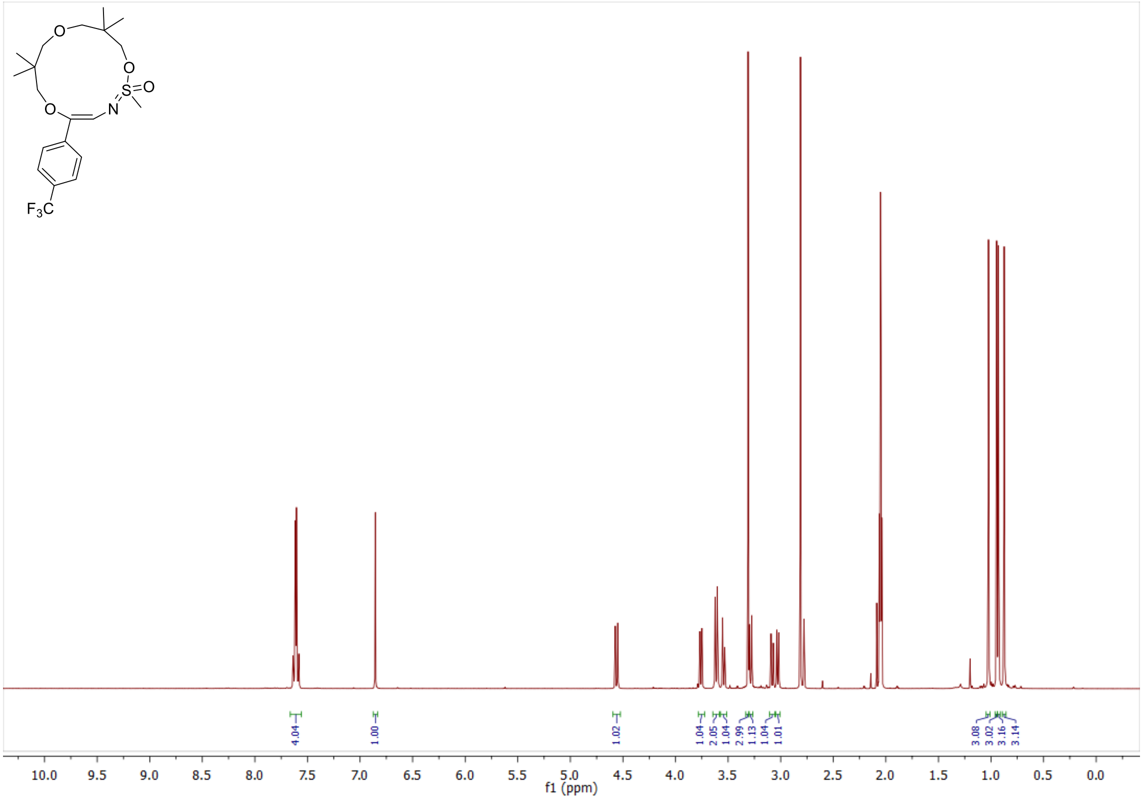

Compound 6nA: ^{13}C NMR (acetone-d_6, 100 MHz)

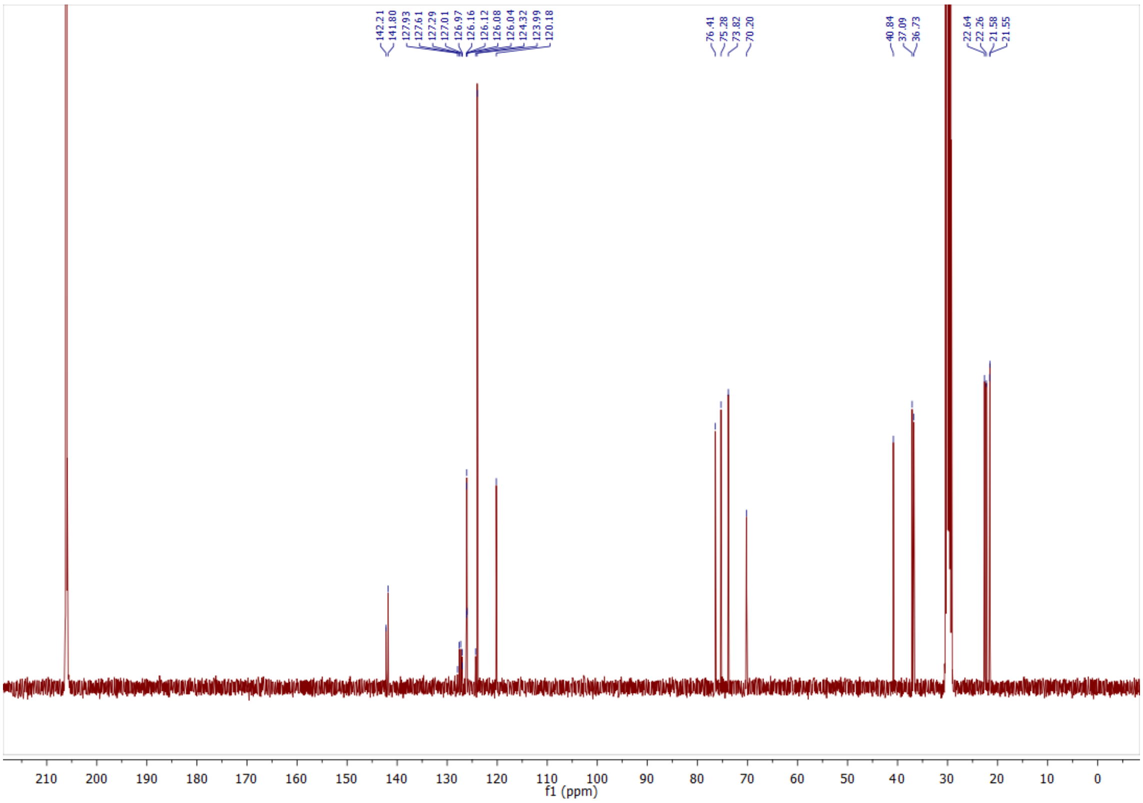

Compound 6oA: ^{1}H NMR (acetone-d_6, 400 MHz)

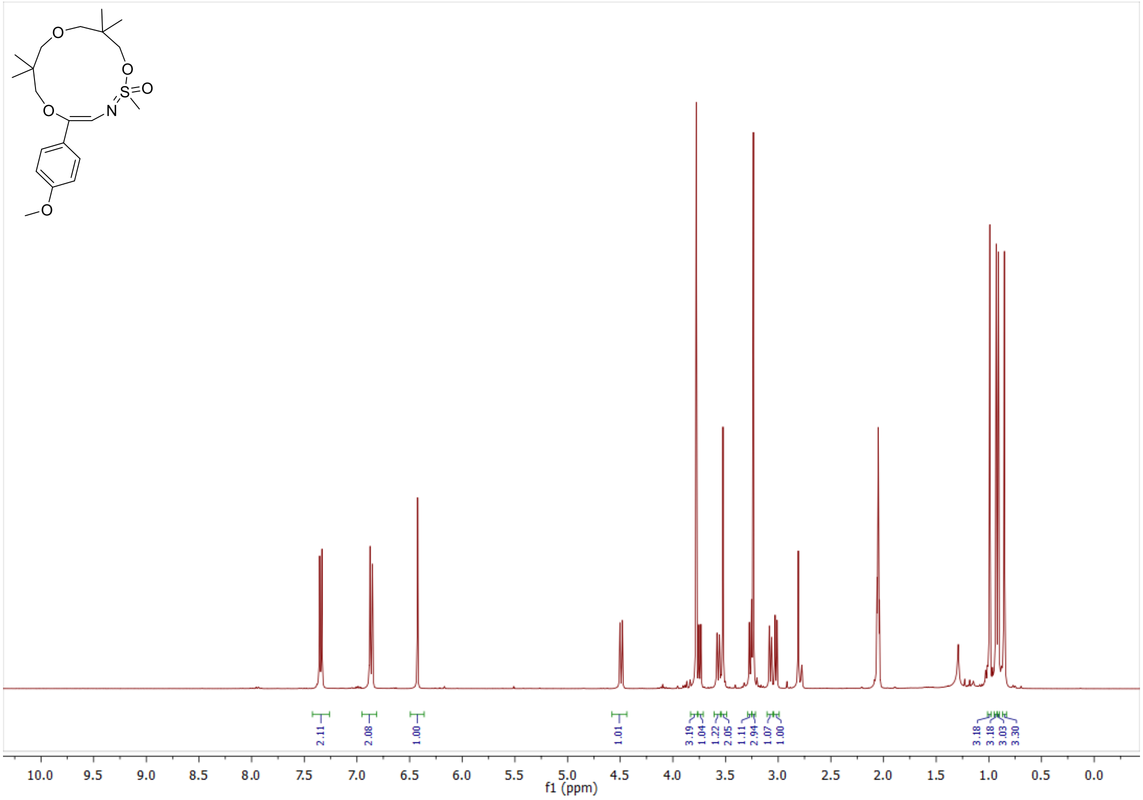

Compound 6oA: ^{13}C NMR (acetone-d_6, 100 MHz)

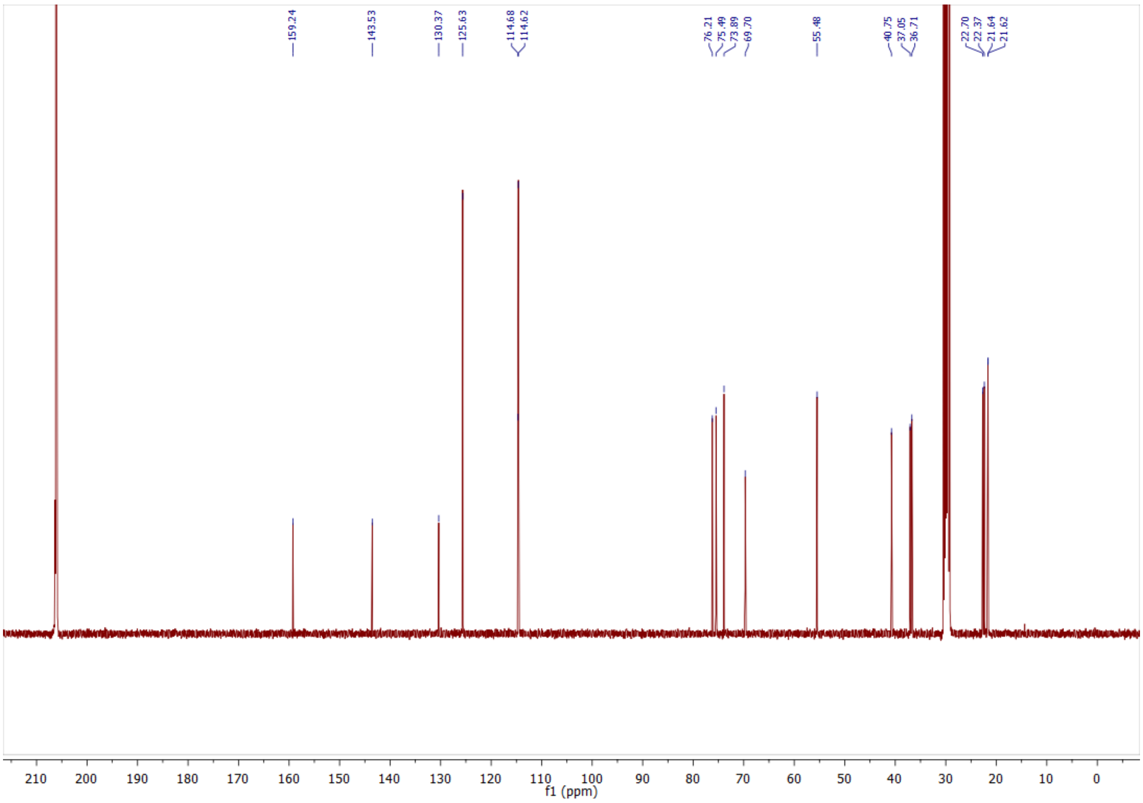

Compound 6aA: ^{1}H NMR (acetone-d_6, 400 MHz)

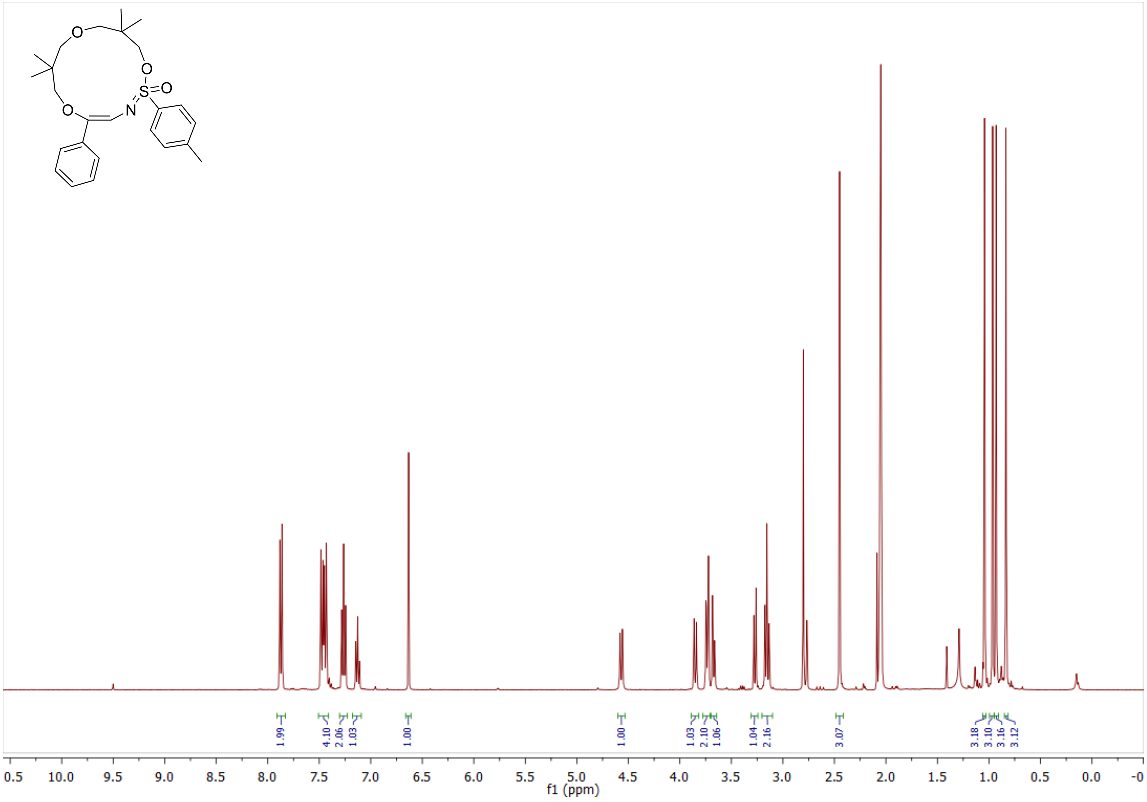

Compound 6aA: ^{13}C NMR (acetone-d_6, 100 MHz)

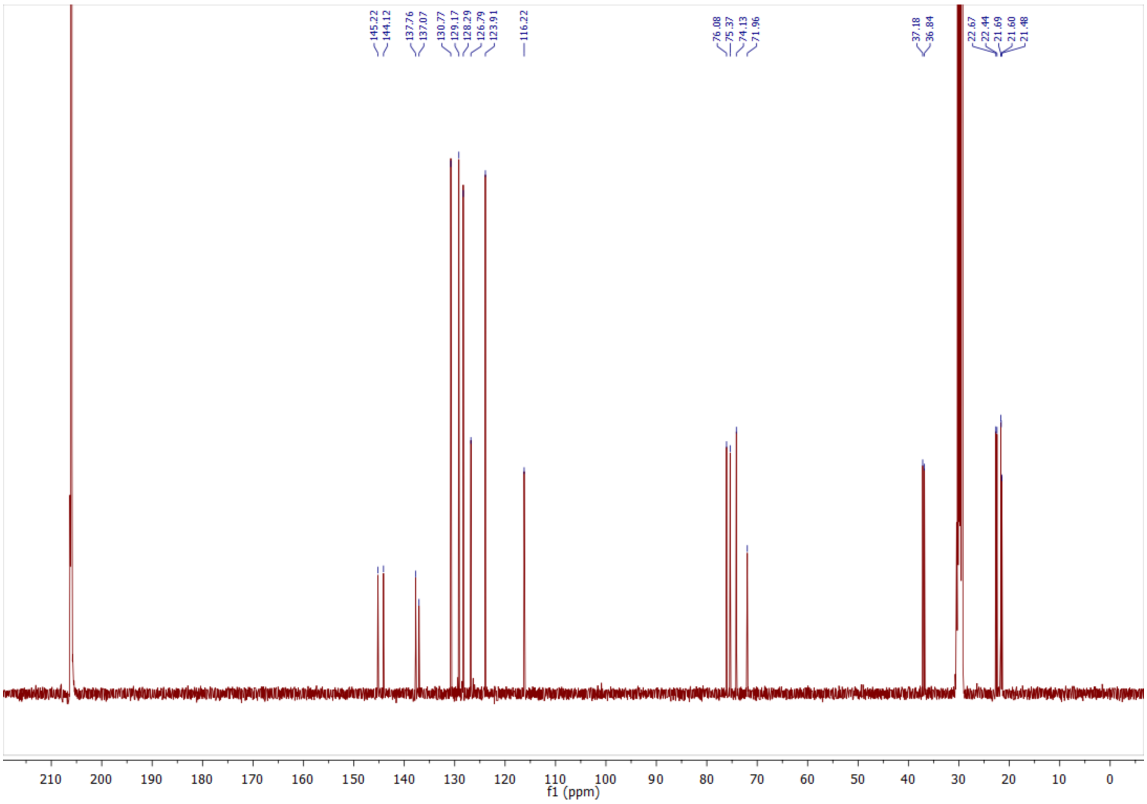

Compound 10aA: ^{1}H NMR (acetone-d_6, 400 MHz)

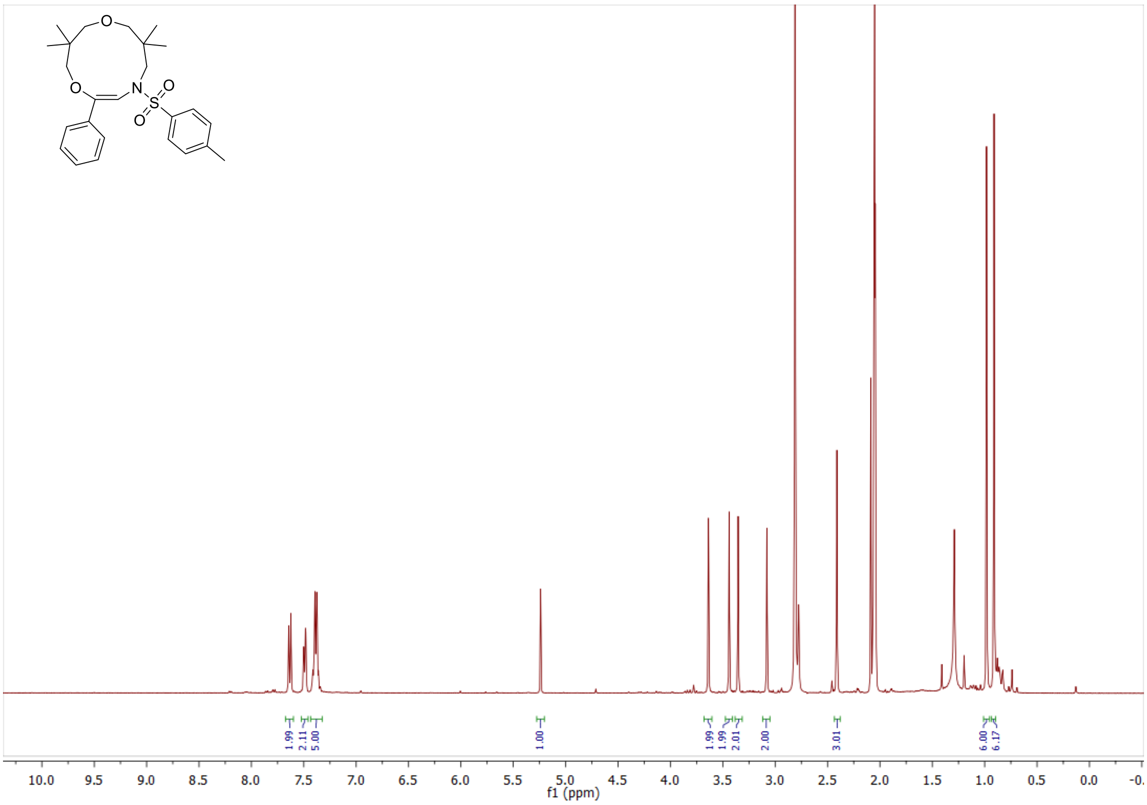

Compound 10aA: ^{13}C NMR (acetone-d_6, 100 MHz)

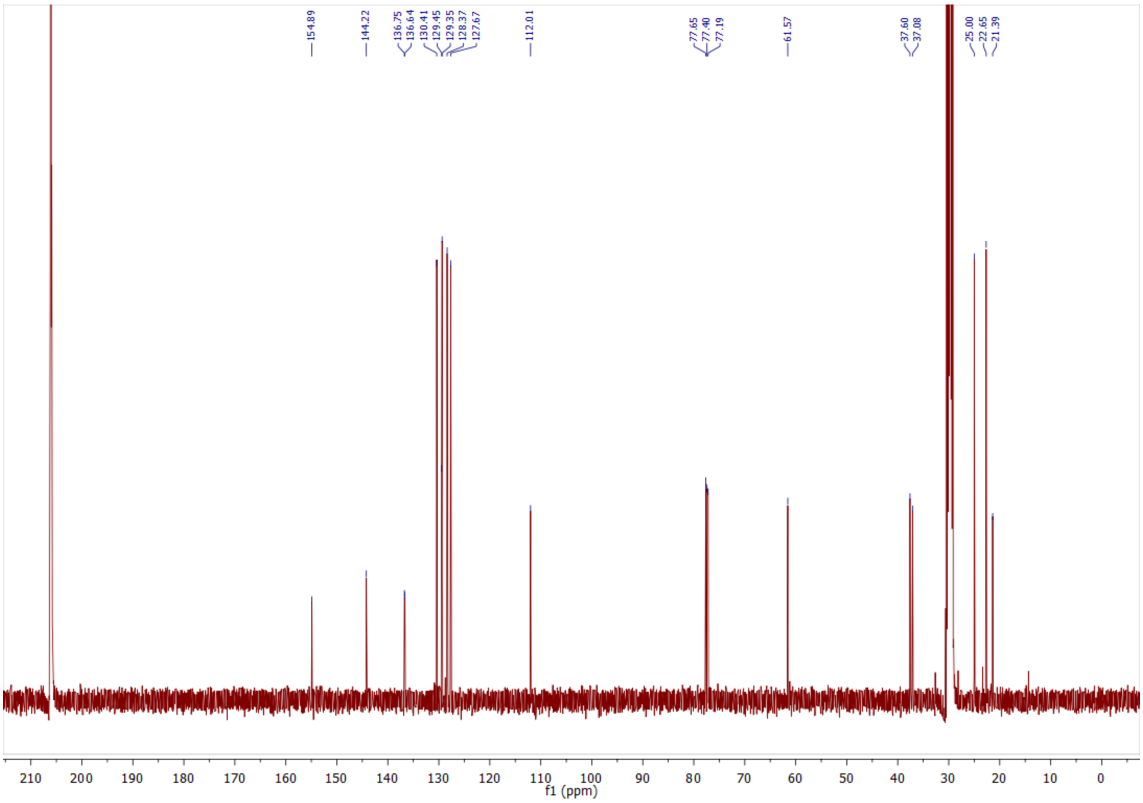

Compound 16aA: ^{1}H NMR ($CDCl_3$, 400 MHz)

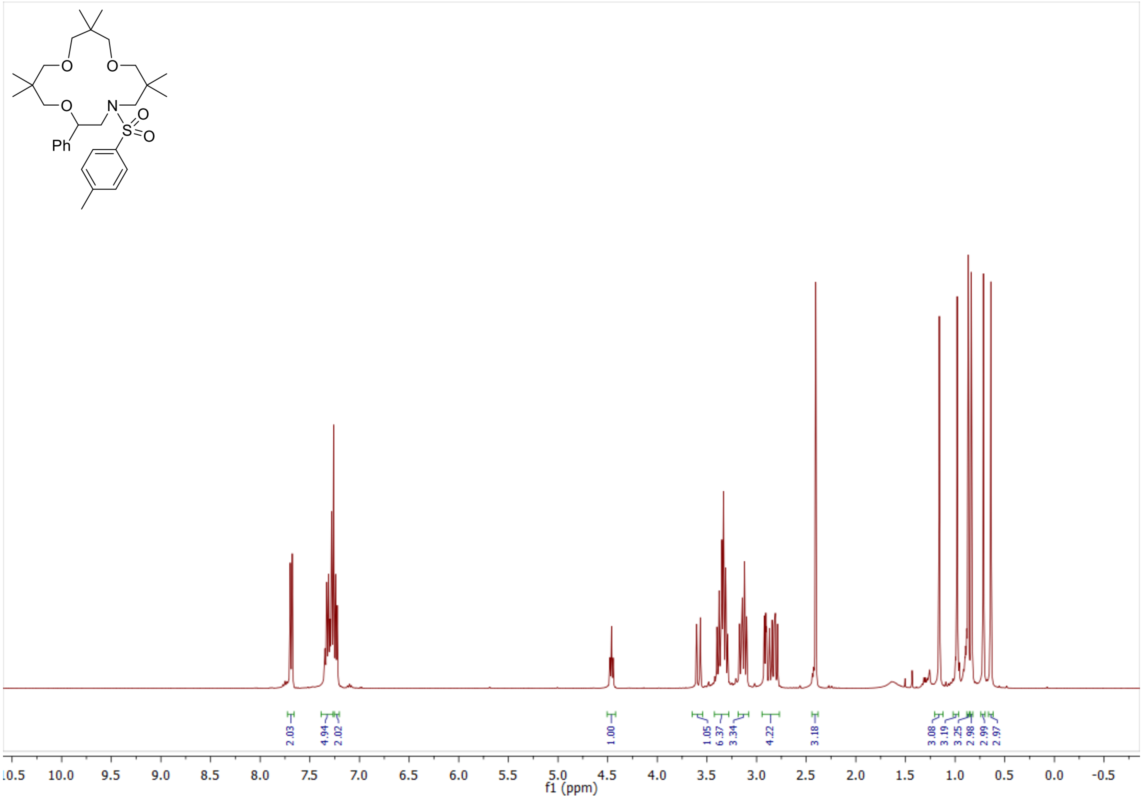

Compound 16aA: ^{13}C NMR ($CDCl_3$, 100 MHz)

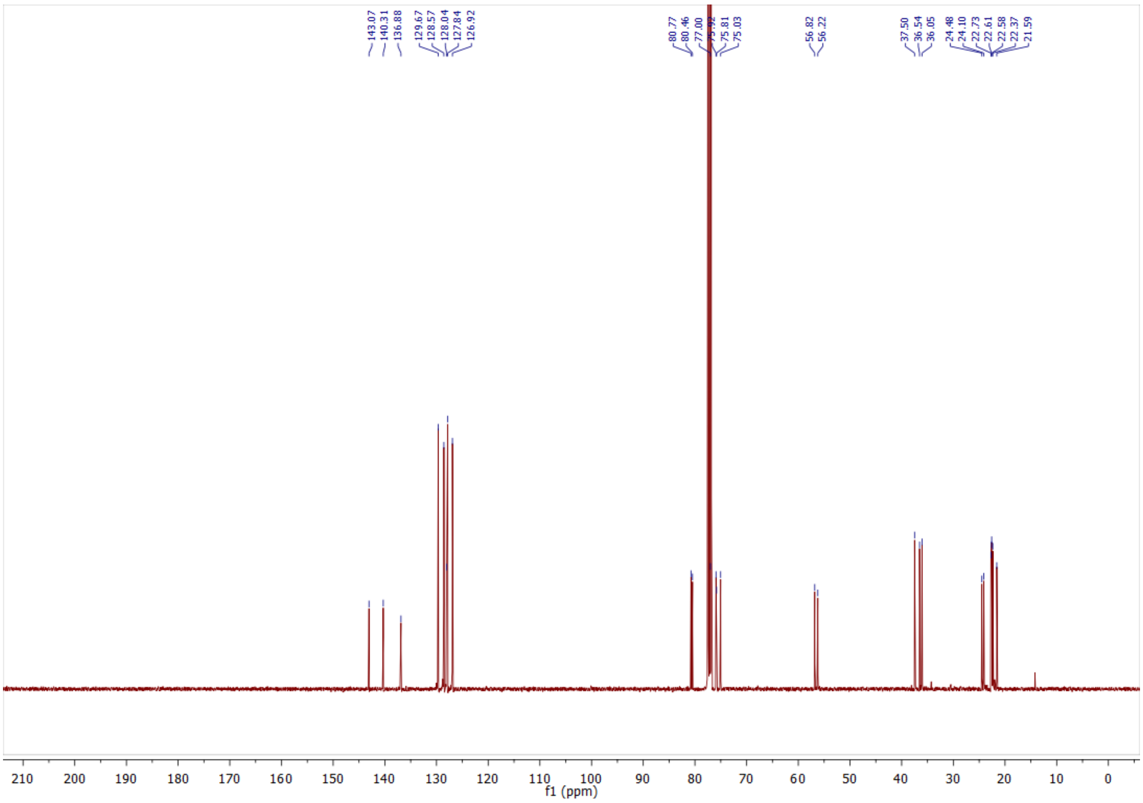

Compound 16hA: ^{1}H NMR ($CDCl_3$, 400 MHz)

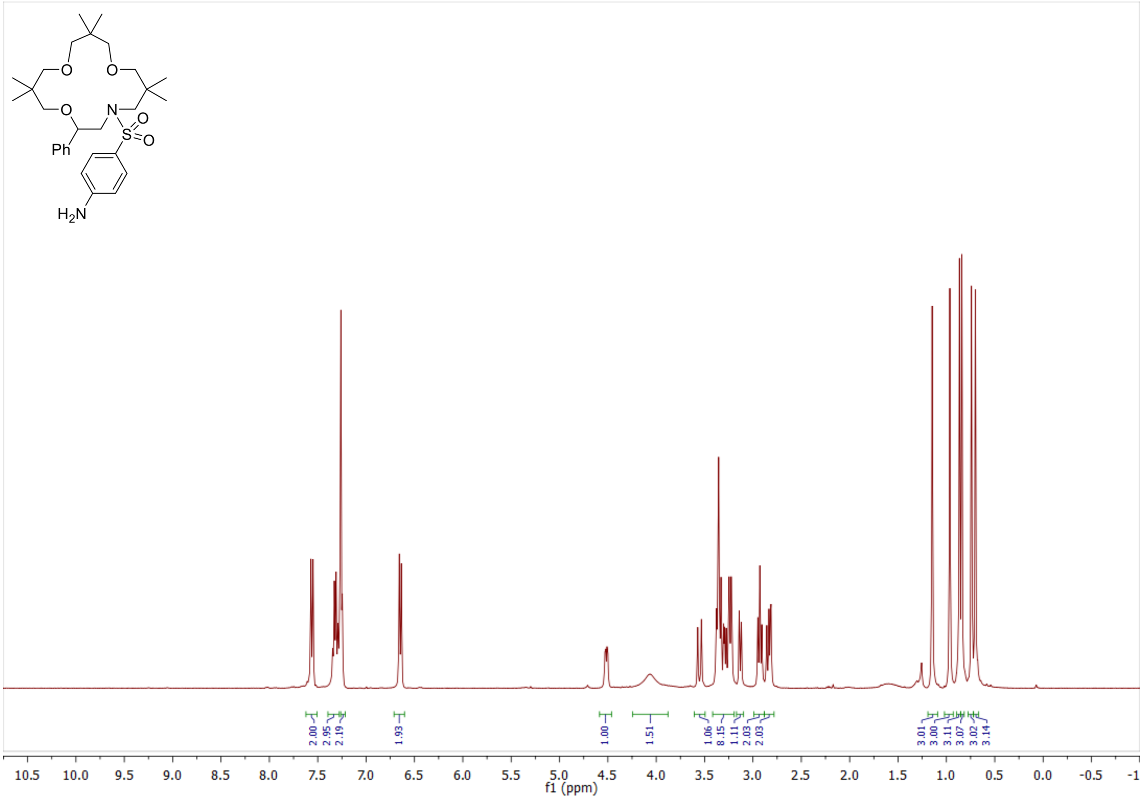

Compound 16hA: ^{13}C NMR ($CDCl_3$, 100 MHz)

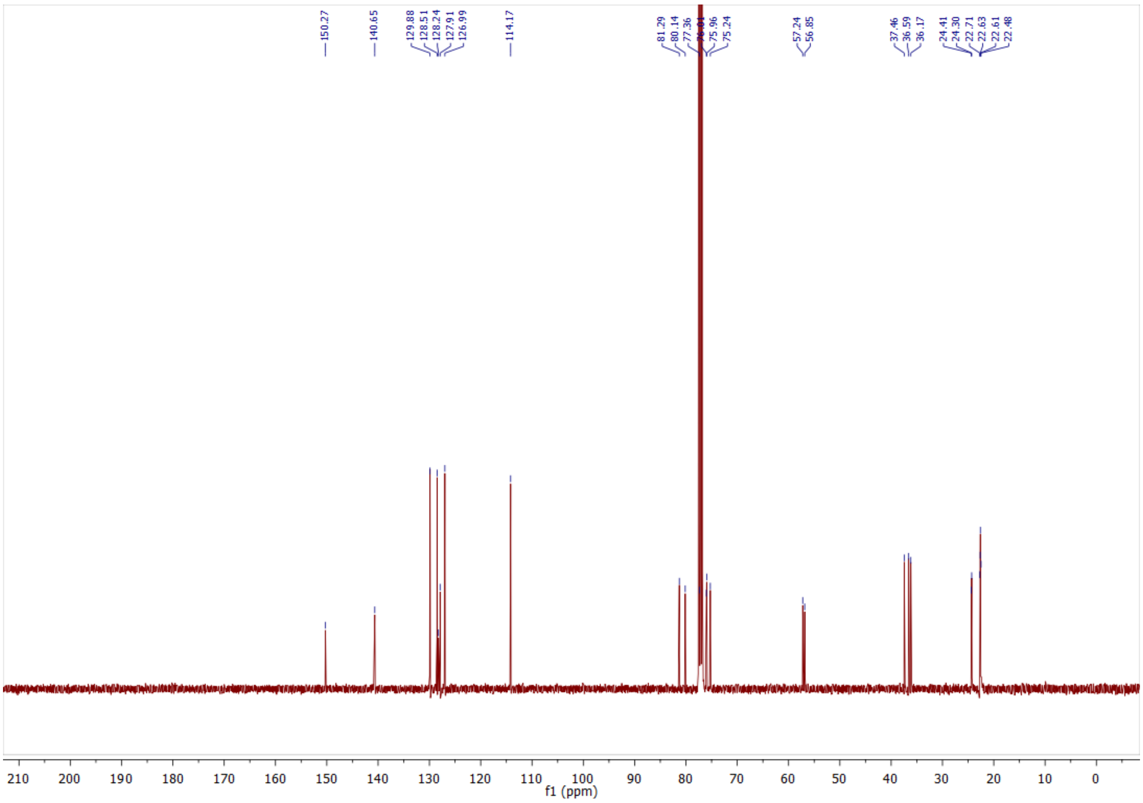

Compound 16lA: ^{1}H NMR (acetone-d_6, 400 MHz)

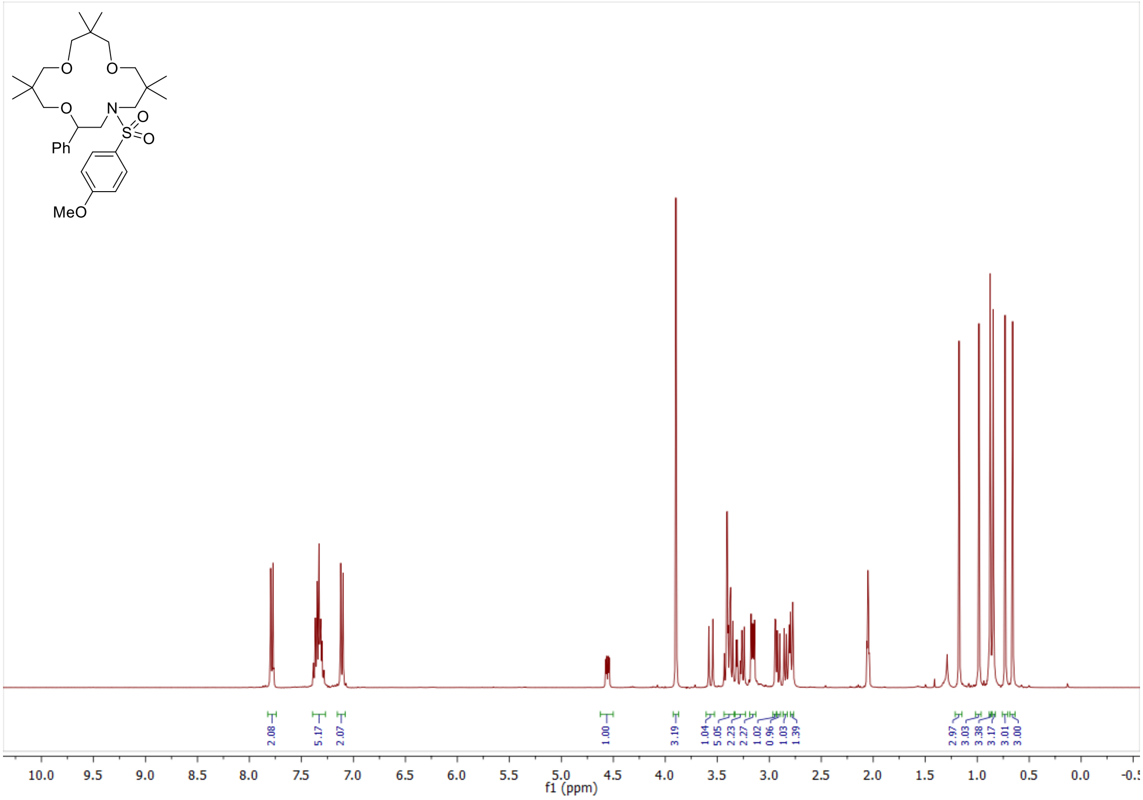

Compound 16lA: ^{13}C NMR (acetone-d_6, 100 MHz)

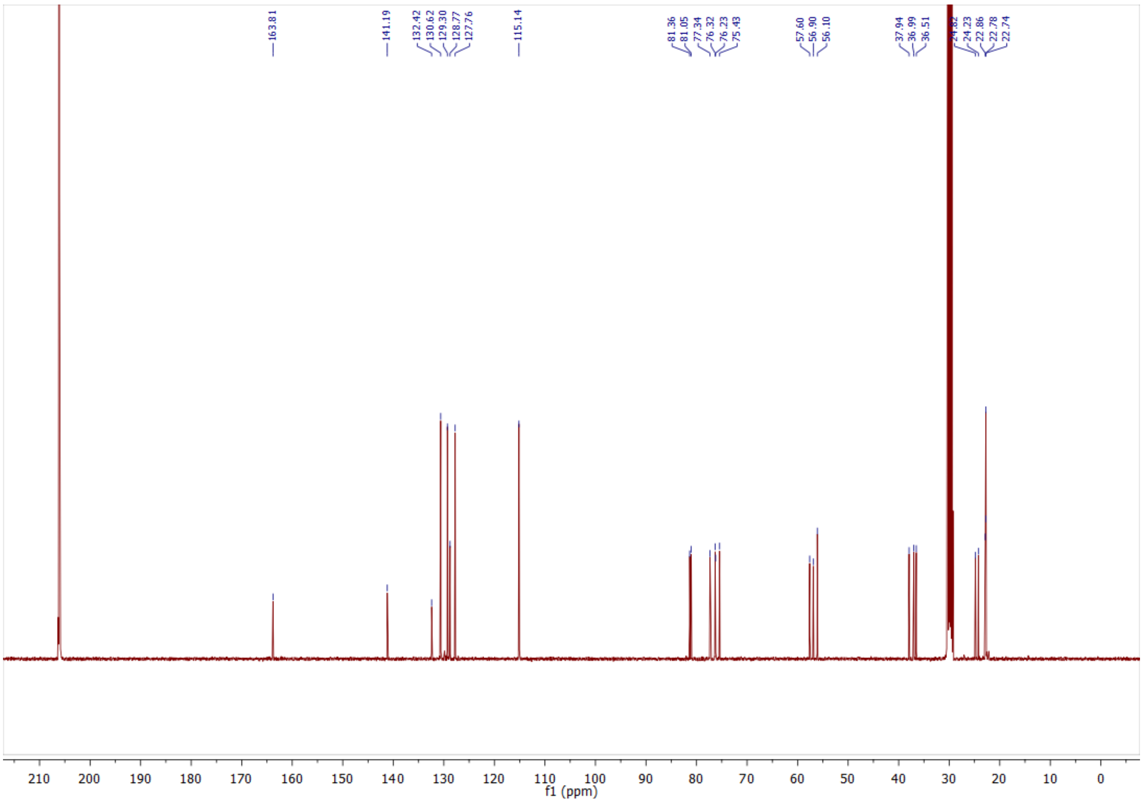

Compound 16mA: ^{1}H NMR ($CDCl_3$, 400 MHz)

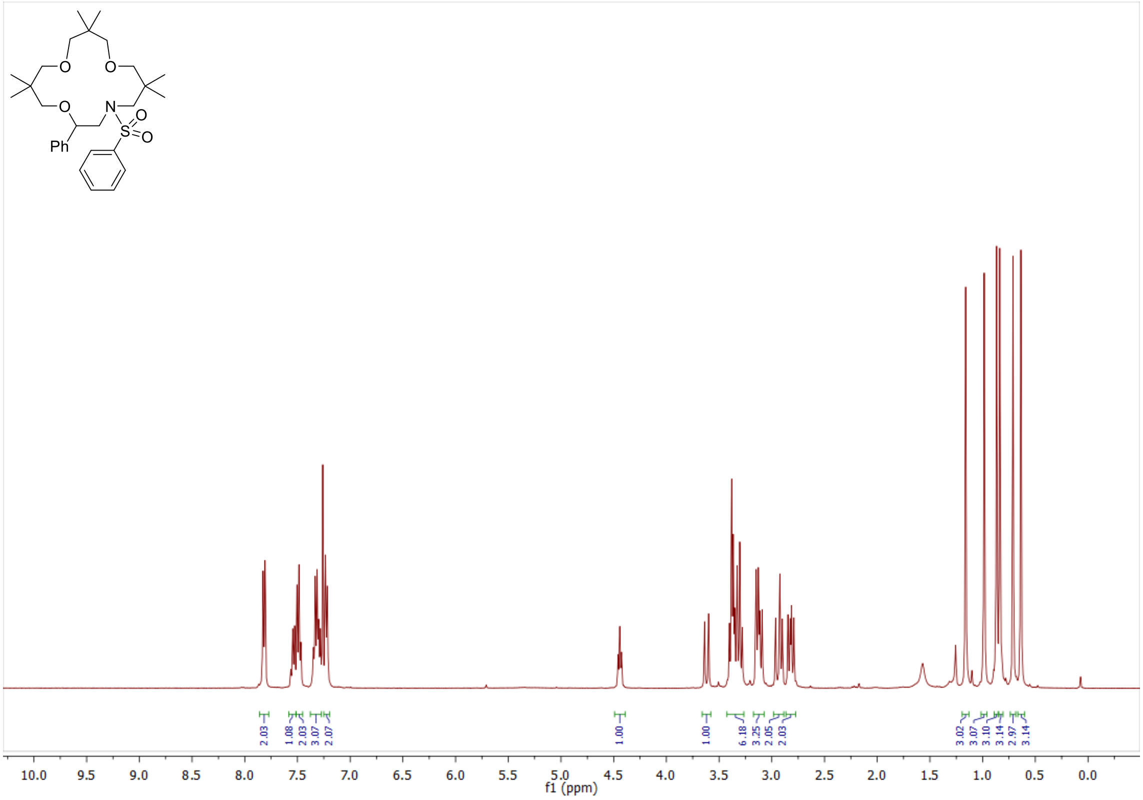

Compound 16mA: ^{13}C NMR ($CDCl_3$, 100 MHz)

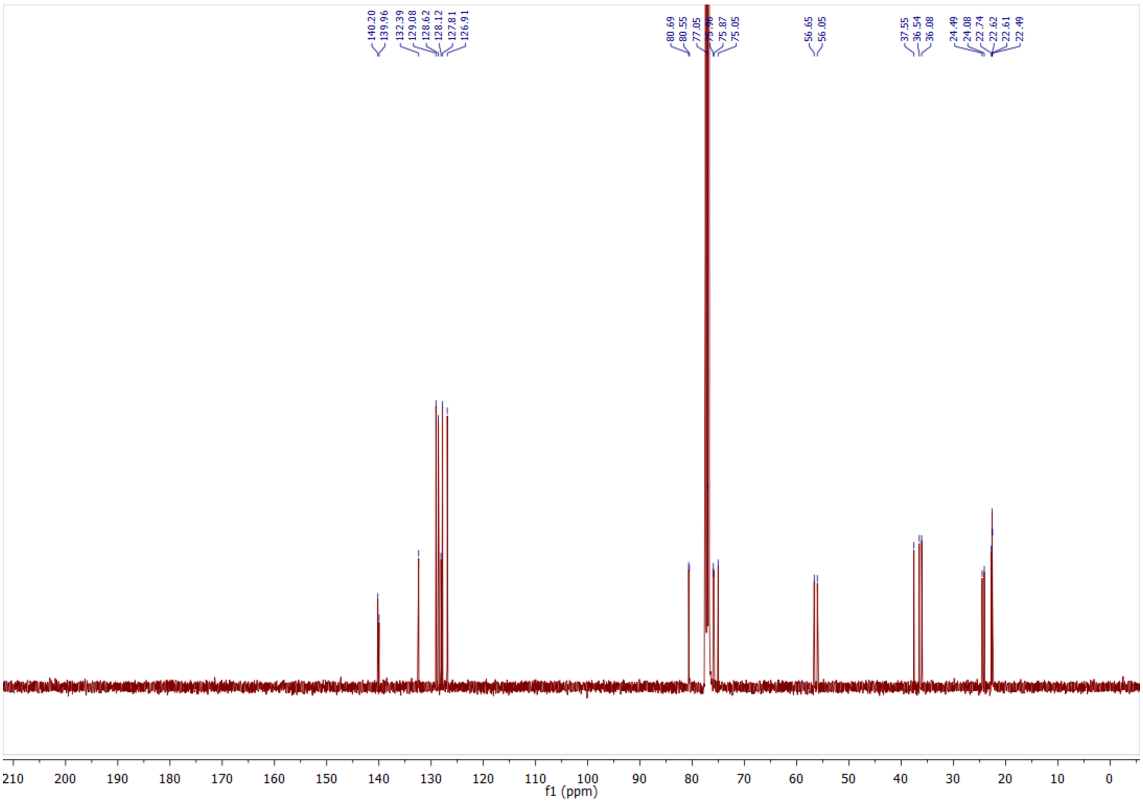

Compound 16aB: ^{1}H NMR ($CDCl_3$, 400 MHz)

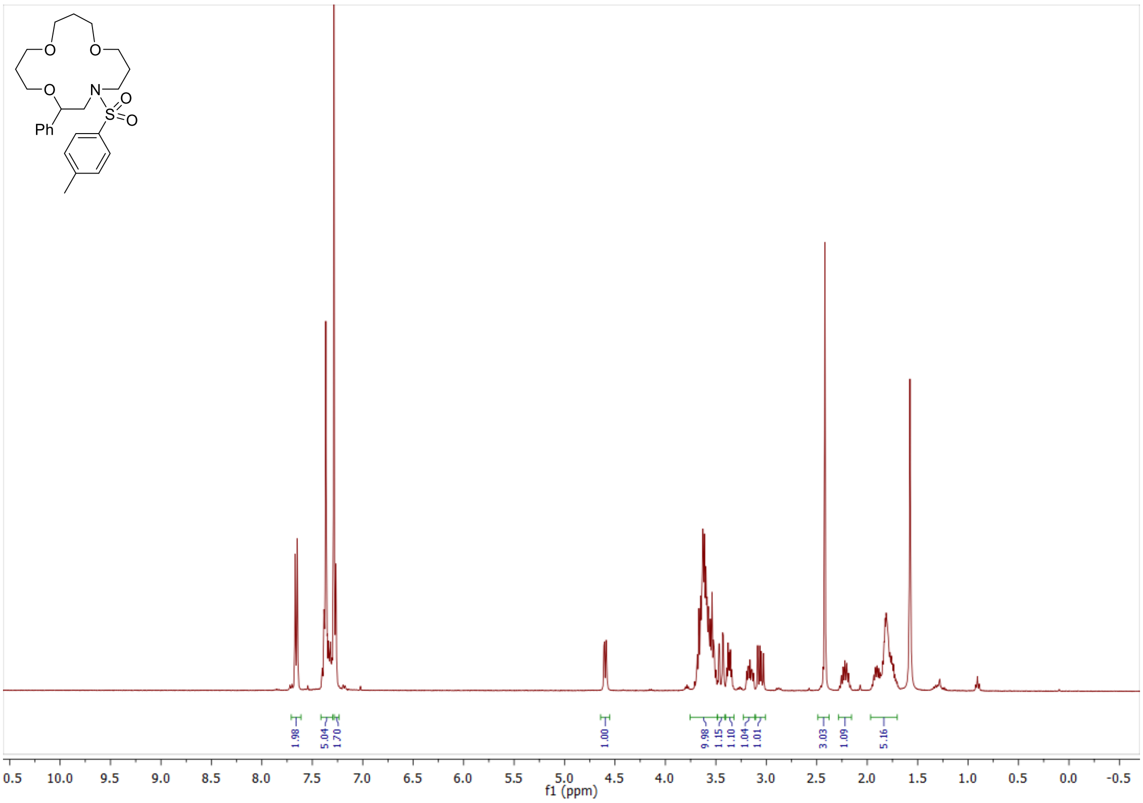

Compound 16aB: ^{13}C NMR ($CDCl_3$, 100 MHz)

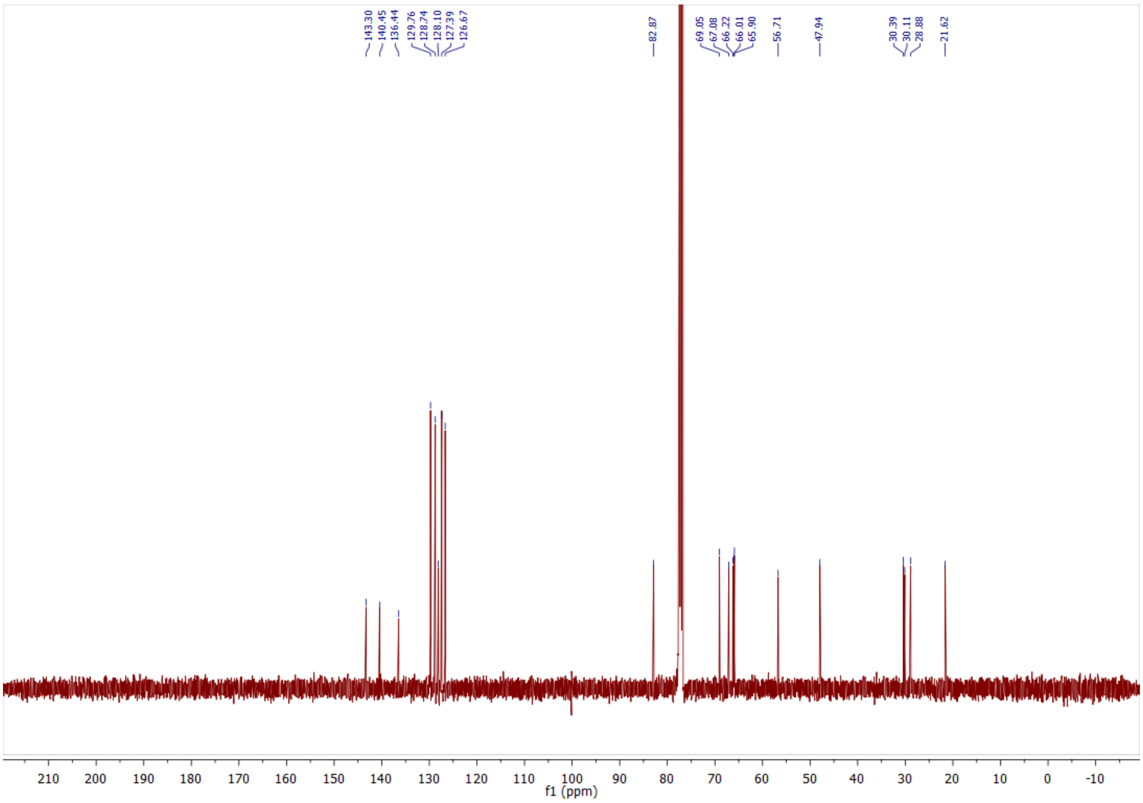

Compound 16hB: ^{1}H NMR (acetone-d_6, 400 MHz)

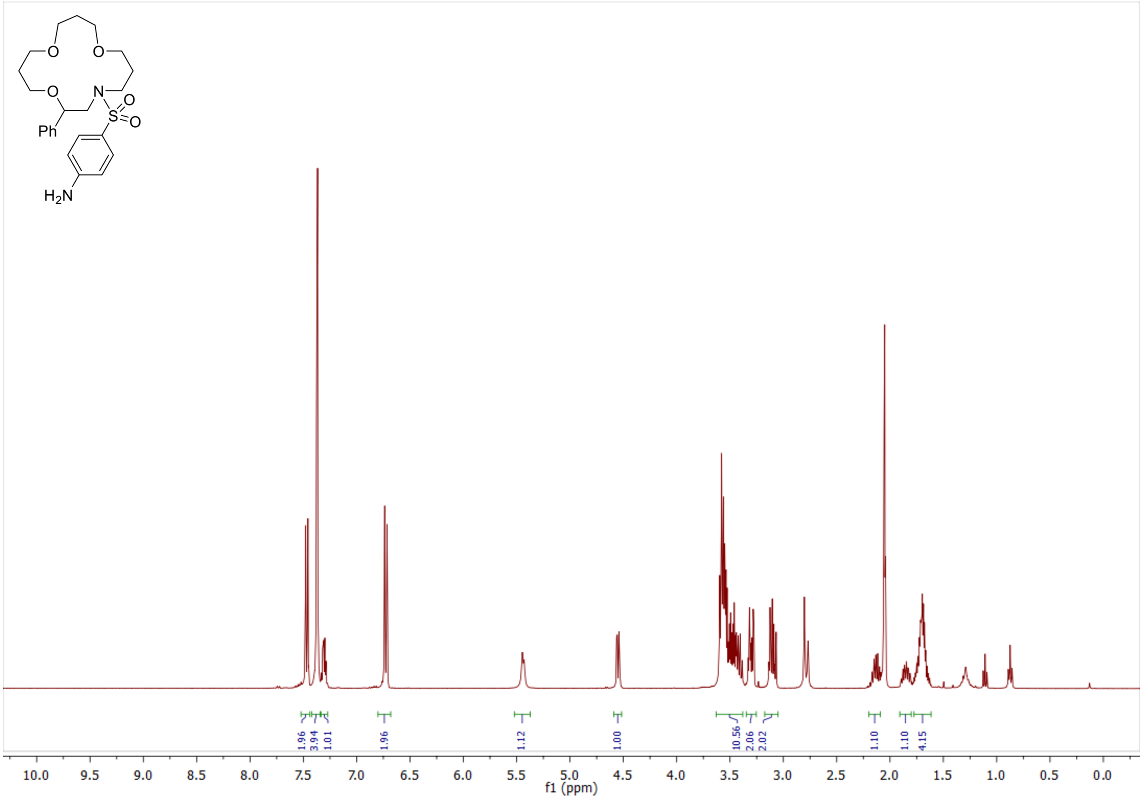

Compound 16hB: ^{13}C NMR (acetone-d_6, 100 MHz)

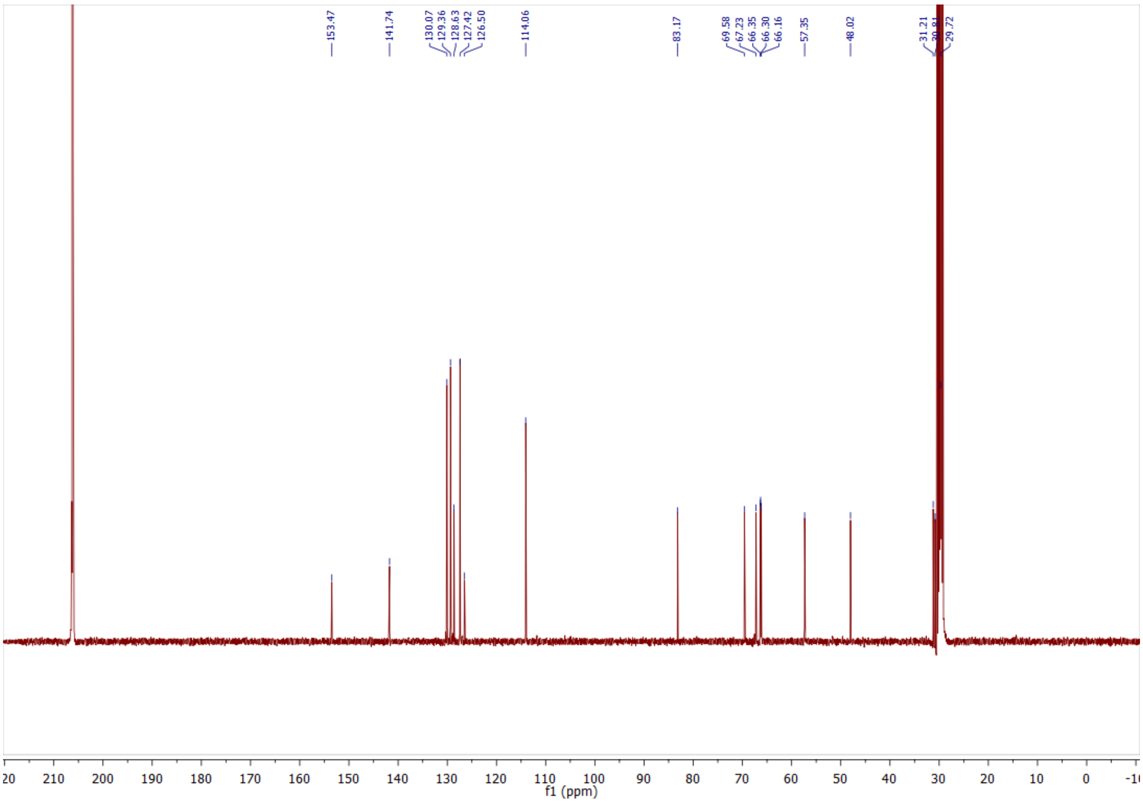

Compound 16lB: ^{1}H NMR (acetone-d_6, 400 MHz)

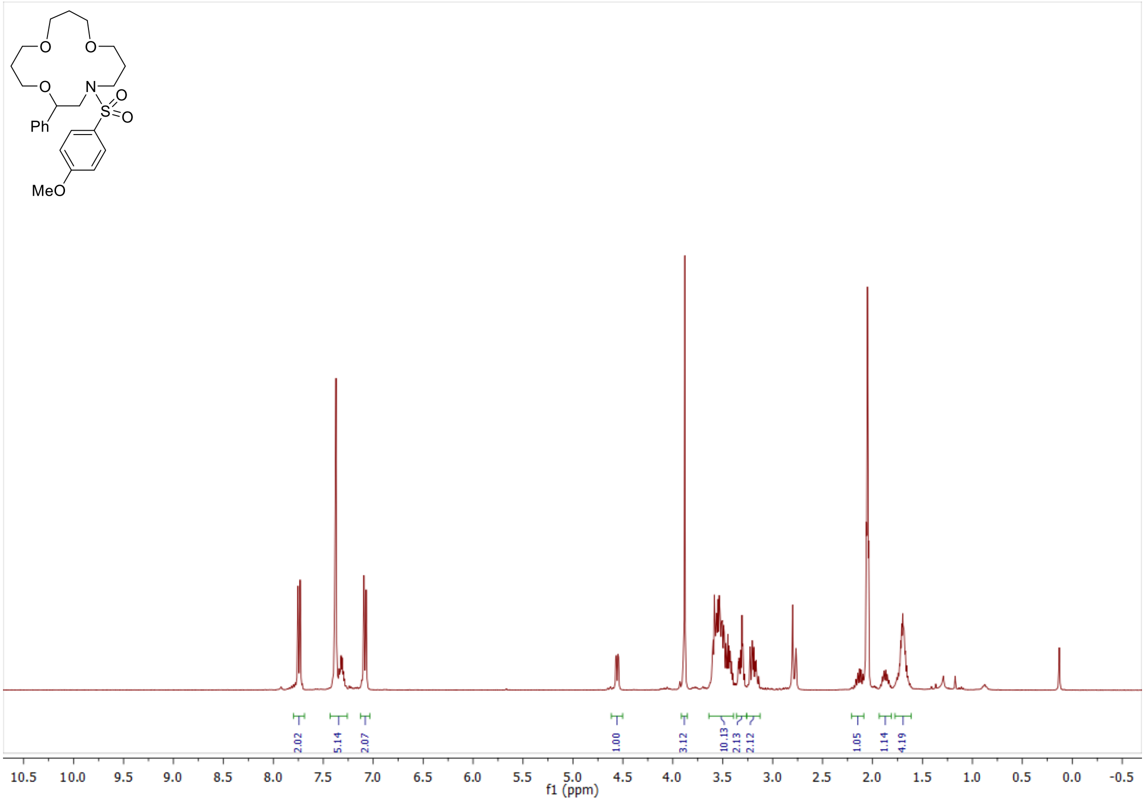

Compound 16lB: ^{13}C NMR (acetone-d_6, 100 MHz)

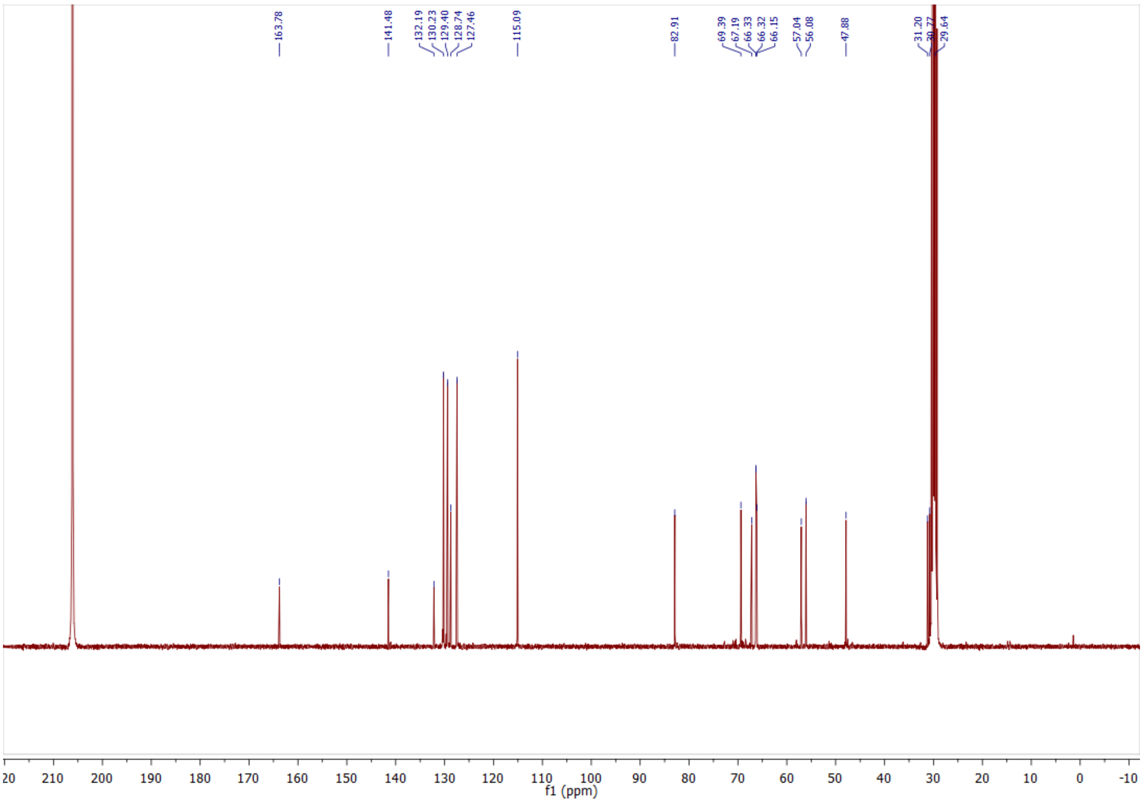

Compound 16mB: ^{1}H NMR (acetone-d_6, 400 MHz)

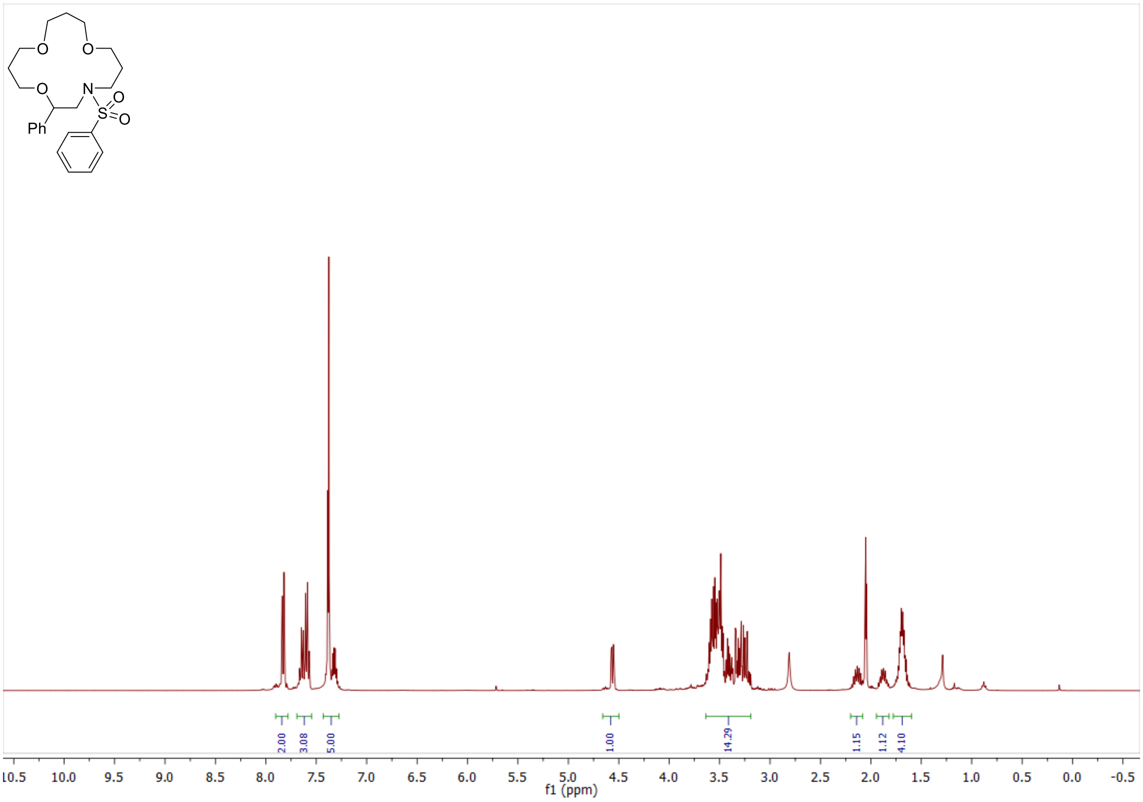

Compound 16mB: ^{13}C NMR (acetone-d_6, 100 MHz)

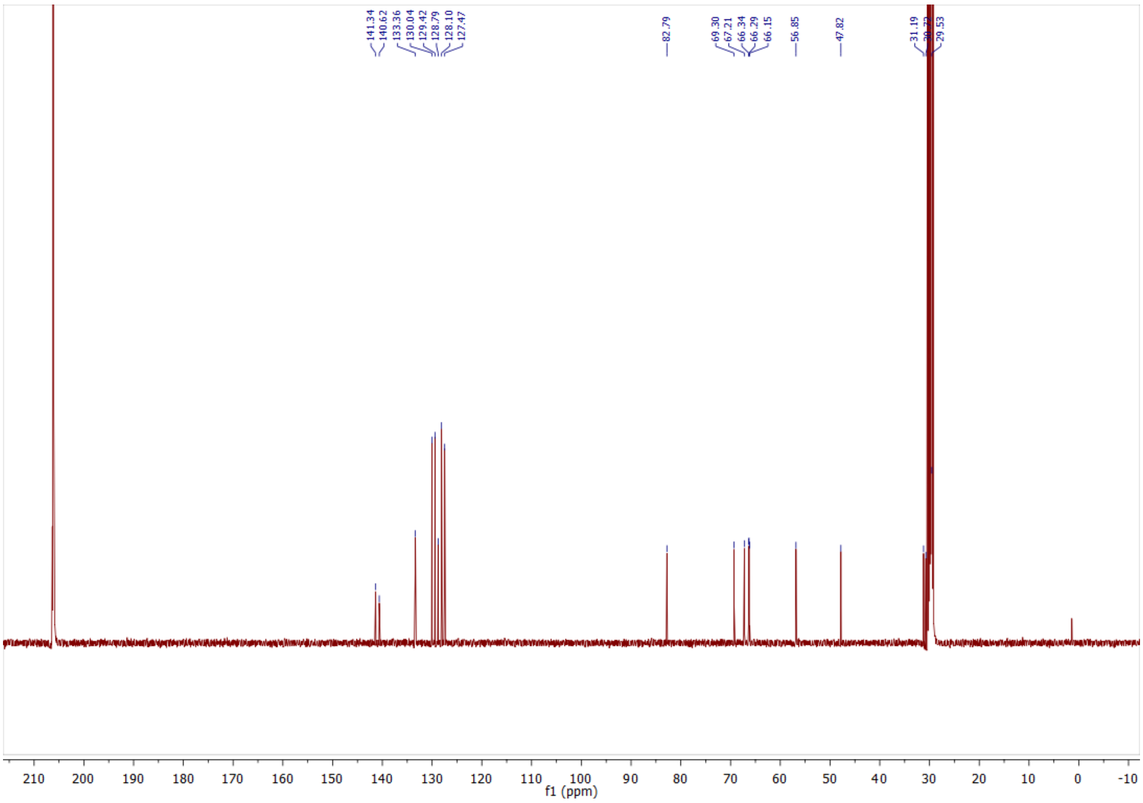

Compound 17aA: ^{1}H NMR ($CDCl_3$, 400 MHz)

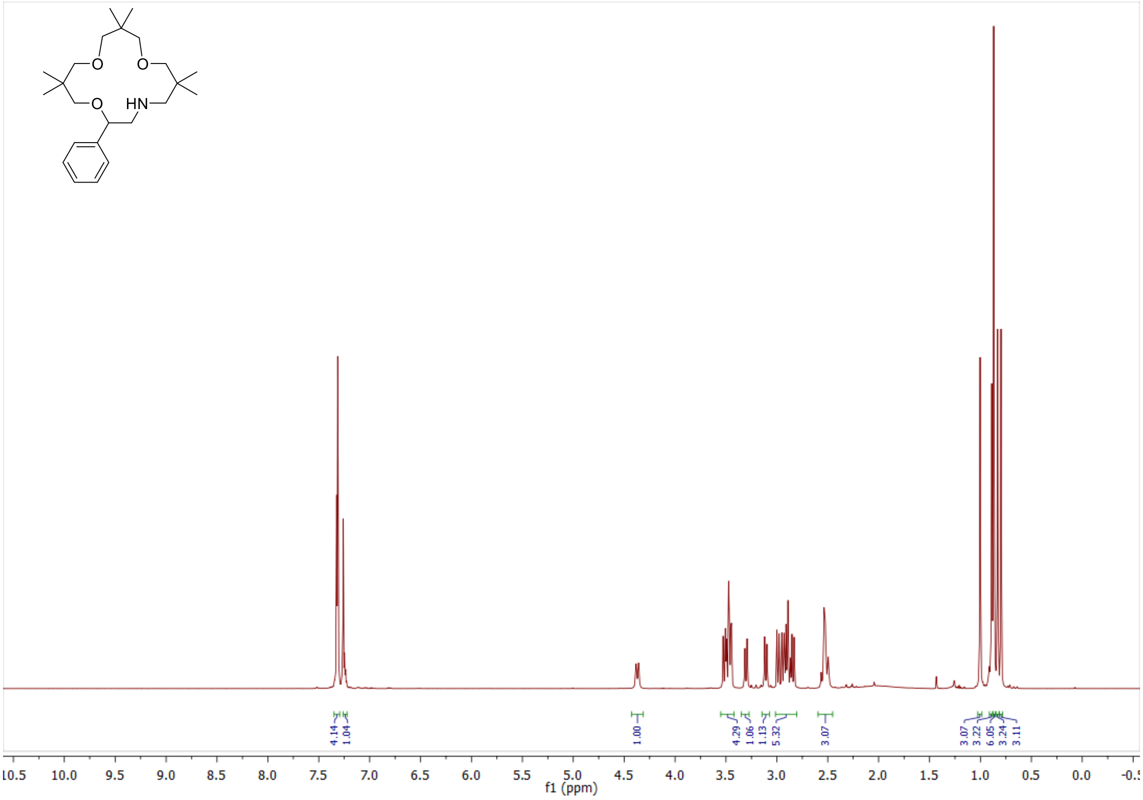

Compound 17aA: ^{13}C NMR ($CDCl_3$, 100 MHz)

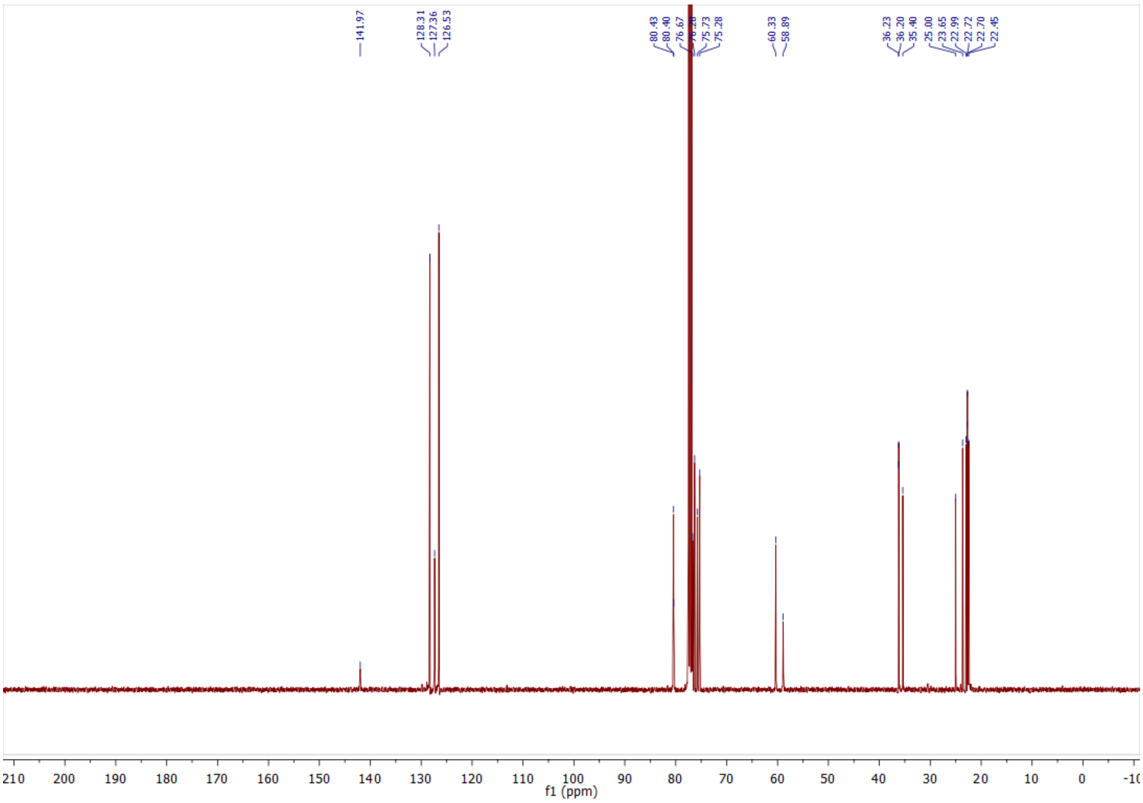

Compound 17aB: ^{1}H NMR ($CDCl_3$, 400 MHz)

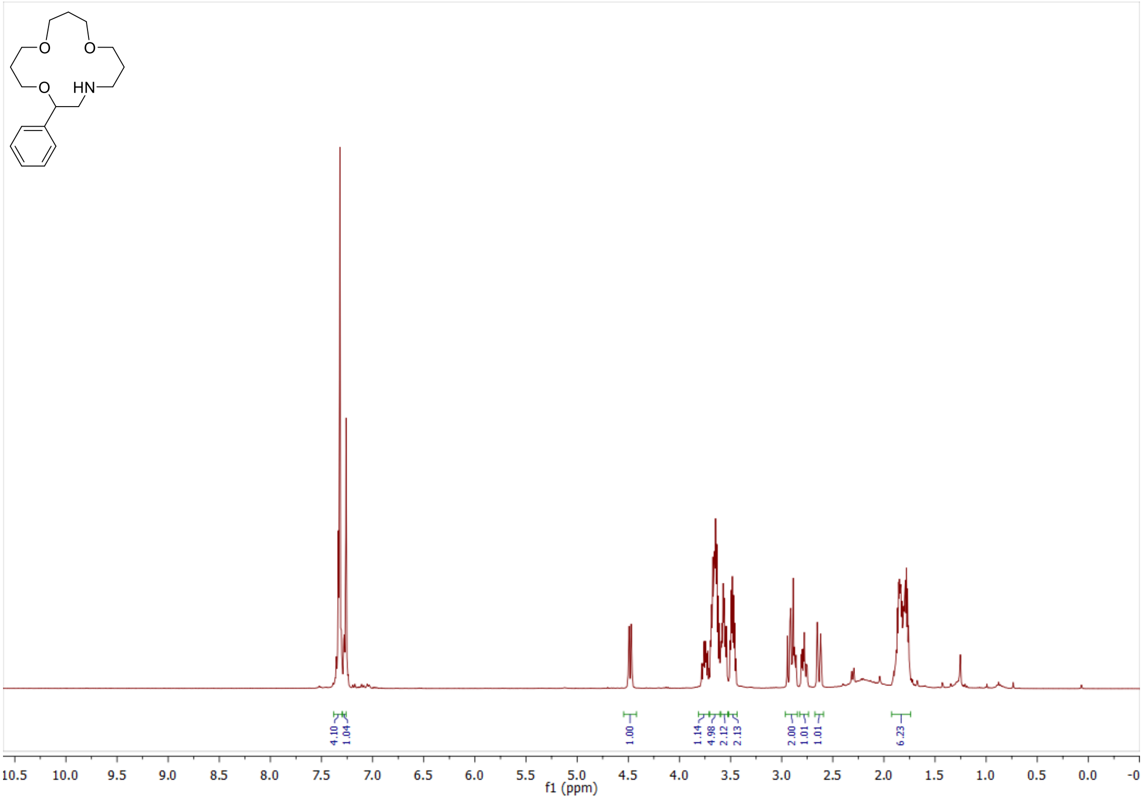

Compound 17aB: ^{13}C NMR ($CDCl_3$, 100 MHz)

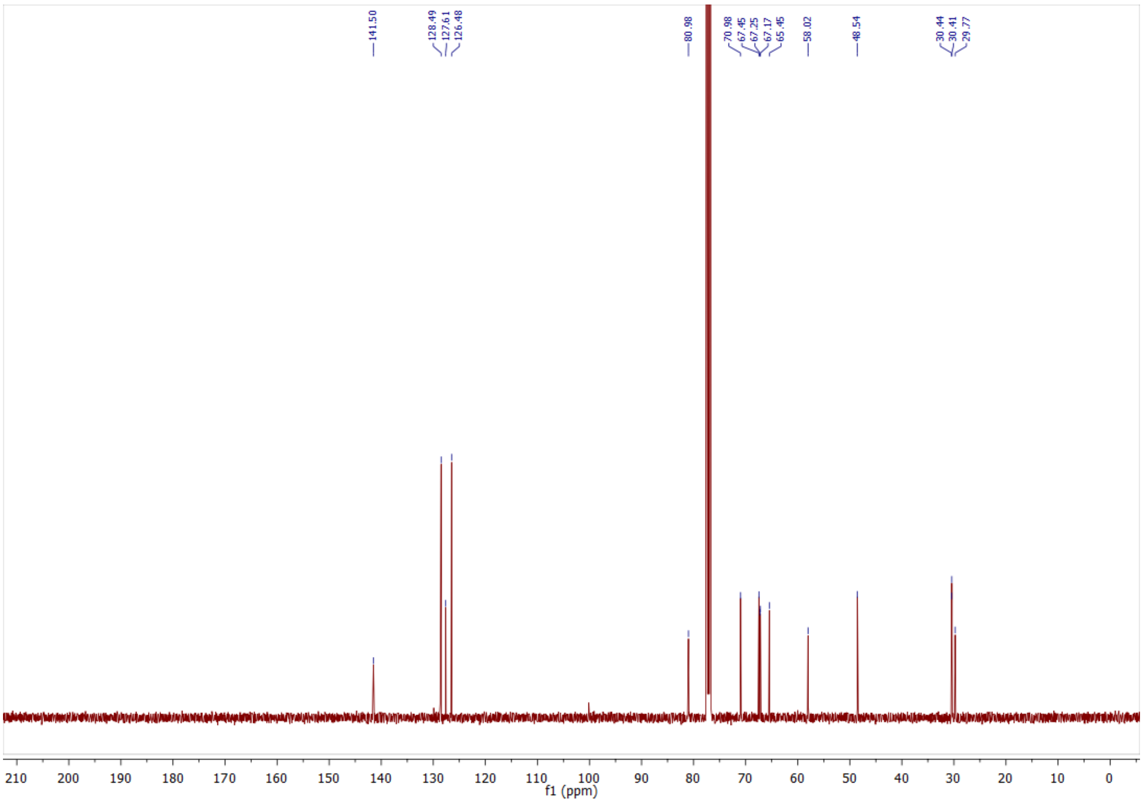

Compound 18: ^{1}H NMR ($CDCl_3$, 400 MHz)

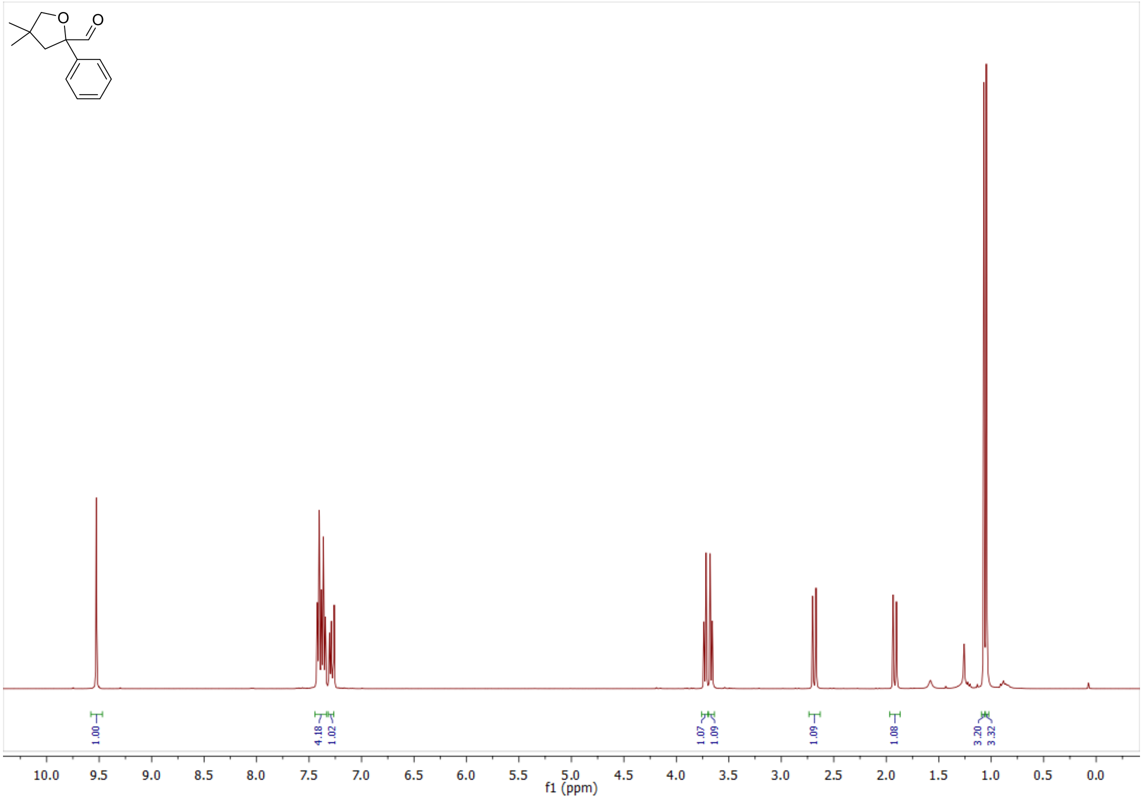

Compound 18: ^{13}C NMR ($CDCl_3$, 400 MHz)

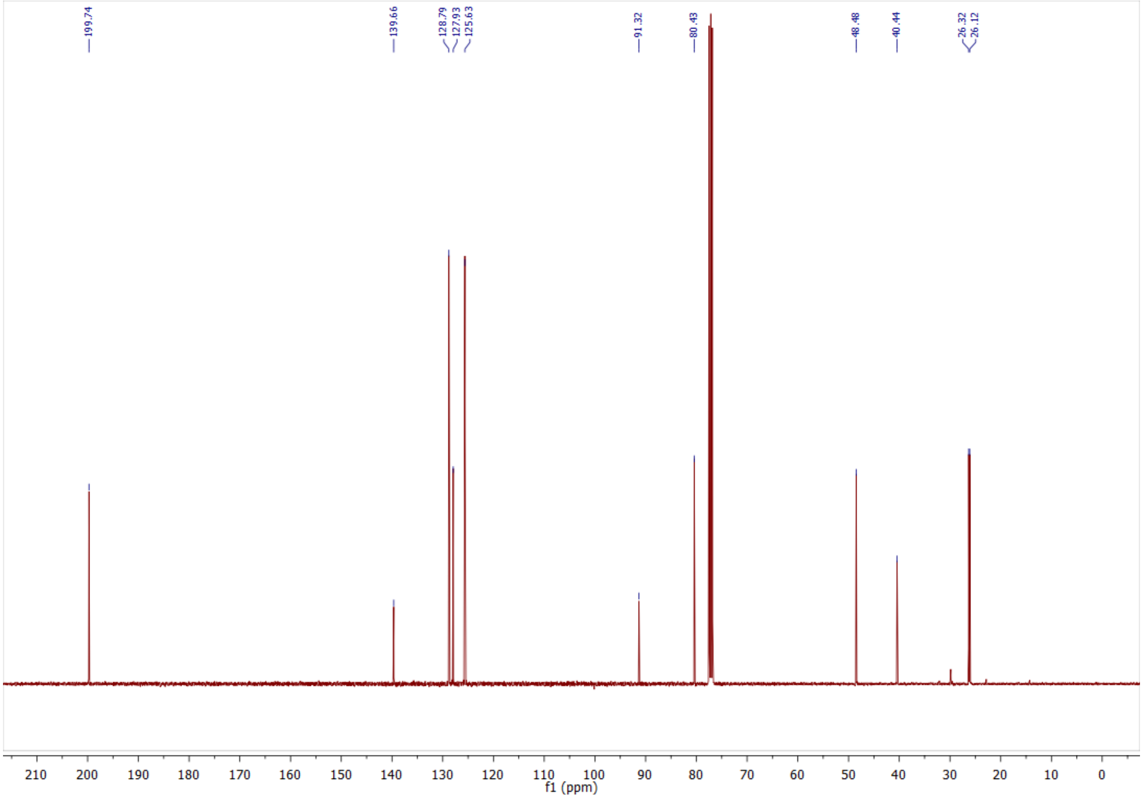

Compound 19: ^{1}H NMR ($CDCl_3$, 400 MHz)

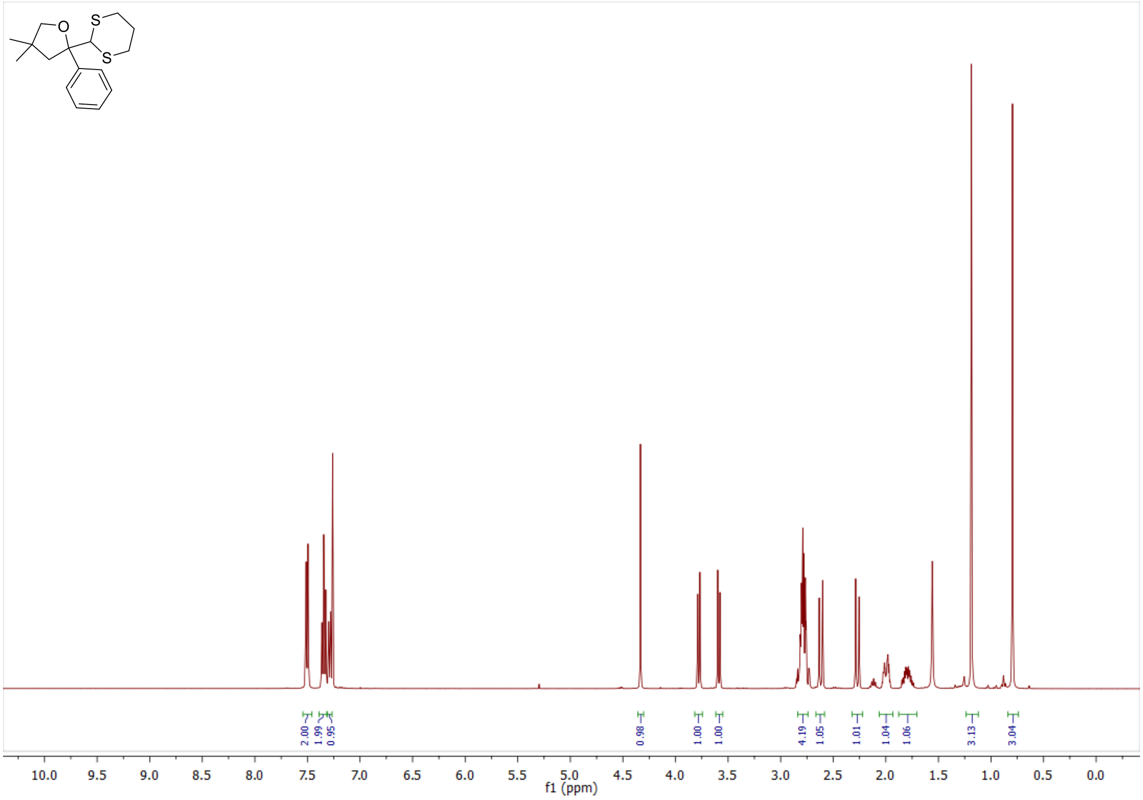

Compound 19: ^{13}C NMR ($CDCl_3$, 400 MHz)

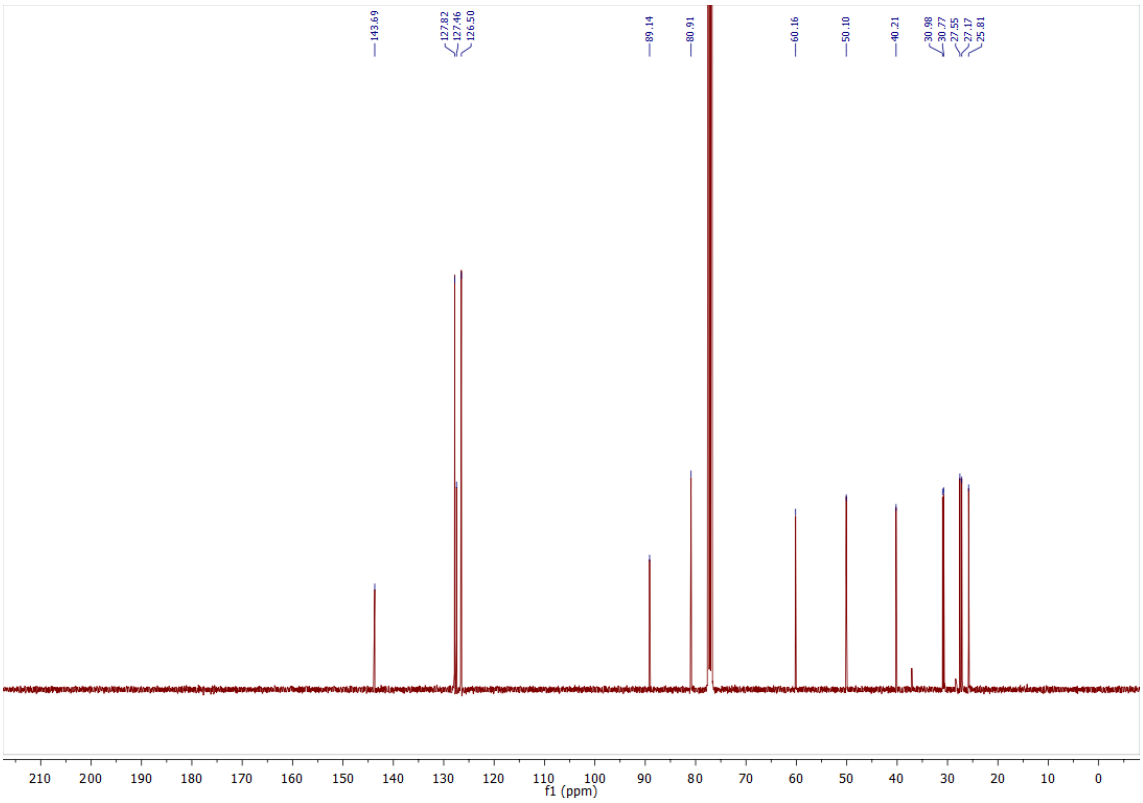

Compound 20: ^{1}H NMR ($CDCl_3$, 400 MHz)

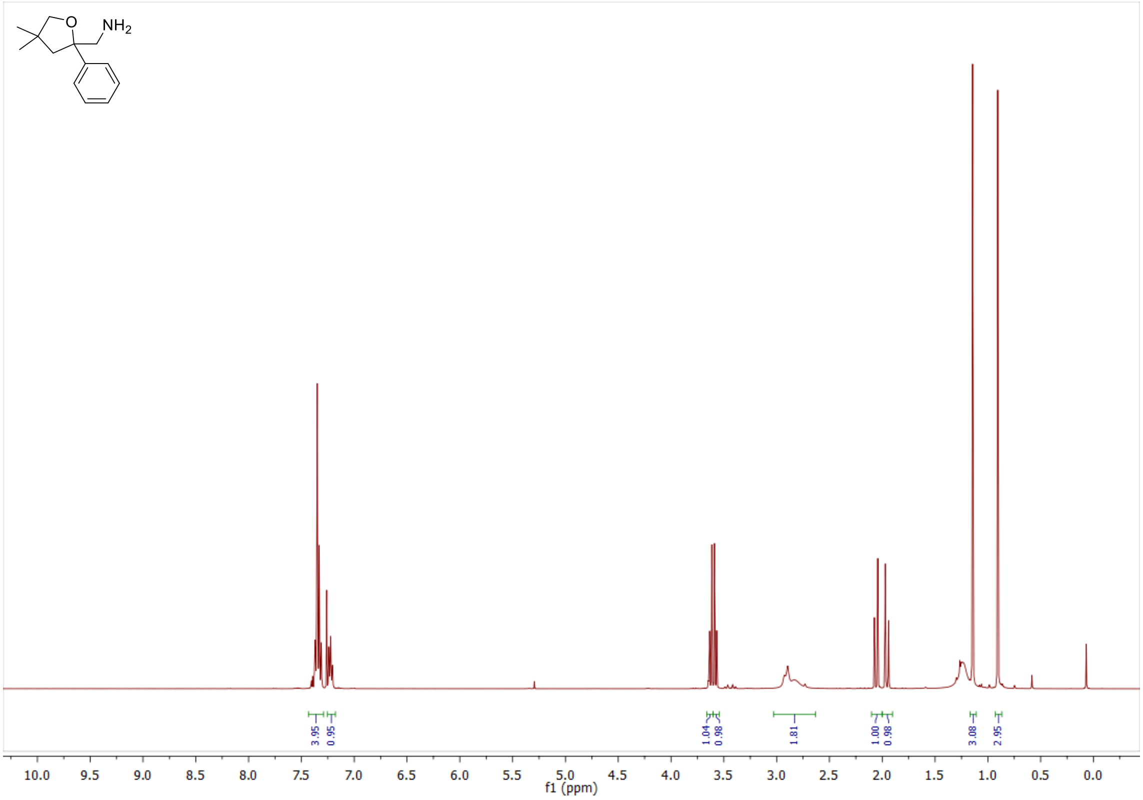

Compound 20: ^{13}C NMR ($CDCl_3$, 400 MHz)

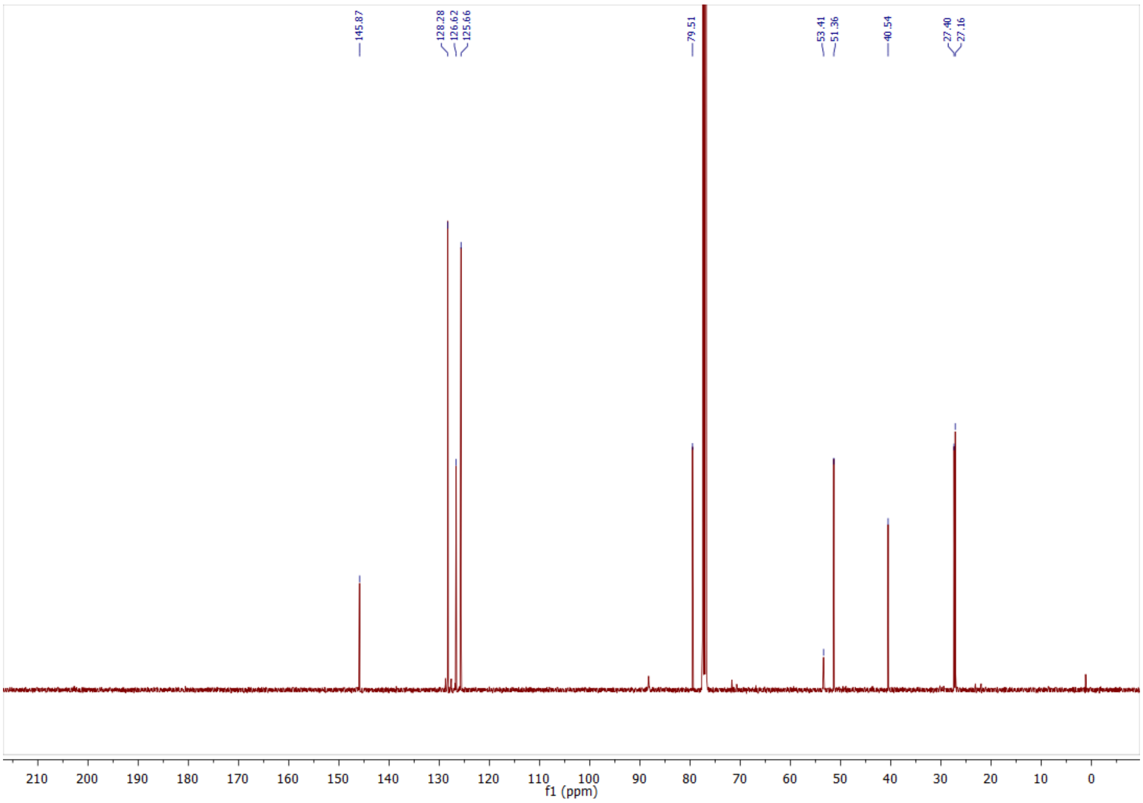

Compound 7: ^{1}H NMR ($CDCl_3$, 400 MHz)

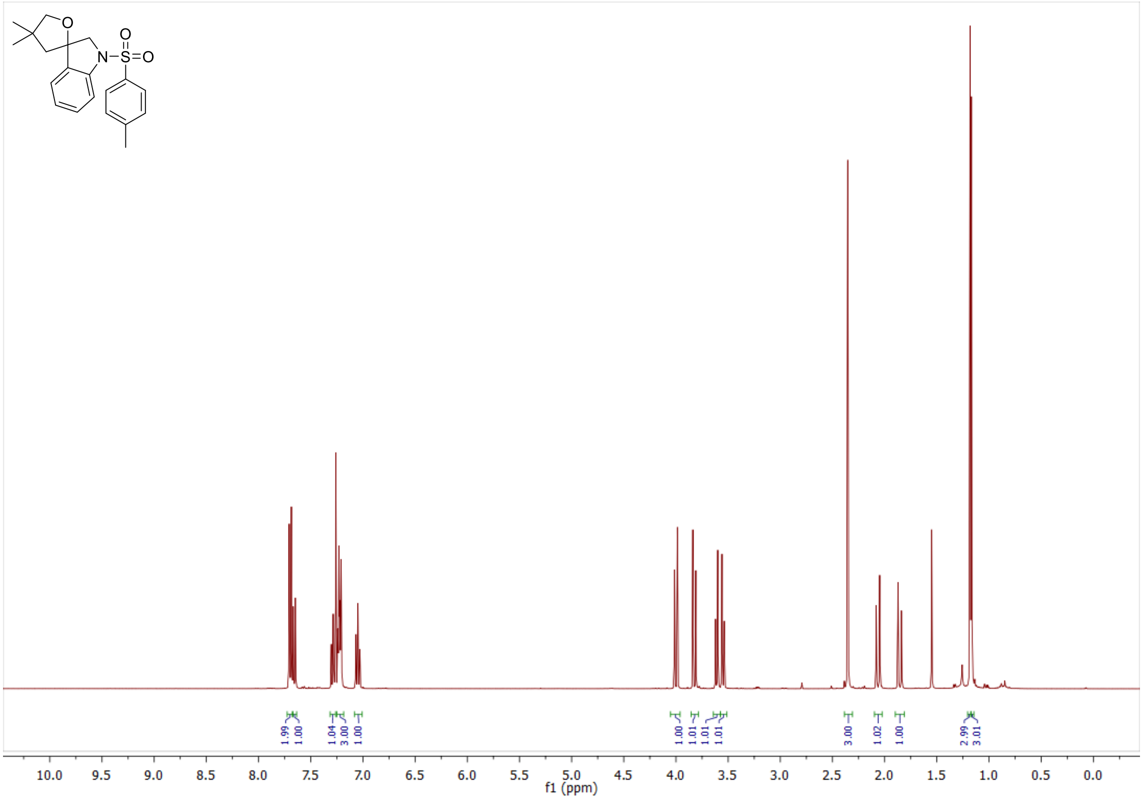

Compound 7: ^{13}C NMR ($CDCl_3$, 400 MHz)

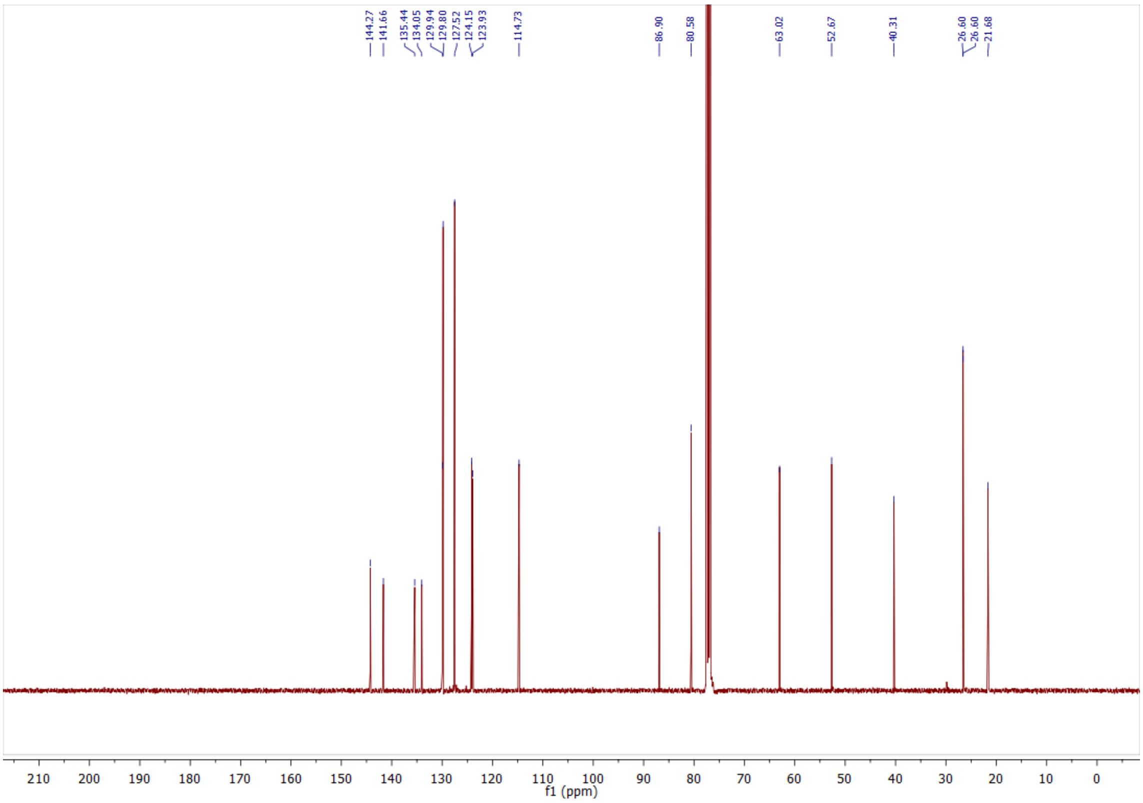

Compound 8A: ^{1}H NMR ($CDCl_3$, 400 MHz)

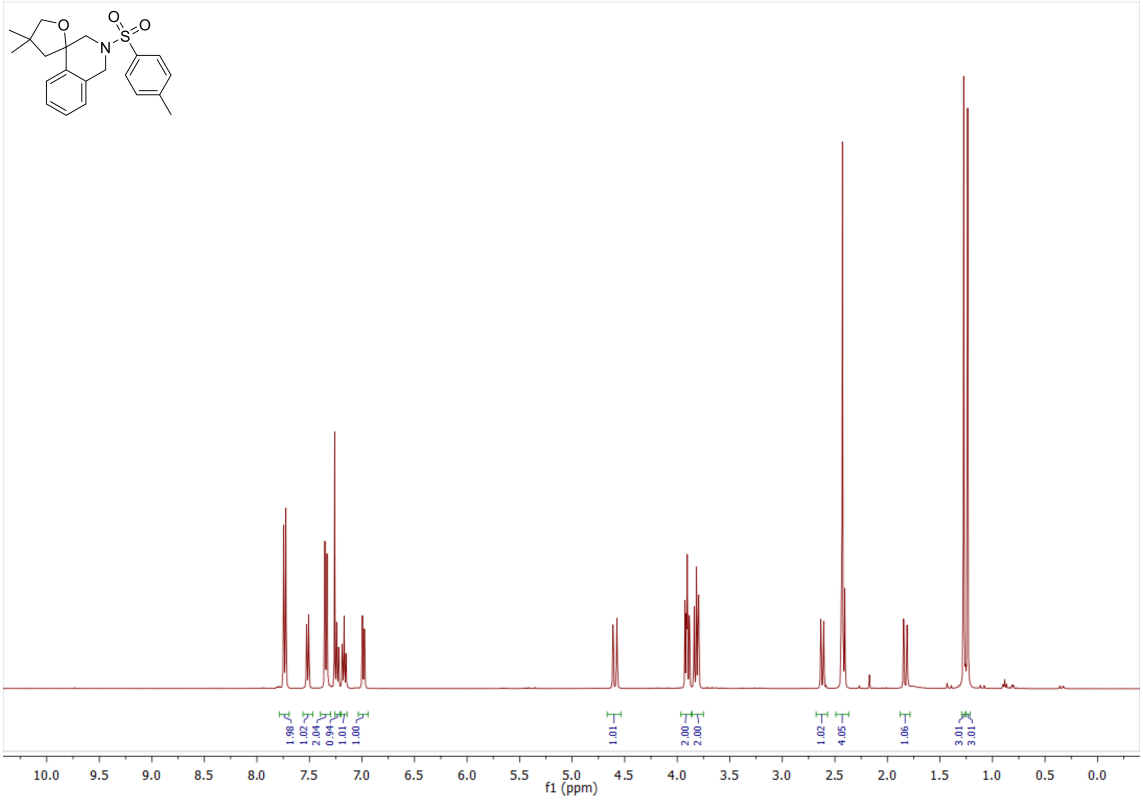

Compound 8A: ^{13}C NMR ($CDCl_3$, 400 MHz)

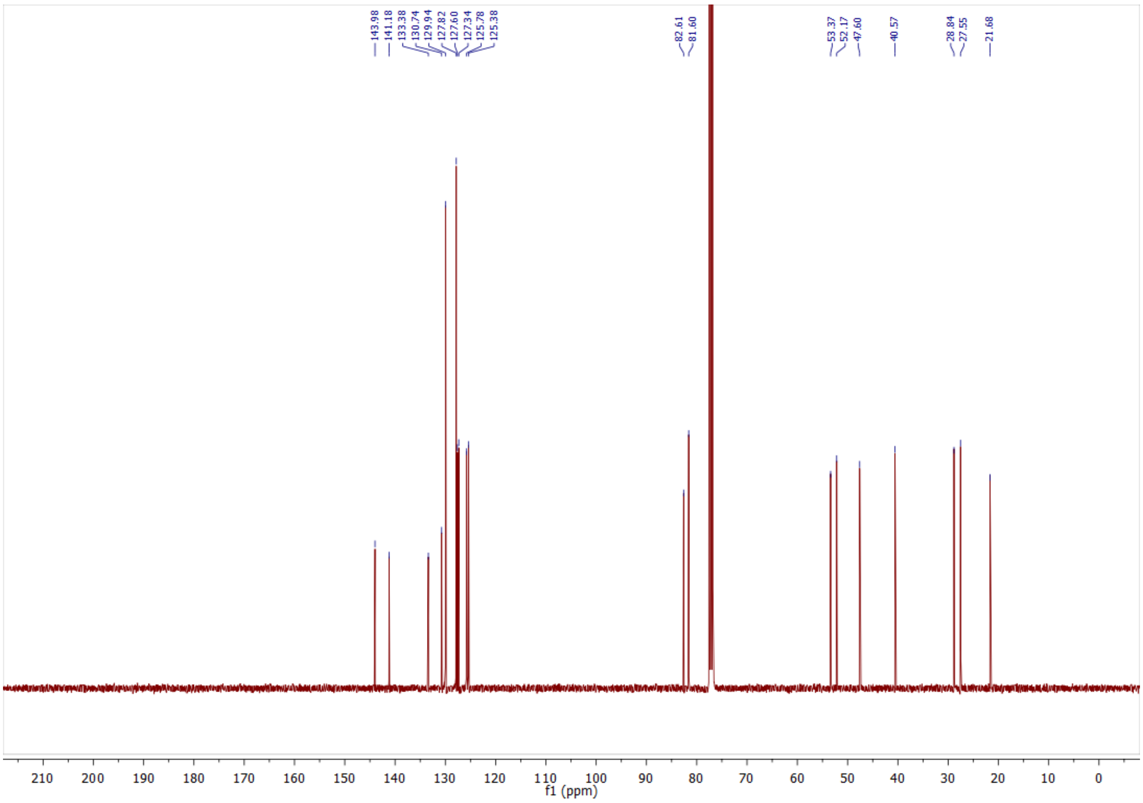

Compound 8B: ^{1}H NMR ($CDCl_3$, 400 MHz)

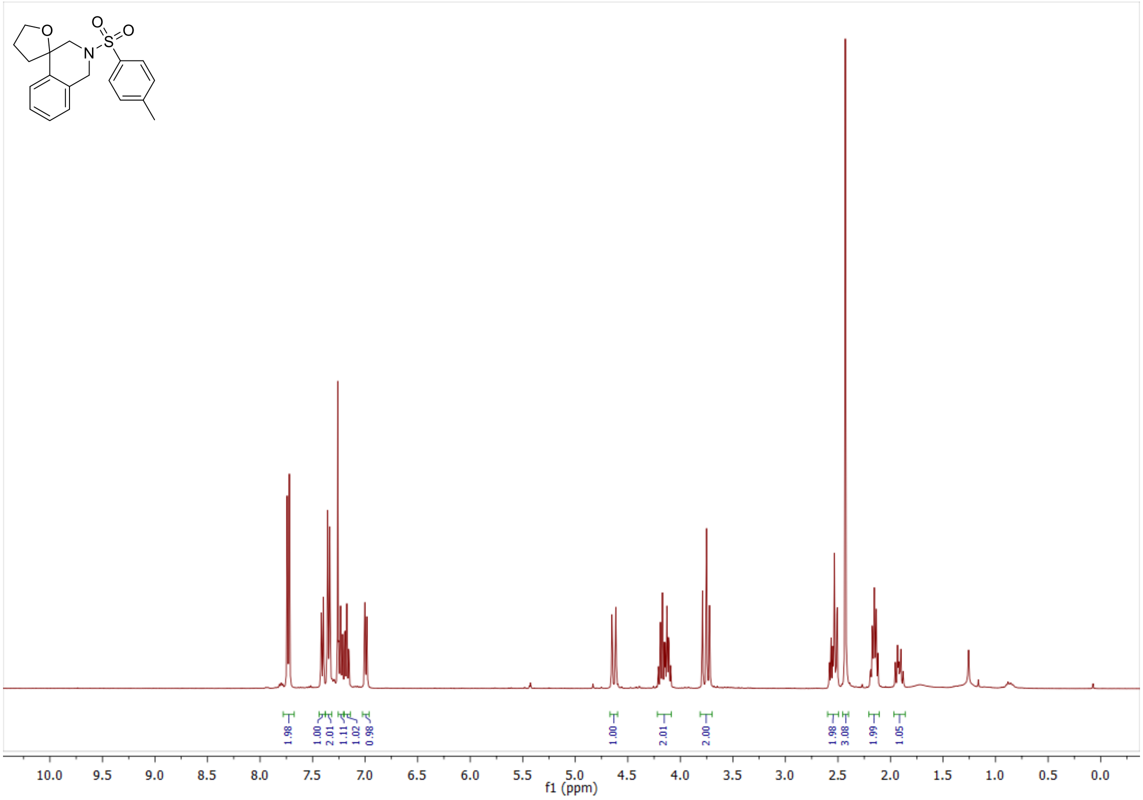

Compound 8B: ^{13}C NMR ($CDCl_3$, 400 MHz)

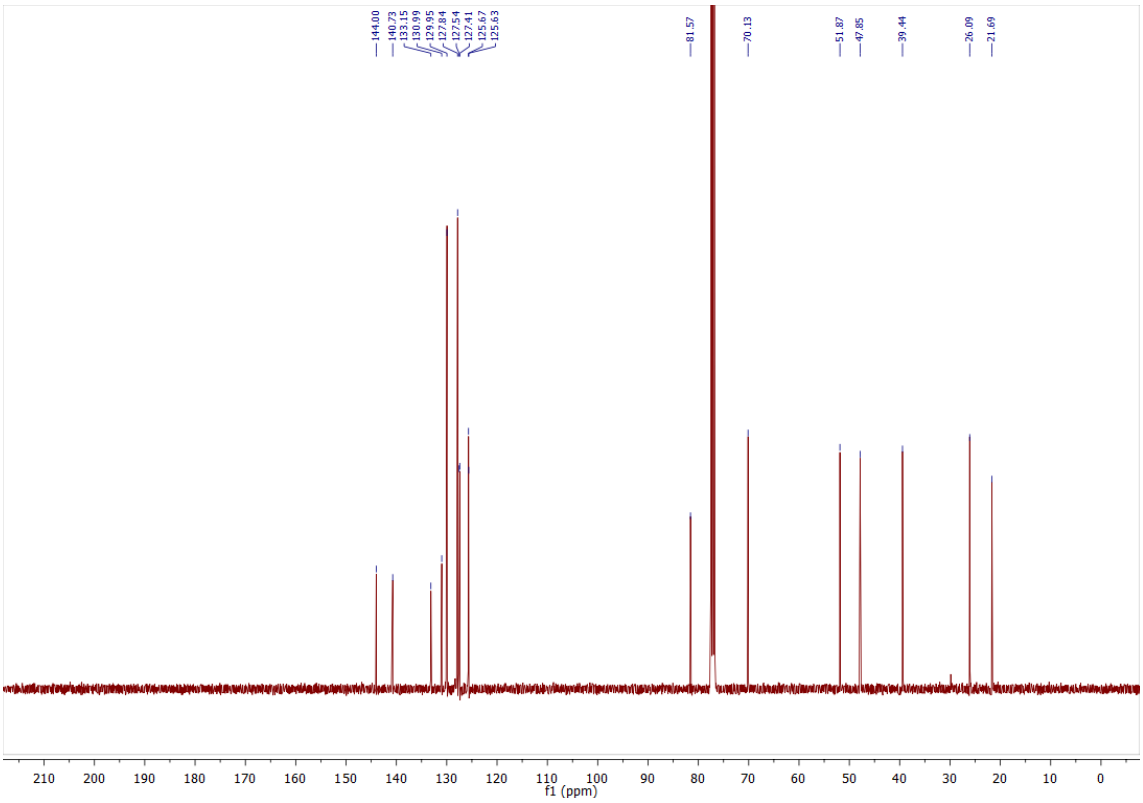

Compound 8C: ^{1}H NMR ($CDCl_3$, 400 MHz)

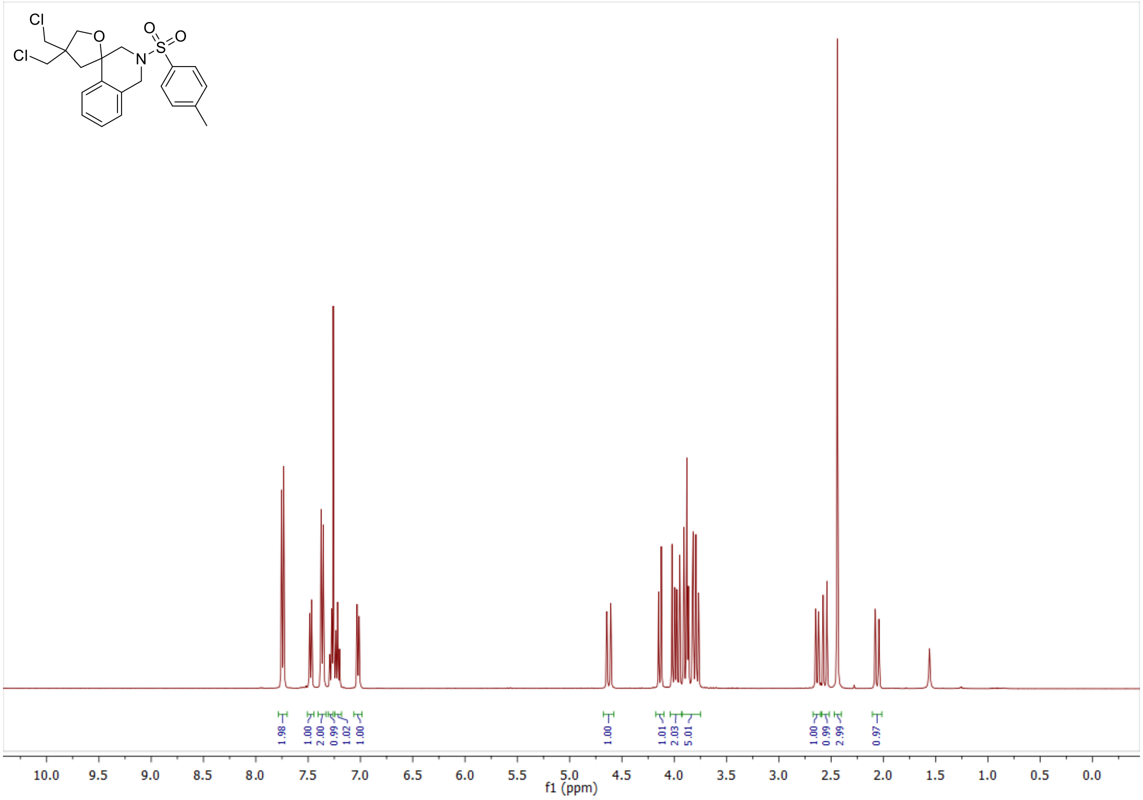

Compound 8C: ^{13}C NMR ($CDCl_3$, 400 MHz)

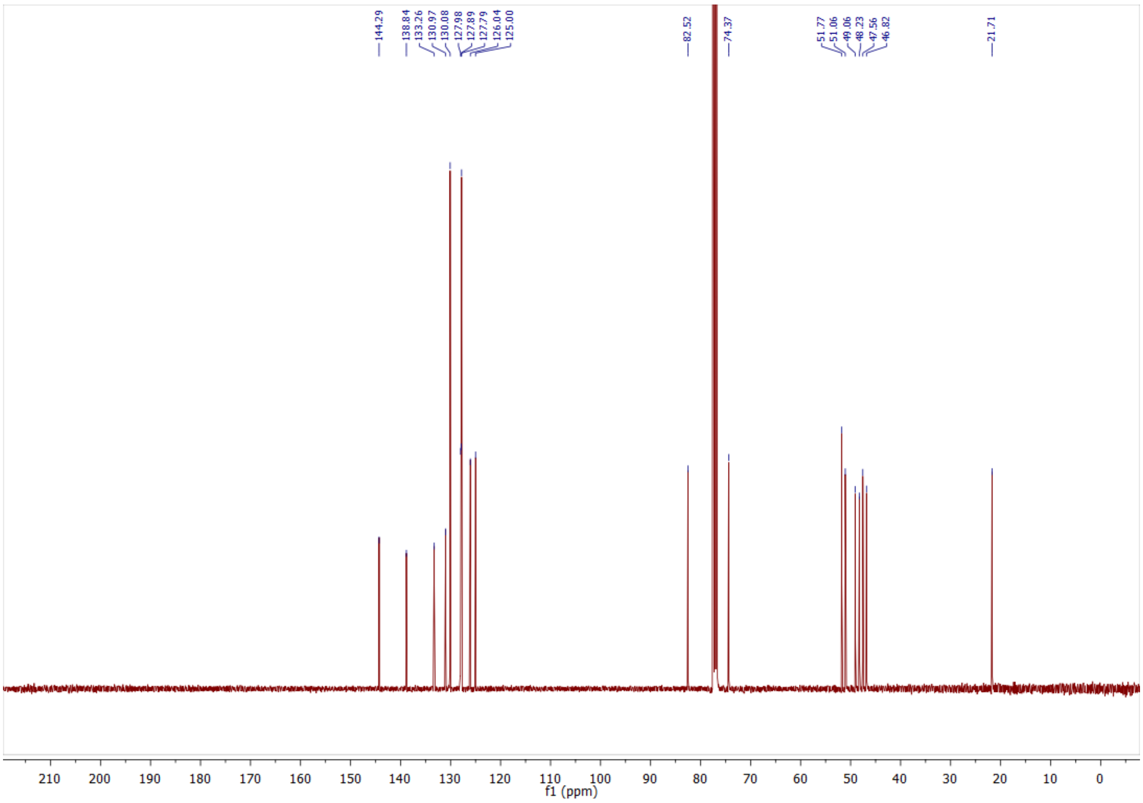

16. Computational details

Geometry optimizations have been performed with the Gaussian 09[5] package at the BP86[6] level of hybrid density functional theory. The sulphur and bromine atoms were represented by the relativistic effective core potential (RECP) from the Stuttgart group and the associated basis sets[7] augmented by a d polarization function[8]. The remaining atoms (H, C, O, N) were represented by a 6-311G** basis set[9]. All energies reported in the present work are Zero-point energies corrected.

Computed Energies and Cartesian Coordinates

Compound (*E*)-5pA

BP86 Energy = -1891.39857398
Z-point correction = 0.677155
Enthalpy correction = 0.721550
Gibbs correction = 0.599795

C	0.405681	4.835754	-1.472396
C	0.603322	3.487510	-1.829765
C	-0.329913	2.860808	-2.677814
C	-1.437636	3.568547	-3.160748
C	-1.628482	4.907937	-2.797294
C	-0.704781	5.538514	-1.951439
C	1.802123	2.727315	-1.346128
S	1.769104	2.331834	0.483978
N	0.274439	1.431938	0.774647
C	-0.814329	2.155149	1.474284
C	-1.162884	1.574668	2.871130
C	0.068918	1.637309	3.795222
O	2.920771	1.420750	0.746391
O	1.645197	3.604500	1.255519
C	-0.137057	0.466199	-0.161439
C	0.528579	-0.596985	-0.669830
N	1.797061	-1.074451	-0.248313
C	2.053116	-1.705919	1.005212
C	3.512696	-2.019494	0.995392
C	4.064544	-1.576924	-0.214447
C	2.988169	-0.935615	-1.022862
C	5.418543	-1.740388	-0.499287
C	6.213464	-2.371171	0.472920
C	5.659309	-2.816259	1.686346
C	4.292670	-2.645046	1.965540
O	1.225704	-1.943624	1.866395
O	3.057715	-0.388155	-2.108912
O	-0.045766	-1.255210	-1.732018
C	-0.333610	-2.665611	-1.502259
C	-1.486727	-3.093253	-2.428552
C	-1.077256	-2.922115	-3.905498
C	-1.605432	0.103571	2.778058
O	-2.752657	-0.030826	1.925356
C	-3.277530	-1.359912	1.979484
C	-4.496005	-1.511070	1.047680
C	-5.078120	-2.925398	1.250868
C	-2.305651	2.437400	3.447202
C	-4.077480	-1.324925	-0.423231
O	-3.095675	-2.299009	-0.785201
C	-2.720419	-2.208811	-2.159631
C	-5.561155	-0.447874	1.382833
C	-1.809743	-4.571666	-2.129989
H	-1.158939	0.596414	-0.532694
H	-1.710047	2.134007	0.829157
H	-0.504693	3.202641	1.597733
H	-0.777339	-0.528655	2.402316
H	-1.862512	-0.252995	3.799091
H	-3.589987	-1.591439	3.021444
H	-2.496184	-2.089782	1.690703
H	-4.973057	-1.429447	-1.073725
H	-3.666613	-0.305257	-0.569762
H	-2.479666	-1.158649	-2.418274
H	-3.561064	-2.537006	-2.808622
H	-0.620775	-2.805733	-0.447888
H	0.570015	-3.264403	-1.721188
H	3.851626	-2.981255	2.906490
H	6.304494	-3.299572	2.424223
H	7.280563	-2.515963	0.286715
H	5.838962	-1.386063	-1.443015
H	2.737975	3.304391	-1.405129
H	1.946992	1.760825	-1.850018
H	-0.176760	1.817092	-2.965619
H	-2.158987	-4.700275	-1.094679
H	-0.923158	-5.209445	-2.283865
H	-2.603044	-4.933156	-2.804850
H	-2.150076	3.073340	-3.825585
H	-2.492103	5.461225	-3.175271
H	-0.847514	6.584351	-1.667568
H	1.121033	5.325312	-0.807298
H	-1.999119	3.493433	3.531641
H	-2.580345	2.084913	4.455090
H	-3.204305	2.381909	2.814320

H	0.896363	1.031219	3.395030
H	-0.179666	1.257619	4.800215
H	0.427120	2.673529	3.898738
H	-5.161077	0.569046	1.256592
H	-5.892009	-0.552298	2.429305
H	-6.448081	-0.561375	0.736910
H	-1.914011	-3.175917	-4.577443
H	-0.235188	-3.589357	-4.152143
H	-0.762753	-1.888536	-4.114755
H	-5.955216	-3.076890	0.599826
H	-5.404011	-3.070403	2.294185
H	-4.336860	-3.700028	1.004056

Compound (*Z*)-5pA

BP86 Energy = -1891.39567739
Z-point correction = 0.677810
Enthalpy correction = 0.722225
Gibbs correction = 0.600529

C	4.029316	-1.771380	0.760432
C	5.414596	-1.519552	0.768417
C	6.030510	-0.981356	1.902794
C	5.270637	-0.689994	3.044371
C	3.891264	-0.934882	3.043597
C	3.271862	-1.468145	1.906466
C	3.370173	-2.364444	-0.446292
S	3.022848	-1.142385	-1.816313
N	1.701880	-0.197117	-1.203811
C	1.854904	1.265489	-1.047885
C	1.438970	2.153980	-2.267242
C	-0.091114	2.341335	-2.310861
O	-0.511886	3.154178	-1.213188
C	-1.936428	3.232395	-1.094118
C	-2.308887	4.333539	-0.078867
C	-1.503714	4.130747	1.217722
O	-1.775914	2.839966	1.776352
C	-0.702180	2.349424	2.573372
C	-1.039747	0.934915	3.080973
C	-1.399119	0.014063	1.903949
O	-0.263268	-0.096866	1.000259
C	-0.412842	-0.869034	-0.117214
C	0.490006	-0.908631	-1.127906
O	2.532548	-1.967440	-2.963905
O	4.184107	-0.219110	-1.970077
C	-1.945590	5.720866	-0.647416
C	-3.821744	4.248640	0.201592
C	2.124954	3.525056	-2.076935
C	1.884182	1.542112	-3.608616
C	0.175201	0.388257	3.853962
C	-2.278468	0.992587	4.002060
N	-1.549053	-1.727676	-0.211799
C	-2.813790	-1.369027	-0.766549
C	-3.633338	-2.616967	-0.721554
C	-2.862338	-3.646315	-0.162312
C	-1.511299	-3.106270	0.174175
C	-4.943430	-2.835441	-1.143041
C	-5.466116	-4.130654	-0.987255
C	-4.693262	-5.163041	-0.425840
C	-3.372929	-4.933467	-0.002868
O	-3.121064	-0.264405	-1.175169
O	-0.554700	-3.673126	0.667163
H	1.801366	4.231618	-2.859079
H	3.219879	3.417551	-2.144779
H	1.872364	3.968823	-1.102401
H	-0.039982	-0.614468	4.259126
H	0.425189	1.046448	4.702617
H	1.058078	0.313926	3.201726
H	3.295510	-0.715713	3.933292
H	-0.526457	3.012853	3.448158
H	0.229631	2.322589	1.974039
H	-5.127955	-6.159995	-0.319795
H	-2.341626	2.254211	-0.776111
H	-2.385937	3.483307	-2.079103
H	5.753576	-0.275644	3.933234
H	2.389682	-2.813177	-0.227690
H	3.999179	-3.103049	-0.965196
H	-2.762312	-5.728169	0.431371
H	-6.488996	-4.341867	-1.308553
H	-5.532836	-2.026228	-1.579774
H	6.007420	-1.741720	-0.122458
H	7.106849	-0.791145	1.897254
H	1.381920	0.583435	-3.814586
H	2.968517	1.357019	-3.613948
H	1.648927	2.232219	-4.435760
H	-3.139037	1.434122	3.477690
H	-2.063308	1.616333	4.884899
H	-2.558309	-0.012034	4.361151
H	2.195837	-1.661238	1.904157
H	-0.374164	2.833738	-3.266975
H	-0.601448	1.357720	-2.278560
H	-2.133489	6.517907	0.091829
H	-2.554085	5.942659	-1.539751
H	-0.885206	5.758320	-0.940341
H	-4.082565	3.290962	0.676432
H	-4.400740	4.341063	-0.732826
H	-4.137642	5.063491	0.874245
H	-0.426918	4.213814	0.981809
H	-1.763354	4.921991	1.952718
H	0.288488	-1.603922	-1.946011
H	-1.654185	-0.987752	2.293048

H	-2.257662	0.432639	1.356196
H	2.916212	1.442200	-0.822699
H	1.264205	1.562456	-0.166457

Compound (*E*)-5qA

BP86 Energy = -1966.62943710
Z-point correction = 0.681272
Enthalpy correction = 0.726910
Gibbs correction = 0.603560

S	-2.167260	-0.766279	-1.748416
O	0.353012	0.378895	1.899245
O	-2.391434	-1.154885	-3.171068
O	3.173812	-0.478273	-1.719044
O	-2.841535	0.419328	-1.153621
N	-0.410730	-0.444666	-1.612906
O	0.387199	2.906975	-1.142074
N	-0.909264	1.782004	0.472278
O	3.666787	-0.045473	1.766311
O	-2.653334	1.269375	1.971512
O	-3.225335	-5.572155	1.604596
C	-0.140829	0.600895	0.639691
C	0.107418	-0.337393	-0.304258
H	0.819781	-1.115405	-0.007322
C	-2.514112	-2.195463	-0.702018
C	1.257721	1.403923	2.401788
H	0.670718	2.238059	2.828722
H	1.875352	1.777790	1.569094
C	-2.624999	3.338021	0.661167
C	-1.717880	3.818224	-0.292271
C	-0.601706	2.836889	-0.434667
C	0.433511	-1.241968	-2.537043
H	-0.235103	-1.783495	-3.221474
H	1.016231	-1.979949	-1.955571
C	4.243214	0.226448	-1.084753
H	3.845454	1.083815	-0.507147
H	4.932081	0.628970	-1.859550
C	-2.479922	-3.480608	-1.271584
H	-2.289919	-3.598738	-2.339968
C	2.399310	0.400778	-2.550441
H	3.079336	0.956050	-3.232231
H	1.866559	1.143571	-1.926882
C	-2.143328	2.016727	1.158094
C	-1.917887	5.036015	-0.939032
H	-1.204337	5.398470	-1.682314
C	2.151595	0.777601	3.487796
C	-2.795420	-2.009886	0.657360
H	-2.838079	-1.005861	1.085664
C	1.399845	-0.394804	-3.408176
C	1.289065	0.294530	4.671464
H	0.782810	1.147195	5.153145
H	1.911551	-0.201404	5.434845
H	0.515723	-0.413045	4.335865
C	-3.000625	-4.420333	0.907681
C	-2.724107	-4.588422	-0.466456
H	-2.719505	-5.600075	-0.877194
C	-3.770085	4.054603	1.002147
H	-4.473079	3.666362	1.742390
C	2.898618	-0.438329	2.904387
H	3.563093	-0.873871	3.681825
H	2.160283	-1.210782	2.613347
C	4.146176	-1.170358	1.024736
H	3.287774	-1.753016	0.633166
H	4.738112	-1.838007	1.687872
C	5.034675	-0.702391	-0.143645
C	0.602482	0.613021	-4.259734
H	-0.150593	0.093311	-4.873028
H	1.275485	1.165739	-4.936241
H	0.078795	1.342378	-3.623935
C	-3.070306	5.766304	-0.601878
H	-3.264902	6.723876	-1.091339
C	-3.982166	5.283042	0.353489
H	-4.871137	5.872686	0.591100
C	-3.038506	-3.127686	1.465030
H	-3.262415	-2.974576	2.521257
C	3.152610	1.853784	3.954842
H	3.816390	2.160313	3.132880
H	3.780276	1.463515	4.772766
H	2.626140	2.746099	4.333138
C	2.158905	-1.379987	-4.323205
H	2.762997	-2.086899	-3.734380
H	2.841186	-0.829771	-4.992255
H	1.459736	-1.954205	-4.954085
C	6.242948	0.086867	0.401860
H	6.905835	0.408699	-0.418469
H	6.836141	-0.542818	1.085933
H	5.917052	0.977289	0.959730
C	5.520348	-1.951049	-0.906725
H	4.672757	-2.522323	-1.313323
H	6.107301	-2.612330	-0.247208
H	6.166182	-1.659190	-1.751113
C	-3.522823	-5.461911	3.002908
H	-3.666080	-6.491337	3.353674
H	-4.447577	-4.883377	3.173002
H	-2.688090	-4.997391	3.556475

Compound (*Z*)-5qA

BP86 Energy = -1966.62619351
Z-point correction = 0.681408

Enthalpy correction = 0.727253
Gibbs correction = 0.602100

C	-0.118172	5.787489	-0.752080
C	0.277685	4.451302	-0.738925
C	1.601069	4.076837	-1.011720
C	2.579377	5.024160	-1.307880
C	2.189133	6.374111	-1.323887
C	0.861548	6.749780	-1.050704
C	1.707781	2.589476	-0.925924
N	0.395540	2.134391	-0.592444
C	-0.524714	3.221369	-0.465643
C	0.016320	0.761932	-0.470773
O	0.133549	0.157848	0.746647
C	0.806575	0.882827	1.811639
C	0.764497	0.050320	3.103650
C	-0.691724	-0.233159	3.519800
O	-1.705923	3.122434	-0.189357
O	2.681481	1.878908	-1.091760
C	-0.453312	0.136979	-1.579985
N	-0.863635	-1.203736	-1.697200
C	-0.158477	-2.359344	-1.112047
C	0.953404	-2.976811	-2.025167
C	0.551337	-2.946322	-3.511268
S	-2.519124	-1.407320	-2.213672
O	-2.674725	-2.843182	-2.587542
C	-3.534638	-1.116003	-0.746072
C	-3.702110	0.187334	-0.261649
C	-4.484390	0.402531	0.878989
C	-5.105181	-0.686686	1.521040
C	-4.929974	-1.993191	1.018964
C	-4.144717	-2.210294	-0.110009
O	-5.897757	-0.584787	2.629624
C	-6.128041	0.722602	3.168940
O	-2.769850	-0.302313	-3.181031
C	2.270519	-2.191073	-1.863838
O	2.815615	-2.424549	-0.562494
C	3.906377	-1.553823	-0.248860
C	4.590234	-2.034580	1.048778
C	5.605407	-0.963810	1.492749
C	1.159419	-4.439599	-1.575543
C	3.527218	-2.261414	2.139821
O	2.802135	-1.051920	2.391979
C	1.491834	-1.291939	2.898824
C	5.303131	-3.380359	0.800660
C	1.484420	0.872028	4.195274
H	2.007636	-4.895535	-2.112449
H	0.257566	-5.035598	-1.792060
H	1.374406	-4.503361	-0.498265
H	-1.221507	0.709152	3.736650
H	-0.724112	-0.852395	4.431646
H	-1.241452	-0.755729	2.723169
H	1.537791	-1.829371	3.871061
H	0.924156	-1.925138	2.188064
H	0.589375	7.807865	-1.073063
H	3.538700	-0.517199	-0.136102
H	4.650890	-1.563396	-1.073922
H	-1.152684	6.066874	-0.540749
H	2.927255	7.146361	-1.553960
H	3.606621	4.720038	-1.520551
H	0.489363	-1.915239	-3.893898
H	-0.434163	-3.412342	-3.659807
H	1.293266	-3.493868	-4.115931
H	2.543221	1.027090	3.939279
H	1.443021	0.337520	5.158279
H	1.005826	1.855545	4.337234
H	2.997824	-2.523212	-2.636468
H	2.091516	-1.107379	-2.015523
H	5.731800	-3.783985	1.733632
H	6.128952	-3.251178	0.081744
H	4.603370	-4.122817	0.387234
H	5.099028	-0.021091	1.748941
H	6.336178	-0.757810	0.692373
H	6.166089	-1.304601	2.379241
H	2.830844	-3.048429	1.796637
H	4.015134	-2.609751	3.074861
H	-0.573386	0.747375	-2.476312
H	0.287741	1.842407	1.983581
H	1.852619	1.067610	1.520587
H	-0.916455	-3.132562	-0.918268
H	0.284225	-2.066454	-0.146821
H	-3.226949	1.035943	-0.755890
H	-4.602606	1.421215	1.249440
H	-5.429813	-2.819271	1.529035
H	-4.013747	-3.213565	-0.519063
H	-6.788397	0.571636	4.031929
H	-5.186828	1.192447	3.504658
H	-6.626879	1.379207	2.434639

Compound (*E*)-5kA

BP86 Energy = -1583.95316043
Z-point correction = 0.625018
Enthalpy correction = 0.665893
Gibbs correction = 0.551049

C	3.891230	-0.175728	-1.460338
C	2.895867	0.724398	-1.062621
C	2.872973	1.177615	0.264061
C	3.833748	0.762251	1.195027
C	4.833259	-0.133291	0.796953

C	4.845131	-0.596818	-0.524735
S	1.547703	2.309663	0.786782
N	0.130731	1.275746	1.153773
C	0.351848	0.412084	2.342864
C	-0.801693	0.436880	3.383253
C	-0.375544	-0.476431	4.553175
Br	6.212459	-1.825865	-1.071777
O	1.954819	2.915869	2.087542
O	1.168824	3.126824	-0.393996
C	-0.399734	0.593550	0.027972
C	-1.178713	1.123634	-0.954145
C	-1.784056	2.471246	-1.050235
C	-2.288110	3.150545	0.076950
O	-1.353030	0.284896	-2.046683
C	-2.728802	-0.061700	-2.371137
C	-2.740724	-1.431758	-3.075427
C	-4.206289	-1.787654	-3.399023
C	-1.907046	-1.371388	-4.371277
C	-2.121761	-2.496735	-2.149468
O	-2.850207	-2.558829	-0.922330
C	-2.190631	-3.354774	0.063973
C	-3.032239	-3.403059	1.353876
C	-2.295090	-4.287594	2.379140
C	-2.123322	-0.079349	2.785658
O	-1.972796	-1.419738	2.313380
C	-3.226967	-1.981322	1.915131
C	-1.023411	1.873031	3.894841
C	-4.425243	-3.992983	1.051321
H	-0.120049	-0.462476	-0.081794
H	-3.165344	0.709892	-3.030939
H	-3.317231	-0.112603	-1.440783
H	1.254760	0.769695	2.859997
H	0.526620	-0.629593	2.015962
H	-3.700389	-1.342319	1.143799
H	-3.909137	-2.022937	2.791928
H	3.813921	1.159249	2.211757
H	-2.906483	-0.044764	3.574052
H	-2.457006	0.576207	1.955343
H	2.158428	1.089042	-1.779475
H	-2.349213	-0.653380	-5.081460
H	-1.877296	-2.356543	-4.865747
H	-0.873883	-1.053832	-4.163937
H	5.597522	-0.459294	1.503847
H	-2.139887	-3.486417	-2.654988
H	-1.064556	-2.234035	-1.947808
H	-1.192394	-2.926377	0.286385
H	-2.043497	-4.388849	-0.316580
H	-0.111553	2.254058	4.381006
H	-1.843510	1.904986	4.631348
H	-1.262729	2.561720	3.070844
H	3.930676	-0.536617	-2.489115
H	-4.802494	-1.891009	-2.480220
H	-4.255790	-2.744375	-3.944819
H	-4.670432	-1.014842	-4.034298
H	-0.237845	-1.515110	4.217952
H	-1.147091	-0.474857	5.340677
H	0.565918	-0.122336	5.004905
H	-5.036448	-4.050932	1.967257
H	-4.329444	-5.014293	0.646897
H	-4.962222	-3.385388	0.307951
H	-1.301493	-3.879307	2.616488
H	-2.170860	-5.313759	1.994718
H	-2.868633	-4.347799	3.318830
C	-1.894826	3.086507	-2.314908
C	-2.485210	4.347120	-2.446239
C	-2.985870	5.011440	-1.318839
C	-2.885383	4.406475	-0.057706
H	-1.490727	2.572622	-3.190087
H	-2.550019	4.815143	-3.432121
H	-3.448782	5.996347	-1.420721
H	-3.273681	4.917956	0.826957
H	-2.203388	2.688226	1.061375

Compound (*Z*)-5kA

BP86 Energy = -1583.95300197
Z-point correction = 0.625808
Enthalpy correction = 0.666527
Gibbs correction = 0.552032

Br	-5.039316	-2.866541	-1.018091
S	-0.906394	0.093697	3.037348
C	0.870354	0.376856	-3.081256
C	1.036786	1.434926	-1.973242
H	2.055275	1.379038	-1.549591
H	0.878507	2.444404	-2.389267
C	-0.571509	3.540443	-0.557313
C	1.105649	-1.041525	-2.524139
H	0.410491	-1.226951	-1.681219
H	0.881399	-1.785772	-3.318774
C	-2.818024	-2.746527	0.802822
H	-2.731720	-3.817116	0.611504
C	-3.825000	-1.994352	0.184913
C	3.939644	0.175500	1.309402
H	3.878301	0.880991	0.452276
H	4.918862	0.338952	1.808906
C	-0.171988	1.996146	1.328806
H	-0.587252	2.773747	1.972850
C	-1.409664	3.625391	-1.691663
H	-1.741177	2.704558	-2.176599
C	4.663341	-1.399131	-0.319067
H	5.719347	-1.555078	-0.010821

H	4.627765	-0.519143	-0.990380
C	1.458377	0.072477	1.574020
H	1.558484	0.202013	0.487820
H	1.268011	-0.995619	1.747206
C	-3.975853	-0.623774	0.432251
H	-4.778119	-0.060932	-0.047083
C	-0.172992	2.217374	-0.018958
C	-3.092823	0.011606	1.312577
H	-3.207121	1.072095	1.542222
C	-1.931638	-2.108237	1.678269
H	-1.150478	-2.672421	2.191265
C	-1.838783	4.866555	-2.169919
H	-2.494900	4.912788	-3.043301
C	4.157254	-2.633918	-1.087641
C	1.900047	0.694317	-4.185852
H	1.721598	1.694006	-4.615664
H	1.822169	-0.042520	-5.001886
H	2.927955	0.656840	-3.796375
C	-1.443049	6.048648	-1.526372
H	-1.779826	7.018227	-1.901795
C	-0.167975	4.737404	0.071651
H	0.510015	4.688312	0.927170
C	-2.073482	-0.735632	1.918297
C	2.792937	0.472981	2.295162
C	4.292532	-3.899975	-0.218001
H	5.354026	-4.112331	-0.009373
H	3.869540	-4.780891	-0.730050
H	3.775502	-3.773302	0.745264
C	-0.609212	5.978089	-0.403135
H	-0.287240	6.894467	0.098480
C	4.993480	-2.773593	-2.375748
H	4.823045	-1.920511	-3.050238
H	4.722074	-3.695745	-2.915886
H	6.070742	-2.826812	-2.145267
C	2.667049	-2.449263	-1.436031
H	2.342468	-3.279075	-2.100242
H	2.058805	-2.491022	-0.511610
C	-0.557757	0.425054	-3.661824
H	-1.311042	0.203613	-2.890119
H	-0.671745	-0.309167	-4.476116
H	-0.771849	1.422404	-4.079496
C	2.985906	-0.337916	3.591940
H	3.073855	-1.412328	3.368718
H	2.134441	-0.203622	4.274838
H	3.905887	-0.018915	4.110788
C	2.844749	1.980421	2.612648
H	3.830345	2.246221	3.029934
H	2.080190	2.246909	3.357228
H	2.675569	2.598010	1.715136
O	2.457840	-1.192666	-2.089426
O	0.076927	1.186120	-0.903874
O	3.838892	-1.162897	0.823300
N	0.275506	0.843176	2.008600
O	-0.198115	-0.966034	3.807776
O	-1.640859	1.208873	3.695897

17. Crystallographic data

All data were collected on an Agilent supernova dual source diffractometer equipped with an Atlas detector, using Cu Kα radiation. Data reduction was carried out in the crysalis Pro Software.[10] Structure solution was made using direct methods (Shelxs[11] or sir2004[12]). Refinements were carried out in ShexlL[11] within the Olex2[13] software.

Details for the refinement for each structure can be found below with. For each structure, a representation of the asymmetric units shown as displacement ellipsoids, drawn as 50 percent probability, is depicted.

Compound 9aA

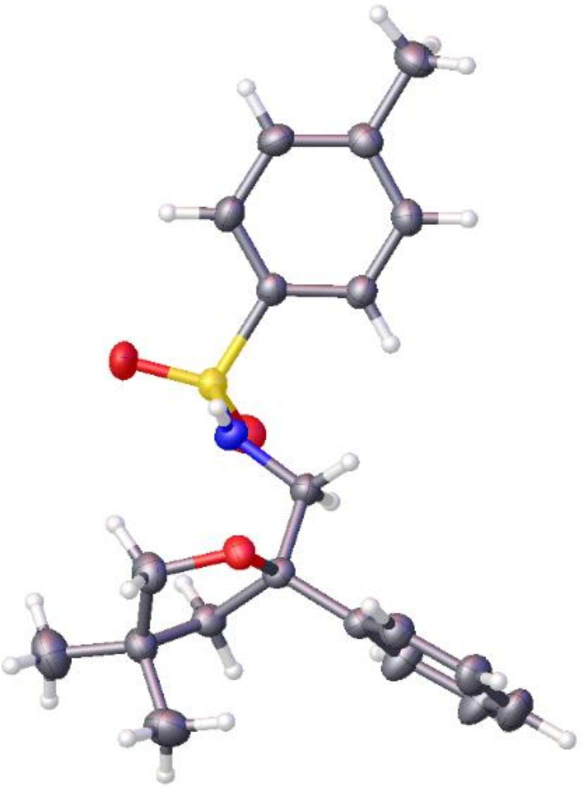

CCDC number	1534813
Empirical formula	$C_{20}H_{25}NO_3S$
Formula weight	359.47
Temperature/K	180.1(9)
Crystal system	triclinic
Space group	P-1
a/Å	8.3686(4)
b/Å	10.3246(4)
c/Å	12.6600(5)
α/°	70.135(3)
β/°	77.897(3)
γ/°	68.067(4)
Volume/Å^3	950.00(7)
Z	2
ρ_{calc}g/cm^3	1.257
μ/mm^{-1}	1.657
F(000)	384.0
Crystal size/mm^3	0.4844 × 0.2604 × 0.1423
Radiation	CuKα (λ = 1.54184)
2Θ range for data collection/°	7.458 to 146.836
Index ranges	$-10 \le h \le 9$, $-12 \le k \le 12$, $-15 \le l \le 15$
Reflections collected	14320
Independent reflections	3724 [R_{int} = 0.0274, R_{sigma} = 0.0189]
Data/restraints/parameters	3724/1/232
Goodness-of-fit on F^2	1.039
Final R indexes [I>=2σ (I)]	R_1 = 0.0486, wR_2 = 0.1410
Final R indexes [all data]	R_1 = 0.0510, wR_2 = 0.1437
Largest diff. peak/hole / e Å^{-3}	0.88/-0.37

Compound 5kA

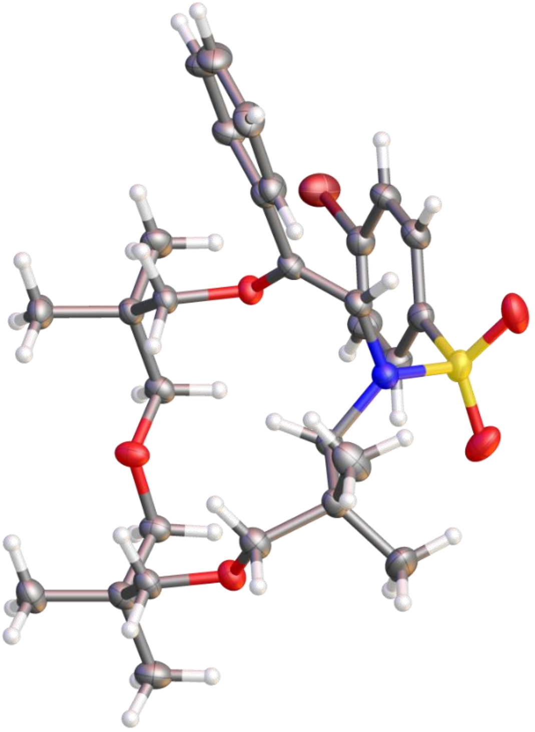

CCDC number	1443619
Empirical formula	$C_{29}H_{40}BrNO_5S$
Formula weight	594.59
Temperature/K	180.10(14)
Crystal system	monoclinic
Space group	$P2_1/n$
a/Å	14.0045(3)
b/Å	12.0323(2)
c/Å	17.8455(4)
α/°	90
β/°	100.017(2)
γ/°	90
Volume/Å^3	2961.25(11)
Z	4
ρ_{calc}g/cm^3	1.334
μ/mm^{-1}	2.850
F(000)	1248.0
Crystal size/mm^3	0.7111 × 0.5249 × 0.2855
Radiation	CuKα (λ = 1.54184)
2Θ range for data collection/°	7.428 to 145.164
Index ranges	$-17 \leq h \leq 13$, $-14 \leq k \leq 14$, $-21 \leq l \leq 21$
Reflections collected	20048
Independent reflections	5813 [R_{int} = 0.0226, R_{sigma} = 0.0170]
Data/restraints/parameters	5813/0/340
Goodness-of-fit on F^2	1.060
Final R indexes [I>=2σ (I)]	R_1 = 0.0293, wR_2 = 0.0797
Final R indexes [all data]	R_1 = 0.0306, wR_2 = 0.0806
Largest diff. peak/hole / e Å^{-3}	0.38/-0.41

Compound 6gA

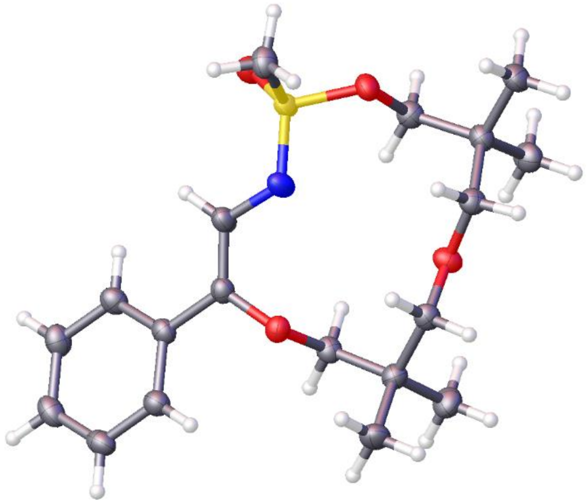

CCDC number	1534812
Empirical formula	$C_{19}H_{29}NO_4S$
Formula weight	367.49
Temperature/K	181(2)
Crystal system	monoclinic
Space group	$P2_1/c$
a/Å	17.4991(2)
b/Å	5.67440(6)
c/Å	20.5923(3)
α/°	90
β/°	107.1557(13)
γ/°	90
Volume/Å^3	1953.78(4)
Z	4
ρ_{calc}g/cm^3	1.249
μ/mm^{-1}	1.655
F(000)	792.0
Crystal size/mm^3	0.683 × 0.123 × 0.032
Radiation	CuKα (λ = 1.54184)
2Θ range for data collection/°	8.984 to 147.822
Index ranges	$-21 \le h \le 16$, $-6 \le k \le 7$, $-25 \le l \le 24$
Reflections collected	12491
Independent reflections	3874 [R_{int} = 0.0279, R_{sigma} = 0.0241]
Data/restraints/parameters	3874/0/231
Goodness-of-fit on F^2	1.051
Final R indexes [I>=2σ (I)]	R_1 = 0.0377, wR_2 = 0.1005
Final R indexes [all data]	R_1 = 0.0412, wR_2 = 0.1050
Largest diff. peak/hole / e Å^{-3}	0.31/-0.40

Compound (Z)-5pA

CCDC number	1443620
Empirical formula	$C_{32}H_{42}N_2O_7S$
Formula weight	598.73
Temperature/K	180.00(10)
Crystal system	monoclinic
Space group	$P2_1/c$
a/Å	9.20537(16)
b/Å	23.7526(5)
c/Å	14.5525(3)
α/°	90
β/°	92.7036(16)
γ/°	90
Volume/Å^3	3178.39(10)
Z	4
ρ_{calc}mg/mm^3	1.251
m/mm^{-1}	1.302
F(000)	1280.0
Crystal size/mm^3	0.3563 × 0.2427 × 0.1806
Radiation	CuKα (λ = 1.54184)
2Θ range for data collection	7.13 to 144.92°
Index ranges	$-7 \leq h \leq 11$, $-29 \leq k \leq 27$, $-16 \leq l \leq 17$
Reflections collected	12141
Independent reflections	6139 [R_{int} = 0.0165, R_{sigma} = 0.0204]
Data/restraints/parameters	6139/0/385
Goodness-of-fit on F^2	1.024
Final R indexes [I>=2σ (I)]	R_1 = 0.0481, wR_2 = 0.1176
Final R indexes [all data]	R_1 = 0.0507, wR_2 = 0.1196
Largest diff. peak/hole / e Å^{-3}	0.59/-0.58

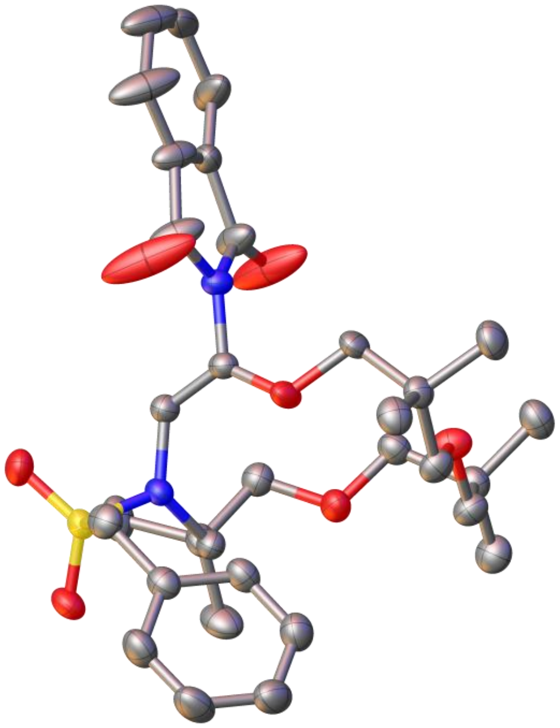

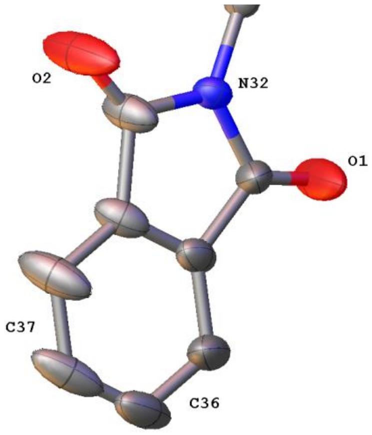

The geometry of the phthalimido group is ill-defined, due to a disorder affecting this group. This disorder seems to be partly due to a libration around an axis passing through N32 and the centre of C36-C37, as reflected on the large ellipsoids of O1 and O2 .The combination of this with a rotation around an axis perpendicular to the ring and passing through N32 may also explain the large displacement parameters of C36 C37.

Compound (E)-5pA

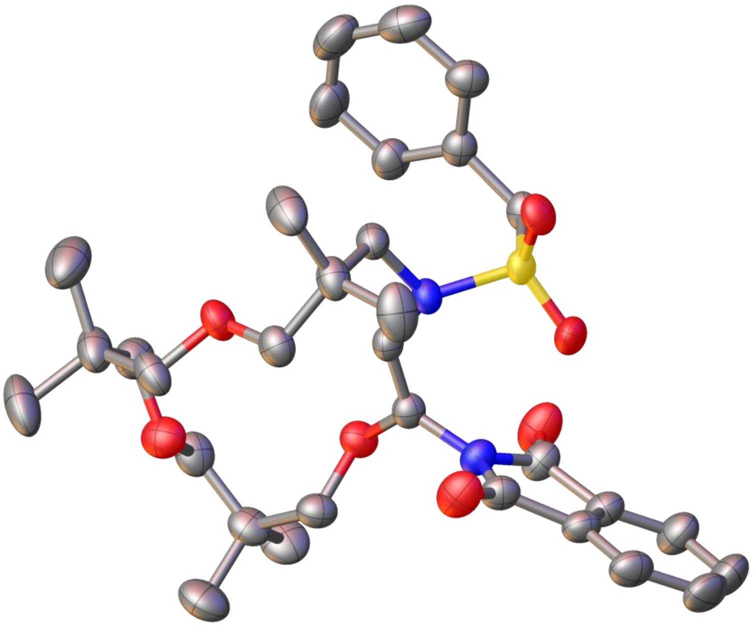

CCDC number	1443618
Empirical formula	$C_{32}H_{42}N_2O_7S$
Formula weight	598.73
Temperature/K	210.00(14)
Crystal system	triclinic
Space group	P-1
a/Å	8.58394(13)
b/Å	10.36548(13)
c/Å	19.8233(3)
α/°	96.6637(11)
β/°	102.3792(13)
γ/°	102.1801(12)
Volume/Å^3	1659.83(4)
Z	2
ρ_{calc}mg/mm^3	1.198
m/mm^{-1}	1.247
F(000)	640.0
Crystal size/mm^3	0.3551 × 0.2181 × 0.0347
Radiation	CuKα (λ = 1.54184)
2Θ range for data collection	8.854 to 144.824°
Index ranges	-9 ≤ h ≤ 10, -12 ≤ k ≤ 9, -24 ≤ l ≤ 23
Reflections collected	11170
Independent reflections	6391 [R_{int} = 0.0198, R_{sigma} = 0.0272]
Data/restraints/parameters	6391/0/385
Goodness-of-fit on F^2	1.018
Final R indexes [I>=2σ (I)]	R_1 = 0.0362, wR_2 = 0.0994
Final R indexes [all data]	R_1 = 0.0412, wR_2 = 0.1054
Largest diff. peak/hole / e Å^{-3}	0.27/-0.30

Compound 7

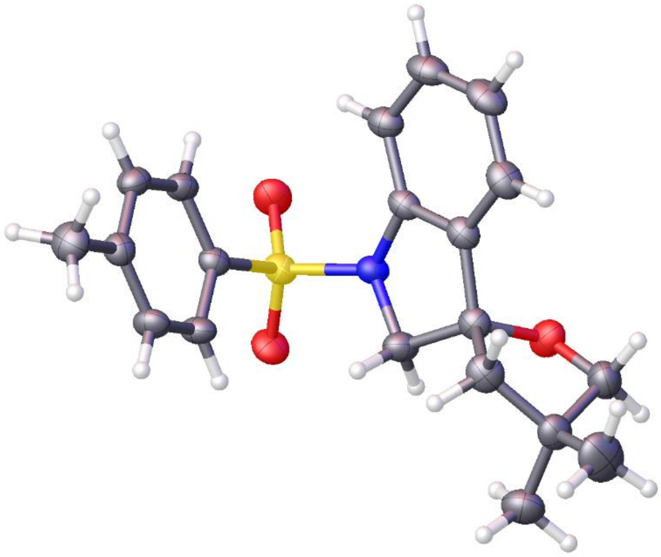

CCDC number	1534815
Empirical formula	$C_{20}H_{23}NO_3S$
Formula weight	357.45
Temperature/K	180.12(10)
Crystal system	monoclinic
Space group	$P2_1/n$
a/Å	11.0583(3)
b/Å	12.1489(3)
c/Å	13.6776(3)
α/°	90
β/°	98.545(2)
γ/°	90
Volume/Å^3	1817.14(8)
Z	4
ρ_{calc}g/cm^3	1.307
μ/mm^{-1}	1.732
F(000)	760.0
Crystal size/mm^3	0.595 × 0.422 × 0.108
Radiation	CuKα (λ = 1.54184)
2Θ range for data collection/°	9.786 to 147.176
Index ranges	-13 ≤ h ≤ 13, -14 ≤ k ≤ 14, -17 ≤ l ≤ 10
Reflections collected	11597
Independent reflections	3592 [R_{int} = 0.0280, R_{sigma} = 0.0221]
Data/restraints/parameters	3592/0/229
Goodness-of-fit on F^2	1.040
Final R indexes [I>=2σ (I)]	R_1 = 0.0409, wR_2 = 0.1118
Final R indexes [all data]	R_1 = 0.0431, wR_2 = 0.1141
Largest diff. peak/hole / e Å^{-3}	0.36/-0.40

Compound 8A

CCDC number	1534814
Empirical formula	$C_{21}H_{25}NO_3S$
Formula weight	371.48
Temperature/K	179(2)
Crystal system	triclinic
Space group	P-1
a/Å	10.6084(6)
b/Å	10.7200(5)
c/Å	10.8762(7)
α/°	117.600(6)
β/°	110.658(5)
γ/°	94.009(4)
Volume/Å^3	984.19(11)
Z	2
ρ_{calc}g/cm^3	1.254
μ/mm^{-1}	1.617
F(000)	396.0
Crystal size/mm^3	0.493 × 0.13 × 0.026
Radiation	Cu Kα (λ = 1.54184)
2Θ range for data collection/°	9.284 to 146.602
Index ranges	-13 ≤ h ≤ 13, -13 ≤ k ≤ 13, -13 ≤ l ≤ 12
Reflections collected	14460
Independent reflections	3882 [R_{int} = 0.0391, R_{sigma} = 0.0291]
Data/restraints/parameters	3882/88/269
Goodness-of-fit on F^2	1.039
Final R indexes [I>=2σ (I)]	R_1 = 0.0413, wR_2 = 0.1124
Final R indexes [all data]	R_1 = 0.0485, wR_2 = 0.1187
Largest diff. peak/hole / e Å^{-3}	0.41/-0.36

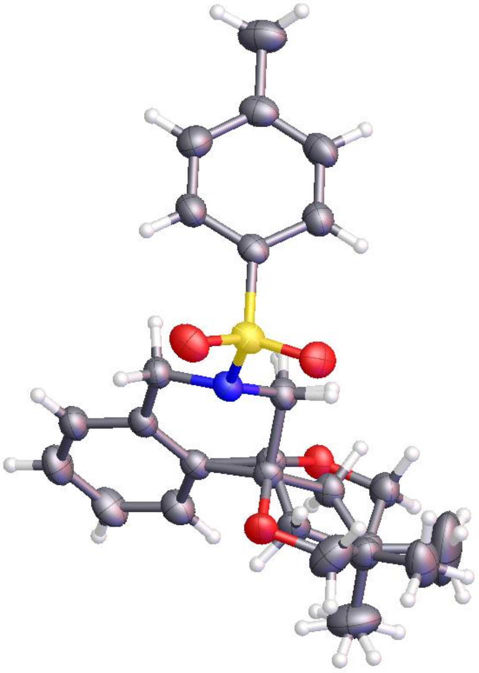

One part of the molecule is disordered and was refined using two components (green and red part on the side figure).
Restraints (DFIX) were applied on distances.
Restraints (RIGU) and constraints (EADP) were applied on anisotropic displacement parameter.

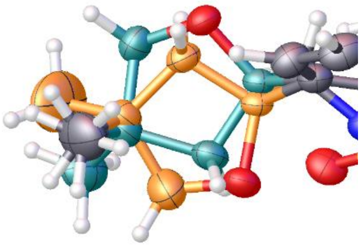

18. Structural Diversity Computational Analysis

A) Principal Moment of Inertia (PMI)

General details

Principal Moment of Inertia (PMI) was performed using Molecular Operating Environment (MOE) software package version 2012.10 from the Chemical Computing Group. Merck molecular force field 94X (MMFF94x), an all-atom force field parameterised for small organic molecules with the Generalised Born solvation model, was used to minimise the energy potential of the library members. A LowModeMD search was employed for the conformation generation. Detailed settings for conformational search are listed below.

Rejection Limit	100
RMS Gradient	0.005
Iteration Limit	10000
MM Iteration Limit	500
RMSD Limit	0.15
Energy window	3
Conformation Limit	100

Only the conformer with the lowest energy was retained for principal moment of inertia (PMI) calculations. Normalized PMI ratios (I1/I3 and I2/I3) of these conformers were obtained from MOE and then plotted on a triangular graph, with the canonical coordinates (0,1), (0.5,0.5) and (1,1) representing a perfect rod, disc and sphere respectively (Figure 1).

Collection 1: The 53 DOS compounds from this work.

Collection 2: 40 high-profile synthetic drugs currently produced by the pharmaceutical industry.

See F. Kopp, C. F. Stratton, L. B. Akella and D. S. Tan, *Nat. Chem. Biol,* **2012**, *8*, 358.

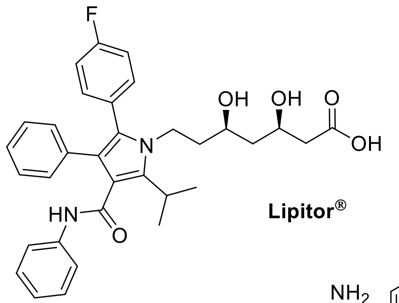
Lipitor®

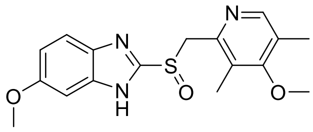
Nexium®

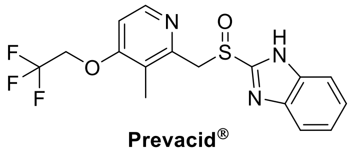
Prevacid®

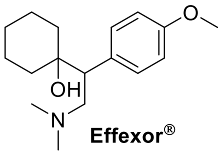
Effexor®

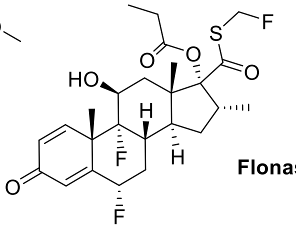
Flonase®

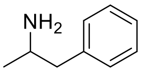
Adderall®

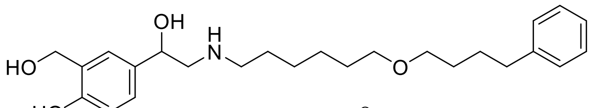
Eerevent®

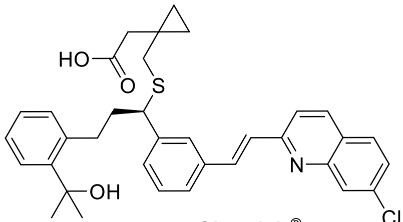
Singulair®

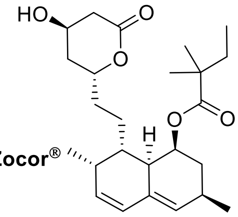
Zocor®

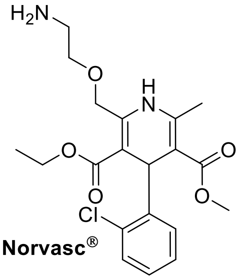
Norvasc®

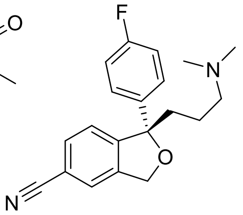
Lexapro®

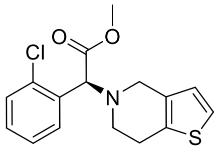
Plavix®

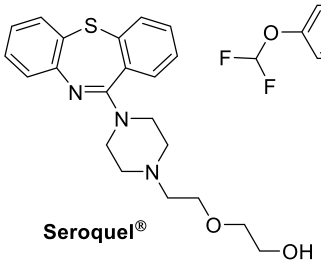
Seroquel®

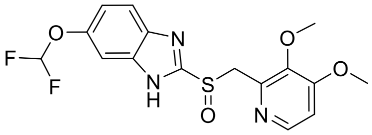
Protonix®

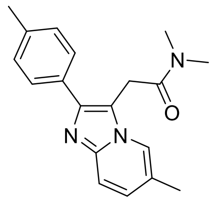
Ambien®

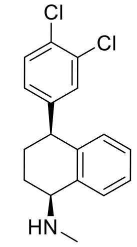
Zoloft®

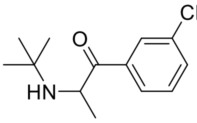
Wellbutrin®

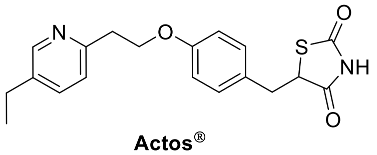
Actos®

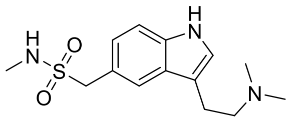
Imitrex®

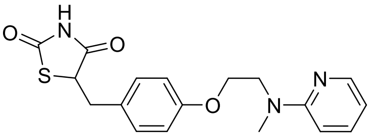
Avandia®

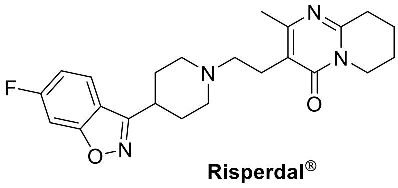

Risperdal®

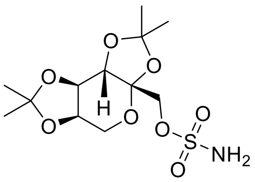

Topomax®

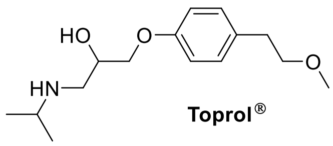

Toprol®

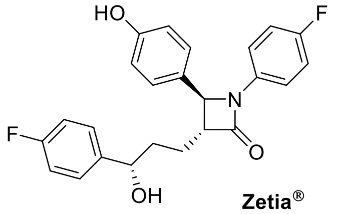

Zetia®

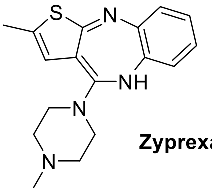

Zyprexa®

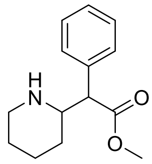

Concerta®

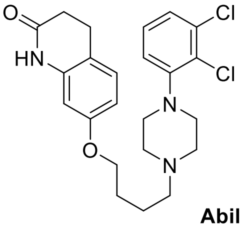

Ability®

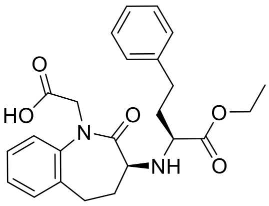

Benazepril®

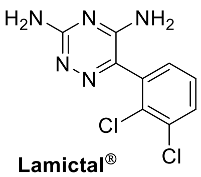

Lamictal®

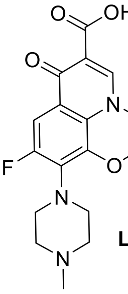

Levaquin®

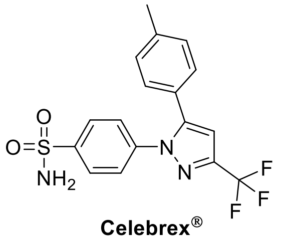

Celebrex®

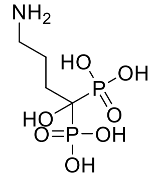

Fosamax®

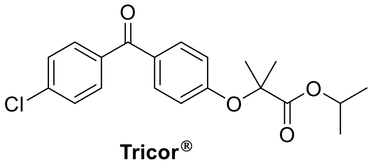

Tricor®

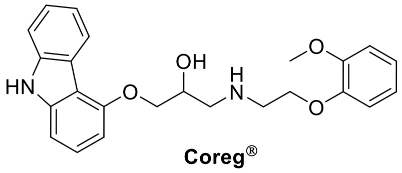

Coreg®

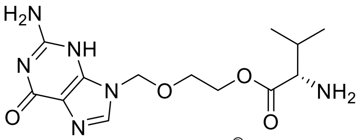

Valtrex®

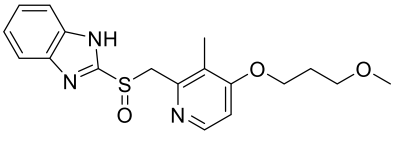

Aciphex®

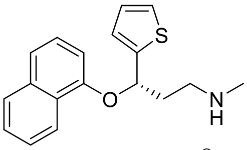

Cymbalta®

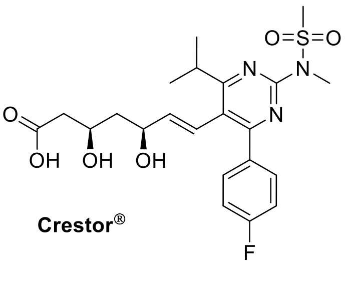

Crestor®

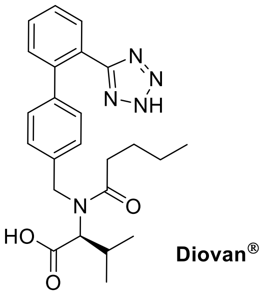

Diovan®

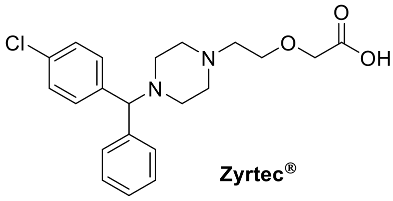

Zyrtec®

Collection 3: 60 randomly selected natural products.

See F. Kopp, C. F. Stratton, L. B. Akella and D. S. Tan, *Nat. Chem. Biol,* **2012**, *8*, 358.

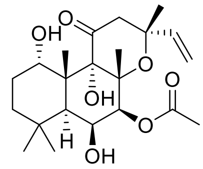

forskolin

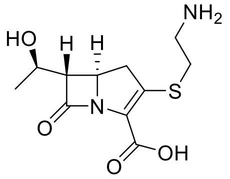

thienamycin

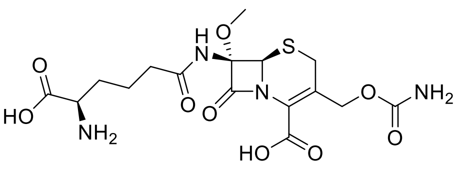

cephamycin C

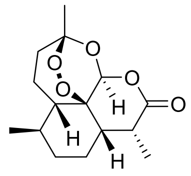

artemisinin

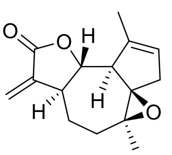

arglabin

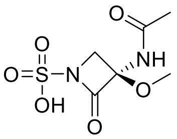

SQ26180

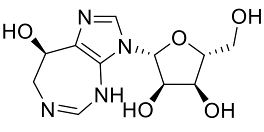

coformycin

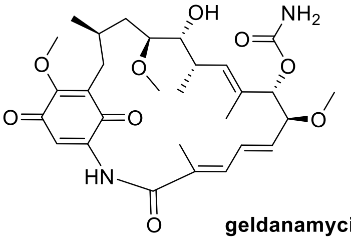

geldanamycin

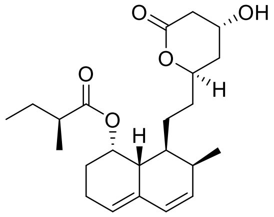

compactin

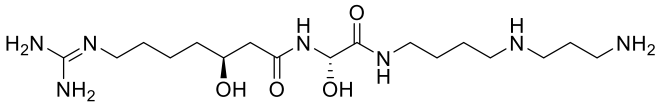

spergualin

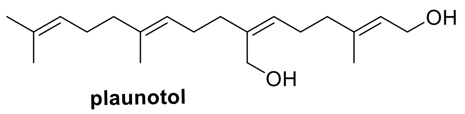

plaunotol

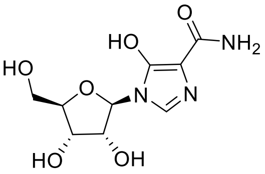

mizoribine

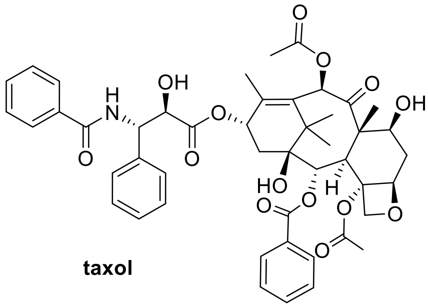

taxol

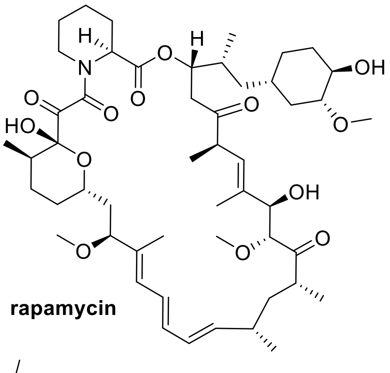

rapamycin

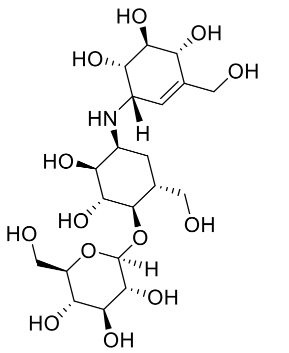

validamycin

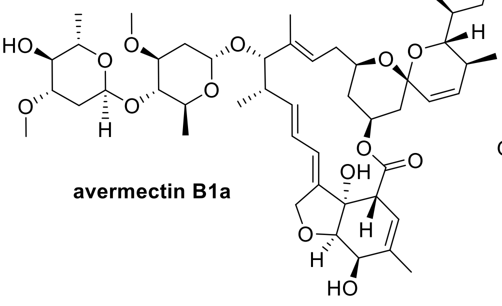

avermectin B1a

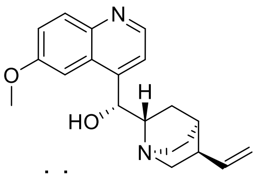

quinine

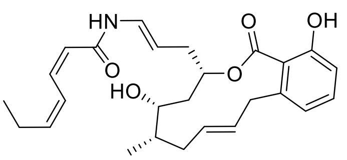

salicylihalamide A

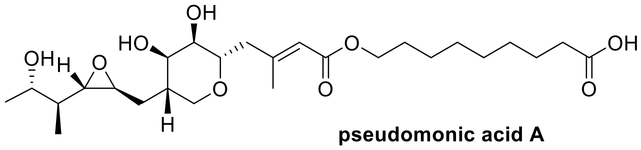

pseudomonic acid A

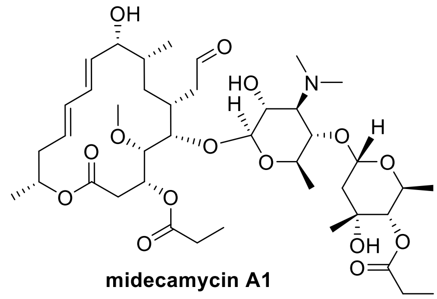

midecamycin A1

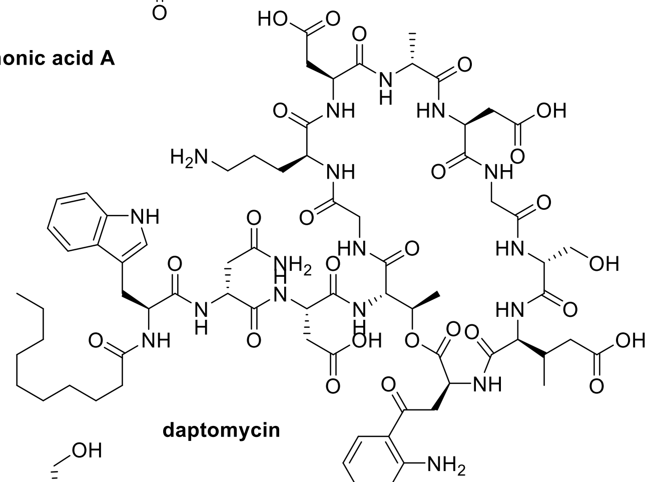

daptomycin

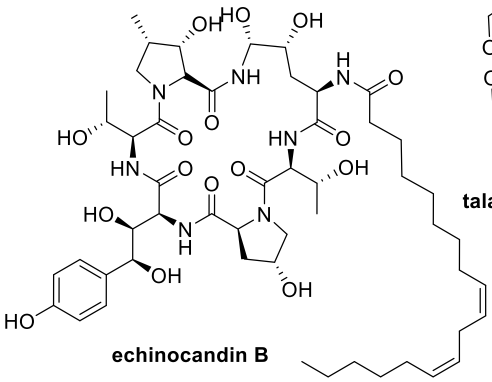

echinocandin B

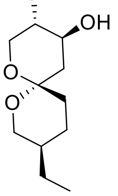

talaromycin B

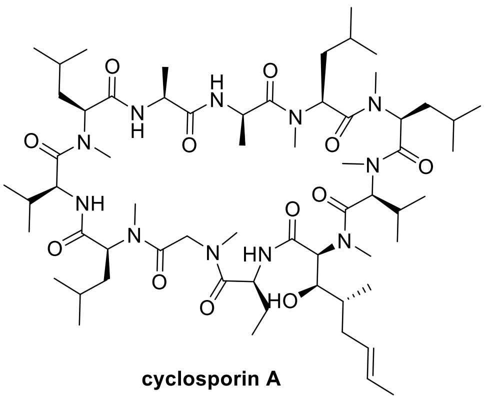

cyclosporin A

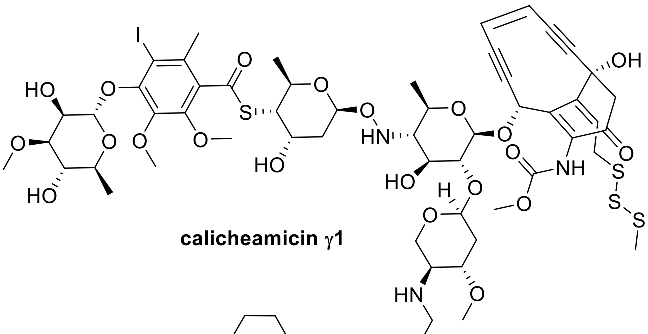

calicheamicin γ1

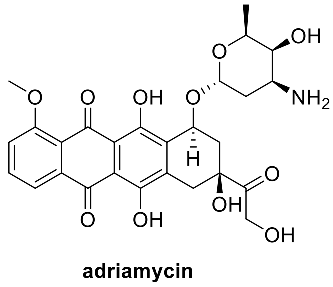

adriamycin

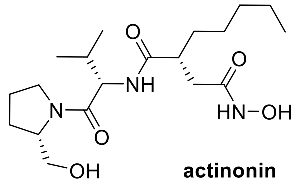

actinonin

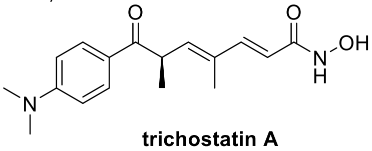

trichostatin A

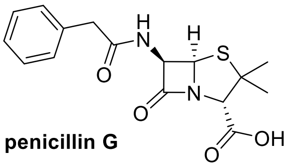

penicillin G

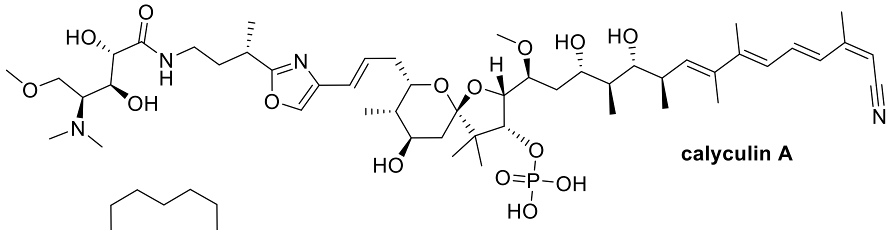

calyculin A

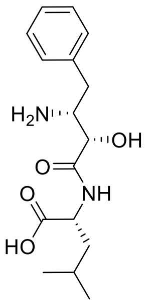

bestatin

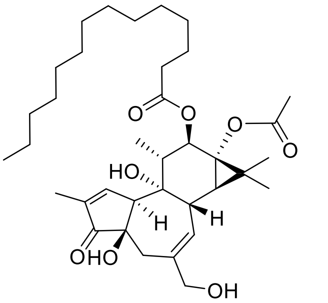

phorbol myristate acetate

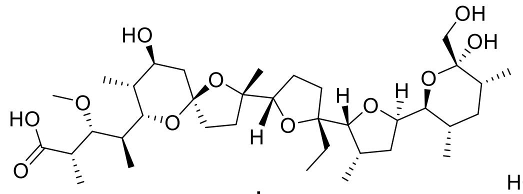

monensin

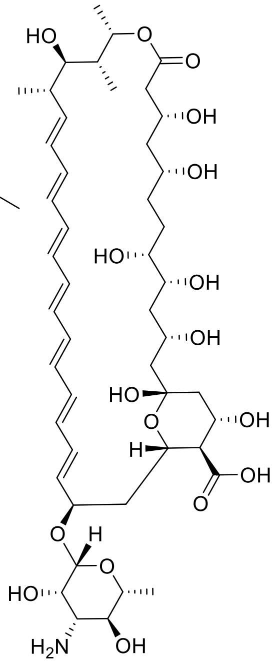

amphotericin B

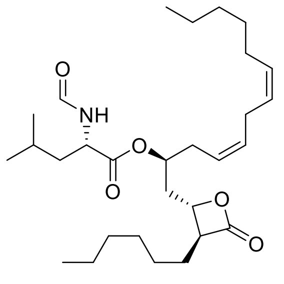

lipstatin

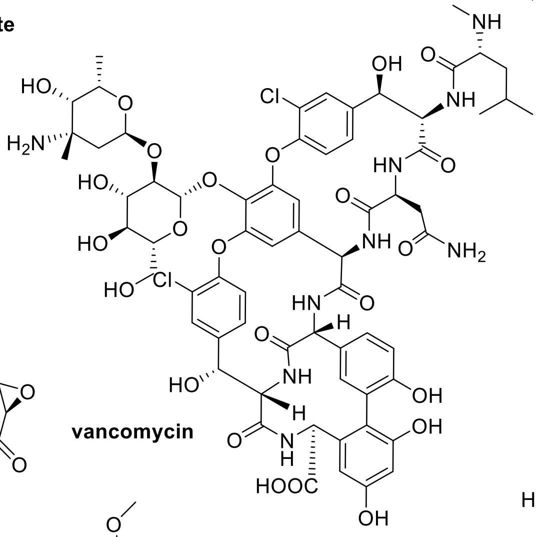

vancomycin

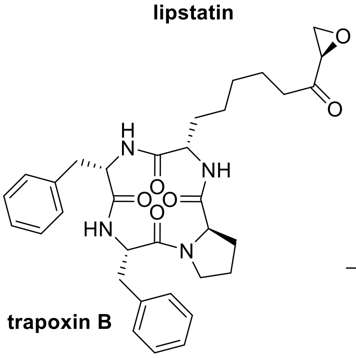

trapoxin B

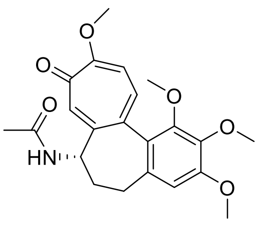

colchicine

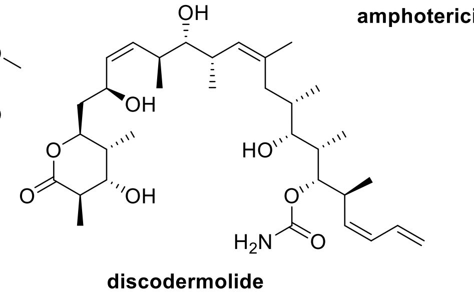

discodermolide

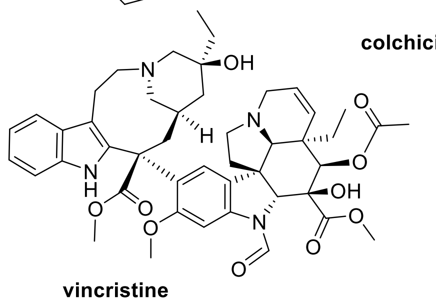

vincristine

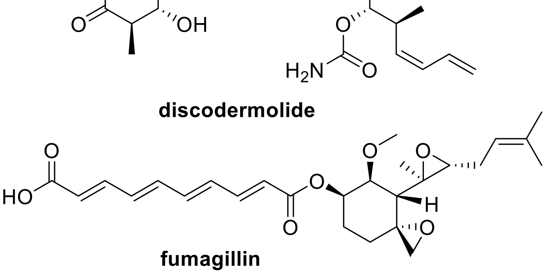

fumagillin

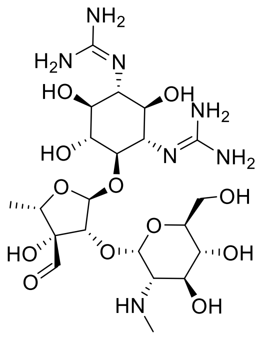

streptomycin

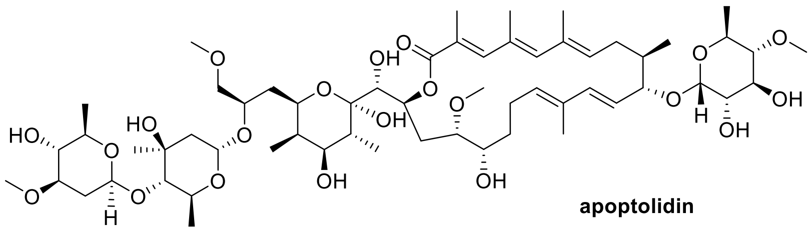

apoptolidin

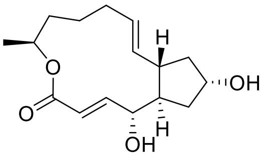

brefeldin A

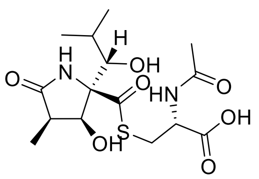

lactacystin

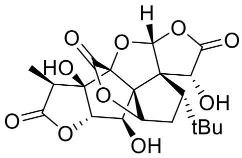

ginkgolide B

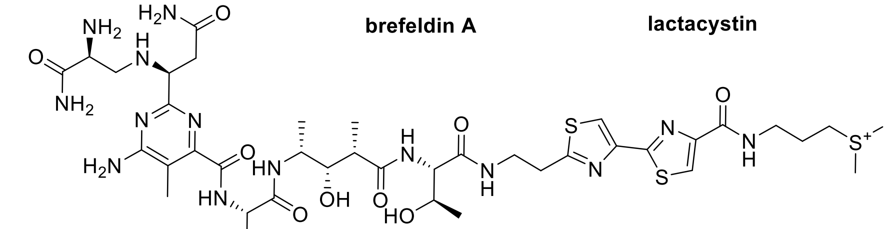
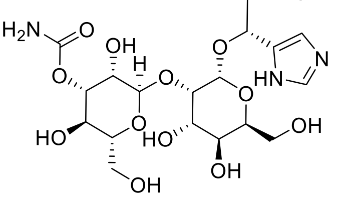

bleomycin A2

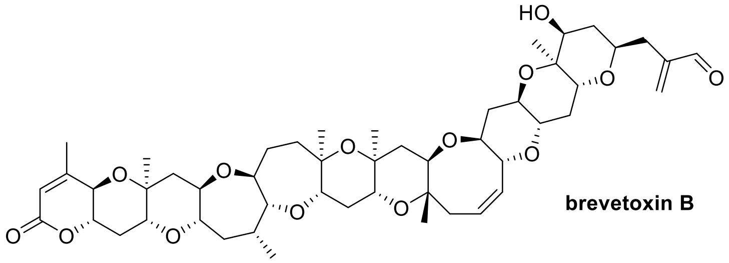

brevetoxin B

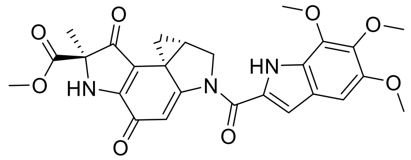

duocarmycin A

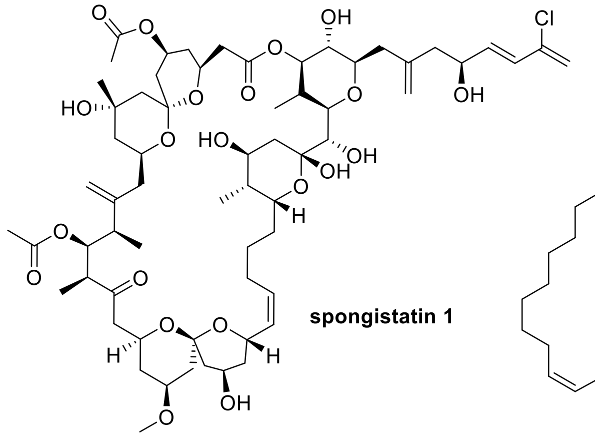

spongistatin 1

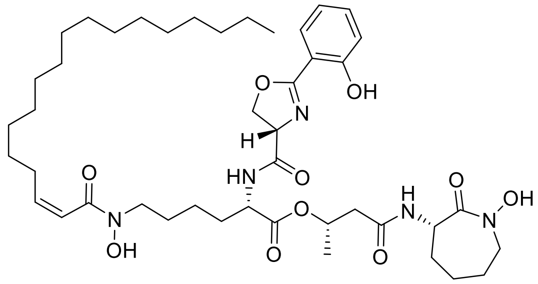

mycobactin S

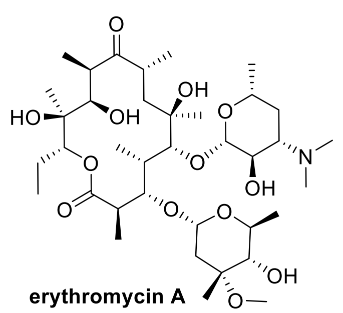

erythromycin A

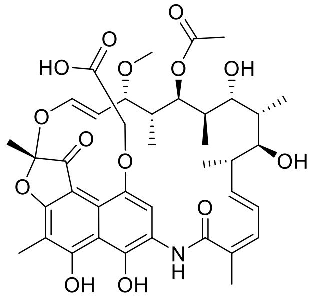

rifamycin B

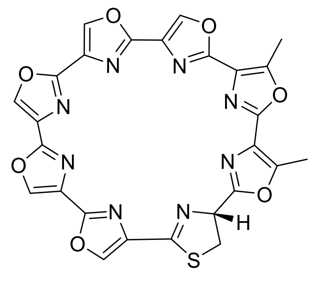

telomestatin

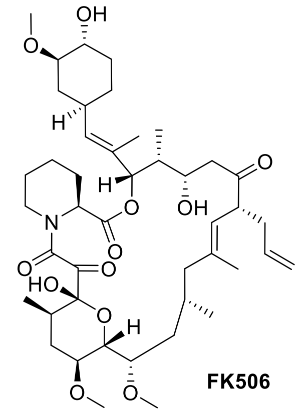

FK506

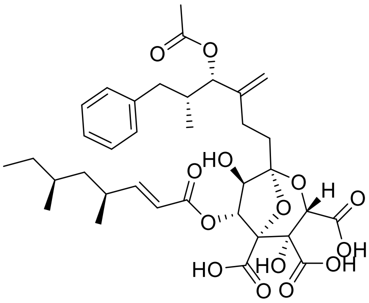

zaragozic acid A

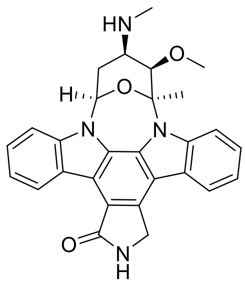

staurosporine

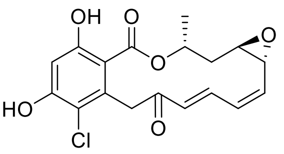

radicicol

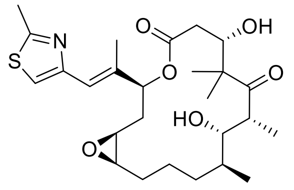

epothilone A

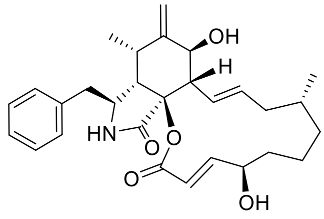

cytochalasin B

Table S3
Normalised PMI ratio (npr) values of conformers the DOS library with the lowest energy (energy level = 0 kcal/mol).

Compound	npr1	npr2
**9aC**	0.325912	0.80446
**9aB**	0.383799	0.810257
**9aA**	0.376249	0.783016
**9aD**	0.335695	0.821046
**9gA**	0.383033	0.776198
**9bA**	0.470873	0.69701
**9cA**	0.488971	0.665658
**9hA**	0.369411	0.793983
**9dA**	0.518106	0.824772
**9eA**	0.445111	0.695143
**9fA**	0.409042	0.780497
**9iA**	0.336815	0.774293
**5kB**	0.359148	0.823546
**5hB**	0.424372	0.794063
**5mB**	0.531798	0.712014
**5aB**	0.489386	0.743327
**5lB**	0.439253	0.771686
**6oA**	0.281595	0.813335
**6gA**	0.366694	0.742862
**6nA**	0.222281	0.857253
**18**	0.330134	0.911499
**19**	0.534916	0.692087
**20**	0.423576	0.836702
**16lA**	0.536893	0.716695
**16mA**	0.438995	0.697837
**16aB**	0.38762	0.713581
**16hB**	0.374193	0.727747
**16lB**	0.338537	0.758569
**16mB**	0.409253	0.696073
**17aA**	0.430651	0.792407
**17aB**	0.605241	0.757192
**6aA**	0.435848	0.661454
**10aA**	0.456618	0.957579
**Z - 5pA**	0.56481	0.773025
**E - 5pA**	0.441691	0.781744
**Z -5qA**	0.414529	0.864167
**E-5qA**	0.458075	0.73801
**5kA**	0.459637	0.843805
**5ha**	0.528161	0.819399
**5lA**	0.580163	0.800972
**5mA**	0.655226	0.753116
**5aA**	0.615298	0.775393
**5cA**	0.43385	0.828854
**5bA**	0.526538	0.788566
**5jA**	0.626913	0.834981
**5gA**	0.635945	0.779043

**16aA**	0.61757	0.740542
**16hA**	0.405866	0.721253
**8b**	0.387198	0.909784
**7**	0.638487	0.826468

**5nA**	0.476834	0.83236
**8a**	0.395942	0.868401
**8c**	0.369394	0.820057

Table S4
Normalised PMI ratio (npr) values of conformers of 40 top selling drugs with the lowest energy (energy level = 0 kcal/mol).

Compound	npr1	npr2
**Lipitor**	0.3343	0.8427
**Nexium**	0.2387	0.7858
**Prevacid**	0.1367	0.9103
**Flonase**	0.2843	0.9666
**Servent**	0.8749	0.9282
**Singulair**	0.3979	0.7155
**Effexor**	0.3994	0.7418
**Plavix**	0.3507	0.8350
**Zocor**	0.3846	0.7750
**Norvasc**	0.4396	0.8183
**Lexapro**	0.4172	0.7481
**Seroquel**	0.2078	0.9130
**Protonix**	0.2323	0.8070
**Ambien**	0.3818	0.6870
**Actos**	0.1733	0.8826
**Zoloft**	0.3094	0.9498
**Wellbutrin**	0.1861	0.9472
**Avandia**	0.0876	0.9585
**Risperdal**	0.2654	0.7797
**Zyprexa**	0.4262	0.6254

Compound	npr1	npr2
**Topomax**	0.3721	0.7907
**Toprol**	0.0854	0.9449
**Zetia**	0.3674	0.8320
**Fosamax**	0.6565	0.7739
**Ability**	0.4836	0.6354
**Levaquin**	0.2100	0.8459
**Lamictal**	0.2412	0.9155
**Celebrex**	0.3738	0.6824
**Benazepril**	0.3379	0.9290
**Zyrtec**	0.3208	0.8402
**Coreg**	0.6401	0.7545
**Valtrex**	0.4538	0.8509
**Adderall**	0.2184	0.9253
**Aciphex**	0.1239	0.9138
**Cymbalta**	0.3327	0.7663
**Crestor**	0.3525	0.8687
**Diovan**	0.3509	0.9594
**Tricor**	0.1028	0.9422
**Concerta**	0.5477	0.6565
**Imitrex**	0.2068	0.9075

Table S5
Normalised PMI ratio (npr) values of conformers of 60 natural products with the lowest energy (energy level = 0 kcal/mol).

**Compound**	**npr1**	**npr2**
**Taxol**	0.4444	0.7558
**Actinonin**	0.4418	0.7805
**Discodermolide**	0.1283	0.9329
**Validamycin**	0.2010	0.9501
**Monensin**	0.3209	0.8721
**Calyculin A**	0.4042	0.9305
**Coformycin**	0.3093	0.8134
**Arglabin**	0.3932	0.6626
**Mizoribine**	0.2479	0.8433
**Forskolon**	0.5081	0.7477
**SQ 26180**	0.3285	0.9244
**Cephamycin C**	0.5613	0.6949
**Avermectin B1a**	0.3723	0.8151
**Adriamycin**	0.3135	0.7704
**Phorbol myristate acetate**	0.4660	0.7501
**Thienamycin**	0.3015	0.8545
**Cyclosporin A**	0.4809	0.8960
**FK506**	0.4472	0.8793
**Trapoxin B**	0.7165	0.9000
**Vincristine**	0.5370	0.9655
**Colchicine**	0.4272	0.8346
**Trichostatin A**	0.2197	0.8615

**Compound**	**npr1**	**npr2**
**Ginkgolide B**	0.4546	0.8718
**Vancomycin**	0.5097	0.6634
**Amphotericin B**	0.1342	0.9067
**Radicicol**	0.4995	0.8727
**Salicylihalamide A**	0.1935	0.8944
**Telomestatin**	0.4927	0.5148
**Rifamycin B**	0.4922	0.7587
**Apoptolidin**	0.1922	0.8755
**Midecamycin A1**	0.3650	0.9474
**Zaragozic acid A**	0.5011	0.7235
**Talaromycin B**	0.1546	0.9504
**Spongistatin 1**	0.4968	0.8135
**Brevetoxin B**	0.0410	0.9818
**Quinine**	0.3647	0.8711
**Mycobactin S**	0.3865	0.9065
**Duocarmycin A**	0.1237	0.9519
**Bleomycin A2**	0.3651	0.9343
**Brefeldin A**	0.3068	0.7850
**Cytochalasin B**	0.4974	0.6762
**Epothilone A**	0.3116	0.8340
**Lactacystin**	0.3764	0.8347
**Calicheamicin γ1**	0.1774	0.9247

**Fumagillin**	0.0865	0.9668	**Artemisinin**	0.5476	0.6380
**Staurosporine**	0.4822	0.6733	**Compactin**	0.3930	0.7646
**Erythromycin A**	0.4902	0.7797	**Lipstatin**	0.4059	0.8457
**Streptomycin**	0.3162	0.9282	**Pseudomonic acid A**	0.3896	0.6714
**Penicillin G**	0.3061	0.9575	**Daptomycin**	0.5603	0.8611
**Sperguallin**	0.2793	0.8633	**Bestatin**	0.3910	0.7358
**Rapamycin**	0.6347	0.8330	**Plaunotol**	0.4702	0.6467
**Echinocandin B**	0.6140	0.8022	**Geldanamycin**	0.3478	0.7321

Figure S2
PMI plot of the DOS library alone (red dots).The DOS compound library exhibited a broad shape distribution with limited 'rod-like' and 'disk-like' features as shown by the absence of compounds within these extreme areas of the plot.

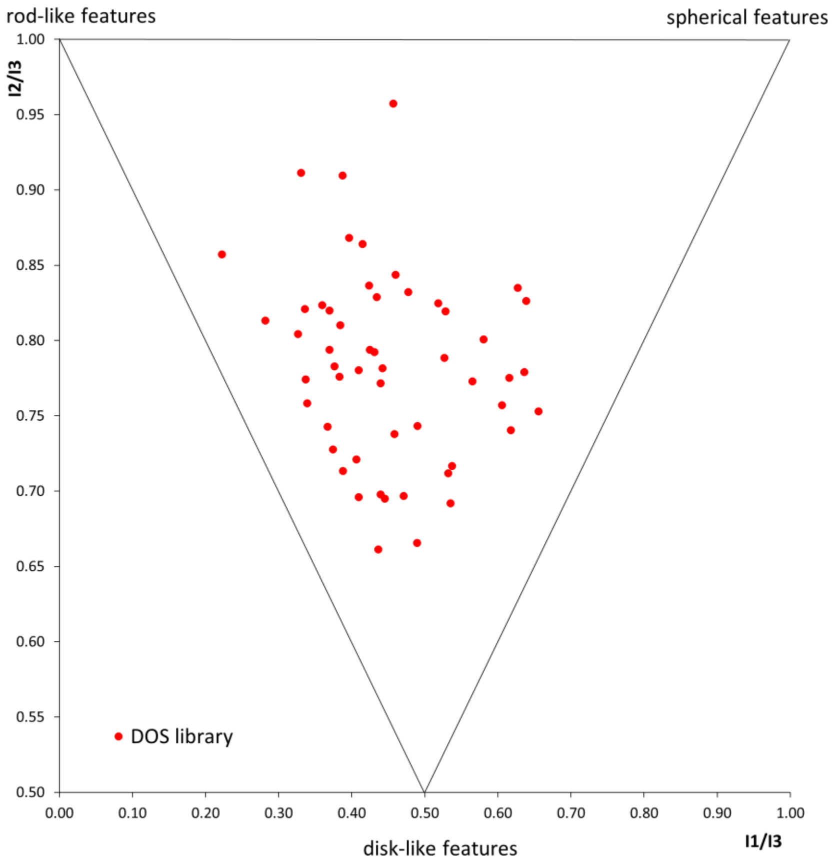

B) Principal component analysis

Principal component settings

Weight field	None
Prefix	PCA
Component limit	0
Minimum variance (%)	95
Condition limit	1e+006

Structural and physicochemical parameters used in PCA

**Parameter**	**Description**	**2D or 3D**
ASA_H	Total hydrophobic surface area	3D
ASA_P	Total polar surface area	3D
a_acc	Number of hydrogen bond acceptor atoms	2D
a_aro	Number of aromatic atoms	2D
a_don	Number of hydrogen bond donor atoms	2D
a_nN	Number of nitrogen atoms	2D
a_nO	Number of oxygen atoms	2D
b_rotN	Number of rotatable bonds	2D
chiral	Number of chiral centres	2D
KierFlex	Molecular flexibility	2D
logS	Log solubility in water	2D
mr	Molar refractivity	2D
rings	Number of rings	2D
SlogP	Log octanol/water partition coefficient	2D
TPSA	Topological polar surface area (A2)	2D
vol	Van der Waals volume	3D
weight	Molecular weight	2D

Variance

PC#	Deviation[a]	Condition[b]	Proportion of variance	% Variance[c]
1	3.280	1.000	63.267	63.267
2	1.533	4.574	77.099	77.099
3	1.305	6.311	87.123	87.123
4	0.954	11.811	92.480	92.480
5	0.577	32.361	94.435	94.435
6	0.500	43.022	95.905	95.905

[a] The standard deviation of the data along the principal component vector.

[b] Condition number of the covariance matrix if the principal component list were terminated at that row.

[c] Percentage of the variance retained if the component list were truncated at that row.

Component loadings

Component loadings for PCA of DOS library with three reference sets

Descriptors	PC1	PC2	PC3	PC4	PC5	PC6
ASA_H	0.0003	-0.0011	0.0008	-0.0009	0.0014	-0.0013
ASA_P	0.0006	0.0012	-0.0008	0.0017	-0.0032	0.0017
KierFlex	0.0137	-0.0005	0.0225	-0.0458	0.0232	0.0273
SlogP	-0.0100	-0.0914	0.0637	-0.0101	-0.0192	0.1591
TPSA	0.0007	0.0010	-0.0007	0.0002	0.0011	0.0010
Weight	0.0003	-0.0002	0.0001	-0.0001	0.0005	-0.0004
a_acc	0.0187	0.0112	0.0307	0.0448	0.0970	0.1759
a_aro	0.0020	-0.0285	-0.0597	0.0132	0.0238	0.1274
a_don	0.0221	0.0453	-0.0239	0.0111	0.0591	0.1555
a_nN	0.0191	0.0137	-0.0894	-0.0723	0.1661	-0.2409
a_nO	0.0172	0.0113	0.0288	0.0387	-0.0137	0.0802
b_1rotN	0.0102	0.0079	-0.0124	-0.0555	-0.1829	0.0183
chiral	0.012	0.0143	0.0481	0.0602	-0.0312	-0.0640
logS	-0.0239	0.0971	0.0125	0.0542	0.1427	0.0166
mr	0.0116	-0.0129	0.0018	-0.0107	0.0220	-0.0274
rings	0.0227	-0.1089	-0.0826	0.3502	-0.1863	-0.3542
vol	0.0003	-0.0003	0.0002	-0.0003	0.0007	-0.0008

Top contributing parameters to each principal component are marked in grey, the darker grey, the more contribution in each column. The values were normalised automatically by the MOE software.

PCA values for compounds

Compound	PC1	PC2	PC3
**9aC**	-0.5214	-0.2195	-0.3267
**9aB**	-0.7266	-0.1262	-0.3089
**9aA**	-0.6802	-0.3079	-0.2161
**9aD**	-0.5991	-0.1316	-0.4027
**9gA**	-0.8790	0.5662	0.0089
**9bA**	-0.5895	-0.1697	-0.2442
**9cA**	-0.5975	-0.3272	-0.2948
**9hA**	-0.5479	0.0273	-0.4913
**9dA**	-0.6144	-0.4545	-0.1918
**9eA**	-0.4555	-0.7735	-0.4649
**9fA**	-0.7066	-0.1556	-0.2089
**9iA**	-0.7148	0.3593	0.1499
**9kB**	-0.4369	-0.6563	0.1295
**5hB**	-0.3450	-0.2454	-0.1697
**5mB**	-0.5323	-0.3972	0.0104
**5aB**	-0.4968	-0.5175	0.0582
**5iB**	-0.4163	-0.3524	0.0562
**6oA**	-0.6286	-0.0091	0.5635
**6gA**	-0.7433	-0.0647	0.5260
**6nA**	-0.6317	-0.1307	0.4837
**18**	-1.1323	0.4261	0.0683
**19**	-0.9795	-0.2188	0.1370
**20**	-1.1207	0.5723	-0.1543
**16aA**	-0.3484	-1.0900	0.4378

Compound	PC1	PC2	PC3
**16hA**	-0.2855	-0.6662	0.1238
**16iA**	-0.2742	-0.8496	0.3795
**16mA**	-0.3785	-0.9328	0.3638
**16aB**	-0.4657	-0.5294	0.1615
**16hB**	-0.4136	-0.1130	-0.1495
**16iB**	-0.3831	-0.3272	0.1335
**16mB**	-0.5036	-0.3778	0.0911
**17aA**	-0.7542	-0.1569	0.4712
**17aB**	-0.8635	0.3808	0.2209
**6aA**	-0.5446	-0.9055	0.2727
**10aA**	-0.5713	-0.7594	0.0720
**Z - 5pA**	-0.1324	-1.0068	0.1652
**E - 5pA**	-0.1358	-1.0485	0.1950
**Z -5qA**	-0.0617	-0.8532	0.1254
**E-5qA**	-0.0624	-0.9052	0.1629
**5kA**	-0.3156	-1.2007	0.3925
**5ha**	-0.2236	-0.7952	0.1025
**5iA**	-0.2943	-0.8950	0.3211
**5mA**	-0.4092	-0.9431	0.2789
**5aA**	-0.3728	-1.0694	0.3317
**5cA**	-0.2584	-1.1320	0.2879
**5bA**	-0.2570	-1.0105	0.3662
**5jA**	-0.3476	-0.1976	0.6000
**5gA**	-0.5645	-0.2193	0.5797

**5nA**	-0.4541	-0.2879	0.5388	**8c**	-0.5732	-0.4883	-0.3280
**8a**	-0.7234	-0.5520	-0.2265	**7**	-0.7575	-0.5003	-0.2765
**8b**	-0.7737	-0.3775	-0.3151				

PCA for 40 drugs

**Compound**	**PC1**	**PC2**	**PC3**	**Compound**	**PC1**	**PC2**	**PC3**
**Lipitor**	-0.0037	-1.1465	-0.8868	**Zyprexa**	-0.8492	0.1473	-0.6153
**Nexium**	-0.5678	0.1034	-0.7787	**Topomax**	-0.5336	1.1929	0.4201
**Prevacid**	-0.5803	0.0609	-0.9010	**Toprol**	-0.7806	0.8399	0.0456
**Flonase**	-0.3292	0.1231	0.8602	**Zetia**	-0.5320	-0.6198	-0.6027
**Servent**	-0.3252	0.1990	-0.2285	**Fosamax**	-0.8807	2.8344	-0.3950
**Singulair**	-0.1032	-1.9681	-0.6143	**Ability**	-0.5155	-0.5341	-0.5628
**Effexor**	-0.8758	0.5244	-0.0503	**Levaquin**	-0.6743	0.6963	-0.6524
**Plavix**	-0.8666	-0.1560	-0.3177	**Lamictal**	-0.8627	0.6634	-1.1629
**Zocor**	-0.4764	0.1177	0.9703	**Celebrex**	-0.6087	-0.1229	-1.1103
**Norvasc**	-0.4489	0.4731	-0.0759	**Benazepril**	-0.4176	0.0697	-0.4933
**Lexapro**	-0.7821	-0.0974	-0.5671	**Zyrtec**	-0.5837	0.1466	-0.6502
**Seroquel**	-0.5533	-0.1231	-0.7516	**Coreg**	-0.4112	-0.3704	-0.9588
**Protonix**	-0.4622	0.3592	-0.8673	**Valtrex**	-0.5082	1.5022	-0.8324
**Ambien**	-0.8398	-0.1799	-0.5196	**Adderall**	-1.2938	0.9066	-0.2386
**Actos**	-0.6492	-0.1327	-0.4748	**Aciphex**	-0.4920	-0.0007	-0.7167
**Zoloft**	-0.9531	-0.5022	-0.2830	**Cymbalta**	-0.8527	-0.4412	-0.5503
**Wellbutrin**	-1.0484	0.3578	0.0618	**Crestor**	-0.1769	0.5329	-0.5057
**Avandia**	-0.6430	0.0749	-0.6547	**Diovan**	-0.3562	-0.4047	-1.1559
**Risperdal**	-0.5860	-0.4524	-0.5219	**Tricor**	-0.6959	-0.5221	0.0315

**Concerta**	-0.9994	0.6400	-0.1029

**Imitrex**	-0.7704	1.0155	-0.7653

PCA for Natural products

**Compound**	**PC1**	**PC2**	**PC3**
**Taxol**	0.9255	-1.0677	0.1006
**Actinonin**	-0.3623	1.0510	0.2595
**Discodermolide**	0.3615	0.6786	1.4436
**Validamycin**	0.4678	3.4146	0.2918
**Monensin**	0.4428	0.0458	1.7028
**Calyculin A**	1.6078	0.2816	1.1994
**Coformycin**	-0.5618	2.0187	-0.7029
**Arglabin**	-0.9922	0.6135	0.3881
**Mizoribine**	-0.5841	2.2410	-0.5818
**Forskolon**	-0.4110	0.7926	0.8393
**SQ 26180**	-0.8937	2.0443	-0.2113
**Cephamycin C**	-0.1206	2.2535	-0.6028
**Avermectin B1a**	1.0120	-0.6629	2.2568
**Adriamycin**	0.2184	0.8786	-0.3825
**Phorbolmyristate acetate**	0.2485	-0.5175	1.2169
**Thienamycin**	-0.7759	1.7844	-0.2599
**Cyclosporin A**	1.8259	-0.5744	1.4331
**FK506**	0.7485	-0.1863	1.8816
**Trapoxin B**	0.1366	-0.3212	-0.4482
**Vincristine**	0.7535	-0.8349	-0.6452
**Colchicine**	-0.4722	0.3530	0.0514
**Trichostatin A**	-0.7478	0.6134	-0.1741
**Fumagillin**	-0.2143	0.1150	0.8304

**Staurosporine**	-0.3240	-1.2130	-1.2835
**Compound**	**PC1**	**PC2**	**PC3**
**Erythromycin A**	0.6930	0.8578	1.8669
**Streptomycin**	0.7382	3.8176	-0.6451
**Penicillin G**	-0.6513	0.7769	-0.4131
**Sperguallin**	-0.0253	2.7539	-0.8072
**Rapamycin**	0.9832	-0.4266	2.1270
**Echinocandin B**	2.0483	0.9687	0.3183
**Ginkgolide B**	-0.2213	1.1637	0.3873
**Vancomycin**	3.2072	-0.1680	-1.5775
**Amphotericin B**	1.4367	1.4064	1.8718
**Radicicol**	-0.6241	0.4572	0.1867
**Salicylihalamide A**	-0.3265	-0.0188	0.4357
**Telomestatin**	0.2538	-2.0052	-2.6016
**Rifamycin B**	0.6374	-0.1313	0.7220
**Apoptolidin**	2.1198	0.0404	3.0226
**Midecamycin A1**	0.8984	0.5235	1.9927
**Zaragozic acid A**	0.6605	0.5951	0.5646
**Talaromycin B**	-0.9077	1.1635	0.5006
**Spongistatin 1**	2.0595	-0.5571	2.7758
**Brevetoxin B**	1.0300	-1.4491	2.1498
**Quinine**	-0.6858	0.2372	-0.4269
**Mycobactin S**	1.0595	-1.1841	0.5053

**Duocarmycin A**	-0.0340	0.1944	-0.5793
**Bleomycin A2**	3.6391	2.5659	-2.0158
**Compound**	**PC1**	**PC2**	**PC3**
**Brefeldin A**	-0.8333	0.9548	0.6341
**Cytochalasin B**	-0.2636	-0.0814	0.3929
**Epothilone A**	-0.2908	0.1136	0.8195
**Lactacystin**	-0.3472	1.9207	0.1130
**Calicheamicin γ1**	2.6030	-0.6085	1.5956
**Artemisinin**	-0.8297	0.4450	0.6876

**Compactin**	-0.5225	0.3140	0.8956
**Compound**	**PC1**	**PC2**	**PC3**
**Lipstatin**	0.0115	-0.4049	1.0584
**Pseudomonic acid A**	0.0671	0.9242	0.9823
**Daptomycin**	3.8774	1.8931	-1.7702
**Bestatin**	-0.6043	1.3052	-0.3592
**Plaunotol**	-0.6902	0.4533	0.7347
**Geldanamycin**	0.0815	0.7974	0.8208

Figure S3

PCA of DOS library and two reference libraries. a) PC1 *versus* PC2, b) PC1 *versus* PC3 and c) PC2 *versus* PC3. The DOS library (red dots), 40 drugs (blue triangles) and natural products (green rhombus).

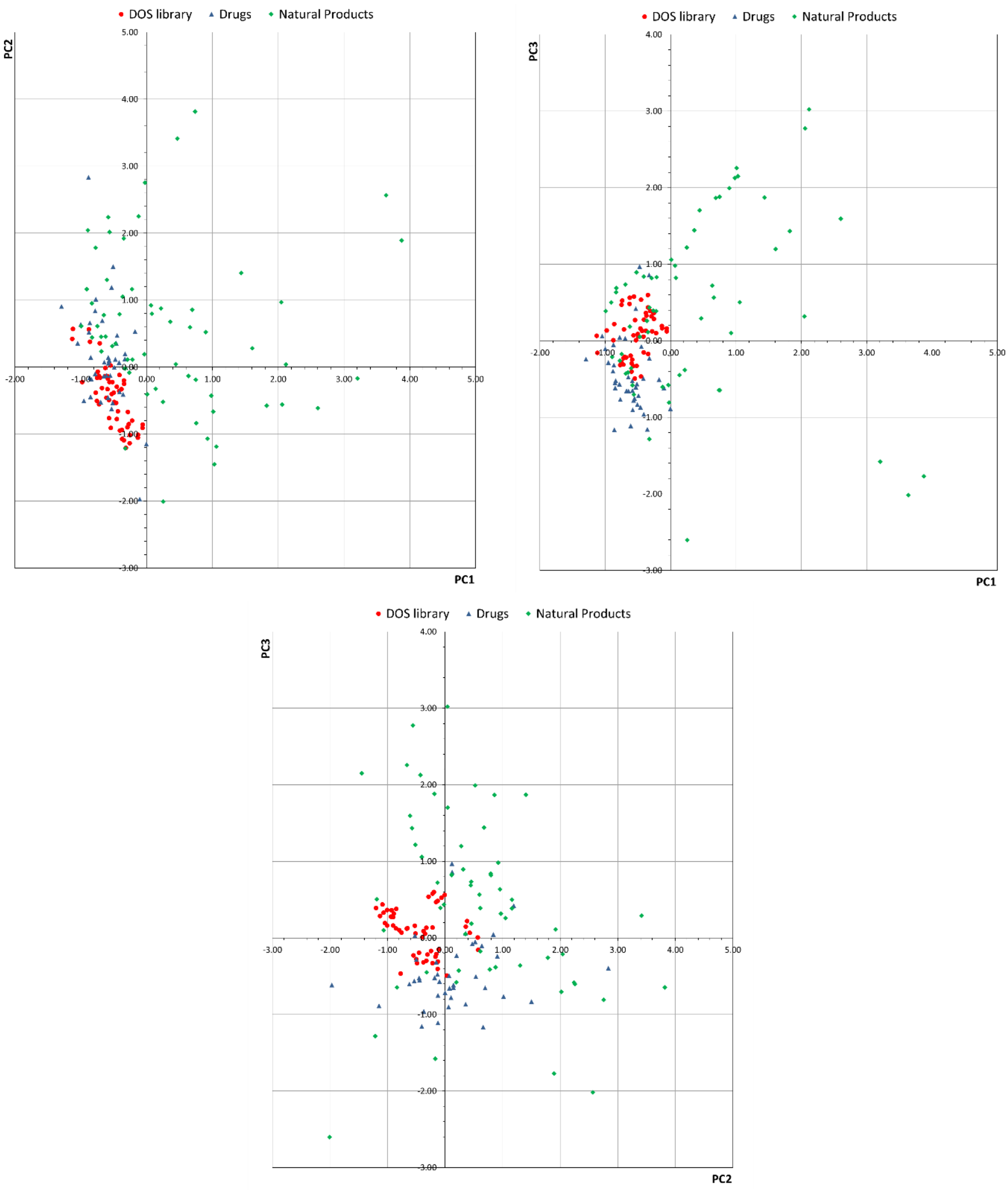

Figure S3 suggests the DOS library has significant overlap with both reference sets. In particular, the overlap with both reference sets is greatest Figures S3a and S3b, suggesting PCA1 contributed more favourably to the overlap. This hypothesis is strengthened by the reduced overlap between the DOS library and reference sets in Figure S3c showing PCA2 *versus* PCA3.

19. References

1 (a) A.-R. Abdun-Nur and C. H. Issidorides, *J. Org. Chem.*, 1962, **27**, 67-70; (b) G. Wuitschik, M. Rogers-Evans, A. Buckl, M. Bernasconi, M. Märki, T. Godel, H. Fischer, B. Wagner, I. Parrilla, F. Schuler, J. Schneider, A. Alker, W. B. Schweizer, K. Müller and E. M. Carreira, *Angew. Chem. Int. Ed.*, 2008, **47**, 4512-4515; (c) E. M. Carreira and T. C. Fessard, *Chem. Rev.*, 2014, **114**, 8257-8322.

2 L. S. Campbell-Verduyn, L. Mirfeizi, R. A. Dierckx, P. H. Elsinga and B. L. Feringa, *Chem. Commun.*, 2009, 2139-2141.

3 J. Raushel and V. V. Fokin, *Org. Lett.*, 2010, **12**, 4952-4955.

4 J. S. Alford and H. M. L. Davies, *Org. Lett.*, 2012, **14**, 6020-6023.

5 M. J. Frisch, G. W. Trucks, H. B. Schlegel, G. E. Scuseria, M. A. Robb, J. R. Cheeseman, G. Scalmani, V. Barone, B. Mennucci, G. A. Petersson, H. Nakatsuji, M. Caricato, X. Li, H. P. Hratchian, A. F. Izmaylov, J. Bloino, G. Zheng, J. L. Sonnenberg, M. Hada, M. Ehara, K. Toyota, R. Fukuda, J. Hasegawa, M. Ishida, T. Nakajima, Y. Honda, O. Kitao, H. Nakai, T. Vreven, J. A. Montgomery, J. E. Peralta, F. Ogliaro, M. Bearpark, J. J. Heyd, E. Brothers, K. N. Kudin, V. N. Staroverov, R. Kobayashi, J. Normand, K. Raghavachari, A. Rendell, J. C. Burant, S. S. Iyengar, J. Tomasi, M. Cossi, N. Rega, J. M. Millam, M. Klene, J. E. Knox, J. B. Cross, V. Bakken, C. Adamo, J. Jaramillo, R. Gomperts, R. E. Stratmann, O. Yazyev, A. J. Austin, R. Cammi, C. Pomelli, J. W. Ochterski, R. L. Martin, K. Morokuma, V. G. Zakrzewski, G. A. Voth, P. Salvador, J. J. Dannenberg, S. Dapprich, A. D. Daniels, Farkas, J. B. Foresman, J. V. Ortiz, J. Cioslowski and D. J. Fox, Wallingford CT, 2009.

6 (a) H. L. Schmider and A. D. Becke, *J Chem Phys*, 1998, **108**, 9624-9631; (b) J. P. Perdew, *Phys. Rev. B.*, 1986, **33**, 8822-8824.

7 D. Andrae, U. Häußermann, M. Dolg, H. Stoll and H. Preuß, *Theor. Chim. Acta*, 1990, **77**, 123-141.

8 A. Höllwarth, M. Böhme, S. Dapprich, A. W. Ehlers, A. Gobbi, V. Jonas, K. F. Köhler, R. Stegmann, A. Veldkamp and G. Frenking, *Chem. Phys. Lett.*, 1993, **208**, 237-240.

9 R. Krishnan, J. S. Binkley, R. Seeger and J. A. Pople, *J. Chem. Phys.*, 1980, **72**, 650-654.

10 CrysalisPro Software system, Agilent Technologies UK Ltd., Oxford, UK.

11 G. M. Sheldrick, *Acta Crystallogr. A*, 2008, **64**, 112-122.

12 M. C. Burla, R. Caliandro, M. Camalli, B. Carrozzini, G. L. Cascarano, L. De Caro, C. Giacovazzo, G. Polidori and R. Spagna, *J. Appl. Crystallogr.*, 2005, **38**, 381-388.

13 O. V. Dolomanov, L. J. Bourhis, R. J. Gildea, J. A. K. Howard and H. Puschmann, *J. Appl. Crystallogr.*, 2009, **42**, 339-341.
